# Association between blood pressure and risk of cancer development: a systematic review and meta-analysis of observational studies

**DOI:** 10.1038/s41598-019-45014-4

**Published:** 2019-06-12

**Authors:** Aristeidis Seretis, Sofia Cividini, Georgios Markozannes, Xanthippi Tseretopoulou, David S. Lopez, Evangelia E. Ntzani, Konstantinos K. Tsilidis

**Affiliations:** 1 Department of Hygiene and Epidemiology, University of Ioannina School of Medicine, Ioannina, Greece; 2 Independent Researcher in Biostatistics, Como, Italy; 3 The University of Texas School of Public Health, Houston, TX USA; 4 Center for Evidence-Based Medicine, Department of Health Services, Policy and Practice, School of Public Health, Brown University, Providence, RI USA; 5 Department of Epidemiology and Biostatistics, The School of Public Health, Imperial College London, London, UK

**Keywords:** Cancer epidemiology, Hypertension

## Abstract

With the exception of renal cell carcinoma, studies assessing the association between hypertension and other cancers are inconsistent. We conducted a meta-analysis to assess this evidence. We included observational studies investigating the association between any definition of hypertension or systolic and diastolic blood pressure and risk of any cancer, after searching PubMed until November 2017. We calculated summary relative risks (RR) and 95% confidence intervals (CI) using inverse-variance weighted random effects methods. A total of 148 eligible publications were identified out of 39,891 initially screened citations. Considering only evidence from 85 prospective studies, positive associations were observed between hypertension and kidney, colorectal and breast cancer. Positive associations between hypertension and risk of oesophageal adenocarcinoma and squamous cell carcinoma, liver and endometrial cancer were also observed, but the majority of studies did not perform comprehensive multivariable adjustments. Systolic and diastolic blood pressure were positively associated with risk of kidney cancer but not with other cancers. In addition to the previously well-described association between hypertension and risk of kidney cancer, the current meta-analysis suggested that hypertensive individuals may also be at higher risk of colorectal and breast cancer. However, careful interpretation is required as most meta-analyses included relatively small number of studies, several relative risks had weak or moderate magnitude and maybe affected by residual confounding.

## Introduction

Hypertension and cancer are two multifactorial, severe and chronic conditions. Hypertension is a major health problem worldwide, as it affects approximately 3 in 10 adults over age 20, leading to high morbidity and mortality^1^. It is responsible for almost 50% of heart disease, stroke, and heart failure and about 14% of total deaths in 2015 were related to hypertension^2^. In addition, an estimated 10% of the healthcare spending is directly related to increased blood pressure and its complications^3^.

Cancer is also a leading cause of morbidity and mortality. Worldwide, there were approximately 18 million new cases and 9.6 million cancer-related deaths in 2018, and these numbers are expected to rise within the next two decades^4^. Therefore, if blood pressure and/or hypertension are associated with the risk of cancer, this may have important public health consequences.

Renal cell carcinoma is the only cancer type that has been consistently associated with hypertension^5,6^, although it is not clear yet whether reverse causation explains part or all of this association^7^. Claims of association also exist for colorectal, breast, endometrial and prostate cancer^8–12^, but the evidence is inconsistent. But recently there has been an influx of new studies investigating the association of metabolic syndrome, which includes hypertension, and cancer risk^9,13,14^, and therefore we conducted a systematic review and meta-analysis to summarize and evaluate this literature.

## Methods

### Literature search and data extraction

We searched for eligible published studies in PubMed from inception to November 2017 using the following search algorithm: (blood pressure OR hypertension OR “metabolic syndrome”) AND (cancer OR neoplas* OR malignan* OR tumor OR carcinoma), and also hand-searched for potential missed studies in the references of eligible systematic or narrative reviews^9,10,15–24^. We considered all cohort and case-control studies conducted in humans assessing the association between systolic blood pressure (SBP) or diastolic blood pressure (DBP) or hypertension and risk of any cancer development or cancer death. Hypertension was defined as any of the following: self-reported or measured SBP or DBP higher than predefined cut-off points (e.g. WHO or NCEP/ATP III criteria), self-reported medical history of hypertension or drug-treated hypertension. When the exposure was metabolic syndrome, we only used the hypertension component. We excluded narrative or systematic reviews, case-series or case-reports, cross-sectional studies, prognostic studies among cancer patients, studies in children, cohort studies with a sample size of less than 100 total individuals, case-control studies with less than 100 cancer cases, studies that did not provide enough information for calculating measures of association, studies in other than the English language and studies of pre-malignant outcomes (e.g., colorectal adenomas). Whenever published reports pertained to the same cohort evaluated at different follow-up periods, we retained the publication with the longer follow-up.

We recorded information from each eligible study on author name, year and journal of publication, study design, sample size, population characteristics, definition of hypertension, relative risk (RR), 95% confidence interval (CI) and adjustments for confounders. When results from multiple statistical models were reported, we always retained the most adjusted model. The literature search and data extraction were performed independently by two investigators (AS and XT), and data was re-checked for consistency by a third author (SC).

We used the “Newcastle-Ottawa Scale” to assess the quality of the included studies, and this task was performed independently by two investigators (AS and SC), and disagreements were resolved by a third author (KKT). Based on this tool, every study was judged on eight quality items, grouped into three categories: i) selection, ii) comparability of study groups and iii) exposure or outcome ascertainment for case-control and cohort studies, respectively. A star was awarded for every quality item for a maximum of 9 stars for the highest quality studies^25^.

### Statistical analyses

Our primary analysis included only prospective studies (e.g., cohort or nested case-control designs), whereas analyses including case-control studies are provided in the online supplement. We conducted separate meta-analyses according to systolic or diastolic blood pressure and hypertension. Analyses for systolic and diastolic blood pressure used both a top to bottom category comparison approach and a dose-response approach per 10 mmHg. Studies that reported a dose-response estimate were either pooled directly or after re-scaling to the corresponding increment unit using the generalized weighted least-squares regression model approach^26,27^. We summarized RRs and 95% CIs using fixed-effects and random-effects meta-analysis models, if three or more studies were available per exposure and outcome comparison^28^. Between-study heterogeneity was assessed by the Cochran’s Q test and the I^2^ statistic^29^. 95% prediction intervals were also calculated to further assess heterogeneity, which represents the range in which the effect estimates of future studies will lie^30^. Subgroup meta-analyses were conducted according to sex, study design (prospective vs. case-control) and adjustment factors (at least age vs. age plus further multivariable adjustment). Presence of small-study effects was assessed using the Egger’s regression asymmetry test^31^. Analyses were performed in STATA 12 (College Station, Texas). All p-values were two-tailed. The study is reported according to the MOOSE checklist^32^.

## Results

### Study characteristics

**Figure 1:**
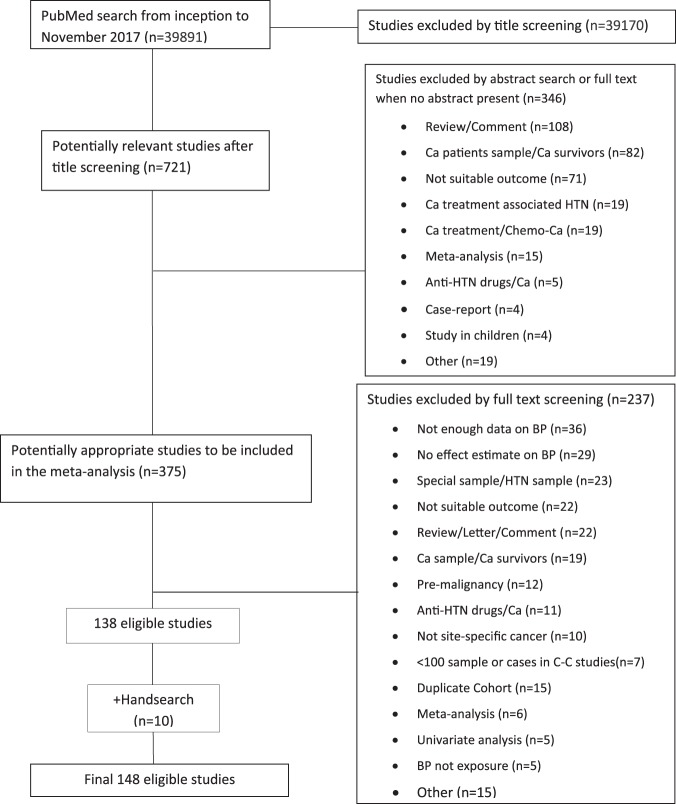
Flow diagram of studies assessed for eligibility per screening stage. Abbreviations: Ca, cancer; HTN, hypertension; BP, blood pressure; C–C, case-control.

Figure 1 presents the meta-analysis flowchart. A total of 148 individual studies met the eligibility criteria out of the 39,891 initially screened citations. Supplemental Table 1 provides a detailed description of the characteristics of all included studies. Specifically, we included 85 prospective studies^14,33–116^, 72 of which were cohort studies, 11 were nested case-control studies^33,34,36,49,50,67,68,89,94,96,111^ and 2 were record-linkage studies^76,79^, and 63 case-control studies^13,117–178^. Most studies (n = 133; 90%) investigated associations with cancer incidence. A total of 48 (32%) studies were conducted only in men, whereas 57 (39%) studies were conducted only in women. The majority of the studies (n = 51; 34%) were conducted in the USA followed by Scandinavian countries (n = 15; 10%), Italy (n = 13; 9%), UK and Korea (n = 7; 5%), and finally Japan with 6 studies (4%). Out of the 128 studies that used hypertension as the exposure of interest, this was defined in 20 studies (16%) using the NCEP-ATPIII criteria (≥130/85 mmHg), 17 studies (13%) used the WHO definition (≥140/90 mmHg), 4 studies (3%) used ≥160/95 mmHg, 38 studies (30%) pertained to self-reported hypertension, 16 studies (13%) used self-reported drug treatment for hypertension and 26 studies (20%) used a combination of self-reported disease and treatment, whereas 7 studies used other definitions.

Detailed information on the quality assessment for each study, using the Newcastle-Ottawa scale, can be found on Supplementary Table 2. For prospective studies, the median number of stars per study was 7, and the interquartile range (IQR) was 1. Case-control studies scored lower with a median of 6 and IQR of 2. However, the minority of prospective (31%) and case-control studies (19%) adjusted for age and three of the following five potential confounders, namely body mass index, smoking, alcohol, physical activity and family history of cancer.

### Evidence Synthesis

For the primary analysis using only prospective studies, we conducted 30 meta-analyses of hypertension and 16 different cancers, and 29 meta-analyses between SBP or DBP and 11 cancers. Summary random effects relative risk estimates and 95% CIs per cancer site, heterogeneity statistics, 95% prediction intervals and tests of small-study effects are summarized in Fig. 2 for hypertension, in Fig. 3 per 10 mmHg of systolic and diastolic blood pressure, and in Supplemental Fig. 1 for top vs. bottom category comparisons of systolic and diastolic blood pressure. Supplemental Table 3 describes in more detail the meta-analyses of prospective studies for the association between hypertension, systolic or diastolic blood pressure and risk of cancer, and Supplemental Table 4 includes meta-analyses of both prospective and case-control studies. Supplemental Figs 2–60 depict all forest plots by cancer site using only prospective studies, and Supplemental Figs 61–79 include both prospective and case-control studies.

**Figure 2:**
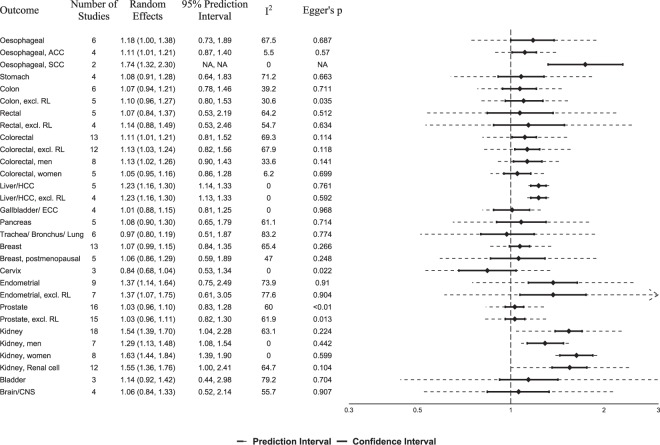
Summary relative risks and 95% confidence intervals of prospective studies for the association between hypertension and cancer risk. Abbreviations: ACC, adenocarcinoma; SCC, squamous cell carcinoma; RL, record-linkage studies; HCC, hepatocellular carcinoma; ECC, extrahepatic cholangiocarcinoma; CNS, central nervous system, NA, Not applicable.

**Figure 3:**
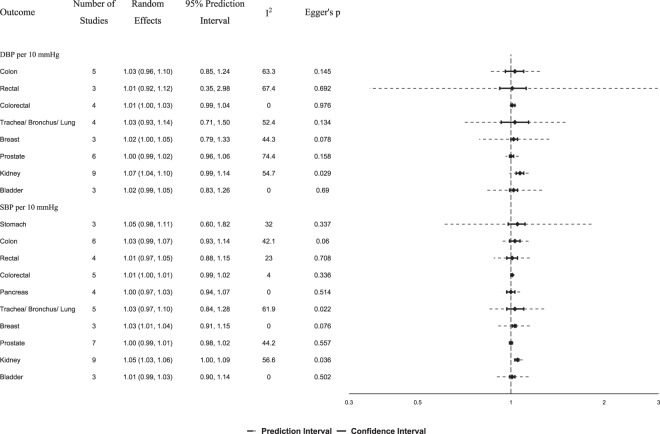
Summary relative risks and 95% confidence intervals of prospective studies for the association between cancer risk and 10 mmHg increase in systolic and diastolic blood pressure. Abbreviations: DBP, diastolic blood pressure; SBP, systolic blood pressure.

We found a statistically significant association between hypertension and risk of kidney cancer (Fig. 2; n = 18 prospective studies; summary random effects RR, 1.54; 95% CI, 1.39–1.70)^37,46,55,56,58,66,67,76,80,83,91–94,99,105,107,109^. We observed large heterogeneity (I^2^, 63.1%), but no indication for small study effects (Supplemental Fig. 26). When we meta-analysed studies clearly mentioning that only renal cell carcinoma cases were used, similar results were obtained (n = 12 studies, RR, 1.55; 95% CI, 1.36–1.76; I^2^, 64.7%)^55,56,58,66,67,80,92–94,105,109,179^. The association between hypertension and kidney cancer was statistically significant in both women (n = 8 studies^55,66,76,83,91,93,94,105^, RR, 1.63; 95% CI, 1.44–1.84; I^2^, 0%) and men (n = 7 studies^46,55,66,76,93,94,105^, RR, 1.29; 95% CI, 1.13–1.48; I^2^, 0%) without between-study heterogeneity within each sex-specific analysis, but considerable larger estimates were observed in women (P-heterogeneity, 0.01). Similar results were obtained in subgroup meta-analyses of prospective studies that at least adjusted for age vs. studies that adjusted for age plus at least three of the following five risk factors: body mass index, smoking, alcohol, physical activity and family history of kidney cancer (Table 1). When 14 case-control studies were meta-analysed together with the prospective studies, a summary RR of 1.60 (95% CI, 1.48–1.73; I^2^, 61.3%) was observed (Supplemental Table 4). We also observed statistically significant associations between SBP (Fig. 3; RR per 10 mmHg, 1.05; 95% CI 1.03–1.06; I^2^, 57%) and DBP (RR, 1.07; 95% CI, 1.04–1.10; I^2^, 55%) with kidney cancer, but these meta-analyses had evidence of small-study effects (Supplemental Figs 40–48; P, 0.03).

**Table 1:** Meta-analysis of prospective studies for the association between hypertension and risk of cancer according to type of adjustments.

Outcome	Age adjustment (at least)		I^2^	Multivariate adjustment*		I^2^
	Studies	Random Effect		Studies	Random Effect	
Oesophageal	5	1.18 (1.00–1.40)	73.0%	1	1.13 (0.58–2.21)	n/a
Oesophageal ACC	3	1.12 (1.02–1.22)	05.7%	1	0.82 (0.46–1.46)	n/a
Oesophageal SCC	1	1.77 (1.30–2.41)	n/a	1	1.62 (0.85–3.08)	n/a
Stomach	3	1.02 (0.88–1.18)	57.9%	1	1.52 (1.16–1.99)	n/a
Colon	4	0.97 (0.86–1.08)	0%	2	1.31 (1.12–1.54)	0%
Colon, excl. RL	3	0.97 (0.83–1.14)	0%	2	1.31 (1.12–1.54)	0%
Rectal	4	1.08 (0.80–1.45)	70.0%	1	1.07 (0.77–1.49)	n/a
Rectal, excl. RL	3	1.16 (0.82–1.63)	62.5%	1	1.07 (0.77–1.49)	n/a
Colorectal	9	1.07 (0.97–1.17)	68.2%	4	1.30 (1.03–1.66)	66.6%
Colorectal, excl. RL	8	1.09 (0.98–1.21)	67.3%	4	1.30 (1.03–1.66)	66.6%
Colorectal, men	5	1.10 (1.03–1.18)	0%	3	1.38 (0.92–2.06)	75.9%
Colorectal, women	4	1.02 (0.93–1.12)	0%	1	1.35 (1.01–1.80)	n/a
Liver/HCC	4	1.23 (1.16–1.30)	0%	1	1.20 (0.81–1.77)	n/a
Liver/HCC, excl. RL	3	1.22 (1.14–1.32)	1.8%	1	1.20 (0.81–1.77)	n/a
Gallbladder/ECC	3	1.01 (0.88–1.16)	0%	1	0.95 (0.56–1.62)	n/a
Pancreas	4	1.12 (0.90–1.40)	64.9%	1	0.94 (0.75–1.18)	n/a
Trachea/Bronchus/Lung	5	0.93 (0.76–1.12)	75.7%	1	1.30 (1.11–1.52)	n/a
Breast	8	1.03 (0.92–1.15)	76.6%	5	1.10 (1.02–1.18)	0%
Breast, postmenopausal	3	0.93 (0.80–1.09)	0%	2	1.38 (1.03–1.85)	0%
Cervix	3	0.84 (0.68–1.04)	0%	0	—	n/a
Endometrial	7	1.44 (1.17–1.76)	76.1%	2	1.05 (0.83–1.34)	0%
Endometrial, excl. RL	5	1.46 (1.09–1.95)	80.1%	2	1.05 (0.83–1.34)	0%
Prostate	13	1.05 (0.97–1.14)	66.9%	3	0.98 (0.90–1.07)	0%
Prostate, excl. RL	12	1.06 (0.96–1.16)	68.9%	3	0.98 (0.90–1.07)	0%
Kidney	14	1.54 (1.36–1.74)	70.5%	4	1.52 (1.32–1.75)	0%
Kidney, men	4	1.25 (1.01–1.55)	30.3%	3	1.40 (1.12–1.74)	0%
Kidney, women	6	1.65 (1.43–1.89)	0%	2	1.54 (1.17–2.04)	0%
Kidney, renal cell	9	1.56 (1.32–1.85)	72.0%	3	1.52 (1.31–1.75)	0%
Bladder	3	1.14 (0.92–1.42)	79.2%	0	—	n/a
Brain/CNS	4	1.06 (0.84–1.33)	55.7%	0	—	n/a

*Studies adjusted for age and at least 3 out of five 5 of the following risk factors: smoking, family history of cancer, BMI, alcohol and physical activity.

Abbreviations: n/a, not applicable; ACC, adenocarcinoma; SCC, squamous cell carcinoma; RL, record-linkage studies; HCC, hepatocellular carcinoma; ECC, extrahepatic cholangiocarcinoma; CNS, central nervous system.

We also noted a positive association between hypertension and colorectal cancer using 13 prospective studies (Fig. 2; RR 1.11; 95% CI, 1.01–1.21; I^2^, 69.3%), an association that remained significant after excluding one record-linkage study (RR 1.13; 95% CI, 1.03–1.24; I^2^, 67.9%)^76^ or after meta-analysing only the four studies that performed multivariable adjustments (Table 1; RR 1.30; 95% CI, 1.03–1.66; I^2^, 66.6%)^35,36,113,114^. This association was statistically significant in men (RR 1.13; 95% CI, 1.02–1.26; I^2^, 33.6%)^36,45,69,98,103,108,113,114^, but not in women (RR 1.05; 95% CI, 0.95–1.16; I^2^, 6.2%)^36,69,103,108,113^. No significant associations were identified between SBP or DBP with colorectal cancer risk (Fig. 3). All meta-analyses for either hypertension or SBP/DBP separately on colon and rectal cancer risk did not yield any statistically significant findings (Figs 2, 3).

The meta-analysis yielded a borderline significant association between hypertension and risk of breast cancer (Fig. 2; n = 13 prospective studies; RR, 1.07; 95% CI, 0.99–1.15; I^2^, 65.4%)^33,34,37,44,65,69,73,76,84,85,87,99,110^, but the association was not significant for post-menopausal breast cancer risk (n = 5 studies; RR, 1.06; 95% CI, 0.86–1.29; I^2^, 47%)^33,34,37,44,87^. However, statistically significant associations were observed for total (n = 5; RR 1.10; 95% CI, 1.02–1.18; I^2^, 0%)^33,34,73,84,85^, and post-menopausal (n = 2; RR 1.38; 95% CI, 1.03–1.85; I^2^, 0%) breast cancer risk in prospective studies that performed multivariable adjustment (Table 1)^33,34^. The meta-analysis was also statistically significant for an increase of 10 mmHg in systolic (Fig. 3; n = 3, RR 1.03; 95% CI, 1.01–1.04; I², 0%), but not diastolic (n = 3, RR 1.02; 95% CI, 1.00–1.05; I², 44.3%) blood pressure^42,70,102^. After including case-control studies, the meta-analyses yielded statistically significant findings for both total (Supplemental Table 4; n = 28; RR, 1.11; 95% CI, 1.04–1.19; I^2^, 70.2%) and post-menopausal disease (n = 11; RR, 1.13; 95% CI, 1.02–1.25; I^2^, 37.5%).

Our meta-analysis showed also a statistically significant association between hypertension and risk of endometrial cancer (Fig. 2; n = 9 prospective studies; RR, 1.37; 95% CI, 1.14–1.64)^37,49,59,76,79,99,104,115,116^, but with substantial heterogeneity (Supplemental Fig. 22; I^2^, 73.9%). After removing two record linkage studies^76,79^, we observed a significant RR of 1.37 (95% CI, 1.07–1.75) but again the heterogeneity was substantial (Supplemental Fig. 23; I^2^, 77.6%). Only two out of the nine prospective studies used multivariable adjusted models, and the meta-analysis among them yielded null results (Table 1; RR, 1.05; 95% CI, 0.83–1.34; I^2^, 0%)^59,116^. When 13 case-control studies were meta-analysed together with the prospective studies, a statistically significant association was found (Supplemental Table 4; RR, 1.58; 95% CI, 1.35–1.85; I^2^, 88%). No analysis on SBP or DBP was performed, because there were not enough studies using continuous BP data.

Using data from 5 prospective studies^37,69,76,84,112^, we also noted a statistically significant association between hypertension and liver cancer (Fig. 2; RR, 1.23; 95% CI, 1.16–1.30; I^2^, 0%), but four out the five studies did not perform comprehensive multi-variable adjustments (Table 1 and Supplemental Table 1). After removing one record linkage study^76^, the result remained identical. After adding two case control studies, the association lost significance (Supplemental Table 4; RR, 1.30; 95% CI, 0.99–1.71).

The meta-analysis of 4 prospective studies between hypertension and esophageal adenocarcinoma yielded a statistically significant positive association (Fig. 2; RR 1.11; 95% CI, 1.01–1.21; I^2^, 5.5%)^14,50,77,111^. We identified only 2 prospective studies on hypertension and esophageal squamous cell carcinoma^14,77^, and the meta-analysis yielded a statistically significant estimate (RR, 1.74; 95% CI, 1.32–2.30; I^2^, 0%), but was based only on 248 cases. Finally, the meta-analysis of all 6 prospective studies on total oesophageal cancer yielded a statistically not significant estimate (RR, 1.18; 95% CI, 1.00–1.38; I^2^, 67.5%). Most of these studies however did not perform comprehensive multivariable adjustments (Table 1 and Supplemental Table 1).

We did not observe statistically significant associations between hypertension or SBP/DBP and cancer of stomach, gallbladder, pancreas, lung, cervix, prostate, bladder and brain (Figs 2, 3 and Table 1).

## Discussion

In this meta-analysis of observational studies, we summarized the associations between hypertension or blood pressure and risk of 18 cancers. We confirmed the positive association between hypertension and risk of kidney cancer, but also found possible positive associations between hypertension and risk of colorectal, breast, endometrial, liver and oesophageal cancer. We did not observe statistically significant associations for cancers of the stomach, gallbladder, pancreas, lung, breast, cervix, prostate, bladder and brain.

Over the past few decades, many prospective observational studies have investigated the association between hypertension and risk of kidney cancer. Most of these studies have showed a positive association, which summarized in our meta-analysis to a 54% higher risk that was stronger in women compared to men (63% vs. 29%). Furthermore, a dose-response approach revealed a 5% and 7% higher risk for kidney cancer per every 10 mmHg higher SBP and DBP, respectively. Similar associations have been reported in previous meta-analyses^6,20,180^. However, this association is complex and it is still unclear whether it is causal, as both hypertension and cancer are affected by similar risk factors such as smoking, obesity, alcohol consumption and physical inactivity^181^. Several studies have not adjusted for many of these potential confounders, but four prospective studies that did follow a comprehensive adjustment approach still observed positive associations as outlined in the current and other meta-analyses^180^. Future large prospective studies or consortia thereof and Mendelian randomization studies are needed to clarify if the observed association is likely causal^182^. A recent Mendelian randomization study of renal cell cancer showed a positive association with diastolic but not with systolic blood pressure^183^. The biological mechanisms underlying the association between hypertension and kidney cancer remain unclear, but are hypothesized to involve chronic renal hypoxia, lipid peroxidation and deregulation of renin-angiotensin system and specifically the overexpression of angiotensin receptors and the down-regulation of the angiotensin-converting enzyme^184–186^.

We also estimated a positive summary association between hypertension and risk of colorectal cancer with a higher risk of 11% for hypertensive individuals. This meta-analysis included data from 13 prospective studies, only four of which performed comprehensive multivariable adjustments, and the potential higher risk was 30% for hypertensive individuals in these studies. Esposito *et al*. published another meta-analysis in 2013, which found a 9% relative increase in colorectal cancer due to hypertension using both cohort and case-control studies^10^. To our knowledge, no clear mechanism has been proposed to link hypertension to colorectal cancer, but hypertension has been shown to increase cancer risk by blocking apoptosis^187^. Current findings should be approached with caution given the scarcity of well-designed studies in the literature.

In a meta-analysis of 13 prospective studies, hypertension was associated with a 7% higher risk of total breast cancer. Only five of these studies performed comprehensive multivariable adjustments, where the risk was 10% for total breast cancer and 38% for post-menopausal disease comparing hypertensive to normotensive individuals. These findings are in agreement with a recent meta-analysis of 12 prospective studies by Han *et al*. that reported a 7% higher risk for total breast risk in hypertensive individuals regardless of performed adjustments^11^. Suggestive mechanisms to explain this association involve blocking of apoptosis, adipose tissue related hypoxia and chronic inflammation promoting reactive oxygen species formation^188^.

Positive associations between hypertension and risk of esophageal adenocarcinoma and squamous cell carcinoma, liver, and endometrial cancer were also observed in the current meta-analysis, but these meta-analyses included a small number of prospective studies, ranging from 2 for oesophageal squamous cell carcinoma to 9 for endometrial cancer, and the majority of them did not perform comprehensive multivariable adjustments, raising serious concern over the validity of the estimated associations. Previous meta-analyses exist for endometrial cancer, but have in general shaped their conclusions without taking into consideration study design and quality^8,9^.

We did not observe associations between hypertension or blood pressure and risk of stomach, gallbladder, pancreas, lung, cervix, prostate, bladder and brain cancer. A recent meta-analysis examining the association between hypertension and risk of prostate cancer used 21 cohort and case-control studies and found a statistically significant 8% higher risk, but again this report shaped its conclusions without taking into consideration individual study design and quality^12^.

Strengths of the present meta-analysis include the comprehensive search strategy with the inclusion of “metabolic syndrome” in the search algorithm, the wide scope of investigating associations with many individual cancers and the detailed sensitivity and subgroup analyses. There are also limitations in this work. First, the association between hypertension and malignancy may be due to shared risk factors such as age, smoking, alcohol consumption, diet and adiposity. Unfortunately, the majority of the literature in this field contains studies that did not perform comprehensive multivariable adjustments, which raises concerns over the robustness of individual study findings even for the well-described association between hypertension and kidney cancer. Second, most studies did not perform subgroup analyses by potential effect modifiers (e.g. anti-hypertensive medication use), which would have allowed a more accurate assessment of associations in different patient subgroups. Third, detection bias may also account for some of the reported associations, as individuals treated for hypertension are under closer medical surveillance that may lead to easier detection of cancer compared to untreated persons. Future well-designed prospective studies and Mendelian randomization studies should assist in estimating valid measures of association.

In conclusion, the currently available published observational evidence across 18 cancer sites showed that hypertensive individuals were at higher risk of kidney, colorectal and breast cancer. However, careful interpretation is required as most meta-analyses included relatively small number of studies, several relative risks had weak or moderate magnitude and maybe affected by residual confounding.

## Supplementary information

Supplementary Information

## Data Availability

All data generated or analysed during this study are included in this published article (and its Supplementary Information files).

## References

[1] Go AS (2014). Heart disease and stroke statistics–2014 update: a report from the American Heart Association. Circulation.

[2] Forouzanfar MH (2017). Global Burden of Hypertension and Systolic Blood Pressure of at Least 110 to 115 mm Hg, 1990-2015. Jama.

[3] Campbell NR, Lackland DT, Niebylski ML (2014). High blood pressure: why prevention and control are urgent and important: a 2014 fact sheet from the World Hypertension League and the International Society of Hypertension. Journal of clinical hypertension (Greenwich, Conn.).

[4] Ferlay, J., Colombet, M., Soerjomataram, I., Mathers, C. & Parkin, D. M. Estimating the global cancer incidence and mortality in 2018: GLOBOCAN sources and methods. **144**, 1941–1953, 10.1002/ijc.31937 (2019). 10.1002/ijc.31937 30350310

[5] Chow WH, Dong LM, Devesa SS (2010). Epidemiology and risk factors for kidney cancer. Nature reviews. Urology.

[6] Corrao G, Scotti L, Bagnardi V, Sega R (2007). Hypertension, antihypertensive therapy and renal-cell cancer: a meta-analysis. Current drug safety.

[7] Hamet P (1996). Cancer and hypertension. An unresolved issue. Hypertension.

[8] Aune D, Sen A, Vatten LJ (2017). Hypertension and the risk of endometrial cancer: a systematic review and meta-analysis of case-control and cohort studies. Scientific Reports.

[9] Esposito K (2014). Metabolic syndrome and endometrial cancer: a meta-analysis. Endocrine.

[10] Esposito K (2013). Colorectal cancer association with metabolic syndrome and its components: a systematic review with meta-analysis. Endocrine.

[11] Han, H. *et al*. Hypertension and breast cancer risk: a systematic review and meta-analysis. *Scientific Reports* **7**, 10.1038/srep44877 (2017). 10.1038/srep44877 PMC5357949 28317900

[12] Liang, Z. *et al*. Hypertension and risk of prostate cancer: a systematic review and meta-analysis. *Scientific Reports* **6**, 10.1038/srep31358 (2016). 10.1038/srep31358 PMC4980763 27511796

[13] Trabert B (2015). Metabolic syndrome and risk of endometrial cancer in the united states: a study in the SEER-medicare linked database. Cancer epidemiology, biomarkers & prevention: a publication of the American Association for Cancer Research, cosponsored by the American Society of Preventive Oncology.

[14] Lin Y, Ness-Jensen E, Hveem K, Lagergren J, Lu Y (2015). Metabolic syndrome and esophageal and gastric cancer. Cancer causes & control: CCC.

[15] De Nunzio C, Aronson W, Freedland SJ, Giovannucci E, Parsons JK (2012). The correlation between metabolic syndrome and prostatic diseases. European urology.

[16] Dhote R, Thiounn N, Debre B, Vidal-Trecan G (2004). Risk factors for adult renal cell carcinoma. The Urologic clinics of North America.

[17] Esposito K (2013). Effect of metabolic syndrome and its components on prostate cancer risk: meta-analysis. Journal of endocrinological investigation.

[18] Esposito K (2013). Metabolic syndrome and postmenopausal breast cancer: systematic review and meta-analysis. Menopause (New York, N.Y.).

[19] Esposito K, Chiodini P, Colao A, Lenzi A, Giugliano D (2012). Metabolic syndrome and risk of cancer: a systematic review and meta-analysis. Diabetes care.

[20] Grossman E, Messerli FH, Boyko V, Goldbourt U (2002). Is there an association between hypertension and cancer mortality?. The American journal of medicine.

[21] Jinjuvadia R, Lohia P, Jinjuvadia C, Montoya S, Liangpunsakul S (2013). The association between metabolic syndrome and colorectal neoplasm: systemic review and meta-analysis. Journal of clinical gastroenterology.

[22] Jinjuvadia R, Patel S, Liangpunsakul S (2014). The association between metabolic syndrome and hepatocellular carcinoma: systemic review and meta-analysis. Journal of clinical gastroenterology.

[23] Navai N, Wood CG (2012). Environmental and modifiable risk factors in renal cell carcinoma. Urologic oncology.

[24] Xiang YZ (2013). The association between metabolic syndrome and the risk of prostate cancer, high-grade prostate cancer, advanced prostate cancer, prostate cancer-specific mortality and biochemical recurrence. Journal of experimental & clinical cancer research: CR.

[25] Wells, G. A. *et al*. The Newcastle-Ottawa Scale (NOS) for assessing the quality of nonrandomised studies in meta-analyses. *Available from:* http://www.ohri.ca/programs/clinical_epidemiology/oxford.asp.

[26] Greenland S, Longnecker MP (1992). Methods for trend estimation from summarized dose-response data, with applications to meta-analysis. Am J Epidemiol.

[27] Orsini N, Bellocco R, Greenland S (2006). Generalized least squares for trend estimation of summarized dose-response data. Stata Journal.

[28] Borenstein M, Hedges LV, Higgins JP, Rothstein HR (2010). A basic introduction to fixed-effect and random-effects models for meta-analysis. Research synthesis methods.

[29] Higgins, J. P. T., Thompson, S. G., Deeks, J. J. & Altman, D. G. Measuring inconsistency in meta-analyses. *BMJ (Clinical research ed.)* **327**, 557–560 (2003). 10.1136/bmj.327.7414.557 PMC192859 12958120

[30] Riley RD, Higgins JP, Deeks JJ (2011). Interpretation of random effects meta-analyses. BMJ (Clinical research ed.).

[31] Sterne J. A. C., Sutton A. J., Ioannidis J. P. A., Terrin N., Jones D. R., Lau J., Carpenter J., Rucker G., Harbord R. M., Schmid C. H., Tetzlaff J., Deeks J. J., Peters J., Macaskill P., Schwarzer G., Duval S., Altman D. G., Moher D., Higgins J. P. T. (2011). Recommendations for examining and interpreting funnel plot asymmetry in meta-analyses of randomised controlled trials. BMJ.

[32] Stroup DF (2000). Meta-analysis of observational studies in epidemiology: a proposal for reporting. Meta-analysis Of Observational Studies in Epidemiology (MOOSE) group. Jama.

[33] Agnoli C (2010). Metabolic syndrome and postmenopausal breast cancer in the ORDET cohort: a nested case-control study. Nutrition, metabolism, and cardiovascular diseases: NMCD.

[34] Agnoli C (2015). Metabolic syndrome and breast cancer risk: a case-cohort study nested in a multicentre italian cohort. PLoS ONE.

[35] Ahmed RL, Schmitz KH, Anderson KE, Rosamond WD, Folsom AR (2006). The metabolic syndrome and risk of incident colorectal cancer. Cancer.

[36] Aleksandrova K (2011). Metabolic syndrome and risks of colon and rectal cancer: the European prospective investigation into cancer and nutrition study. Cancer prevention research (Philadelphia, Pa.).

[37] Assimes TL, Suissa S (2009). Age at incident treatment of hypertension and risk of cancer: a population study. Cancer causes & control: CCC.

[38] Batty GD, Kivimaki M, Clarke R, Davey Smith G, Shipley MJ (2011). Modifiable risk factors for prostate cancer mortality in London: forty years of follow-up in the Whitehall study. Cancer causes & control: CCC.

[39] Batty GD (2009). Risk factors for pancreatic cancer mortality: extended follow-up of the original Whitehall Study. Cancer epidemiology, biomarkers & prevention: a publication of the American Association for Cancer Research, cosponsored by the American Society of Preventive Oncology.

[40] Batty GD, Shipley MJ, Marmot MG, Davey Smith G (2003). Blood pressure and site-specific cancer mortality: evidence from the original Whitehall study. British journal of cancer.

[41] Berrington de Gonzalez A, Yun JE, Lee SY, Klein AP, Jee SH (2008). Pancreatic cancer and factors associated with the insulin resistance syndrome in the Korean cancer prevention study. Cancer epidemiology, biomarkers & prevention: a publication of the American Association for Cancer Research, cosponsored by the American Society of Preventive Oncology.

[42] Bjorge T (2010). Metabolic syndrome and breast cancer in the me-can (metabolic syndrome and cancer) project. Cancer epidemiology, biomarkers & prevention: a publication of the American Association for Cancer Research, cosponsored by the American Society of Preventive Oncology.

[43] Borena W (2014). A prospective study on metabolic risk factors and gallbladder cancer in the metabolic syndrome and cancer (Me-Can) collaborative study. Plos One.

[44] Bosco JL, Palmer JR, Boggs DA, Hatch EE, Rosenberg L (2012). Cardiometabolic factors and breast cancer risk in U.S. black women. Breast cancer research and treatment.

[45] Bowers K (2006). A prospective study of anthropometric and clinical measurements associated with insulin resistance syndrome and colorectal cancer in male smokers. Am J Epidemiol.

[46] Choi MY, Jee SH, Sull JW, Nam CM (2005). The effect of hypertension on the risk for kidney cancer in Korean men. Kidney international.

[47] Chow WH, Gridley G, Fraumeni JF, Jarvholm B (2000). Obesity, hypertension, and the risk of kidney cancer in men. The New England journal of medicine.

[48] Colangelo LA, Gapstur SM, Gann PH, Dyer AR, Liu K (2002). Colorectal cancer mortality and factors related to the insulin resistance syndrome. Cancer epidemiology, biomarkers & prevention: a publication of the American Association for Cancer Research, cosponsored by the American Society of Preventive Oncology.

[49] Cust AE (2007). Metabolic syndrome, plasma lipid, lipoprotein and glucose levels, and endometrial cancer risk in the European Prospective Investigation into Cancer and Nutrition (EPIC). Endocrine-related cancer.

[50] Drahos J, Li L, Jick SS, Cook MB (2016). Metabolic syndrome in relation to Barrett’s esophagus and esophageal adenocarcinoma: Results from a large population-based case-control study in the Clinical Practice Research Datalink. Cancer epidemiology.

[51] Dyer AR, Stamler J, Berkson DM, Lindberg HA, Stevens E (1975). High blood-pressure: a risk factor for cancer mortality?. Lancet (London, England).

[52] Edlinger M (2012). Blood pressure and other metabolic syndrome factors and risk of brain tumour in the large population-based Me-Can cohort study. Journal of hypertension.

[53] Eijgenraam P (2013). Diabetes type II, other medical conditions and pancreatic cancer risk: a prospective study in The Netherlands. British journal of cancer.

[54] Fitzpatrick AL, Daling JR, Furberg CD, Kronmal RA, Weissfeld JL (2001). Hypertension, heart rate, use of antihypertensives, and incident prostate cancer. Annals of epidemiology.

[55] Flaherty KT (2005). A prospective study of body mass index, hypertension, and smoking and the risk of renal cell carcinoma (United States). Cancer causes & control: CCC.

[56] Fraser GE, Phillips RL, Beeson WL (1990). Hypertension, antihypertensive medication and risk of renal carcinoma in California Seventh-Day Adventists. International journal of epidemiology.

[57] Friedman GD (1997). Blood pressure and heart rate: no evidence for a positive association with prostate cancer. Annals of epidemiology.

[58] Fryzek JP (2005). A cohort study of antihypertensive treatments and risk of renal cell cancer. British journal of cancer.

[59] Furberg AS, Thune I (2003). Metabolic abnormalities (hypertension, hyperglycemia and overweight), lifestyle (high energy intake and physical inactivity) and endometrial cancer risk in a Norwegian cohort. International journal of cancer.

[60] Goldbourt U (1986). Elevated systolic blood pressure as a predictor of long-term cancer mortality: analysis by site and histologic subtype in 10,000 middle-aged and elderly men. Journal of the National Cancer Institute.

[61] Grove JS, Nomura A, Severson RK, Stemmermann GN (1991). The association of blood pressure with cancer incidence in a prospective study. Am J Epidemiol.

[62] Haggstrom C (2013). Metabolic factors associated with risk of renal cell carcinoma. Plos One.

[63] Haggstrom C (2011). Metabolic syndrome and risk of bladder cancer: prospective cohort study in the metabolic syndrome and cancer project (Me-Can). International journal of cancer.

[64] Haggstrom C (2012). Prospective study on metabolic factors and risk of prostate cancer. Cancer.

[65] Harding J (2015). The metabolic syndrome and cancer: Is the metabolic syndrome useful for predicting cancer risk above and beyond its individual components? *Diabetes &*. metabolism.

[66] Heath CW, Lally CA, Calle EE, McLaughlin JK, Thun MJ (1997). Hypertension, diuretics, and antihypertensive medications as possible risk factors for renal cell cancer. Am J Epidemiol.

[67] Hofmann JN (2015). Chronic kidney disease and risk of renal cell carcinoma: differences by race. Epidemiology (Cambridge, Mass.).

[68] Houben MP (2006). The association between antihypertensive drugs and glioma. British journal of cancer.

[69] Inoue M (2009). Impact of metabolic factors on subsequent cancer risk: results from a large-scale population-based cohort study in Japan. European journal of cancer prevention: the official journal of the European Cancer Prevention Organisation (ECP).

[70] Kabat GC (2009). A longitudinal study of the metabolic syndrome and risk of postmenopausal breast cancer. Cancer epidemiology, biomarkers & prevention: a publication of the American Association for Cancer Research, cosponsored by the American Society of Preventive Oncology.

[71] Kabat GC (2012). A longitudinal study of the metabolic syndrome and risk of colorectal cancer in postmenopausal women. European journal of cancer prevention: the official journal of the European Cancer Prevention Organisation (ECP).

[72] Ko Seulki, Yoon Seok-Jun, Kim Dongwoo, Kim A-Rim, Kim Eun-Jung, Seo Hye-Young (2016). Metabolic Risk Profile and Cancer in Korean Men and Women. Journal of Preventive Medicine and Public Health.

[73] Largent JA (2010). Hypertension, antihypertensive medication use, and breast cancer risk in the California Teachers Study cohort. Cancer causes & control: CCC.

[74] Lawrence YR (2013). Association between metabolic syndrome, diabetes mellitus and prostate cancer risk. Prostate cancer and prostatic diseases.

[75] Lee SY, Kim MT, Jee SH, Im JS (2002). Does hypertension increase mortality risk from lung cancer? A prospective cohort study on smoking, hypertension and lung cancer risk among Korean men. Journal of hypertension.

[76] Lindgren AM, Nissinen AM, Tuomilehto JO, Pukkala E (2005). Cancer pattern among hypertensive patients in North Karelia, Finland. Journal of human hypertension.

[77] Lindkvist B (2014). Metabolic risk factors for esophageal squamous cell carcinoma and adenocarcinoma: a prospective study of 580,000 subjects within the Me-Can project. BMC cancer.

[78] Lund Haheim L, Wisloff TF, Holme I, Nafstad P (2006). Metabolic syndrome predicts prostate cancer in a cohort of middle-aged Norwegian men followed for 27 years. Am J Epidemiol.

[79] Maatela J (1994). The risk of endometrial cancer in diabetic and hypertensive patients: a nationwide record-linkage study in Finland. Annales chirurgiae et gynaecologiae. Supplementum.

[80] Macleod LC (2013). Risk factors for renal cell carcinoma in the VITAL study. The Journal of urology.

[81] Martin RM, Vatten L, Gunnell D, Romundstad P (2010). Blood pressure and risk of prostate cancer: Cohort Norway (CONOR). Cancer causes & control: CCC.

[82] Morrison DS (2011). Risk factors for colonic and rectal cancer mortality: evidence from 40 years’ follow-up in the Whitehall I study. Journal of epidemiology and community health.

[83] Nicodemus KK, Sweeney C, Folsom AR (2004). Evaluation of dietary, medical and lifestyle risk factors for incident kidney cancer in postmenopausal women. International journal of cancer.

[84] Osaki Y, Taniguchi S, Tahara A, Okamoto M, Kishimoto T (2012). Metabolic syndrome and incidence of liver and breast cancers in Japan. Cancer epidemiology.

[85] Peeters PH (2000). Hypertension and breast cancer risk in a 19-year follow-up study (the DOM cohort). Diagnostic investigation into mammarian cancer. Journal of hypertension.

[86] Peeters PH, van Noord PA, Hoes AW, Grobbee DE (1998). Hypertension, antihypertensive drugs, and mortality from cancer among women. Journal of hypertension.

[87] Reeves KW, McLaughlin V, Fredman L, Ensrud K, Cauley JA (2012). Components of metabolic syndrome and risk of breast cancer by prognostic features in the study of osteoporotic fractures cohort. Cancer causes & control: CCC.

[88] Romero FR, Romero AW, Almeida RM, Oliveira FC, Tambara Filho R (2012). The significance of biological, environmental, and social risk factors for prostate cancer in a cohort study in Brazil. International braz j urol: official journal of the Brazilian Society of Urology.

[89] Ronquist G (2004). Association between captopril, other antihypertensive drugs and risk of prostate cancer. The Prostate.

[90] Rosengren A, Himmelmann A, Wilhelmsen L, Branehog I, Wedel H (1998). Hypertension and long-term cancer incidence and mortality among Swedish men. Journal of hypertension.

[91] Sanfilippo KM (2014). Hypertension and obesity and the risk of kidney cancer in 2 large cohorts of US men and women. Hypertension.

[92] Schouten LJ (2005). Hypertension, antihypertensives and mutations in the Von Hippel-Lindau gene in renal cell carcinoma: results from the Netherlands Cohort Study. Journal of hypertension.

[93] Setiawan VW, Stram DO, Nomura AM, Kolonel LN, Henderson BE (2007). Risk factors for renal cell cancer: the multiethnic cohort. Am J Epidemiol.

[94] Shen T (2015). Association of Hypertension and Obesity with Renal Cell Carcinoma Risk: A Report from the Shanghai Men’s and Women’s Health Studies. Cancer causes & control: CCC.

[95] Stocks T, Hergens MP, Englund A, Ye W, Stattin P (2010). Blood pressure, body size and prostate cancer risk in the Swedish Construction Workers cohort. International journal of cancer.

[96] Stocks T (2008). Components of the metabolic syndrome and colorectal cancer risk; a prospective study. International journal of obesity (2005).

[97] Stocks T (2012). Blood pressure and risk of cancer incidence and mortality in the Metabolic Syndrome and Cancer Project. Hypertension.

[98] Suadicani P, Hein HO, Gyntelberg F (1993). Is the use of antihypertensives and sedatives a major risk factor for colorectal cancer?. Scandinavian journal of gastroenterology.

[99] Sun LM (2015). Hypertension and subsequent genitourinary and gynecologic cancers risk: a population-based cohort study. Medicine.

[100] Tande AJ, Platz EA, Folsom AR (2006). The metabolic syndrome is associated with reduced risk of prostate cancer. Am J Epidemiol.

[101] Thompson MM (1989). Heart disease risk factors, diabetes, and prostatic cancer in an adult community. Am J Epidemiol.

[102] Tornberg SA, Holm LE, Carstensen JM (1988). Breast cancer risk in relation to serum cholesterol, serum beta-lipoprotein, height, weight, and blood pressure. Acta oncologica (Stockholm, Sweden).

[103] Trevisan M (2001). Markers of insulin resistance and colorectal cancer mortality. Cancer epidemiology, biomarkers & prevention: a publication of the American Association for Cancer Research, cosponsored by the American Society of Preventive Oncology.

[104] Tulinius H, Sigfusson N, Sigvaldason H, Bjarnadottir K, Tryggvadottir L (1997). Risk factors for malignant diseases: a cohort study on a population of 22,946 Icelanders. Cancer epidemiology, biomarkers & prevention: a publication of the American Association for Cancer Research, cosponsored by the American Society of Preventive Oncology.

[105] Vatten LJ, Trichopoulos D, Holmen J, Nilsen TI (2007). Blood pressure and renal cancer risk: the HUNT Study in Norway. British journal of cancer.

[106] Wallner LP (2011). The effects of metabolic conditions on prostate cancer incidence over 15 years of follow-up: results from the Olmsted County Study. BJU international.

[107] Washio M (2014). Cigarette smoking and other risk factors for kidney cancer death in a Japanese population: Japan Collaborative Cohort Study for evaluation of cancer risk (JACC study). Asian Pacific journal of cancer prevention: APJCP.

[108] Watanabe Y (2005). Medical history of circulatory diseases and colorectal cancer death in the JACC Study. Journal of epidemiology.

[109] Weikert S (2008). Blood pressure and risk of renal cell carcinoma in the European prospective investigation into cancer and nutrition. Am J Epidemiol.

[110] Cook NR, Rosner BA, Hankinson SE, Colditz GA (2009). Mammographic screening and risk factors for breast cancer. Am J Epidemiol.

[111] Drahos J, Ricker W, Pfeiffer RM, Cook MB (2017). Metabolic syndrome and risk of esophageal adenocarcinoma in elderly patients in the United States: An analysis of SEER-Medicare data. Cancer.

[112] Kasmari AJ (2017). Independent of Cirrhosis, Hepatocellular Carcinoma Risk Is Increased with Diabetes and Metabolic Syndrome. The American journal of medicine.

[113] Shin CM (2017). Association Among Obesity, Metabolic Health, and the Risk for Colorectal Cancer in the General Population in Korea Using the National Health Insurance Service-National Sample Cohort. Diseases of the colon and rectum.

[114] Sturmer T, Buring JE, Lee IM, Gaziano JM, Glynn RJ (2006). Metabolic abnormalities and risk for colorectal cancer in the physicians’ health study. Cancer epidemiology, biomarkers & prevention: a publication of the American Association for Cancer Research, cosponsored by the American Society of Preventive Oncology.

[115] Folsom AR, Demissie Z, Harnack L (2003). Glycemic index, glycemic load, and incidence of endometrial cancer: the Iowa women’s health study. Nutrition and cancer.

[116] Sponholtz TR (2016). Body Size, Metabolic Factors, and Risk of Endometrial Cancer in Black Women. Am J Epidemiol.

[117] Beebe-Dimmer JL, Dunn RL, Sarma AV, Montie JE, Cooney KA (2007). Features of the metabolic syndrome and prostate cancer in African-American men. Cancer.

[118] Beebe-Dimmer JL (2009). Racial differences in risk of prostate cancer associated with metabolic syndrome. Urology.

[119] Bhindi B (2015). Dissecting the association between metabolic syndrome and prostate cancer risk: analysis of a large clinical cohort. European urology.

[120] Blanc-Lapierre A (2015). Metabolic syndrome and prostate cancer risk in a population-based case-control study in Montreal, Canada. BMC public health.

[121] Brennan P (2008). Tobacco smoking, body mass index, hypertension, and kidney cancer risk in central and eastern Europe. British journal of cancer.

[122] Brinton LA (1992). Reproductive, menstrual, and medical risk factors for endometrial cancer: results from a case-control study. American journal of obstetrics and gynecology.

[123] Chow WH (1995). Risk of renal cell cancer in relation to diuretics, antihypertensive drugs, and hypertension. Cancer epidemiology, biomarkers & prevention: a publication of the American Association for Cancer Research, cosponsored by the American Society of Preventive Oncology.

[124] Colt JS (2011). Hypertension and risk of renal cell carcinoma among white and black Americans. Epidemiology (Cambridge, Mass.).

[125] Finkle WD, McLaughlin JK, Rasgon SA, Yeoh HH, Low JE (1993). Increased risk of renal cell cancer among women using diuretics in the United States. Cancer causes & control: CCC.

[126] Fortuny J (2009). Risk of endometrial cancer in relation to medical conditions and medication use. Cancer epidemiology, biomarkers & prevention: a publication of the American Association for Cancer Research, cosponsored by the American Society of Preventive Oncology.

[127] Friedenreich CM (2011). Case-control study of the metabolic syndrome and metabolic risk factors for endometrial cancer. Cancer epidemiology, biomarkers & prevention: a publication of the American Association for Cancer Research, cosponsored by the American Society of Preventive Oncology.

[128] Ganesh B, Saoba SL, Sarade MN, Pinjari SV (2011). Risk factors for prostate cancer: An hospital-based case-control study from Mumbai, India. Indian journal of urology: IJU: journal of the Urological Society of India.

[129] Hardell L, Fredrikson M, Axelson O (1996). Case-control study on colon cancer regarding previous diseases and drug intake. International journal of oncology.

[130] Inoue M (1994). A case-control study on risk factors for uterine endometrial cancer in Japan. Japanese journal of cancer research: Gann.

[131] Jiang X (2010). Hypertension, diuretics and antihypertensives in relation to bladder cancer. Carcinogenesis.

[132] Jung SJ (2013). Association of selected medical conditions with breast cancer risk in Korea. Journal of preventive medicine and public health=Yebang Uihakhoe chi.

[133] Kreiger N, Marrett LD, Dodds L, Hilditch S, Darlington GA (1993). Risk factors for renal cell carcinoma: results of a population-based case-control study. Cancer causes & control: CCC.

[134] Kune GA, Kune S, Watson LF (2007). Colorectal cancer risk, chronic illnesses, operations and medications: case control results from the Melbourne Colorectal Cancer Study. 1988. International journal of epidemiology.

[135] Lai SW (2013). Kidney cancer and diabetes mellitus: a population-based case-control study in Taiwan. Annals of the Academy of Medicine, Singapore.

[136] Largent JA (2006). Hypertension, diuretics and breast cancer risk. Journal of human hypertension.

[137] Li CI (2013). Use of antihypertensive medications and breast cancer risk among women aged 55 to 74 years. JAMA internal medicine.

[138] Li CI (2003). Relation between use of antihypertensive medications and risk of breast carcinoma among women ages 65-79 years. Cancer.

[139] Liaw KL (1997). Possible relation between hypertension and cancers of the renal pelvis and ureter. International journal of cancer.

[140] McCredie M, Stewart JH (1992). Risk factors for kidney cancer in New South Wales, Australia. II. Urologic disease, hypertension, obesity, and hormonal factors. Cancer causes & control: CCC.

[141] McLaughlin JK (1995). International renal-cell cancer study. VIII. Role of diuretics, other anti-hypertensive medications and hypertension. International journal of cancer.

[142] Mellemgaard A (1994). Risk factors for renal cell carcinoma in Denmark: role of medication and medical history. International journal of epidemiology.

[143] Noh HM, Song YM, Park JH, Kim BK, Choi YH (2013). Metabolic factors and breast cancer risk in Korean women. Cancer causes & control: CCC.

[144] Oishi K (1989). Case-control study of prostatic cancer in Kyoto, Japan: demographic and some lifestyle risk factors. The Prostate.

[145] Pelucchi C (2010). Metabolic syndrome is associated with colorectal cancer in men. European journal of cancer (Oxford, England: 1990).

[146] Pelucchi C (2011). The metabolic syndrome and risk of prostate cancer in Italy. Annals of epidemiology.

[147] Pereira A, Garmendia ML, Alvarado ME, Albala C (2012). Hypertension and the risk of breast cancer in Chilean women: a case-control study. Asian Pacific journal of cancer prevention: APJCP.

[148] Perron L, Bairati I, Harel F, Meyer F (2004). Antihypertensive drug use and the risk of prostate cancer (Canada). Cancer causes & control: CCC.

[149] Reis N, Beji NK (2009). Risk factors for endometrial cancer in Turkish women: results from a hospital-based case-control study. European journal of oncology nursing: the official journal of European Oncology Nursing Society.

[150] Ronco AL, De Stefani E, Deneo-Pellegrini H (2012). Risk factors for premenopausal breast cancer: a case-control study in Uruguay. Asian Pacific journal of cancer prevention: APJCP.

[151] Ronco AL, De Stefani E, Deneo-Pellegrini H, Quarneti A (2012). Diabetes, overweight and risk of postmenopausal breast cancer: a case-control study in Uruguay. Asian Pacific journal of cancer prevention: APJCP.

[152] Rosato V (2016). Medical conditions, family history of cancer, and the risk of biliary tract cancers. Tumori.

[153] Rosato V (2011). Metabolic syndrome and the risk of breast cancer in postmenopausal women. Annals of oncology: official journal of the European Society for Medical Oncology.

[154] Rosato V (2011). Metabolic syndrome and pancreatic cancer risk: a case-control study in Italy and meta-analysis. Metabolism: clinical and experimental.

[155] Rosato V (2011). Metabolic syndrome and endometrial cancer risk. Annals of oncology: official journal of the European Society for Medical Oncology.

[156] Shapiro JA (1999). Hypertension, antihypertensive medication use, and risk of renal cell carcinoma. Am J Epidemiol.

[157] Shebl FM (2011). Metabolic syndrome and insulin resistance in relation to biliary tract cancer and stone risks: a population-based study in Shanghai, China. British journal of cancer.

[158] Soler M (1999). Hypertension and hormone-related neoplasms in women. Hypertension.

[159] Thompson WD, Jacobson HI, Negrini B, Janerich DT (1989). Hypertension, pregnancy, and risk of breast cancer. Journal of the National Cancer Institute.

[160] Turati F (2013). Metabolic syndrome and hepatocellular carcinoma risk. British journal of cancer.

[161] Wang G (2012). Risk factor for clear cell renal cell carcinoma in Chinese population: a case-control study. Cancer epidemiology.

[162] Weiderpass E (2000). Body size in different periods of life, diabetes mellitus, hypertension, and risk of postmenopausal endometrial cancer (Sweden). Cancer causes & control: CCC.

[163] Weinmann S (1994). Use of diuretics and other antihypertensive medications in relation to the risk of renal cell cancer. Am J Epidemiol.

[164] Weinmann S (2010). Medical history, body size, and cigarette smoking in relation to fatal prostate cancer. Cancer causes & control: CCC.

[165] Welzel TM (2011). Metabolic syndrome increases the risk of primary liver cancer in the United States: a study in the SEER-Medicare database. Hepatology (Baltimore, Md.).

[166] Xu S (2015). The association between metabolic syndrome and the risk of urothelial carcinoma of the bladder: a case-control study in China. World journal of surgical oncology.

[167] Yuan JM, Castelao JE, Gago-Dominguez M, Ross RK, Yu MC (1998). Hypertension, obesity and their medications in relation to renal cell carcinoma. British journal of cancer.

[168] Zhang Y (2010). The association between metabolic abnormality and endometrial cancer: a large case-control study in China. Gynecologic oncology.

[169] Zucchetto A (2007). History of treated hypertension and diabetes mellitus and risk of renal cell cancer. Annals of oncology: official journal of the European Society for Medical Oncology.

[170] Austin H, Austin JM, Partridge EE, Hatch KD, Shingleton HM (1991). Endometrial cancer, obesity, and body fat distribution. Cancer research.

[171] Franceschi S, la Vecchia C, Negri E, Parazzini F, Boyle P (1990). Breast cancer risk and history of selected medical conditions linked with female hormones. European journal of cancer (Oxford, England: 1990).

[172] Montella M (2015). Metabolic syndrome and the risk of urothelial carcinoma of the bladder: a case-control study. BMC cancer.

[173] Moseson M, Koenig KL, Shore RE, Pasternack BS (1993). The influence of medical conditions associated with hormones on the risk of breast cancer. International journal of epidemiology.

[174] Weiss HA (1999). Breast cancer risk in young women and history of selected medical conditions. International journal of epidemiology.

[175] Wu AH, Vigen C, Butler LM, Tseng CC (2017). Metabolic conditions and breast cancer risk among Los Angeles County Filipina Americans compared with Chinese and Japanese Americans. International journal of cancer.

[176] Goodman MT (1997). Diet, body size, physical activity, and the risk of endometrial cancer. Cancer research.

[177] Shao Y (2016). Insulin is an important risk factor of endometrial cancer among premenopausal women: a case-control study in China. Tumour biology: the journal of the International Society for Oncodevelopmental Biology and Medicine.

[178] Soliman PT (2006). Association between adiponectin, insulin resistance, and endometrial cancer. Cancer.

[179] Washio M (2005). Risk factors for kidney cancer in a Japanese population: findings from the JACC Study. Journal of epidemiology.

[180] Hidayat K, Du X, Zou SY, Shi BM (2017). Blood pressure and kidney cancer risk: meta-analysis of prospective studies. Journal of hypertension.

[181] Koene RJ, Prizment AE, Blaes A, Konety SH (2016). Shared Risk Factors in Cardiovascular Disease and Cancer. Circulation.

[182] Dimou NL, Tsilidis KK (2018). A Primer in Mendelian Randomization Methodology with a Focus on Utilizing Published Summary Association Data. Methods in molecular biology (Clifton, N.J.).

[183] Johansson Mattias, Carreras-Torres Robert, Scelo Ghislaine, Purdue Mark P., Mariosa Daniela, Muller David C., Timpson Nicolas J., Haycock Philip C., Brown Kevin M., Wang Zhaoming, Ye Yuanqing, Hofmann Jonathan N., Foll Matthieu, Gaborieau Valerie, Machiela Mitchell J., Colli Leandro M., Li Peng, Garnier Jean-Guillaume, Blanche Helene, Boland Anne, Burdette Laurie, Prokhortchouk Egor, Skryabin Konstantin G., Yeager Meredith, Radojevic-Skodric Sanja, Ognjanovic Simona, Foretova Lenka, Holcatova Ivana, Janout Vladimir, Mates Dana, Mukeriya Anush, Rascu Stefan, Zaridze David, Bencko Vladimir, Cybulski Cezary, Fabianova Eleonora, Jinga Viorel, Lissowska Jolanta, Lubinski Jan, Navratilova Marie, Rudnai Peter, Benhamou Simone, Cancel-Tassin Geraldine, Cussenot Olivier, Weiderpass Elisabete, Ljungberg Börje, Tumkur Sitaram Raviprakash, Häggström Christel, Bruinsma Fiona, Jordan Susan J., Severi Gianluca, Winship Ingrid, Hveem Kristian, Vatten Lars J., Fletcher Tony, Larsson Susanna C., Wolk Alicja, Banks Rosamonde E., Selby Peter J., Easton Douglas F., Andreotti Gabriella, Beane Freeman Laura E., Koutros Stella, Männistö Satu, Weinstein Stephanie, Clark Peter E., Edwards Todd L., Lipworth Loren, Gapstur Susan M., Stevens Victoria L., Carol Hallie, Freedman Matthew L., Pomerantz Mark M., Cho Eunyoung, Wilson Kathryn M., Gaziano J. Michael, Sesso Howard D., Freedman Neal D., Parker Alexander S., Eckel-Passow Jeanette E., Huang Wen-Yi, Kahnoski Richard J., Lane Brian R., Noyes Sabrina L., Petillo David, Teh Bin Tean, Peters Ulrike, White Emily, Anderson Garnet L., Johnson Lisa, Luo Juhua, Buring Julie, Lee I-Min, Chow Wong-Ho, Moore Lee E., Eisen Timothy, Henrion Marc, Larkin James, Barman Poulami, Leibovich Bradley C., Choueiri Toni K., Lathrop G. Mark, Deleuze Jean-Francois, Gunter Marc, McKay James D., Wu Xifeng, Houlston Richard S., Chanock Stephen J., Relton Caroline, Richards J. Brent, Martin Richard M., Davey Smith George, Brennan Paul (2019). The influence of obesity-related factors in the etiology of renal cell carcinoma—A mendelian randomization study. PLOS Medicine.

[184] Gago-Dominguez M, Castelao JE, Yuan JM, Ross RK, Yu MC (2002). Lipid peroxidation: a novel and unifying concept of the etiology of renal cell carcinoma (United States). Cancer causes & control: CCC.

[185] Sharifi N, Farrar WL (2006). Perturbations in hypoxia detection: a shared link between hereditary and sporadic tumor formation?. Medical hypotheses.

[186] Sobczuk P, Szczylik C, Porta C, Czarnecka AM (2017). Renin angiotensin system deregulation as renal cancer risk factor. Oncology Letters.

[187] Hamet P (1997). Cancer and hypertension: a potential for crosstalk?. Journal of hypertension.

[188] Rausch LK, Netzer NC, Hoegel J, Pramsohler S (2017). The Linkage between Breast Cancer, Hypoxia, and Adipose Tissue. Frontiers in Oncology.

