# Supplementary material for: Association between blood pressure and risk of cancer development: a systematic review and meta-analysis of observational studies
Source: Sci Rep. 2019 Jun 12;9:8565. doi: 10.1038/s41598-019-45014-4 (PMC6561976; doi:10.1038/s41598-019-45014-4)
Supplement: Supplementary file 1 — Supplementary Information [file 41598_2019_45014_MOESM1_ESM.pdf]

Association between blood pressure and risk of cancer development: a systematic review and meta-analysis of observational studies

Aristeidis Seretis, Sofia Cividini, Georgios Markozannes, Xanthippi Tseretopoulou, David S. Lopez, Evangelia E. Ntzani, Konstantinos K. Tsilidis

Supplementary Information

Supplement_Table_1.pdf

Supplement_Table_2.pdf

Supplement_Table_3.pdf

Supplement_Table_4.pdf

Supplement_Figure_1.pdf

Supplement_Figure_2.pdf

Supplement_Figure_3.pdf

Supplement_Figure_4.pdf

Supplement_Figure_5.pdf

Supplement_Figure_6.pdf

Supplement_Figure_7.pdf

Supplement_Figure_8.pdf

Supplement_Figure_9.pdf

Supplement_Figure_10.pdf

Supplement_Figure_11.pdf

Supplement_Figure_12.pdf

Supplement_Figure_13.pdf

Supplement_Figure_14.pdf

Supplement_Figure_15.pdf

Supplement_Figure_16.pdf

Supplement_Figure_17.pdf

Supplement_Figure_18.pdf

Supplement_Figure_19.pdf

Supplement_Figure_20.pdf

Supplement_Figure_21.pdf

Supplement_Figure_22.pdf

Supplement_Figure_23.pdf

Supplement_Figure_24.pdf

Supplement_Figure_25.pdf

Supplement_Figure_26.pdf

Supplement_Figure_27.pdf

Supplement_Figure_28.pdf

Supplement_Figure_29.pdf

Supplement_Figure_30.pdf

Supplement_Figure_31.pdf

Supplement_Figure_32.pdf

Supplement_Figure_33.pdf

Supplement_Figure_34.pdf

Supplement_Figure_35.pdf

Supplement_Figure_36.pdf

Supplement_Figure_37.pdf

Supplement_Figure_38.pdf

Supplement_Figure_39.pdf

Supplement_Figure_40.pdf

Supplement_Figure_41.pdf

Supplement_Figure_42.pdf

Supplement_Figure_43.pdf

Supplement_Figure_44.pdf

Supplement_Figure_45.pdf

Supplement_Figure_46.pdf

Supplement_Figure_47.pdf

Supplement_Figure_48.pdf

Supplement_Figure_49.pdf

Supplement_Figure_50.pdf

Supplement_Figure_51.pdf

Supplement_Figure_52.pdf

Supplement_Figure_53.pdf

Supplement_Figure_54.pdf

Supplement_Figure_55.pdf

Supplement_Figure_56.pdf

Supplement_Figure_57.pdf

Supplement_Figure_58.pdf

Supplement_Figure_59.pdf

Supplement_Figure_60.pdf

Supplement_Figure_61.pdf

Supplement_Figure_62.pdf

Supplement_Figure_63.pdf

Supplement_Figure_64.pdf

Supplement_Figure_65.pdf

Supplement_Figure_66.pdf

Supplement_Figure_67.pdf

Supplement_Figure_68.pdf

Supplement_Figure_69.pdf

Supplement_Figure_70.pdf

Supplement_Figure_71.pdf

Supplement_Figure_72.pdf

Supplement_Figure_73.pdf

Supplement_Figure_74.pdf

Supplement_Figure_75.pdf

Supplement_Figure_76.pdf

Supplement_Figure_77.pdf

Supplement_Figure_78.pdf

Supplement_Figure_79.pdf

Supplement Table 1. Detailed information on all eligible studies used.

Author-Year	Cohort	Country	Study Design	Outcome	Sex	Cases	Controls	Exposure Assessment	Definition of Hypertension	Result	Adjustments
**OESOPHAGEAL CANCER (hypertension)**											
Assimes TL 2009	Saskatchewan Health Database	Canada	cohort	incidence	both	41<br>32	22,113<br>20,157	computerized database files of Saskatchewan Health	medication use	≥60y RR 0.75(0.56–1.00)<br><60y RR 1.49(1.13–1.97)	age, sex, calender year
Drahos J 2016	General Practice Research Database (GPRD)	United Kingdom	nested case-control	incidence	both	592	2,901	computerized medical records	medication use	OR 1.25(1.01-1.53) adenocarcinoma	age, years of GPRD data prior to selection, sex
Drahos J 2017	Surveillance, Epidemiology, and End Results (SEER)-Medicare Database	USA	nested case-control	incidence	both	3,167	15,835	computerized database	≥130/85mmHg and/or history of	OR 1.10(1.00-1.20) adenocarcinoma	age, sex, race, registry, smoking, and history of gastroesophageal reflux disease
Lin Y 2015	CONOR/HUNT 3	Norway	cohort	incidence	both	62<br>64	192,903	interview/measured	≥130/85mmHg and/or history of	HR 0.82 (0.46–1.46) adenocarcinoma<br>HR 1.62 (0.85–3.08) squamous cell carcinoma	age, sex, BMI, education, smoking status, family cancer history<br>age, sex, BMI, education, smoking status, alcohol intake, family cancer history
Lindkvist B 2014	The Metabolic Syndrome and Cancer (Me-Can) Project	Austria, Norway, Sweden	cohort	incidence	both	114<br>184	578,700	measured	≥140/90 mmHg (adenocarcninoma and squamous cell carcinoma)	HR 0.93(0.63–1.37) adenocarcinoma<br>HR 1.77(1.30–2.42) squamous cell carcinoma	sex, age at baseline and smoking status,z-score of analyzed factors(BMI, mid BP, glucose, cholesterol and triglycerides) and corrected by regression calibration. (Combined estimate 1.37(1.08-1.74)
Lindgren AM 2005	North Karelia Project	Finland	record-linkage	incidence	both	59	20,529	drug-treated or measured	>150/90mmHg (<29 years), 160/95mmHg (30–64 years) and >170/95mmHg (>65 years)	SIR 1.25(0.95–1.61)	-
**STOMACH (hypertension)**											
Assimes TL 2009	Saskatchewan Health Database	Canada	cohort	incidence	both	154<br>70	22,113<br>20,157	computerized database files of Saskatchewan Health	medication use	≥60y RR 0.90(0.76-1.06)<br><60y RR 1.33(1.08-1.65)	age, sex, calender year
Inoue M 2009	Japan Public Health Center-Based Prospective Study	Japan	cohort	incidence	men<br>women	233<br>138	9,548<br>18,156	questionnaire/health examination	≥130/85mmHg and/or medication use	HR 1.01(0.76-1.34)<br>HR 0.83(0.58-1.18)	age, study area, smoking, weekly ethanol intake, total serum cholesterol
Lin Y 2015	CONOR/HUNT 3	Norway	cohort	incidence	both	373	192,903	interview/measured	≥130/85mmHg and/or history of	HR 1.52(1.16–1.98)	age, sex, BMI, education, smoking status, family cancer history
Lindgren AM 2005	North Karelia Project	Finland	record-linkage	incidence	both	201	20,529	drug-treated or measured	>150/90mmHg (<29 years), 160/95mmHg (30–64 years) and >170/95mmHg (>65 years)	SIR 1.02(0.88-1.16)	-

STOMACH (blood pressure)

Batty GD 2003	Whitehall Study	United Kingdom	cohort	mortality	men	157	17,498	per 10 mmHg of SBP	-	HR 1.05(0.98-1.14)	age, employment grade, smoking status, blood pressure-lowering medication, marital status, disease at study entry, body mass index, triceps skinfold thickness, FEV1, cholesterol
Goldbourt U 1986	IIHD Study	Israel	cohort	mortality	men	47	10,059	per 10 mmHg of SBP	-	HR 1.13(1.00-1.28)	age
Grove JS 1991	Hawaiian island of Oahu	USA	cohort	incidence	men	166	8,006	per 10 mmHg of SBP	-	HR 1.00(0.93-1.08)	age, alcohol consumption, smoking

COLON (hypertension)

Aleksandrova K 2011	The European Prospective Investigation into Cancer and Nutrition Study (EPIC)	Europe	nested case-control	incidence	men	218	208	questionnaire/measured	NCEP-ATPIII crieria, (≥130/85 mmHg) or self-reported/medication use	OR 1.25(0.86–1.83)	age, sex, study center, follow-up time since blood collection, time of the day at blood collection and fasting status, menopausal status and phase of menstrual cycle at blood collection, HRT use (for women), smoking status, education, alcohol consumption, physical activity, fiber intake, consumption of fruits and vegetables, red and processed meat, fish, and shellfish
					women	224	205			OR 1.40(0.98–1.98)	
					both	442	413			OR 1.26(0.98-1.61)	
Bowers K 2006	The Alpha-Tocopherol, Beta-Carotene Cancer Prevention Study (ATBC)	Finland	cohort	incidence	men	227	28,983	questionnaire/measured	NCEP-ATPIII crieria, (≥130/85 mmHg)	HR 1.02(0.78-1.34)	age, smoking
Hardell L 1996	Umeå University Hospital Cancer Registry	Sweden	case-control	incidence	both	329	658	questionnaire	self-reported/medication use	OR 1.00(0.70-1.50)	age, physical activity
Inoue M 2009	Japan Public Health Center-Based Prospective (JPHC) Study	Japan	cohort	incidence	men	102	9,548	questionnaire/health examination	≥130/85mmHg and/or medication use	HR 0.99(0.64-1.52)	age, study area, smoking, weekly ethanol intake, total serum cholesterol
					women	106	18,176			HR 1.07(0.71-1.62)	
Ko S 2016	Korean National Health Insurance Service	Korea	cohort	incidence	both	637	99,565	interview/health examination	NCEP-ATPIII, (≥130/85 mmHg) or history of	HR 1.35(1.10-1.66)	age group, smoking status, alcohol intake, regular exercise, other MetS factors
Lindgren AM 2005	North Karelia Project	Finland	record-linkage	incidence	both	148	20,529	drug-treated or measured	>150/90mmHg (<29 years), 160/95mmHg (30–64 years) and >170/95mmHg (>65 years)	SIR 0.85(0.69-1.03)	-
Watanabe Y 2005	The Japan Collaborative Cohort Study (JACC)	Japan	cohort	mortality	men	366	110,792	questionnaire	self-reported	HR 1.01(0.68-1.51)	age, BMI and exercise
					women	382				HR 0.77(0.52-1.13)	

COLON (blood pressure)

Bowers K 2006	The Alpha-Tocopherol, Beta-Carotene Cancer Prevention Study (ATBC)	Finland	cohort	incidence	men	227	28,983	Top/Bottom SBP	-	HR 1.09(0.71-1.68)	age and number of cigarettes smoked per day.
								Top/Bottom DBP	-	HR 0.88(0.56-1.38)	
								per 10mmHg of SBP	-	HR 1.00(0.99-1.02)	
								per 10 mmHg of DBP	-	HR 1.00(0.97-1.03)	
Dyer 1975	The Chicago Peoples Gas Company study	USA	cohort	mortality	men	15	1,233	per 10mmHg of SBP	-	OR 1.27(1.02-1.59)	age
								per 10 mmHg of DBP	-	OR 1.92(1.26-2.91)	
Goldbourt U 1986	IIHD Study	Israel	cohort	mortality	men	17	10,059	per 10mmHg of SBP	-	HR 1.04(0.84-1.29)	age
Grove JS 1991	Hawaiian island of Oahu	USA	cohort	mortality	men	188	8,006	per 10mmHg of SBP	-	HR 1.07(1.00-1.15)	age, alcohol consumption, smoking
								per 10 mmHg of DBP	-	HR 1.09(0.95-1.24)	
								Top/Bottom SBP	-	HR 1.65(0.86-3.19)	age, ethnicity, BMI, alcohol, family history of

Kabat GC 2012	Women's Health Initiative	USA	cohort	incidence	women	65	4,862	Top/Bottom DBP	-	HR 1.12(0.55-2.31)	age, ethnicity, BMI, alcohol, family history of colorectal cancer, physical activity, participation in the observational study or clinical trial, and treatment arm in the clinical trials
								per 10mmHg of SBP	-	HR 1.10(0.95-1.28)	
								per 10 mmHg of DBP	-	HR 1.00(0.74-1.35)	
Morrison DS 2011	Whitehall I Study	United Kingdom	cohort	mortality	men	329	17,949	Top/Bottom SBP	-	HR 0.97(0.73-1.29)	age, height, BMI, smoking, plasma cholesterol, diabetes, physical activity, socioeconomic position
								Top/Bottom DBP	-	HR 1.06(0.80-1.40)	
								per 10mmHg of SBP	-	HR 1.00(0.95-1.06)	
								per 10 mmHg of DBP	-	HR 0.99(0.95-1.04)	

RECTUM (hypertension)

Aleksandrova K 2011	The European Prospective Investigation into Cancer and Nutrition Study (EPIC)	Europe	nested case-control	incidence	men	152	147	questionnaire/measured	NCEP-ATPIII crieria, (≥130/85 mmHg) or self-reported/medication use	OR 1.02(0.61–1.70)	age, sex, study center, follow-up time since blood collection, time of the day at blood collection and fasting status, menopausal status and phase of menstrual cycle at blood collection, HRT use (for women), smoking status, education, alcohol consumption, physical activity, fiber intake, consumption of fruits and vegetables, red and processed meat, fish, and shellfish
					women	109	104			OR 1.25(0.77–2.03)	
					both	261	251			OR 1.07(0.77-1.50)	
Bowers K 2006	The Alpha-Tocopherol, Beta-Carotene Cancer Prevention Study	Finland	cohort	incidence	men	183	28,983	questionnaire/measured	NCEP-ATPIII crieria, (≥130/85 mmHg)	HR 1.40(1.04-1.87)	age, smoking
Inoue M 2009	Japan Public Health Center-Based Prospective Study (JPHC)	Japan	cohort	incidence	men	53	9,548	questionnaire/health examination	≥130/85mmHg and/or medication use	HR 1.18(0.65-2.15)	age, study area, smoking, weekly ethanol intake, total serum cholesterol
					women	51	18,176			HR 0.60(0.34-1.06)	
Lindgren AM 2005	North Karelia Project	Finland	record-linkage	incidence	both	99	20,529	drug-treated or measured	>150/90mmHg (<29 years), 160/95mmHg (30–64 years) and >170/95mmHg (>65 years)	SIR 0.85(0.69-1.03)	-
Watanabe Y 2005	The Japan Collaborative Cohort Study (JACC)	Japan	cohort	mortality	men	318	110,792	questionnaire	self-reported	HR 0.98(0.63-1.51)	age, BMI and exercise
					women	150				HR 1.97(1.13-3.43)	

RECTUM (blood pressure)

Bowers K 2006	The Alpha-Tocopherol, Beta-Carotene Cancer Prevention Study (ATBC)	Finland	cohort	incidence	men	183	28,983	per 10 mmHg of SBP	-	HR 1.01(0.99-1.03)	age and number of cigarettes smoked per day
								per 10 mmHg of DBP	-	HR 1.03(0.99-1.07)	
Goldbourt U 1986	IIHD Study	Israel	cohort	incidence	men	24	10,059	per 10 mmHg of SBP	-	HR 1.17(0.99-1.37)	age
Grove JS 1991	Hawaiian island of Oahu	USA	cohort	mortality	men	95	8,006	per 10 mmHg of SBP	-	HR 0.96(0.87-1.07)	age, alcohol consumption
								per 10 mmHg of DBP	-	HR 0.84(0.70-1.01)	
Morrison DS 2011	Whitehall Study I	United Kingdom	cohort	mortality	men	121	17,949	per 10 mmHg of SBP	-	HR 0.99(0.90-1.09)	age, height, BMI, smoking, plasma cholesterol, diabetes, physical activity, socioeconomic position
								per 10 mmHg of DBP	-	HR 1.09(0.99-1.21)	

COLORECTAL (hypertension)

Ahmed RL 2006	Atherosclerosis Risk in Communities (ARIC) study	USA	cohort	incidence	both	194	14,109	measured	≥130/85 mmHg	HR 1.35(1.00-1.80)	age, gender, family history of colorectal cancer, physical activity, nonsteroidal antiinflammatory drug use, aspirin use, pack-years of cigarette use, and grams of alcohol per week. Also adjusted for HRT in women

Aleksandrova K 2011	The European Prospective Investigation into Cancer and Nutrition Study (EPIC)	Europe	nested case-control	incidence	men	370	355	questionnaire/measured	NCEP-ATPIII crieria, (≥130/85 mmHg) or self-reported/drug-treated	Combined fixed estimate 1.16(0.86-1.57)	age, sex, study center, follow-up time since blood collection, time of the day at blood collection and fasting status, menopausal status and phase of menstrual cycle at blood collection, HRT use (for women), smoking status, education, alcohol consumption, physical activity, fiber intake, consumption of fruits and vegetables, red and processed meat, fish, and shellfish
					women	333	309			Combined fixed estimate 1.35(1.01-1.79)	
					both	703	664			Combined fixed estimate 1.19(0.97-1.45)	
Assimes TL 2009	Saskatchewan Health Database	Canada	cohort	incidence	both	757	22,113	computerized database files of Saskatchewan Health	medication use	≥60y RR 0.82(0.72-0.93)	age, sex, calender year
						421	20,157			<60y RR 1.31(1.13-1.53)	
Bowers K 2006	The Alpha-Tocopherol, Beta-Carotene Cancer Prevention Study (ATBC)	Finland	cohort	incidence	men	410	28,983	questionnaire/measured	NCEP-ATPIII crieria, (≥130/85 mmHg)	HR 1.18(0.96-1.44)	age, smoking
Harding J 2015	Australian and New Zealand Diabetes and Cancer Collaboration (ANZDCC)	Australia/New Zealand	cohort	incidence	both	468	20,468	medical records	≥130/85mmHg and/or medication use	HR 1.29(1.01–1.64)	age, sex, smoking, education
Inoue M 2009	Japan Public Health Center-Based Prospective Study (JPHC)	Japan	cohort	incidence	men	155	9,548	questionnaire/health examination	≥130/85mmHg and/or medication use	Combined fixed estimate 1.05(0.74-1.49)	age, study area, smoking, weekly ethanol intake, total serum cholesterol
					women	157	18,176			Combined fixed estimate 0.88(0.63-1.22)	
Kune GA 1988	Melbourne Colorectal Cancer Study	Australia	case-control	incidence	both	715	727	questionnaire	self-reported, medication use	OR 0.73(0.59-0.91)	age, sex
Lindgren AM 2005	North Karelia Project	Finland	record-linkage	incidence	both	247	20,529	measured or medication use	>150/90mmHg (<29 years), 160/95mmHg (30–64 years) and >170/95mmHg (>65 years)	Combined fixed estimate 0.92(0.81-1.04)	-
Pelucchi C 2010	Provinces of Pordenone, Gorizia and Forlì, urban areas of Milan, Genoa and Naples, Latina (Italy). Canton Vaud (Switzerland).	Italy, Switzerland	case-control	incidence	men	1,310	2,364	medical records	medication use	OR 1.24(1.03-1.48)	age, study centre, education, smoking habit, alcohol drinking, occupational physical activity and non-alcohol energy intake.
					women	946	2,297			OR 0.87(0.71-1.06)	
Shin CM 2017	National Health Insurance Service–National Sample Cohort	Korea	cohort	incidence	men	3,044	212,525	medical records	WHO (BP≥140/90) or medication use	HR 1.09(1.01-1.19)	age, sex, smoking, drinking, exercise, and income
					women	2,064	196,406			HR 1.03(0.93-1.14)	
					both	5,108	408,931			HR 1.05(0.99-1.12)	
Stocks T 2008	The Northern Sweden Health and Disease Cohort	Sweden	nested case-control	incidence	both	306	595	medical records/measured	WHO (BP≥140/90) or medication use	OR 1.30(0.90-1.86)	age, sex, control status, smoking status, fasting time before blood draw, BMI
Stürmer T 2006	The Physicians' Health Study	USA	cohort	incidence	men	494	22,071	medical records (Physicians' Health Study)	NCEP-ATPIII crieria, (≥130/85 mmHg) or medication use	HR 1.10(0.90-1.30)	age ,vigorous exercise, smoking, alcohol consumption, multivitamin use, regular NSAID use, history of arthritis, and consumption of fruits and vegetables
Suadicani P 1993	Copenhagen	Denmark	cohort	incidence	men	51	5,249	interview/health examination	self-reported/medication use	HR 3.50(1.60-7.40)	age, physical activity, smoking, coffee, alcohol, social class
Trevisan M 2001	The Risk Factors and Life Expectancy Project	Italy	cohort	mortality	men	41	21,311	measured (pooled analysis)	WHO (BP≥140/90)	HR 1.30(0.90-1.88)	age, smoking, alcohol consumption, and sex
					women	13	15,991			HR 1.14(0.61-2.15)	
					both	54	37,302			HR 1.28(0.93-1.75)	
Watanabe Y 2005	The Japan Collaborative Cohort Study (JACC)	Japan	cohort	mortality	men	233	110,792	questionnaire	self-reported	Combined fixed estimate 1.00(0.74-1.34)	age, BMI and exercise

Watanabe Y 2005	Cohort Study (JACC)	Japan	cohort	mortality	women	184	110,792	questionnaire	self-reported	Combined fixed estimate<br>1.05(0.76-1.44)	age, BMI and exercise

COLORECTAL (blood pressure)

Bowers K 2006	The Alpha-Tocopherol, Beta-Carotene Cancer Prevention Study	Finland	cohort	incidence	men	410	28,983	Top/Bottom SBP<br>Top/Bottom DBP<br>per 10mmHg of SBP<br>per 10mmHg of DBP	-<br>-<br>-<br>-	HR 1.09(0.79-1.50)<br>HR 1.09(0.78-1.53)<br>HR 1.01(0.99-1.02)<br>HR 1.01(0.99-1.04)	age and number of cigarettes smoked per day
Colangelo L 2002	CHA Detection Project in Industry	USA	cohort	mortality	men	191	20,433	Top/Bottom SBP<br>per 10mmHg of SBP	-<br>-	HR 1.36(0.91-2.05)<br>HR 1.01(0.90-1.13)	age
					women	126	15,149	Top/Bottom SBP<br>per 10mmHg of SBP	-<br>-	HR 1.24(0.66-2.31)<br>HR 1.01(0.97-1.05)	
Kabat GC 2012	Women's Health Initiative	USA	cohort	incidence	women	81	4,862	Top/Bottom SBP<br>Top/Bottom DBP<br>per 10mmHg of SBP<br>per 10mmHg of DBP	-<br>-<br>-<br>-	HR 1.82(0.98-3.38)<br>HR 1.09(0.57-2.11)<br>HR 1.10(1.00-1.22)<br>HR 1.00(0.98-1.28)	age, ethnicity, BMI, alcohol, family history of colorectal cancer, physical activity, participation in the observational study or clinical trial, and treatment arm in the clinical trials
Rosengren A 1998	The multifactor primary preventive trial in Göteborg	Sweden	cohort	incidence	men	150	7,396	Top/Bottom SBP	-	HR 1.46(0.88-2.44)	age
Stocks T 2008	The Northern Sweden Health and Disease Cohort	Sweden	nested case-control	incidence	both	306	595	Top/Bottom SBP<br>Top/Bottom DBP<br>per 10mmHg of SBP<br>per 10mmHg of DBP	-<br>-<br>-<br>-	OR 1.26(0.73-2.18)<br>OR 1.59(0.94-2.70)<br>OR 1.01(0.98-1.04)<br>OR 1.03(0.98-1.09)	age, sex, control status, smoking status, fasting time before blood draw, BMI
Stocks T 2011	The Metabolic Syndrome and Cancer Project (Me-Can)	Austria, Norway, Sweden	cohort	incidence	men	2,834	289,866	Top/Bottom SBP<br>Top/Bottom DBP<br>per 10mmHg of SBP<br>per 10mmHg of DBP	-<br>-<br>-<br>-	HR 1.26(1.00-1.59)<br>HR 1.42(1.08-1.82)<br>HR 1.01(0.99-1.02)<br>HR 1.02(0.99-1.04)	baseline age, body mass index, and smoking status
					women	1,861	288,834	Top/Bottom SBP<br>Top/Bottom DBP<br>per 10mmHg of SBP<br>per 10mmHg of DBP	-<br>-<br>-<br>-	HR 0.79(0.55-1.10)<br>HR 1.00(0.71-1.42)<br>HR 0.99(0.97-1.01)<br>HR 1.00(0.96-1.03)	

LIVER (hypertension)

Assimes TL 2009	Saskatchewan Health Database	Canada	cohort	incidence	both	28	22,113	computerized database files of Saskatchewan Health	medication use	≥60y RR 1.21(0.86-1.70)	age, sex, calender year
						12	20,157			<60y RR 1.18(0.77-1.81)	
Inoue M 2009	Japan Public Health Center-Based Prospective Study (JPHC)	Japan	cohort	incidence	men	74	9,548	questionnaire/health examination	≥130/85mmHg and/or medication use	HR 1.60(0.93-2.75)	age, study area, smoking, weekly ethanol intake, total serum cholesterol
					women	40	18,176			HR 0.68(0.35-1.30)	
Kasmari A 2017	The MarketScan Commercial Claims and Encounters Database	USA	nested case-control	incidence	both	7,473	22,110	The MarketScan Commercial Claims and Encounters Database	medication use	OR 1.23(1.16-1.31)	age, sex, diabetes, hyperlipidemia, hepatitis C, diabetes medication, hyperlipidemia medication
Lindgren AM 2005	North Karelia Project	Finland	record-linkage	incidence	both	44	20,529	measured or medication use	>150/90mmHg (<29 years), 160/95mmHg (30–64 years) and >170/95mmHg (>65 years)	SIR 1.36(0.99-1.82)	age, BMI
Osaki Y 2012	Tottori Prefecture Cohort	Japan	cohort	incidence	both	129	23,625	measured	WHO (BP > 140/90)	HR 1.20(0.82-1.78)	age, smoking, heavy drinking, every component of metabolic syndrome

Turati F 2013	Province of Pordenone and city of Naples	Italy	case-control	incidence	both	185	404	medical records (questionnaire/measured)	history or medication use	OR 1.13(0.61-2.09)	center, sex, age, education, drinking status, maximum lifetime alcohol intake, smoking habits, HBsAg and/or anti-HCV positivity, non-alcohol energy intake
Welzel TM 2011	Surveillance, Epidemiology, and End Results (SEER)-Medicare Database	USA	case-control	incidence	both	3,649	195,953	medical records (SEER database)	NCEP/ATPIII ≥130/85mmHg and/or medication use	≥65y OR 2.22(2.04-2.42)	age,sex,race, location, Medicare/Medicaid dual enrollement

GALLBLADDER/BILIARY TRACT (hypertension)

Assimes TL 2009	Saskatchewan Health Database	Canada	cohort	incidence	both	22<br>69	20,157<br>22,113	computerized database files of Saskatchewan Health	medication use (gall bladder and biliary tract)	<60y RR 1.13(0.82-1.56)<br>≥60y RR 0.98(0.79-1.21)	age, sex, calender year
Borena W 2014	Metabolic Syndrome and Cancer (Me-Can) Project	Austria, Norway, Sweden	cohort	incidence	both	184	578,700	medical records (EPIC)	WHO systolic hypertension >140 mmHg (gall bladder) WHO >90mmHg diastolic hypertension (gall bladder)	HR 0.93(0.66-1.29)<br>HR 0.96(0.74-1.25) not used to avoid double count	smoking status, age at baseline, BMI
Ko S 2016	Korean National Health Insurance Service	Korea	cohort	incidence	both	171	99,565	interview/health examination	NCEP-ATPIII, (≥130/85 mmHg) or history of	HR 0.95(0.56, 1.63)	age group, smoking status, alcohol intake, regular exercise, other MetS factors
Lindgren AM 2005	North Karelia Project	Finland	record-linkage	incidence	both	54	20,529	measured or medication use	>150/90mmHg (<29 years), 160/95mmHg (30–64 years) and >170/95mmHg (>65 years), (gall bladder)	SIR 1.04(0.78-1.35)	-
Rosato V 2016	Milan, Pordenone, Naples	Italy	case-control	incidence	both	159	795	interview	self-reported	OR 0.65(0.39-1.11)	age, sex, study centre, year of interview, education, BMI, smoking, alcohol
Shebl F 2011	42 Hospitals in Shanghai (cases). Shanghai Resident Registry (controls).	China	case-control	incidence	both	368<br>191	959	interview	self-reported	OR 0.69(0.53-0.90) gall bladder, OR 0.61(0.43-0.86) extrahepatic bile duct. Used calculated Fixed Estimate 0.66(0.53-0.81) to avoid double count of controls	age, gender, and body mass index

PANCREAS (hypertension)

Assimes TL 2009	Saskatchewan Health Database	Canada	cohort	incidence	both	74<br>157	20,157<br>22,113	computerized database files of Saskatchewan Health	medication use	<60y RR 1.36(1.09-1.7)<br>≥60y RR 0.91(0.76-1.1)	age, sex, calender year
Eijgenraam P 2013	Netherlands Cohort Study (NLCS)	The Netherlands	cohort	incidence	both	448	5,000	questionnaire	self-reported	HR 0.94(0.75-1.19)	age, sex, smoking, body mass index,level of education, alcohol, and family history of pancreatic cancer
Inoue M 2009	Japan Public Health Center-Based Prospective Study (JPHC)	Japan	cohort	incidence	men<br>women	24<br>41	9,548<br>18,156	questionnaire/health examination	≥130/85mmHg and/or medication use	HR 0.61(0.26-1.41)<br>HR 1.05(0.54-2.03)	age, study area, smoking, weekly ethanol intake, total serum cholesterol
Lindgren AM 2005	North Karelia Project	Finland	record-linkage	incidence	both	123	20,529	drug-treated or measured	>150/90mmHg (<29 years), 160/95mmHg (30–64 years) and >170/95mmHg (>65 years)	SIR 1.07(0.89-1.27)	smoking, antihypertensive drugs at baseline
Rosato V 2011	Pordenone/Milan	Italy	case-control	incidence	both	326	652	interview	medication use	OR 0.77(0.55-1.08)	sex, age, study center, year of interview, education, and tobacco smoking

Tulinius H 1997	The Reykjavík Study	Iceland	cohort	incidence	women	101	11,580	medical records	≥160/95 mmHg or medication use	HR 2.30(1.20-4.50)	age

PANCREAS (blood pressure)

Berrington A 2008	Korean Cancer Prevention Study	Korea	cohort	incidence	both	2,194<br>2,194	631,172<br>631,172	Top/Bottom SBP<br>per 10mmHg of SBP	-<br>-	HR 0.98(0.88-1.10)<br>HR 1.00(0.97-1.04)	age, gender, body mass index, smoking, and fasting serum glucose levels.
Rosengren A 1998	The multifactor primary preventive trial in Göteborg	Sweden	cohort	incidence	men	52	7,396	Top/Bottom SBP	-	HR 1.62(0.65–4.05)	age
Batty GD 2009	The Whitehall Study	United Kingdom	cohort	mortality	men	163	17,898	Top/Bottom SBP<br>per 10mmHg of SBP	-<br>-	HR 0.93(0.62-1.38)<br>HR 0.99(0.91-1.07)	body mass index, plasma cholesterol, physical activity, socioeconomic status, diabetes/blood glucose, marital status, forced expiratory volume in 1 s, height, age at risk, smoking,
Goldbourt U 1986	the IIHD Study	Israel	cohort	mortality	men	21	10,059	per 10mmHg of SBP	-	HR 1.17(0.97-1.40)	age
Grove JS 1991	Hawaiian island of Oahu	USA	cohort	incidence	men	33	8,006	per 10mmHg of SBP	-	HR 0.98(0.84-1.16)	age

TRACHEA/BRONCHUS/LUNG (hypertension)

Assimes TL 2009	Saskatchewan Health Database	Canada	cohort	incidence	both	598<br>408	20,157<br>22,113	computerized database files of Saskatchewan Health	medication use	≥60y RR 0.75(0.6-0.92)<br><60y RR 1.28(1.02-1.6)	age, sex, calender year
Inoue M 2009	Japan Public Health Center-Based Prospective Study (JPHC)	Japan	cohort	incidence	men<br>women	149<br>75	9,548<br>18,176	questionnaire/health examination	≥130/85mmHg and/or medication use	HR 1.17(0.81-1.68)<br>HR 0.75(0.47-1.21)	age, study area, smoking, weekly ethanol intake, total serum cholesterol
Lindgren AM 2005	North Karelia Project	Finland	record-linkage	incidence	both	305	20,529	measured or medication use	>150/90mmHg (<29 years), 160/95mmHg (30–64 years) and >170/95mmHg (>65	SIR 0.86(0.77-0.95)	age, smoking
Lee SY 2002	The Korea Medical Insurance Corporation (KMIC)	Korea	cohort	mortality	men	883	452,645	questionnaire/health examination	≥140/90 mmHg and/or history of	HR 1.30(1.10-1.50)	age, total serum cholesterol, diabetes, BMI, smoking status, alcohol consumption, exercise
Peeters PHM 1998	The DOM Cohort	The Netherlands	cohort	mortality	women	704	11,075	measured	BP>160/95 mmHg	HR 2.19(1.21-3.97)	age, smoking status, and BMI
Tulinius H 1997	The Reykjavík Study	Iceland	cohort	incidence	men<br>women	273<br>199	11,336<br>11,580	medical records	≥160/95 mmHg or medication use	HR 0.70(0.50-1.00)<br>HR 0.70(0.50-0.90)	age

TRACHEA/BRONCHUS/LUNG (blood pressure)

Batty GD 2003	Whitehall Study	United Kingdom	cohort	mortality	men	658	17,498	Top/Bottom SBP<br>per 10mmHg of SBP<br>per 10mmHg of DBP	-<br>-<br>-	HR 0.82(0.67-1.00)<br>HR 0.97(0.93-1.01)<br>HR 0.98(0.92-1.04)	age, employment grade, smoking status, blood pressure-lowering medication, marital status, disease at study entry body mass index, triceps skinfold thickness, FEV1, cholesterol
Dyer 1975	The Chicago Peoples Gas Company study	USA	cohort	mortality	men	28	1,233	per 10mmHg of SBP<br>per 10mmHg of DBP	-<br>-	OR 1.20(1.01-1.43)<br>OR 1.52(1.07-2.16)	age
Goldbourt U 1986	The IIHD Study	Israel	cohort	mortality	men	72	10,059	per 10mmHg of SBP	-	HR 1.01(0.91-1.13)	age
Grove JS 1991	Hawaiian island of Oahu	USA	cohort	incidence	men	190	8,006	per 10mmHg of SBP<br>per 10mmHg of DBP	-<br>-	HR 1.00(0.94-1.09)<br>HR 0.99(0.79-1.04)	age, alcohol consumption, smoking

Lee SY 2002	The Korea Medical Insurance Corporation (KMIC)	Korea	cohort	mortality	men	883	452,645	Top/Bottom SBP<br>per 10mmHg of SBP<br>per 10mmHg of DBP	-<br>-<br>-	HR 1.40(1.00-1.80)<br>HR 1.00(1.00-1.00)<br>HR 1.00(1.00-1.00)	age, BMI, alcohol, diabetes, total serum cholesterol, exercise
Rosengren A 1998	The multifactor primary preventive trial in Göteborg	Sweden	cohort	incidence	men	203	7,396	Top/Bottom SBP	-	HR 0.92(0.58–1.44)	age

BREAST (hypertension)

Agnoli C 2010	The ORDET Cohort	Italy	nested case-control	incidence	women	176	702	interview/measured	mean blood pressure 106.5 mmHg or antihypertensive drug assumption	HR 1.30(0.89-1.89)	age, age at menarche, years from menopause, number of full-term pregnancies, age at first birth, oral contraceptive use, HRT use in the past, years of education, family history of breast cancer, breastfeeding , smoking and alcohol consumption
Agnoli C 2015	The European Prospective Investigation into Cancer and Nutrition Study (EPIC) - 4 (italian centers)	Italy	nested case-control	incidence	women	603<br>286	555<br>250	interview/measured	≥130/85mmHg and/or medication use	HR 1.24(0.95–1.63)<br>HR 1.51(0.96–2.39) for postmenopausal women	age, menopausal status (whole cohort model only), number of full-term pregnancies, age at menarche, smoking status, education, physical activity, alcohol intake; stratified by age and centre
Assimes TL 2009	Saskatchewan Health Database	Canada	cohort	incidence	women	517<br>641	20,157<br>22,113	computerized database files of Saskatchewan Health	medication use	<60y RR 1.49(1.17-1.89)<br>≥60y RR 0.82(0.64-1.04)	age, sex, calender year
Beji NK 2007	Breast and Gynecological Policlinic of Oncological Institute Instabul	Turkey	case-control	incidence	women	405	1,050	interview	self-reported (diagnosis or medication use)	OR 3.44(2.07-5.71)	education, BMI, chronic disease, age at first birth, breastfeeding, age at menarche, HRT use, oral contraceptive use, alcohol use and family history of endometrial cancer in first-degree relatives
Bosco JL 2012	Black Women's Health Study (BWHS)	USA	cohort	incidence	women	1,228	49,172	questionnaire	self-reported plus medication use	HR 0.98(0.69-1.39)<br>HR 1.03(0.63-1.68) for postmenopausal women	age, education, BMI at age 18, vigorous activity
Cook NR 2009	Nurses' Health Study	USA	cohort	incidence	women	1,815	55,625	questionnaire	self-reported	OR 1.17(1.13-1.22)	parity, age at each birth
Franceschi S 1990	Milan	Italy	case-control	incidence	women	2,663<br>413	2,344<br>346	interview	self-reported	OR 1.10(0.90-1.30)<br>OR 1.20(1.00-1.40) for postmenopausal women	terms for medical condition or procedure, age, area of residence, education, age at first birth, menopausal status and, except for severe overweight, BMI
Harding J 2015	Australian and New Zealand Diabetes and Cancer Collaboration (ANZDCC)	Australia/New Zealand	cohort	incidence	women	549	11,031	interview	≥130/85mmHg and/or medication use	HR 1.03(0.86–1.25)	age, sex, smoking, education
Inoue M 2009	Japan Public Health Center-Based Prospective Study (JPHC)	Japan	cohort	incidence	women	120	18,176	questionnaire/health examination	≥130/85mmHg and/or medication use	HR 0.96(0.66-1.41)	age, study area, smoking, weekly ethanol intake, total serum cholesterol
Jung SJ 2013	Seoul Breast Cancer Study (SeBCS)	Korea	case-control	incidence	women	3,242	1,818	questionnaire	self-reported	OR 1.46(1.18-1.83)	age, age of menarche, pregnancy, age of first pregnancy, family history of breast cancer, and menopausal status
Largent JA 2006	Cancer Surveillance Program of Orange County (CSPOC).	USA	case-control	incidence	women	532	131	questionnaire	self-reported/medication use	OR 1.77(1.04-3.03)	age, body mass index (BMI), diabetes, smoking, alcohol use, menopausal status, family history of breast or ovarian cancer, age at first full-term pregnancy and education

Largent JA 2010	California Teachers Study	USA	cohort	incidence	women	4,151	114,549	questionnaire	self-reported/medication use	HR 1.06(0.98-1.16)	race, family history of breast Ca, age at first full-term pregnancy and number of full-term pregnancies combined variable, HRT and menopausal status combined variable, lifetime physical activity, diabetes, BMI index, smoking history, alcohol use, hysterectomy, breastfeeding, and quartiles of percent calories from fat
Li CI 2003	Washington State	USA	case-control	incidence	women	975	1,007	interview	self-reported	OR 1.10(1.00-1.40)	age
Li CI 2013	Seattle	USA	case-control	incidence	women	1,960	891	interview	self-reported	OR 0.95(0.79-1.13)	age, reference year, county, race/ethnicity, recency of alcohol use
Lindgren AM 2005	North Karelia Project	Finland	record-linkage	incidence	women	307	20,529	measured or medication use	>150/90mmHg (<29 years), 160/95mmHg (30–64 years) and >170/95mmHg (>65 years)	SIR 0.94(0.84-1.04)	-
Moseson M 1993	New York City Guttman Breast Diagnostic Institute	USA	case-control	incidence	women	354	747	interview	self-reported	OR 1.00(0.70-1.40)	Age, family history of breast cancer, age at first full-term birth, height, screening variables, null parity, Jewish religion, Latin American birthplace
Noh HM 2013	Health Promotion Center of the Samsung Medical Center	Korea	case-control	incidence	women	270	540	questionnaire/health examination	NCEP-ATPIII crieria, (≥130/85 mmHg) or medication use	OR 0.96(0.67-1.38)	matched by age and menopausal status, adjusted for number of live births, family history of breast cancer , age at menarche, smoking, alcohol drinking , and physical activity
Osaki Y 2012	Tottori Prefecture cohort	Japan	cohort	incidence	women	77	15,386	measured	WHO (BP > 140/90)	HR 1.37(0.82-2.28)	age, smoking status, heavy drinking, and every component of metabolic syndrome (high blood pressure, high triglycerides, low HDL, high glucose, and high BMI)
Peeters PHM 2000	The DOM Cohort	The Netherlands	cohort	incidence	women	523	11,011	measured/questionnaire	SBP >160 mmHg or a DBP >95 mmHg or current use of anti-hypertensive medication	HR 1.14(0.93-1.40)	age, body mass index, height, parity, familial breast cancer, smoking, oral contraceptive use
Pereira A 2012	3 hospitals in Santiago de Chile	Chile	case-control	incidence	women	170	170	questionnaire/measured	NCEP-ATPIII, (≥130/85 mmHg)<br>(JNC VII) ≥140/90	OR 2.55(1.33-4.89)<br>OR 4.18(1.81-9.64) (not used to avoid duplication)	alcohol use, fruit and vegetable intake, physical activity, type 2 diabetes, menopause, use of oral contraceptives, use of hormone replacement therapy, obesity, years of formal education, smoking, number of living births. **adjusted by alcohol use, fruit and vegetable intake, physical activity, type 2 diabetes, use of oral contraceptives, use of hormone replacement therapy, obesity, years of formal education, smoking, number of living births
Reeves KW 2012	Study of Osteoporotic Fractures Cohort	USA	cohort	incidence	women	551	8,956	measured/questionnaire	NCEP-ATPIII, (≥130/85 mmHg)	HR 1.01(0.82-1.24)	age, current hormone use, family history of breast cancer, and other MetS criteria, BMI
Ronco AL 2012 (premenopausal women)	Pereira Rossell Women's Hospital in Montevideo	Uruguay	case-control	incidence	women	253	497	interview/measured	self-reported	OR 1.55(1.03-2.35)	age, education, rural status, age at menarche, number of live births, age at first delivery, years between menarche and first delivery, breastfeeding, oral contraception, family history of breast cancer in 1st degree, family history of breast cancer in 2nd degree and family history of other cancers in 1st degree
Ronco AL 2012 (postmenopausal women)	Pereira Rossell Women's Hospital in Montevideo	Uruguay	case-control	incidence	women	367	545	interview/measured	self-reported	OR 1.49 (0.95-2.33)	age, residence, family history of BC 1ºdegree, age at menarche, number of live births, age at first delivery and number of breastfeeding months

Rosato V 2011	Data from two studies: 1) The greater area of Milan, 2) Six Italian areas and Swiss Canton of Vaud	Italy, Switzerland	case-control	incidence	women	3,869	4,082	interview	medication use	OR 1.19(1.07-1.33)	age, study center, study period, education, alcohol consumption, age at menarche, age at first birth, age at menopause, hormone replacement therapy use, and family history of breast cancer
Sun LM 2015	Taiwanese National Health Insurance program	Taiwan	cohort	incidence	women	-	51,936	medical records (Longitudinal Health Insurance Database 2000) database)	ICD-9-CM Codes (401–405)	HR 0.92(0.76-1.11)	age, sex, urbanization level, occupation, income, diabetes, hyperlipidemia, stroke, ischemic heart disease, chronic obstructive pulmonary disease, alcoholism, alcoholic liver damage, and medication of antihypertensive agents
Thompson WD 1989	Cancer and Steroid Hormone Study	USA	case-control	incidence	women	4,668 (771 nulliparous)<br>4,668 (3,897 parous)	4,635 (604 nulliparous.)<br>4,635 (4,031 parous)	questionnaire	self-reported/medication use	OR 1.08(0.80-1.49) nulliparous<br>OR 0.73(0.59-0.92) parous	age, geographical region<br>age, geographical region, parity, age at first term pregnancy, total duration of breat feeding
Weiss HA 1999	Atlanta, Seattle, New Jersey	USA	case-control	incidence	women	2,173	1,990	questionnaire	diagnosed/medication use	OR 0.86(0.70-1.10)	Age at diagnosis, race. site, menopausal status, age at first birth, number of births, family history, previous breast biopsy, alcohol, BMI, number of mammograms within the 5-year period prior to one year before diagnosis
Wu AH 2017	Surveillance, Epidemiology, and End Results (SEER)-LA County	USA	case-control	incidence	women	2,167	2,035	interview	self-reported	OR 1.11(0.94-1.30)	age, education, income, years of residence in the US among non-US born, interviewer, family history of breast cancer, benign breast diseases, parity, age at menarche, age at and type of menopause, education and BMI

BREAST (blood pressure)

Bjorge T 2010	Me-Can (Metabolic Syndrome and Cancer) Project	Austria, Norway, Sweden	cohort	incidence	women	4,862	288,834	per 10mmHg of SBP<br>per 10mmHg of DBP	-<br>-	HR 1.00(0.93-1.07)<br>HR 1.01(0.99-1.02)	(age) year of birth, smoking, BMI
Kabat GC 2009	Women's Health Initiative (WHI)	USA	cohort	incidence	women	165	4,888	per 10mmHg of SBP<br>per 10mmHg of DBP	-<br>-	HR 1.02(1.00-1.05)<br>HR 1.03(0.99-1.08)	age, education, ethnicity), body mass index, oral contraceptive use, hormone therapy, age at menarche, age at first birth, age at menopause, alcohol, family history of breast cancer, history of breast biopsy, physical activity, energy intake , smoking status, and randomization status in hormone therapy, calcium plus vitamin D, and dietary modification trials
Törnberg SA 1988	Swedish Cancer Registry	Sweden	cohort	incidence	women	1,182	46,570	per 10mmHg of SBP<br>per 10mmHg of DBP	-<br>-	HR 1.03(1.01-1.06)<br>HR 1.05(1.00-1.10)	age, district

CERVIX (hypertension)

Assimes TL 2009	Saskatchewan Health Database	Canada	cohort	incidence	women	24<br>16	22,113<br>20,157	computerized database files of Saskatchewan Health	medication use	≥60y RR 0.76(0.43–1.35)<br><60y RR 0.73(0.4–1.33)	age, sex, calender year

Lindgren AM 2005	North Karelia Project	Finland	record-linkage	incidence	women	29	20,529	drug-treated or measured	>150/90mmHg (<29 years), 160/95mmHg (30–64 years) and >170/95mmHg (>65 years)	SIR 0.84(0.57–1.21)	-
Sun LM 2015	Taiwanese National Health Insurance program	Taiwan	cohort	incidence	women	-	51,936	medical records (Longitudinal Health Insurance Database 2000) database)	ICD-9-CM Codes (401–405)	HR 0.91(0.67. 1.26)	age, sex, urbanization level, occupation, income, and comorbidity of diabetes, hyperlipidemia, stroke, ischemic heart disease, chronic obstructive pulmonary disease, alcoholism, alcoholic liver damage, and medication of antihypertensive agents

ENDOMETRIAL (hypertension)

Assimes TL 2009	Saskatchewan Health Database	Canada	cohort	incidence	women	138<br>142	20,157<br>22,113	computerized database files of Saskatchewan Health	medication use	<60y RR 2.16(1.64-2.85)<br>≥60y RR 0.90(0.65-1.23)	age, sex, calender year
Austin H 1991	Birmingham	United Kingdom	case-control	incidence	women	168	334	interview	self-reported	OR 2.40(1.60-3.60)	age, race, years of schooling
Brinton LA 1992	USA	USA	case-control	incidence	women	405	297	interview	self-reported	OR 0.94(0.60-1.40)	age, years of education, number of births, weight, oral contraceptives, menopausal estrogen use
Cust AE 2007	European Prospective Investigation into Cancer and Nutrition (EPIC)	Europe	nested case-control	incidence	women	284	546	medical records (EPIC)	NCEP-ATPIII crieria, (≥130/85 mmHg)	OR 0.95(0.67-1.34)	age, other MetS metabolic factors
Folsom AR 2003	The Iowa Women's Health Study	USA	cohort	incidence	women	415	23,335	questionnaire	self-reported	HR 1.53(1.26-1.86)	age
Fortuny J 2009	The Estrogen, Diet, Genetics, and Endometrial Cancer (EDGE) Study	USA	case-control	incidence	women	469	467	medical records (EDGE Study)	medication use	OR 0.90(0.60-1.50)	age, BMI, demographic factors (education, race), other estrogen-related variables (menarche, hormone therapy use, oral contraceptives use, age at menopause, parity), smoking, family history of endometrial cancer, diabetes, biguanides, insulin, sulphonylureas, hypercholesterolemia, statins, fibrates, ACE inhibitors, BBs, CCBs, ARBs, thiazide diuretics and its duration of use, loop diuretics, K sparing diuretics, osteoporosis, biphosphonates, calcitonin, endometrial fibroids
Friedenreich C 2011	Alberta Cancer Registry (cases) and population-based controls	Canada	case-control	incidence	women	515	962	interview/measured	NCEP-ATPIII crieria, (≥130/85 mmHg)	OR 1.57(1.18-2.09)	age, MetS factors, age at menarche, number of pregnancies ≥20 wks gestation (0,1, and >1), type of hormone therapy (estrogen,estrogen + progestin, and other HT), leptin, insulin, adiponectin
Furberg AS 2003	Norwegian National Health Screening Service's program	Norway	cohort	incidence	women	130	24,460	questionnaire/measured	WHO criteria, (BP≥140/90)	HR 1.24(0.69-2.25)	age, geographical region, height,BMI, recreational and occupational activity, smoking at baseline and parity
Goodman MT 1997	Hawaii Tumor Registry	Hawaii	case-control	incidence	women	332	511	interview	self-reported	OR 1.40(0.80-2.50)	age, race, pregnancy history, oral contraceptive pill use, unopposed estrogen use, history of diabetes, BMI
Inoue M 1994	Osaka University Medical School	Japan	case-control	incidence	women	143	143	questionnaire/health examination	≥130/85mmHg and/or medication use	OR 1.67(0.57-4.76)	parity, diabetus mellitus, personal cancer history, obesity, age

Lindgren AM 2005	North Karelia Project	Finland	record-linkage	incidence	women	117	12,621	drug-treated or measured	>150/90mmHg (<29 years), 160/95mmHg (30–64 years) and >170/95mmHg (>65 years)	SIR 1.22(1.01-1.44)	age, registration year and BMI
Maatela J 1994	Finnish Cancer Registry (cases), Population Register (controls)	Finland	record-linkage	incidence	women	1,715	1,715	medical records (Finnish Cancer Registry)	medication use	SIR 1.60(1.30-2.10)	age, place of residence
Reis N 2009	Istanbul Medical Faculty Hospital	Turkey	case-control	incidence	women	285	1,050	interview	history of or medication use	OR 3.26(2.21–4.80)	age, education, diabetes, parity, age at menarche, HRT use, first-degree relatives history of breast cancer, endometrial cancer and colorectal cancer, second-degree relatives history of breast cancer and ovarian cancer
Rosato V 2011	Provinces of Milan and Pordenone and the urban area of Naples	Italy	case-control	incidence	women	454	798	interview	self-reported/medication use	OR 1.77(1.34-2.34)	age, study center, year of interview, education, age at menarche, parity, menopausal status, oral contraceptive use, and HTR therapy use
Shao Y 2016	Yantai Yuhuangding Hospital	China	case-control	incidence	women	128	294	interview	self-reported	OR 2.62(0.90–4.40)	Age, time of day of blood collection, CRP, IL-6, TNF-a , insulin, C-peptide, SHBG, birth weight > 4 kg, BMI, WHR, diabetes, age at menarche, FH-cancer
Soliman PT 2006	The University of Texas, M. D. Anderson Cancer Center	USA	case-control	incidence	women	117	238	medical records	not reported	OR 2.64(1.41–4.97)	Age, BMI, diabetes
Sponholtz TR 2016	Black Women's Health Study (BWHS)	USA	cohort	incidence	women	274	47,557	questionnaire	self-reported/drug-treated	HR 1.02(0.78–1.33)	Age, study period, age at menarche, parity, menopausal status, OC use, estrogen-only hormone use, estrogen plus progestin hormone use, smoking status, BMI, vigorous physical activity, statin use, metformin use
Sun LM 2015	Taiwanese National Health Insurance program	Taiwan	cohort	incidence	women	-	51,936	medical records (Longitudinal Health Insurance Database 2000) database)	ICD-9-CM Codes (401–405)	HR 1.88(1.18-3.00)	age, sex, urbanization level, occupation, income, diabetes, hyperlipidemia, stroke, ischemic heart disease, chronic obstructive pulmonary disease, alcoholism, alcoholic liver damage, and medication of antihypertensive agents
Trabert B 2015	Surveillance, Epidemiology, and End Results (SEER)-Medicare Database	USA	case-control	incidence	women	16,323	100,751	medical records	NCEP/ATPIII ≥130/85mmHg and/or medication use	OR 1.21(1.15–1.26)	diagnosis date, age, race/ethnicity, registry area, tobacco use, overweight/obesity, impaired fasting glucose, high blood pressure, high triglycerides
Tulinius H 1997	The Reykjavik Study	Iceland	cohort	incidence	women	98	11,580	medical records	≥160/95 mmHg or medication use	HR 1.90(1.20-2.90)	Univariate analysis
Weiderpass E 2000	Regional cancer registries (cases). Population register (controls)	Sweden	case-control	incidence	women	709	3,368	questionnaire	self-reported	OR 1.10(0.90-1.30)	age, age at menarche, parity, age at last birth, age at menopause, smoking, use of oral contraceptives, and use of HRT, diabetes, BMI
Zhang Y 2010	Zhejiang Province	China	case-control	incidence	women	942	1,721	medical records	WHO criteria, (BP≥140/90) or medication use	OR 6.34(4.53-8.88)	age

OVARY (hypertension)

Assimes TL 2009	Saskatchewan Health Database	Canada	cohort	incidence	women	83<br>54	22,113<br>20,157	computerized database files of Saskatchewan Health	medication use	≥60y RR 0.87(0.66-1.16)<br><60y RR 1.30(0.96-1.78)	age, sex, calender year
Lindgren AM 2005	North Karelia Project	Finland	record-linkage	incidence	women	70	12,621	drug-treated or measured	>150/90mmHg (<29 years), 160/95mmHg (30–64 years) and >170/95mmHg (>65 years)	SIR 0.81(0.63-1.02)	-

Soler M 1999	Network of hospital-based case-control studies conducted in Italy (1983-1996, Pordenone,Naples)	Italy	case-control	incidence	women	970	3,054	interview	self-reported/medication use	OR 0.91(0.73-1.15)	age, area of residence, education, smoking, alcohol intake, parity, menopausal status, BMI

PROSTATE (hypertension)

Assimes TL 2009	Saskatchewan Health Database	Canada	cohort	incidence	men	910<br>511	22,113<br>20,157	computerized database files of Saskatchewan Health	medication use	≥60y RR 0.94(0.73-1.19)<br><60y RR 1.44(1.09-1.90)	age, sex, calender year
Beebe-Dimmer JL 2007	Flint Men's Health Study	USA	case-control	incidence	men	139	359	interview	self-reported/medication use	OR 2.36(1.49-3.73)	age, smoking
Beebe-Dimmer JL 2009	Genes Environment and Prostate Cancer (GECAP) study	USA	case-control	incidence	men	637	244	interview	self-reported	OR 1.02(0.73-1.43)	age, PSA screening history, and 4 remaining features; metabolic syndrome (3 features) adjusted for age and PSA screening history
Blanc-Lapierre A 2015	The Prostate Cancer & Environment Study (PROtEuS)	Canada	case-control	incidence	men	1,937	1,955	questionnaire	self-reported	OR 0.93(0.79-1.08)	age, family history of prostate cancer, ancestry, prostate cancer screening, family income
Fitzpatrick AL 2001	Cardiovascular Health Study (CHS)	USA	cohort	incidence	men	209	2,442	measured	systolic BP≥140	HR 0.70(0.50-0.90)	age, black race, BMI
Friedman G 1997	Kaiser Permanente Medical Care Program (KPMCP)	USA	cohort	incidence	men	2,297	58,704	interview/measured	≥160/95 mmHg, self-reported or medication use	HR 1.04(0.93-1.15)	age, race, BMI, alcohol
Ganesh B 2011	hospital-based, Mumbai-India	India	case-control	incidence	men	123	167	interview	self-reported	OR 2.80(1.20-6.50)	age, religion, education
Harding J 2015	Australian and New Zealand Diabetes and Cancer Collaboration (ANZDCC)	Australia/New Zealand	cohort	incidence	men	651	9,437	interview	≥130/85mmHg and/or medication use	HR 1.05 (0.88–1.25)	age, sex, smoking, education
Inoue M 2009	The Japan Public Health Center-Based Prospective Study (JPHC)	Japan	cohort	incidence	men	119	5,733	questionnaire/health examination	≥130/85mmHg and/or medication use	HR 1.21(0.80-1.83)	age, study area, smoking status, weekly ethanol intake, total serum cholesterol
Lawrence YR 2013	The Bezafibrate Infarction Prevention (BIP) study	USA	cohort	incidence	men	459	11,541	interview/physical examination	≥130/85mmHg and/or history of	HR 1.07(0.9-1.29)	age
Lindgren AM 2005	North Karelia Project	Finland	record-linkage	incidence	men	175	20,529	drug-treated or measured	>150/90mmHg (<29 years), 160/95mmHg (30–64 years) and >170/95mmHg (>65 years)	SIR 1.02(0.87-1.17)	-
Lund Haheim L 2006	The Oslo Study	Norway	cohort	incidence	men	507	15,933	questionnaire/measured	≥130/85mmHg and/or history of	HR 1.12(0.94-2.14)	age
Martin RM 2010	CONOR	Norway	cohort	incidence	men	1,974	78,768	measured/drug-treated	BP≥140/90	HR 0.99(0.89-1.11)	age, height, BMI, smoking, marital status, education, physical activity, diabetes at baseline and country of origin
Oishi K 1989	Kyoto University Hospital, Shiga Medical School Hospital and 11 affiliated hospitals.	Japan	case-control	incidence	men	100	100	interview	self-reported	OR 0.71(0.39-1.32)	matched by age, hospital, admission date
Perron L 2004	Quebec health insurance system	Canada	case-control	incidence	men	2,221	11,105	medical records	medication use	OR 0.98 (0.88-1.08)	age, recent medical contacts, and Aspirin use

Pelucchi C 2011	Greater Milan area, provinces of Pordenone and Gorizia, province of Latina, urban area of Naples	Italy	case-control	incidence	men	1,294	1,451	interview	self-reported/medication use	OR 1.14(0.96-1.36)	age, study center, education, smoking habit, alcohol drinking, occupational physical activity, family history of prostate cancer, and nonalcohol energy intake.
Romero FR 2012	City of Curitiba (PR)	Brazil	cohort	incidence	men	58	2,121	interview/physical examination	self-reported	OR 1.16(0.61-2.23)	age, race, ethnicity, family history, school level, vasectomy, increased blood pressure, diabetes mellitus, and urethritis.
Ronquist G 2004	General Practice Research Database	United Kingdom	nested case-control	incidence	men	1,013	10,000	medical records	medication use	OR 1.00(0.80-1.20)	age, calendar year, smoking, BMI, alcohol, diabetes, IHD, HF, Prostatism
Stocks T 2010	Swedish Construction Workers Cohort	Sweden	cohort	incidence	men	10,002	336,159	measured	≥140/90 mmHg	HR 0.91(0.87-0.94)	year of birth (age), smoking, BMI
Sun LM 2015	Taiwanese National Health Insurance program	Taiwan	cohort	incidence	men	-	59,768	medical records (Longitudinal Health Insurance Database 2000) database)	ICD-9-CM Codes (401–405)	HR 1.20 (0.97. 1.47)	age, sex, urbanization level, occupation, income, diabetes, hyperlipidemia, stroke, ischemic heart disease, chronic obstructive pulmonary disease, alcoholism, alcoholic liver damage, and medication of antihypertensive agents
Tande AJ 2006	The Atherosclerosis Risk in Communities (ARIC) Study	USA	cohort	incidence	men	385	6,429	questionnaire/measured	NCEP/ATPIII ≥130/85mmHg and/or medication use	HR 0.91(0.73-1.14)	age, race, family history, educational level, pack-years of smoking, grams of ethanol per week, total caloric intake, and milk intake
Tulinius H 1997	The Reykjavík Study	Iceland	cohort	incidence	men	524	11,366	medical records	≥160/95 mmHg or medication use	HR 1.20(1.00-1.40)	age
Wallner LP 2010	The Olmsted County Study (OCS) of Urinary Symptoms and Health Status among Men	USA	cohort	mortality	men	206	2,445	questionnaire	self-reported	HR 1.10(0.79-1.40)	age
Weinmann S 2010	The Prostate Cancer Screening and Mortality Study	USA	case-control	mortality	men	768	929	medical records	self-reported	OR 1.20(0.95-1.40)	age, race, health plan, number of months in health plan, reference date and history of prostate cancer screening in 10 years before reference date

PROSTATE (blood pressure)

Batty GD 2011	Whitehall Study	United Kingdom	cohort	mortality	men	578	17,934	Top/Bottom SBP<br>Top/Bottom DBP<br>per 10mmHg of SBP<br>per 10 mmHg of DBP	-<br>-<br>-<br>-	HR 0.93(0.75-1.15)<br>HR 0.98(0.79-1.21)<br>HR 0.98(0.94-1.02)<br>HR 0.99(0.93-1.06)	age at risk, BMI, plasma cholesterol, physical activity, socio-economic status, diabetes/blood glucose, marital status, FEV1, height, smoking
Friedman G 1997	Kaiser Permanente Medical Care Program (KPMCP)	USA	cohort	incidence	men	2,297	58,704	Top/Bottom SBP<br>Top/Bottom DBP<br>per 10mmHg of SBP<br>per 10 mmHg of DBP	-<br>-<br>-<br>-	HR 1.07(0.94-1.23)<br>HR 1.07(0.94-1.23)<br>HR 1.00(1.00-1.01)<br>HR 1.01(0.99-1.02)	age, race, body mass index, and alcohol consumption
Grove JS 1991	Hawaiian island of Oahu	USA	cohort	incidence	men	1,155	8,006	per 10mmHg of SBP<br>per 10 mmHg of DBP	-<br>-	HR 0.98(0.92-1.05)<br>HR 1.01(0.95-1.07)	age
Haggstrom 2012	Me-Can (Metabolic Syndrome and Cancer) Project	Austria, Norway, Sweden	cohort	incidence	men	6,673	289,866	Top/Bottom SBP<br>Top/Bottom DBP<br>per 10mmHg of SBP<br>per 10 mmHg of DBP	-<br>-<br>-<br>-	HR 0.95(0.81-1.12)<br>HR 1.06(0.87-1.28)<br>HR 1.00(0.99-1.01)<br>HR 1.01(0.99-1.03)	baseline age, body mass index, and smoking status
Martin RM 2010	CONOR	Norway	cohort	incidence	men	1,974	78,768	Top/Bottom SBP<br>Top/Bottom DBP<br>per 10mmHg of SBP<br>per 10 mmHg of DBP	-<br>-<br>-<br>-	HR 1.13(0.97-1.31)<br>HR 1.16(1.00-1.36)<br>HR 1.02(1.00-1.05)<br>HR 1.04(1.01-1.08)	age, height, BMI, smoking, marital status, education, physical activity, diabetes at baseline and country of origin
Rosengren A 1998	The multifactor primary preventive trial in Göteborg	Sweden	cohort	incidence	men	263	7,396	Top/Bottom SBP	-	HR 1.27(0.87–1.85)	age

Stocks T 2010	Swedish Construction Workers Cohort	Sweden	cohort	incidence	men	10,002	336,159	Top/Bottom SBP<br>Top/Bottom DBP<br>per 10mmHg of SBP<br>per 10 mmHg of DBP	-<br>-<br>-<br>-	HR 0.84(0.76-0.91)<br>HR 0.82(0.76-0.90)<br>HR 0.99(0.99-1.00)<br>HR 0.98(0.98-0.99)	year of birth, smoking, BMI
Thompson MM 1989	Rancho Bernardo, California	USA	cohort	incidence	men	54	1,776	per 10mmHg of SBP	-	HR 1.00(0.89-1.12)	age

KIDNEY (RENAL CELL) (hypertension)

Assimes TL 2009	Saskatchewan Health Database	Canada	cohort	incidence	both	126<br>156	20,157<br>22,113	computerized database files of Saskatchewan	medication use	<60y RR 1.93(1.59-2.36)<br>≥60y RR 1.17(0.95-1.44)	age, sex, calender year
Brennan P 2008	Hospital based	Europe	case-control	incidence	men<br>women<br>both	647<br>449<br>1,097	951<br>524<br>1,476	interview	self-reported/medication use	OR 1.12(0.90-1.40)<br>OR 1.47(1.11-1.95)<br>OR 1.25(1.06-1.49)	age, smoking, history of hypertension treatment, BMI and country
Choi MY 2005	The National Health Insurance Corporation (NHIC)	Korea	cohort	mortality	men	92	576,562	measured	SBP ≥140 and/or DBP ≥90 mm Hg. (JNC VII criteria)	HR 1.72(0.66-4.49)	age, alcohol consumption, diabetes, total serum cholesterol, body mass index, diet, and exercise
Colt JS 2011	Detroit (SEER member) and Chicago (Cook Country Hospitals)	USA	case-control	incidence	both	1,201	1,226	interview	self-reported/medication use	OR 2.00(1.70-2.50)	study center, race, sex, age, education, smoking status, body mass index, and family history of kidney cancer
Chow W 1995	Minnesota Cancer Surveillance System	USA	case-control	incidence	both	591	691	interview	self-reported/medication use	OR 1.50(1.10-1.90)	age, sex, smoking, BMI, history of hypertension
Finkle 1993	Kaiser Foundation Health Plan	USA	case-control	incidence	women	191	191	medical records	measured SBP ≥140/90 mm Hg or medication use	OR 1.10(0.60-1.90)	age, use of diuretics
Flaherty KT 2005	Nurses' Health Study and Health Professionals Follow-up Study	USA	cohort	incidence	women<br>men	155<br>110	118,191<br>48,953	questionnaire	self-reported	RR 1.90(1.40-2.70)<br>RR 1.80(1.20-2.70)	age, BMI and smoking
Fraser GE 1990	California Seventh-day Adventists	USA	cohort	incidence	both	14	34,198	interview	self-reported	IRR 2.90(1.08-10.27)	age
Fryzek JP 2005	North Jutland County	Denmark	cohort	incidence	both	191	335,682	medical records	medication use	OR 1.60(1.30-1.90)	age, gender and calendar period
Heath CW 1997	Cancer Prevention Study II	USA	cohort	mortality	men<br>women	212<br>123	434,339<br>564,565	medical records/interview	self-reported/medication use	HR 1.10(0.90-1.50)<br>HR 2.20(1.50-3.20)	age
Hofmann J 2015	Kaiser Permanente Northern California medical records (SEER member)	USA	nested case-control	incidence	both	3,136	31,031	medical records (diagnosed, measured)	SBP ≥140 and/or DBP ≥90 mmHg	OR 2.20(1.90-2.40)	smoking, diabetes
Kreiger N 1992	Ontario Cancer Registry	Canada	case-control	incidence	men<br>women	312<br>201	644<br>705	questionnaire	self-reported	OR 1.30(0.70-2.30)<br>OR 1.40(0.80-2.30)	age, sex, region of residence, active cigarette smoking status, diuretic use, and combined Quetelet index
Lai WS 2013	Taiwan National Health Insurance Database	Taiwan	case-control	incidence	both	116	464	medical database	-	OR 2.05(1.23-3.42)	sex, age, diabetes mellitus, hypertension, kidney,infections, chronic kidney diseases, cystic kidney diseases and kidney stones.
Lindgren AM 2005	North Karelia Project	Finland	record-linkage	incidence	men<br>women<br>both	-<br>-<br>113	-<br>-<br>20,529	drug-treated or measured	>150/90mmHg (<29 years), 160/95mmHg (30–64 years) and >170/95mmHg (>65 years)	SIR 1.20(0.86-1.63)<br>SIR 1.43(1.12-1.80)<br>SIR 1.34(1.11-1.60)	age, registration year, use of antihypertensive drugs and BMI
Macleod LC 2013	The VITAL Study	USA	cohort	incidence	both	249	77,260	questionnaire	self-reported	HR 1.70(1.30-2.22)	age, gender, race, BMI, smoking, alcohol consumption, fruit intake tertile, vegetable intake tertile, diabetes, kidney disease, viral hepatitis
McCredie M 1992	New South Wales residents	Australia	case-control	incidence	both	489	523	interview	self-reported/medication use	OR 1.80(1.30-2.30)	age, sex, method of interview

McLaughlin JK 1995	International Renal Cell - Cancer Study	Australia, Denmark, Sweden, Germany, USA	case-control	incidence	both	1,732	2,309	interview	self-reported/medication use	OR 1.40(1.20-1.70)	diuretics and other anti-hypertensive medication
Mellemgaard A 1994	Danish Cancer Registry (cases), Central Population Register (controls)	Denmark	case-control	incidence	men<br>women	226<br>142	237<br>159	interview	self-reported/medication use	OR 1.80(1.00-2.70)<br>OR 1.80(1.10-3.20)	age, smoking, socioeconomic status and body mass index
Nicodemus KK 2004	Iowa Women's Health Study	USA	cohort	incidence	women	124	34,637	interview	self-reported/medication use	RR 1.68(1.18-2.39)	age
Sanfilippo KM 2014	Multiple Risk Factor Intervention Trial (MRFIT) and Women's Health Initiative	USA	cohort	incidence	women	407	156,774	measured	diastolic blood pressure≥90	HR 1.56(1.06-2.29)	ethnicity, BMI, diabetes, smoking, age
Schouten LJ 2005	The Netherlands Cohort Study on Diet and Cancer (NLCS)	The Netherlands	cohort	incidence	both	337	120,852	medical records	self-reported	HR 1.22(0.94-1.58)	age, sex, body mass index, current cigarette smoking at baseline, number of cigarettes smoked per day and years of cigarette smoking
Setiawan VW 2007	The Multiethnic Cohort	USA	cohort	incidence	men<br>women	220<br>127	75,162<br>85,964	questionnaire	self-reported	HR 1.42(1.07-1.87)<br>HR 1.58(1.09-2.28)	age, ethnicity, BMI, smoking, alcohol drinking, and physical activity
Shapiro JA 1999	Members of GHC Washington State	USA	case-control	incidence	men<br>women	155<br>83	261<br>355	medical records	measured/medication use	OR 1.30(0.70-2.50)<br>OR 2.50(1.20-5.10)	BMI, age
Shen T 2015	Shanghai Women’s Health Study (SWHS) and the Shanghai Men’s Health Study (SMHS).	China	nested case-control	incidence	men<br>women<br>both	147<br>124<br>271	1,461<br>1,232<br>2,693	interview	self-reported	OR 1.30(0.90-2.00)<br>OR 1.50(1.00-2.30)<br>OR 1.40(1.10-1.90)	sex, age, calendar time, menopausal status,education, smoking history, current alcohol drinking status, family history of cancer
Sun LM 2015	Taiwanese National Health Insurance program	Taiwan	cohort	incidence	both	-	111,704	medical records (Longitudinal Health Insurance Database 2000) database)	ICD-9-CM Codes (401–405)	HR 1.56(1.13. 2.15)	age, sex, urbanization level, occupation, income, diabetes, hyperlipidemia, stroke, ischemic heart disease, chronic obstructive pulmonary disease, alcoholism, alcoholic liver damage, and medication of antihypertensive agents
Vatten LJ 2007	The HUNT Study	Norway	cohort	incidence	men<br>women	144<br>94	35,688<br>36,728	questionnaire	medication use	HR 1.10(0.60-1.80)<br>HR 1.40(0.80-2.20)	age, blood pressure medication, body mass index, smoking status,education
Wang G 2012	Hospitals of Second Military Medical University	China	case-control	incidence	both	250	299	questionnaire/measured	≥140/90 mmHg	OR 2.68(1.84-3.90)	matched by age and sex
Washio M 2014	The Japan Collaborative Cohort Study (JACC)	Japan	cohort	mortality	both	88	110,585	questionnaire	self-reported	HR 1.40(0.85-2.30)	age, sex
Weikert S 2008	The European Prospective Investigation into Cancer and Nutrition (EPIC)	Europe	cohort	incidence	both	250	296,638	interview/measured	≥140/90 mmHg or medication use	HR 1.34(0.97-1.84)	sex, body mass index, education, duration of smoking, smoking status, and antihypertensive medication, SBP
Weinmann S 1994	Kaiser Permanente Northwest health plan	USA	case-control	incidence	men<br>women	120<br>86	120<br>172	medical records	history of hypertension	OR 2.00(1.10-3.40)<br>OR 1.90(1.03-3.30) (Excluded from meta-analysis, same population sample as Finkle et al. 1993)	matched for sex, race, age, and time period in the plan
Yuan JM 1998	(SEER) cancer registry of Los Angeles County	USA	case-control	incidence	both	1,204	1,204	interview	self-reported or medication use	OR 2.20(1.80-2.60)	sex, age, residence, level of education
Zucchetto A 2007	Provinces of Pordenone and Gorizia, the greater Milan area, province of Latina, urban area of Naples.	Italy	case-control	incidence	both	767	1,534	interview	self-reported	OR 1.74(1.42-2.12)	center, sex, age, and adjusted for calendar period of interview, years of education, smoking habits, body mass index at age 30 years, and family history of kidney cancer in first degree relatives

KIDNEY (RENAL CELL) (blood pressure)

								per 10mmHg of SBP	-	HR 1.06(0.92-1.22)	age, employment grade, smoking status, blood

Batty GD 2003	The Whitehall Study	United Kingdom	cohort	mortality	men	47	17,498	per 10mmHg of DBP	-	HR 1.09(0.88-1.36)	age, employment grade, smoking status, blood pressure-lowering medication, marital status, disease at study entry, body mass index, triceps skinfold thickness, FEV1, cholesterol
								Top/Bottom SBP	-	HR 1.26(0.62-2.59)	
								Top/Bottom DBP	-	HR 1.12(0.54-2.29)	
Choi MY 2005	The National Health Insurance Corporation (NHIC)	Korea	cohort	mortality	men	92	576,562	Top/Bottom SBP	-	HR 3.80(1.70-8.40)	age
								Top/Bottom DBP	-	HR 3.90(1.90-8.00)	
								per 10mmHg of SBP	-	HR 1.06(1.02-1.10)	
								per 10mmHg of DBP	-	HR 1.10(1.04-1.16)	
Chow WH 2000	The Swedish Foundation for Occupational Safety and Health of the Construction Industry	Sweden	cohort	incidence	men	759	363,992	Top/Bottom SBP	-	HR 1.70(1.10-2.60)	age, smoking status, BMI
								Top/Bottom DBP	-	HR 2.20(1.10-4.50)	
								per 10mmHg of SBP	-	HR 1.04(1.01-1.06)	
								per 10mmHg of DBP	-	HR 1.08(1.03-1.14)	
Grove JS 1991	Hawaiian island of Oahu	USA	cohort	incidence	men	17	8,006	per 10mmHg of SBP	-	HR 1.22(1.00-1.49)	age, smoking
								per 10mmHg of DBP	-	HR 1.47(1.04-2.08)	
Haggstrom 2013	Me-Can (Metabolic Syndrome and Cancer) Project	Austria, Norway, Sweden	cohort	incidence	men	592	278,920	Top/Bottom SBP	-	HR 3.40(1.91-6.06)	age, smoking status, BMI
								Top/Bottom DBP	-	HR 3.33(1.85-5.99)	
								per 10mmHg of SBP	-	HR 1.08(1.04-1.12)	
								per 10mmHg of DBP	-	HR 1.13(1.06-1.20)	
					women	263	281,468	Top/Bottom SBP	-	HR 1.58(0.60-4.14)	
								Top/Bottom DBP	-	HR 1.06(0.43-2.62)	
								per 10mmHg of SBP	-	HR 1.02(0.96-1.08)	
								per 10mmHg of DBP	-	HR 0.99(0.90-1.08)	
Rosengren A 1998	The multifactor primary preventive trial in Göteborg	Sweden	cohort	incidence	men	57	7,396	Top/Bottom SBP	-	HR 2.19(0.97-4.98)	age
Sanfilippo KM 2014	Women's Health Initiative	USA	cohort	incidence	women	407	156,774	per 10mmHg of SBP	-	HR 1.03(1.01-1.04)	age, BMI, smoking, diabetes, race
	Multiple Risk Factor Intervention Trial (MRFIT)	USA	cohort	mortality	men	906	353,340	Top/Bottom SBP	-	HR 1.87(1.38-2.53)	age, race, cholesterol, cigarette use, and diabetes status
								Top/Bottom DBP	-	HR 1.39(1.06-1.81)	
								per 10mmHg of SBP	-	HR 1.03(1.03-1.04)	
								per 10mmHg of DBP	-	HR 1.03(1.02-1.05)	
Shapiro JA 1999	Group Health Cooperative of Puget Sound (GHC)	USA	case-control	incidence	men	155	261	Top/Bottom SBP	-	OR 2.00(0.90-4.20)	age, BMI
						155	261	Top/Bottom DBP	-	OR 1.30(0.80-2.30)	
					women	83	355	Top/Bottom SBP	-	OR 2.30(1.00-5.40)	
						83	355	Top/Bottom DBP	-	OR 2.30(1.20-4.40)	
Vatten LG 2007	The HUNT Study	Norway	cohort	incidence	men	144	35,688	Top/Bottom SBP	-	HR 1.00(0.50-1.90)	age, BMI, smoking status, education
								Top/Bottom DBP	-	HR 0.90(0.40-2.00)	
								per 10mmHg of SBP	-	HR 1.00(1.00-1.20)	
								per 10mmHg of DBP	-	HR 1.10(0.90-1.30)	
					women	94	36,728	Top/Bottom SBP	-	HR 2.00(0.90-4.60)	
								Top/Bottom DBP	-	HR 1.60(0.80-3.50)	
								per 10mmHg of SBP	-	HR 1.20(1.10-1.30)	
								per 10mmHg of DBP	-	HR 1.20(1.00-1.50)	
Washio M 2014	The Japan Collaborative Cohort Study (JACC)	Japan	cohort	mortality	both	88	110,585	Top/Bottom SBP	-	HR 2.64(1.12-6.20)	age, sex
								Top/Bottom DBP	-	HR 1.68(0.86-3.29)	
								per 10mmHg of SBP	-	HR 1.08(1.02-1.14)	
								per 10mmHg of DBP	-	HR 1.06(0.98-1.13)	
Weikert S 2008	The European Prospective Investigation into Cancer and Nutrition (EPIC)	Europe	cohort	incidence	both	250	293,638	Top/Bottom SBP	-	HR 2.48(1.53-4.02)	sex , body mass index, education, duration of smoking, and smoking status.
								Top/Bottom DBP	-	HR 2.34(1.54-3.55)	
								per 10mmHg of SBP	-	HR 1.04(1.02-1.07)	
								per 10mmHg of DBP	-	HR 1.04(1.01-1.07)	

URINARY TRACT/RENAL PELVIS (hypertension)

Assimes TL 2009	Saskatchewan Health Database	Canada	cohort	incidence	both	24	22,113	computerized database files of Saskatchewan Health	medication use (urinary tract)	≥60y RR 0.98(0.69-1.38)	age, sex, calender year
						14	20,157			<60y RR 1.7(1.16-2.49)	

Liaw KL 1997	New Jersey, Iowa, Los Angeles County (California)	USA	case-control	incidence	both	502	496	questionnaire	self-reported/medication use(renal pelvis and ureter)	OR 1.30(1.00-1.80)	age, sex, geographic site, smoking
McReadie M 1992	New South Wales	Australia	case-control	incidence	both	147	523	medical records (interview)	self-reported(renal pelvis)	OR 1.20(0.80-1.80)	age, sex, method of interview

BLADDER (hypertension)

Assimes TL 2009	Saskatchewan Health Database	Canada	cohort	incidence	both	270<br>150	22,113<br>20,157	computerized database files of Saskatchewan Health	medication use	≥60y RR 0.92(0.78-1.08)<br><60y RR 1.48(1.23-1.78)	age, sex, calender year
Jiang X 2010	Los Angeles Bladder Cancer Study	USA	case-control	incidence	both	1,585	1,585	interview	self-reported	OR 0.92(0.77-1.11)	age, sex, race, level of education, use of NSAIDs, intake of carotenoids, ever held a high risk occupation,smoking status in reference year, number of years of smoking and number of cigarettes smoked per day
Lindgren AM 2005	North Karelia Project	Finland	record-linkage	incidence	both	113	20,529	drug-treated or measured	>150/90mmHg (<29 years), 160/95mmHg (30–64 years) and >170/95mmHg (>65 years)	SIR 1.10(0.90-1.30)	-
Montella M 2015	Aviano, Milan, Naples, Catania	Italy	case-control	incidence	both	690	665	interview	self-reported	OR 0.88(0.66-1.17)	sex, age, study centre, education, tobacco smoking, MetS components
Sun LM 2015	Taiwanese National Health Insurance program	Taiwan	cohort	incidence	women	-	51,936	medical records (Longitudinal Health Insurance Database 2000) database)	ICD-9-CM Codes (401–405)	HR 1.16(0.88-1.53)	age, sex, urbanization level, occupation, income, diabetes, hyperlipidemia, stroke, ischemic heart disease, chronic obstructive pulmonary disease, alcoholism, alcoholic liver damage, and medication of antihypertensive agents
Xu S 2015	Hospital of Qingdao University	China	case-control	incidence	both	972	1,098	interview/measured	≥140/90mmHg and/or self-reported	OR 1.06(0.87–1.28)	age, smoking status, gender, BMI, hypertension, diabetes mellitus, hypertriglyceridemia, low HDL-cholesterol

BLADDER (blood pressure)

Batty GD 2003	The Whitehall Study	United Kingdom	cohort	mortality	men	92	17,498	per 10 mmHg of SBP<br>per 10 mmHg of DBP	-<br>-	HR 1.06(0.96-1.18)<br>HR 1.08(0.92-1.27)	age, employment grade, smoking status, blood pressure-lowering medication, marital status, disease at study entry body mass index, triceps skinfold thickness, FEV1, cholesterol
Grove JS 1991	Hawaiian island of Oahu	USA	cohort	incidence	men	69	8,006	per 10 mmHg of SBP<br>per 10 mmHg of DBP	-<br>-	HR 1.01(0.90-1.13)<br>HR 1.00(0.82-1.23)	age, smoking
Haggstrom 2011	Me-Can (Metabolic Syndrome and Cancer) Project	Austria, Norway, Sweden	cohort	incidence	men	1,587	289,866	per 10 mmHg of SBP<br>per 10 mmHg of DBP	-<br>-	HR 1.01(0.99-1.03)<br>HR 1.02(0.99-1.05)	smoking, five categories of birth year, age at measurement and quintiles of BMI

BRAIN/CNS (hypertension)

Assimes TL 2009	Saskatchewan Health Database	Canada	cohort	incidence	both	45	20,157	computerized database files of Saskatchewan	medication use	<60y RR 1.27(0.95-1.69)	age, sex, calender year

Assimes TL 2009	Database	Canada	cohort	incidence	both	41	22,113	of Saskatchewan Health	medication use	≥60y RR 0.86(0.61-1.21)	age, sex, calender year
Houben M 2006	PHARMO database	The Netherlands	nested case-control	incidence	both	306	1,108	PHARMO record linkage system	medication use	OR 1.45(1.03-2.04)	age, gender and duration of follow-up
Lindgren AM 2005	North Karelia Project	Finland	record-linkage	incidence	both	65	20,529	drug-treated or measured	>150/90mmHg (<29 years), 160/95mmHg (30–64 years) and >170/95mmHg (>65 years)	SIR 0.86(0.66-1.09)	-
Peeters PHM 1998	The DOM Cohort	The Netherlands	cohort	mortality	women	704	11,075	measured	BP>160/95 mmHg	HR 0.92(0.50–1.69)	age, smoking status, and BMI

BRAIN/CNS (blood pressure)

Batty GD 2003	Whitehall Study	United Kingdom	cohort	mortality	men	47	17,498	Top/Bottom SBP	-	HR 1.07(0.53-2.15)	age, employment grade, smoking status, blood pressure-lowering medication, marital status, disease at study entry body mass index, triceps skinfold thickness, FEV1, cholesterol
Edlinger M 2012	Me-Can (Metabolic Syndrome and Cancer) Project	Austria, Norway, Sweden	cohort	incidence	both	1,312	578 462	Top/Bottom SBP	-	HR 1.45(1.01-2.09)	sex, birth year (in decades), baseline age, smoking status, and quintiles of BMI
Rosengren A 1998	The multifactor primary preventive trial in Göteborg	Sweden	cohort	incidence	men	37	7,396	Top/Bottom SBP	-	HR 1.20(0.39–3.74)	age

Data Management Footnote

We tried to record and depict every possible hypertension and cancer type association. In order to accomplish that we performed grouping of some cancer types examined before data processing. Specifically, we considered liver/hepatocellular, trachea/bronchus/lung, endometrial/corpus uteri, glioma/brain/CNS/nervous system, gall bladder/bile tract/extrahepatic cholangiocarcinoma, kidney/renal cell, renal pelvis/urinary tract/ureter cancers as one. Studies are ordered according to cancer ICD-10 codes and first author name.

Abbreviations: OR, odds ratio; HR, hazard ratio; RR, relative risk; BMI, body mass index; BP, blood pressure; SBP, systolic blood pressure; DBP, diastolic blood pressure; FEV1, forced expiratory volume in one second; MetS, Metabolic Syndrome; HRT, hormone replacement therapy; NSAID, non steroid anti-inflammatory drug; ACE, angiotensin converting enzyme; BB, b-blocker; CCB, calcium channel blocker; ARB, angiotensin receptor blocker; WHR, waist-to-hip ratio; FH, family history; PSA, prostate specific antigen;.

Supplementary Table 2. Quality assessment of the eligible studies, using the "Newcastle-Ottawa" Scale.

CASE-CONTROL STUDIES

		SELECTION				COMPARABILITY	EXPOSURE			
Author Year	Title	Is the case definition adequate?	Representativeness of the cases	Selection of Controls	Definition of Controls	Comparability of cases and controls on the basis of the design or analysis	Ascertainment of exposure	Same method of ascertainment for cases and controls	Non-Response rate	STARS
Austin H 1991	Endometrial Cancer, Obesity, and Body Fat Distribution	a*	a*	b	a*	a*	c	a*	b	5
Beebe-Dimmer JL 2007	Features of the Metabolic Syndrome and Prostate Cancer in African-American Men	a*	a*	a*	a*	a*	c	a*	a*	7
Beebe-Dimmer JL 2009	Racial Differences in Risk of Prostate Cancer Associated With Metabolic Syndrome	a*	a*	b	a*	a*	c	a*	c	5
Beji N.K. 2007	Risk factors for breast cancer in Turkish women: a hospital-based case–control study	a*	a*	b	a*	both**	c	a*	a*	7
Blanc-Lapierre A 2015	Metabolic syndrome and prostate cancer risk in a population-based case–control study in Montreal, Canada	a*	a*	a*	b	a*	c	a*	a*	6
Brennan P 2008	Tobacco smoking, body mass index, hypertension, and kidney cancer risk in central and eastern Europe	a*	a*	b	a*	a*	c	a*	a*	6
Brinton LA 1992	Reproductive, menstrual and medical risk factors for endomerial cancer: Results from a case-control study	a*	a*	a*	a*	a*	c	a*	c	6
Chow W 1995	Risk of Renal Cell Cancer in Relation to Diuretics, Antihypertensive Drugs, and Hypertension	a*	a*	a*	a*	a*	c	a*	c	6
Colt JS 2011	Hypertension and risk of renal cell carcinoma among white and black Americans	a*	a*	a*	a*	both**	d	a*	a*	8
Finkle WD 1993	Increased risk of renal cell cancer among women using diuretics in the United States	a*	a*	a*	a*	a*	a*	a*	a*	8
Fortuny J 2009	Risk of Endometrial Cancer in Relation to Medical Conditions and Medication Use	a*	a*	a*	b	both**	c	a*	a*	7
Franceschi S 1990	Breast Cancer Risk and History of Selected Medical Conditions Linked with Female Hormones	a*	a*	b	a*	a*	c	a*	c	5
Friedenreich CM 2011	Case-Control Study of the Metabolic Syndrome and Metabolic Risk Factors for Endometrial Cancer	a*	a*	a*	a*	a*	c	a*	a*	7
Ganesh B 2011	Risk factors for prostate cancer: An hospital-based case-control study from Mumbai, India	a*	b	b	a*	a*	d	a*	a*	5
Goodman MT 1997	Diet, Body Size, Physical Activity, and the Risk of Endometrial Cancer	a*	a*	a*	a*	a*	c	a*	b	6
Hardell L 1996	Case-control study on colon cancer regarding previous diseases and drug intake	b	a*	a*	a*	a*	d	a*	a*	6
Inoue 1994	A case-control study on risk factors for uterine endometrial cancer in Japan	a*	a*	b	a*	a*	a*	a*	a*	7
Jiang X 2010	Hypertension, diuretics and antihypertensives in relation to bladder cancer	a*	a*	a*	a*	a*	c	a*	a*	7
Jung SJ 2013	Association of Selected Medical Conditions With Breast Cancer Risk in Korea	a*	a*	b	a*	a*	d	a*	a*	6
Kreiger N 1992	Risk factors for renal cell carcinoma: results of a population-based case-control study	a*	a*	a*	a*	a*	d	a*	c	6
Kune GA 1988	Colorectal Cancer Risk, Chronic Illnesses, Operations, andMedications: Case Control Results from the MelbourneColorectal Cancer Study	a*	a*	a*	a*	a*	c	a*	a*	7
Lai WS 2013	Kidney Cancer and Diabetes Mellitus: A Population-Based Case-Control Study in Taiwan	b	a*	a*	a*	a*	a*	a*	a*	7
Largent JA 2006	Hypertension, diuretics and breast cancer risk	b	a*	a*	a*	both**	d	a*	a*	7
Li CI 2003	Relation between Use of Antihypertensive Medications and Risk of Breast Carcinoma among Women Ages 65–79 Years	b	a*	a*	a*	a*	d	a*	b	5
Li CI 2013	Use of anti-hypertensive medications and breast cancer risk among women 55–74 years of age	b	a*	a*	a*	a*	c	a*	a*	6
Liaw K-L 1997	Possible relation between hypertension and cancers of the renal pelvis and ureter.	a*	a*	a*	b	a*	c	a*	c	5
McCredie M 1992	Risk factors for kidney cancer in New South Wales, Australia. II. Urologic disease, hypertension, obesity, and hormonal factors.	a*	a*	a*	b	a*	c	a*	a*	6
McLaughlin JK 1995	INTERNATIONAL RENAL-CELL CANCER STUDY. VIII. ROLE OF DIURETICS, OTHER ANTI-HYPERTENSIVE MEDICATIONS AND HYPERTENSION	a*	a*	a*	b	a*	c	a*	c	5
Mellemgaard A 1994	Risk Factors for Renal Cell Carcinoma in Denmark: Role of Medication and Medical History	a*	a*	a*	b	a*	c	a*	a*	6
Montella M 2015	Metabolic syndrome and the risk of urothelial carcinoma of the bladder: a case-control study	a*	a*	b	a*	a*	c	a*	c	5
Moseson M 1993	The Influence of Medical Conditions Associated with Hormones on the Risk of Breast Cancer	a*	a*	a*	a*	a*	c	a*	a*	7
Noh Hye-Mi 2013	Metabolic factors and breast cancer risk in Korean women.	a*	a*	b	a*	both **	a*	a*	a*	8
Oishi K 1989	Case-Control Study of Prostatic Cancer in Kyoto, Japan: Demographic and Some Lifestyle Risk Factors.	c	a*	b	b	a*	c	a*	c	3
Pelucchi C 2010	Metabolic syndrome is associated with colorectal cancer in men.	a*	a*	b	a*	both **	d	a*	a*	7
Pelucchi C 2011	The Metabolic Syndrome and Risk of Prostate Cancer in Italy.	a*	a*	b	a*	both **	d	a*	a*	7
Pereira A 2012	Hypertension and the Risk of Breast Cancer in Chilean Women: a Case-control Study.	a*	a*	b	a*	both **	a*	a*	c	7
Perron L 2004	Antihypertensive drug use and the risk of prostate cancer* (Canada).	b	a*	a*	b	a*	a*	a*	c	5
Reis N 2009	Risk factors for endometrial cancer in Turkish women: Results from a hospital-based case–control study	a*	a*	b	a*	a*	d	a*	c	5

Ronco AL 2012	Diabetes, Overweight and Risk of Postmenopausal Breast Cancer: A Case-Control Study in Uruguay.	a*	a*	b	a*	a*	c	a*	c	5
Ronco AL 2012	Risk Factors for Premenopausal Breast Cancer: A Case-control Study in Uruguay.	a*	a*	b	a*	a*	c	a*	c	5
Rosato V 2011	Metabolic syndrome and pancreatic cancer risk: a case-control study in Italy and meta-analysis	c	a*	b	b	a*	c	a*	a*	4
Rosato V 2011	Metabolic syndrome and endometrial cancer risk.	a*	a*	b	a*	a*	c	a*	a*	6
Rosato V 2011	Metabolic syndrome and the risk of breast cancer in postmenopausal women.	a*	a*	b	a*	a*	c	a*	a*	6
Rosato V 2016	Medical conditions, family history of cancer, and the risk of biliary tract cancers	a*	a*	b	a*	both**	c	a*	c	6
Shao Y 2016	Insulin is an important risk factor of endometrial cancer among premenopausal women: a case-control study in China	a*	a*	b	a*	a*	c	a*	c	5
Shapiro JA 1999	Hypertension, Antihypertensive Medication Use, and Risk of Renal Cell Carcinoma.	b	a*	a*	a*	a*	d	a*	c	5
Shebl F 2011	Metabolic syndrome and insulin resistance in relation to biliary tract cancer and stone risks: a population-based study in Shanghai, China	a*	a*	a*	a*	a*	c	a*	c	6
Soler M 1999	Hypertension and Hormone-Related Neoplasms in Women.	a*	a*	b	a*	both **	c	a*	a*	7
Soliman PT 2006	Association Between Adiponectin, Insulin Resistance, and Endometrial Cancer	a*	a*	b	a*	a*	c	a*	c	5
Trabert B 2016	Metabolic syndrome and risk of endometrial cancer in the United States: a study in the SEER-Medicare linked database	b	a*	a*	a*	a*	a*	a*	a*	7
Thompson WD 1989	Hypertension, Pregnancy, and Risk of Breast Cancer.	a*	a*	a*	b	a*	c	a*	a*	6
Turati F 2013	Metabolic syndrome and hepatocellular carcinoma risk	a*	a*	b	b	a*	c	a*	a*	5
Wang G 2012	Risk factor for clear cell renal cell carcinoma in Chinese population: A case–control study.	a*	a*	b	a*	a*	c	a*	a*	6
Weiderpass E 2000	Body size in dierent periods of life, diabetes mellitus, hypertension, and risk of postmenopausal endometrial cancer (Sweden).	a*	a*	a*	b	a*	d	a*	c	5
Weinmann S 1994	Use of Diuretics and Other Antihypertensive Medications in Relation to the Risk of Renal Cell Cancer.	a*	a*	a*	b	a*	d	a*	a*	6
Weinmann S 2010	Medical history, body size, and cigarette smoking in relation to fatal prostate cancer.	a*	a*	a*	a*	a*	d	a*	a*	7
Weiss HA 1999	Breast cancer risk in young women and history of selected medical conditions.	b	a*	a*	a*	both**	b*	a*	c	7
Welzel TM 2011	Metabolic Syndrome Increases the Risk of Primary Liver Cancer in the United States: A Study in the SEER-Medicare Database	b	a*	a*	a*	a*	a*	a*	a*	7
Wu AH 2017	Metabolic conditions and breast cancer risk among Los Angeles County Filipina Americans compared with Chinese and Japanese Americans	b	a*	a*	a*	a*	a*	a*	a*	7
Xu S 2015	The association between metabolic syndrome and the risk of urothelial carcinoma of the bladder: a case-control study in China	a*	a*	b	a*	a*	a*	a*	a*	7
Yuan JM 1998	Hypertension, obesity and their medications in relation to renal cell carcinoma	a*	a*	a*	a*	a*	d	a*	c	6
Zhang Y 2010	The association between metabolic abnormality and endometrial cancer: A large case-control study in China.	a*	a*	b	a*	a*	d	a*	a*	6
Zucchetto A 2007	History of treated hypertension and diabetes mellitus and risk of renal cell cancer.	a*	a*	b	a*	both **	d	a*	a*	7

COHORT STUDIES

		SELECTION				COMPARABILITY	OUTCOME			
Author Year	Title	Representativeness of the exposed cohort	Selection of the non exposed cohort	Ascertainment of exposure	Demonstration that outcome of interest was not present at start of study	Comparability of cohorts on the basis of the design or analysis	Assessment of outcome	Was follow-up long enough for outcomes to occur	Adequacy of follow up of cohorts	STARS
Agnoli C 2010	Metabolic syndrome and postmenopausal breast cancer in the ORDET cohort: A nested caseecontrol study	c	a*	a*	a*	both**	b*	a*	b *	8
Agnoli C 2015	Metabolic Syndrome and Breast Cancer Risk: A Case-Cohort Study Nested in a Multicentre Italian Cohort	a*	a*	a*	a*	both**	b*	a*	a*	9
Ahmed R 2006	The Metabolic Syndrome and Risk of Incident Colorectal Cancer	a*	a*	a*	a*	both**	b*	a*	c	8
Aleksandrova K 2011	European Prospective Investigation into Cancer and Nutrition Metabolic Syndrome and Risks of Colon and Rectal Cancer: The Study	a*	a*	a*	a*	both**	b*	a*	a*	9
Assimes TL 2009	Age at incident treatment of hypertension and risk of cancer: a population study	b*	a*	a*	a*	a*	b*	a*	b *	8
Batty GD 2003	Blood pressure and site-specific cancer mortality: evidence from the original Whitehall study	c	a*	a*	b	a*	b*	a*	b*	6
Batty GD 2009	Follow-up of the Original Whitehall Study Risk Factors for Pancreatic Cancer Mortality: Extended	c	a*	a*	b	both**	b*	a*	b*	7
Batty GD 2011	Modifiable risk factors for prostate cancer mortality in London: forty years of follow-up in the Whitehall study	c	a*	a*	b	both**	b*	a*	b*	7
Berrington A 2008	Pancreatic Cancer and Factors Associated with the Insulin Resistance Syndrome in the Korean Cancer Prevention Study	b*	a*	a*	a*	a*	b*	a*	d	7
Bjørge T 2010	Metabolic Syndrome and Breast Cancer in the Me-Can (Metabolic Syndrome and Cancer) Project	a*	a*	a*	a*	a*	b*	a*	b*	8

Borena W 2014	A Prospective Study on Metabolic Risk Factors and Gallbladder Cancer in the Metabolic Syndrome and Cancer (Me-Can) Collaborative Study	a*	a*	a*	a*	a*	b*	a*	b*	8
Bosco JL 2012	Cardiometabolic factors and breast cancer risk in U.S. black women	a*	a*	c	a*	a*	b*	a*	c	6
Bowers K 2006	A Prospective Study of Anthropometric and Clinical Measurements Associated with Insulin Resistance Syndrome and Colorectal Cancer in Male Smokers	b*	a*	a*	a*	a*	b*	a*	d	7
Choi MY 2005	The effect of hypertension on the risk for kidney cancer in Korean men	b*	a*	a*	b	both**	b*	a*	d	7
Chow WH 2000	OBESITY, HYPERTENSION, AND THE RISK OF KIDNEY CANCER IN MEN	c	a*	a*	b	a*	b*	a*	a*	6
Colangelo L 2002	Colorectal Cancer Mortality and Factors Related to the Insulin Resistance Syndrome	b*	a*	a*	b	a*	b*	a*	a*	7
Cook NR 2009	Mammographic Screening and Risk Factors for Breast Cancer	c	a*	c	a*	a*	b*	a*	b*	6
Cust AE 2007	Metabolic syndrome, plasma lipid, lipoprotein and glucose levels, and endometrial cancer risk in the European Prospective Investigation intoCancer and Nutrition (EPIC)	a*	a*	a*	a*	a*	b*	a*	b*	8
Drahos J 2016	Metabolic syndrome in relation to Barrett's esophagus and esophageal adenocarcinoma: Results from a large population-based case-control study in the Clinical Practice Research Datalink	b*	a*	a*	a*	a*	b*	a*	a*	8
Drahos J 2017	Metabolic Syndrome and Risk of Esophageal Adenocarcinoma in Elderly Patients in the United States: An Analysis of SEER Medicare Data	a*	a*	a*	a*	a*	b*	a*	a*	8
Dyer A 1975	HIGH BLOOD-PRESSURE: A RISK FACTOR FOR CANCER MORTALITY ?	a*	a*	a*	b	a*	b*	a*	a*	7
Edlinger M 2012	Blood pressure and othermetabolic syndrome factors and risk of brain tumour in the large population-based Me-Can cohort study	a*	a*	a*	a*	a*	b*	a*	b*	8
Eijgenraam P 2013	Diabetes type II, other medical conditions and pancreatic cancer risk: a prospective study in The Netherlands	a*	a*	c	a*	both**	b*	a*	b*	8
Fitzpatrick A 2001	Hypertension, Heart Rate, Use of Antihypertensives, and Incident Prostate Cancer	a*	a*	a*	a*	a*	c	a*	b*	7
Flaherty K 2005	A prospective study of body mass index, hypertension, and smoking and the risk of renal cell carcinoma (United States)	c	a*	c	a*	a*	b*	a*	b*	6
Folsom AR 2003	Glycemic Index, Glycemic Load, and Incidence of Endometrial Cancer: The Iowa Women's Health Study	a*	a*	c	a*	a*	b*	a*	c	6
Fraser G 1990	Hypertension, Antihypertensive Medication and Risk of Renal Carcinoma in California Seventh-Day Adventists	a*	a*	c	a*	a*	b*	a*	b*	7
Friedman G 1997	Blood Pressure and Heart Rate: No Evidence for a Positive Association with Prostate Cancer	a*	a*	a*	a*	a*	b*	a*	d	7
Fryzek JP 2005	A cohort study of antihypertensive treatments and risk of renal cell cancer	a*	a*	a*	b	a*	b*	a*	d	6
Furberg AS & Thune I 2003	METABOLIC ABNORMALITIES (HYPERTENSION, HYPERGLYCEMIA AND OVERWEIGHT), LIFESTYLE (HIGH ENERGY INTAKE AND PHYSICAL INACTIVITY) AND ENDOMETRIAL CANCER RISK IN A NORWEGIAN COHORT	b*	a*	a*	a*	both**	b*	a*	b*	9
Goldbourt U 1986	Elevated Systolic Blood Pressure as a Predictor of Long-Term Cancer Mortality: Analysis by Site and Histologic Subtype in 10,000 Middle-Aged and Elderly Men	b*	a*	a*	b	a*	b*	a*	b*	7
Grove JS 1991	The Association of Blood Pressure with Cancer Incidence in a Prospective Study	a*	a*	a*	b	a*	b*	a*	b*	7
Haggstrom C 2011	Metabolic syndrome and risk of bladder cancer: prospective cohort study in the metabolic syndrome and cancer project (Me-Can)	a*	a*	a*	a*	a*	b*	a*	b*	8
Haggstrom C 2012	Prospective Study on Metabolic Factors and Risk of Prostate Cancer	a*	a*	a*	a*	a*	b*	a*	b*	8
Haggstrom C 2013	Metabolic Factors Associated with Risk of Renal Cell Carcinoma	a*	a*	a*	a*	a*	b*	a*	b*	8
Heath C 1997	Hypertension, Diuretics, and Antihypertensive Medications as Possible Risk Factors for Renal Cell Cancer	c	a*	c	a*	a*	b*	a*	d	5
Harding J 2015	The metabolic syndrome and cancer: Is the metabolic syndrome useful forpredicting cancer risk above and beyond its individual components?	a*	a*	a*	b	a*	b*	a*	c	6
Hofmann JN 2015	Chronic Kidney Disease and Risk of Renal Cell Carcinoma: Differences by Race	a*	a*	a*	a*	a*	b*	a*	a*	8
Houben M 2006	The association between antihypertensive drugs and glioma	a*	b	a*	a*	a*	b*	a*	a*	7
Inoue M 2009	Impact of metabolic factors on subsequent cancer risk: results from a large-scale population-based cohort study in Japan	a*	a*	a*	a*	a*	a*	a*	b*	8
Kabat GC 2009	A Longitudinal Study of the Metabolic Syndrome and Risk of Postmenopausal Breast Cancer	b*	a*	a*	a*	both**	b*	a*	b*	9
Kabat GC 2012	A longitudinal study of the metabolic syndrome and risk of colorectal cancer in postmenopausal women	b*	a*	a*	a*	both**	b*	a*	b*	9
Kasmari A 2017	Independent of Cirrhosis, Hepatocellular Carcinoma Risk Is Increased with Diabetes and Metabolic Syndrome	a*	a*	a*	a*	a*	b*	b	a*	7
Ko S 2016	Metabolic Risk Profile and Cancer in Korean Men and Women	a*	a*	a*	a*	both**	b*	a*	d	8
Largent J 2010	Hypertension, antihypertensive medication use, and breast cancer risk in the California Teachers Study cohort	a*	a*	c	a*	both**	b*	a*	d	7
Lawrence YR 2013	Association between metabolic syndrome, diabetes mellitus and prostate cancer risk	c	a*	a*	a*	a*	b*	a*	a*	7

Lee SY 2002	Does hypertension increase mortality risk from lung cancer? A prospective cohort study on smoking, hypertension and lung cancer risk among Korean men	b*	a*	a*	a*	both**	b*	b	b*	8
Lin Y 2015	Metabolic syndrome and esophageal and gastric cancer	a*	a*	a*	a*	both**	b*	a*	a*	9
Lindgren AM 2005	Cancer pattern among hypertensive patients in North Karelia, Finland	b*	a*	a*	a*	a*	b*	a*	d	7
Lindkvist B 2014	Metabolic risk factors for esophageal squamous cell carcina and adenocarcinoma: a prospective study of 580000 subjects within the Me-Can project.	a*	a*	a*	a*	a*	b*	a*	b*	7
Lund Haheim L 2006	Metabolic Syndrome Predicts Prostate Cancer in a Cohort of Middle-aged Norwegian Men Followed for 27 Years.	b*	a*	a*	a*	a*	b*	a*	a*	8
Maatela J 1994	The risk of endometrial cancer in diabetic and hypertensive patients: a nationwide record-linkage study in Finland	a*	a*	a*	b	a*	a*	b	d	5
Macleod LC 2013	Risk Factors for Renal Cell Carcinoma in the VITAL Study.	b*	a*	c	a*	both **	b*	a*	b*	8
Martin RM 2010	Blood pressure and risk of prostate cancer: cohort Norway (CONOR).	a*	a*	a*	a*	both**	b*	a*	a*	9
Morrison DS 2011	Risk factors for colonic and rectal cancer mortality: evidence from 40 years' follow-up in the Whitehall I study.	c	a*	a*	b	both**	b*	a*	a*	7
Nicodemus KK 2004	EVALUATION OF DIETARY, MEDICAL AND LIFESTYLE RISK FACTORS FOR INCIDENT KIDNEY CANCER IN POSTMENOPAUSAL WOMEN.	b*	a*	c	a*	a*	b*	a*	c	6
Osaki Y 2012	Metabolic syndrome and incidence of liver and breast cancers in Japan	b*	a*	a*	a*	both**	b*	a*	d	8
Peeters PHM 1998	Hypertension, antihypertensive drugs, and mortality from cancer among women	b*	a*	a*	b	a*	a*	a*	c	6
Peeters PHM 2000	Hypertension and breast cancer risk in a 19-year follow-up study (the DOM cohort)	b*	a*	a*	b	both**	a*	a*	c	7
Reeves KW 2012	Components of Metabolic Syndrome and Risk of Breast Cancer by Prognostic Features in the Study of Osteoporotic Fractures Cohort.	c	a*	a*	a*	a*	a*	a*	b*	7
Romero FR 2012	The significance of biological, environmental, and social risk factors for prostate cancer in a cohort study in Brazil.	c	a*	d	a*	a*	d	b	c	3
Ronquist G 2004	Association Between Captopril, Other Antihypertensive Drugs and Risk of Prostate Cancer.	a*	a*	d	a*	both**	b*	b	d	6
Rosengren A 1998	Hypertension and long-term cancer incidence and mortality among Swedish men.	b*	a*	a*	a*	both**	b*	a*	a*	9
Sanfilippo KM 2014	Hypertension and Obesity and the Risk of Kidney Cancer in 2 Large Cohorts of US Men and Women	b*	a*	a*	b	a*	b*	a*	d	6
Schouten LJ 2005	Hypertension, antihypertensives and mutations in the Von Hippel–Lindau gene in renal cell carcinoma: results from the Netherlands Cohort Study.	b*	a*	c	a*	a*	b*	a*	b*	7
Setiawan VW 2007	Risk Factors for Renal Cell Cancer: The Multiethnic Cohort.	b*	a*	c	a*	both**	b*	a*	b*	8
Shen T 2015	Association of Hypertension and Obesity with Renal Cell Carcinoma Risk: A Report from the Shanghai Men's and Women's Health Studies	a*	a*	b*	a*	both**	b*	a*	a*	9
Shin CM 2017	Association Among Obesity, Metabolic Health, and the Risk for Colorectal Cancer in the General Population in Korea Using the National Health Insurance Service–National Sample Cohort	a*	a*	a*	a*	both**	b*	a*	d	8
Sponholtz TR 2016	Body Size, Metabolic Factors, and Risk of Endometrial Cancer in Black Women	a*	a*	c	a*	both**	b*	a*	c	7
Stocks T 2008	Components of the metabolic syndrome and colorectal cancer risk; a prospective study.	b*	a*	a*	a*	a*	b*	a*	d	7
Stocks T 2010	Blood pressure, body size and prostate cancer risk in the Swedish Construction Workers cohort.	c	a*	a*	a*	a*	b*	a*	b*	7
Stocks T 2011	Metabolic Factors and the Risk of Colorectal Cancer in 580,000 Men and Women in the Metabolic Syndrome and Cancer Project (Me-Can).	a*	a*	a*	a*	a*	b*	a*	b*	8
Stürmer T 2006	Metabolic Abnormalities and Risk for Colorectal Cancer in the Physicians' Health Study.	c	a*	c	a*	both**	a*	a*	b*	7
Suadicani P 1993	Is the Use of Antihypertensives and Sedatives a Major Risk Factor for Colorectal Cancer?	c	a*	a*	b	both**	b*	a*	d	6
Sun Li-Min 2015	Hypertension and Subsequent Genitourinary and Gynecologic Cancers Risk.	a*	a*	a*	a*	a*	b*	a*	c	7
Tande AJ 2006	The Metabolic Syndrome Is Associated with Reduced Risk of Prostate Cancer.	b*	a*	a*	a*	both**	b*	a*	c	8
Thompson MM 1989	Heart disease risk factors, diabetes, and prostatic cancer in an adult community.	c	a*	a*	a*	a*	a*	a*	a*	7
Törnberg SA 1988	Breast cancer risk in relation to serum cholesterol, serum beta-lipoprotein, height, weight, and blood pressure.	b*	a*	a*	b	a*	b*	a*	d	6
Trevisan M 2001	Markers of Insulin Resistance and Colorectal Cancer Mortality.	a*	a*	a*	b	a*	a*	a*	b*	7
Tulinius H 1997	Risk factors for malignant diseases: a cohort study on a population of 22,946 Icelanders.	b*	a*	b*	a*	a*	b*	a*	b*	8
Vatten LJ 2007	Blood pressure and renal cancer risk: the HUNT Study in Norway.	b*	a*	a*	a*	a*	b*	a*	d	7
Wallner LP 2010	The effects of metabolic conditions on prostate cancer incidence over 15 years of follow-up: results from the Olmsted County Study.	b*	a*	c	a*	a*	a*	a*	b*	7
Washio M 2014	Cigarette Smoking and other Risk Factors for Kidney Cancer Death in a Japanese Population: Japan Collaborative Cohort Study for Evaluation of Cancer Risk (JACC study)	b*	a*	c	b	a*	b*	a*	d	5

Watanabe Y 2005	Medical History of Circulatory Diseases and Colorectal Cancer Death in the JACC Study.	b*	a*	c	b	a*	b*	a*	d	5
Weikert S 2008	Blood Pressure and Risk of Renal Cell Carcinoma in the European Prospective Investigation into Cancer and Nutrition.	a*	a*	a*	a*	a*	b*	a*	d	7

Quality Assessment Footnote

We used the "Newcastle-Ottawa Scale" for the quality assessment of the studies included in this meta-analysis. The quality assessment was independently carried out by two investigators (AS, SC) and any discordance in the results was checked over by a third one (KKT). For the consistency of the results we considered the following hypotheses:

• For the "Ascertainment of exposure" section, we considered "secure record" when the study mentioned that blood pressure had been measured.
• The most important factor for "Comparability" was considered the adjustment for "age". In order to give a second "star" to the study the result had to be also adjusted for at least 3/5 of the following risk factors: smoking, family history of cancer, BMI, alcohol and physical activity.
• For the statement "Enough follow-up for outcomes (cancer) to occur", we considered 10 years minimum or 5 years as the mean follow-up.
• The "Different response rate" was considered at least 5%.
• We chose as "Small number of subjects lost to follow-up" a value of < 10% and a "follow-up rate" of ≥ 90% as adequate cohort follow-up.
• Any nested case-control studies were assessed according to the questions of cohort study design.

Supplement Table 3. Meta-analysis of prospective studies for the association between hypertension, systolic or diastolic blood pressure and risk of cancer.

CANCER TYPE	ANALYSIS	SUMMARY FIXED EFFECTS RR (95% CI)	SUMMARY RANDOM EFFECTS RR (95% CI)	P-VALUE (Q TEST)	I^2 TEST	P-VALUE (EGGER'S TEST)	95% PREDICTION INTERVAL	No OF STUDIES USED	No OF CASES	No OF CONTROLS
Oesophagus	HTN (all)	1.14 (1.07-1.23)	1.18 (1.00-1.38)	0.002	67.5%	0.687	0.73-1.89	6	4315	853138
	HTN (ACC)	1.11 (1.02-1.20)	1.11 (1.01-1.21)	0.365	5.5%	0.570	0.87-1.40	4	3935	790339
	HTN (SCC)	1.74 (1.32-2.30)	1.74 (1.32-2.30)	0.808	0.00%	-	-	2	248	771603
Stomach	HTN	1.06 (0.97-1.15)	1.08 (0.91-1.28)	0.004	71.2%	0.663	0.64-1.83	4	1169	284406
	SBP (per 10mmHg)	1.04 (0.99-1.09)	1.05 (0.98-1.11)	0.230	32.0%	0.337	0.60-1.82	3	370	35563
Colon	HTN	1.07 (0.98-1.17)	1.07 (0.94-1.21)	0.118	39.2%	0.711	0.78-1.46	6	2410	288006
	HTN (-RL)	1.13 (1.01-1.27)	1.10 (0.96-1.27)	0.195	30.6%	0.035	0.80-1.53	5	2262	267477
	SBP (per 10mmHg)	1.01 (0.90-1.03)	1.03 (0.99-1.07)	0.124	42.1%	0.060	0.93-1.14	6	841	71047
	DBP (per 10mmHg)	1.00 (0.98-1.03)	1.03 (0.96-1.10)	0.028	63.3%	0.145	0.85-1.24	5	824	60988
	T/B SBP	1.06 (0.85-1.33)	1.07 (0.85-1.36)	0.342	6.9%	0.151	0.19-5.94	3	621	51794
	T/B DBP	1.02 (0.81-1.27)	1.02 (0.81-1.27)	0.760	0.0%	0.888	0.24-4.39	3	621	51794
Rectum	HTN	1.02 (0.90-1.17)	1.07 (0.84-1.37)	0.010	64.2%	0.512	0.53-2.19	5	1115	188279
	HTN (-RL)	1.17 (0.99-1.39)	1.14 (0.88-1.49)	0.051	54.7%	0.634	0.53-2.46	4	965	149574
	SBP (per 10mmHg)	1.01 (0.99-1.03)	1.01 (0.97-1.05)	0.273	23.0%	0.708	0.88-1.15	4	423	64997
	DBP (per 10mmHg)	1.03 (1.01-1.07)	1.01 (0.92-1.12)	0.047	67.4%	0.692	0.35-2.98	3	399	54938
Colorectal	HTN	1.06 (1.01-1.10)	1.11 (1.01-1.21)	0.000	69.3%	0.114	0.81-1.52	13	9942	739687
	HTN (-RL)	1.07 (1.03-1.12)	1.13 (1.03-1.24)	0.000	67.9%	0.118	0.82-1.56	12	9695	719158
	HTN men	1.11 (1.04-1.19)	1.13 (1.02-1.26)	0.160	33.6%	0.141	0.90-1.43	8	4798	410834
	HTN women	1.05 (0.96-1.14)	1.05 (0.95-1.16)	0.372	6.2%	0.699	0.86-1.28	5	2751	341674
	SBP (per 10mmHg)	1.01 (1.00-1.01)	1.01 (1.00-1.01)	0.396	4.0%	0.336	0.99-1.02	5	5809	388722
	DBP (per 10mmHg)	1.01 (1.00-1.03)	1.01 (1.00-1.03)	0.757	0.0%	0.976	0.99-1.04	4	5492	353140
	T/B SBP	1.19 (1.04-1.36)	1.19 (1.02-1.40)	0.268	20.3%	0.449	0.87-1.64	6	5959	396118
	T/B DBP	1.21 (1.05-1.38)	1.23 (1.04-1.45)	0.397	1.7%	0.762	0.93-1.62	4	5618	353140
Liver/HCC	HTN	1.23 (1.16-1.30)	1.23 (1.16-1.30)	0.607	0.00%	0.761	1.14-1.33	5	7800	136258
	HTN (-RL)	1.23 (1.16-1.30)	1.23 (1.16-1.30)	0.537	0.00%	0.592	1.13-1.33	4	7756	115728
Gallbladder/ Biliary tract	HTN	1.01 (0.88-1.15)	1.01 (0.88-1.15)	0.929	0.00%	0.968	0.81-1.25	4	500	741064
Pancreas	HTN	1.06 (0.96-1.16)	1.08 (0.90-1.30)	0.017	61.1%	0.714	0.65-1.79	5	968	107083
	SBP (per 10mmHg)	1.00 (0.97-1.03)	1.00 (0.97-1.03)	0.407	0.0%	0.514	0.94-1.07	4	2411	667135
	T/B SBP	0.98 (0.88-1.09)	0.98 (0.88-1.09)	0.543	0.0%	0.580	0.49-1.96	3	2409	656466
Trachea/ Bronchus/ Lung	HTN	0.96 (0.89-1.03)	0.97 (0.80-1.19)	0.000	83.2%	0.774	0.51-1.87	6	3594	577159
	SBP (per 10mmHg)	1.00 (0.96-1.03)	1.03 (0.97-1.10)	0.033	61.9%	0.022	0.84-1.28	5	1831	489441
	DBP (per 10mmHg)	1.00 (0.95-1.05)	1.03 (0.93-1.14)	0.098	52.4%	0.134	0.71-1.50	4	1759	479382
	T/B SBP	0.97 (0.83-1.13)	1.02 (0.71-1.46)	0.013	77.1%	0.719	0.01-72.26	3	1744	477539
Breast	HTN (overall)	1.12 (1.08-1.15)	1.07 (0.99-1.15)	0.000	65.4%	0.266	0.84-1.35	13	9443	404268
	HTN (postmen.)	1.01 (0.89-1.16)	1.06 (0.86-1.29)	0.110	47.0%	0.248	0.59-1.89	5	2672	81193
	SBP (per 10mmHg)	1.03 (1.01-1.04)	1.03 (1.01-1.04)	0.684	0.0%	0.076	0.91-1.15	3	6209	340292
	DBP (per 10mmHg)	1.01 (1.00-1.03)	1.02 (1.00-1.05)	0.166	44.3%	0.079	0.79-1.33	3	6209	340292
Cervix	HTN	0.84 (0.68-1.04)	0.84 (0.68-1.04)	0.903	0.00%	0.022	0.53-1.34	3	69	114735
Endometrial	HTN	1.36 (1.25-1.49)	1.37 (1.14-1.64)	0.000	73.9%	0.910	0.75-2.49	9	3313	216020
	HTN (-RL)	1.38 (1.23-1.53)	1.37 (1.07-1.75)	0.000	77.6%	0.904	0.61-3.05	7	1481	201684
Prostate	HTN	0.96 (0.93-0.99)	1.03 (0.96-1.10)	0.001	60.0%	0.009	0.83-1.28	16	20336	653106
	HTN (-RL)	0.96 (0.93-0.99)	1.03 (0.96-1.11)	0.001	61.9%	0.013	0.82-1.30	15	9885	298750
	SBP (per 10mmHg)	1.00 (0.99-1.00)	1.00 (0.99-1.01)	0.096	44.2%	0.557	0.98-1.02	7	22733	801206
	DBP (per 10mmHg)	0.99 (0.99-1.00)	1.00 (0.99-1.02)	0.002	74.4%	0.158	0.96-1.06	6	22679	799447
	T/B SBP	0.95 (0.90-1.01)	1.00 (0.98-1.13)	0.003	71.6%	0.170	0.68-1.46	6	21787	788827
	T/B DBP	0.94 (0.89-1.00)	1.00 (0.86-1.17)	0.000	82.1%	0.127	0.58-1.75	5	21524	781431
Kidney	HTN (all)	1.60 (1.52-1.70)	1.54 (1.39-1.70)	0.000	63.1%	0.224	1.04-2.28	18	6739	2636817
	HTN (men)	1.29 (1.13-1.48)	1.29 (1.13-1.48)	0.492	0.00%	0.442	1.08-1.54	7	925	938735
	HTN (women)	1.63 (1.44-1.84)	1.63 (1.44-1.84)	0.658	0.00%	0.599	1.39-1.90	8	1154	998091
	HTN (r. cell)	1.66 (1.55-1.78)	1.55 (1.36-1.76)	0.000	64.7%	0.104	1.00-2.41	12	5721	2408489
	SBP (per 10mmHg)	1.04 (1.03-1.05)	1.05 (1.03-1.06)	0.008	56.6%	0.036	1.00-1.09	9	2753	2513199
	DBP (per 10mmHg)	1.05 (1.03-1.06)	1.07 (1.04-1.10)	0.015	54.7%	0.029	0.99-1.14	9	2346	2356425
	T/B SBP	1.93 (1.64-2.26)	1.94 (1.60-2.36)	0.200	24.8%	0.711	1.26-3.00	9	3530	2074763
	T/B DBP	1.73 (1.46-2.05)	1.80 (1.36-2.38)	0.019	54.6%	0.703	0.81-4.01	8	3473	2349035
Bladder	HTN	1.13 (1.02-1.24)	1.14 (0.92-1.42)	0.002	79.2%	0.704	0.44-2.98	3	533	114735
	SBP (per 10mmHg)*	1.01 (0.99-1.03)	1.01 (0.99-1.03)	0.650	0.0%	0.502	0.90-1.14	3	1748	315370
	DBP (per 10mmHg)*	1.02 (0.99-1.05)	1.02 (0.99-1.05)	0.771	0.0%	0.690	0.83-1.26	3	1748	315370

Brain/CNS	HTN	1.05 (0.91-1.21)	1.06 (0.84-1.33)	0.061	55.7%	0.907	0.52-2.14	4	1161	74982
	T/B SBP	1.35 (0.99-1.84)	1.35 (0.99-1.84)	0.736	0.0%	0.431	0.18-10.07	3	1396	603356

Footnote

Some studies were used two or more times in the same meta-analysis (of a specific cancer type), since they contained data from different population samples that could not be combined (e.g. men and women separately, patients ≥60 years and < 60 years etc.). The ICD-10 codes for cancer used were: Oesophagus (C15), Stomach (C16), Colon (C18, C19), Rectum (C20), Colorectal (C18, C19, C20), Liver/HCC (C22), Gallbladder/ECC/Biliary Tract (C23, C24), Pancreas (C25), Trachea/Bronchus/Lung (C33, C34), Breast (C50), Cervix (C53), Endometrial (C54), Prostate (C61), Kidney (C64), Bladder (C67), Brain/CNS (C70, C71, C72).
*The meta-analyses for the association between bladder cancer and 10mmHg increase in SBP/DBP included only men.

Abbreviations: HTN, hypertension; SBP, systolic blood pressure; DBP, diastolic blood pressure; T/B, top vs. bottom analysis; -RL, record linkage studies not included; HCC, hepatocellular cancer; ECC, extrahepatic cholangiocarcinoma; CNS, central nervous system.

Supplement Table 4. Meta-analysis of prospective and case-control studies for the association between hypertension, systolic or diastolic blood pressure and risk of cancer.

Hypertension								
CANCER TYPE	SUMMARY FIXED EFFECTS RR (95% CI)	SUMMARY RANDOM EFFECTS RR (95% CI)	P-VALUE (Q TEST)	I-SQUARE	P-VALUE (EGGER'S TEST)	STUDIES	CASE-CONTROL	95% PREDICTION INTERVAL
Colon	1.07 (0.98-1.17)	1.06 (0.95-1.20)	0.168	31.2%	0.643	7	1	0.82-1.39
Colorectal (all)	1.04 (1.00-1.08)	1.08 (0.99-1.18)	0.000	72.8%	0.258	15	2	0.77-1.50
Colorectal (men)	1.13 (1.06-1.20)	1.15 (1.05-1.26)	0.162	32.0%	0.110	9	1	0.94-1.41
Colorectal (women)	1.02 (0.94-1.10)	1.02 (0.91-1.14)	0.216	29.1%	0.856	6	1	0.78-1.34
Liver	1.47 (1.41-1.54)	1.30 (0.99-1.71)	0.000	93.9%	0.758	7	2	0.52-3.29
Gallbladder/Biliary Tract	0.88 (0.79-0.99)	0.90 (0.75-1.07)	0.039	54.8%	0.790	6	2	0.55-1.46
Pancreas	1.03 (0.94-1.13)	1.04 (0.87-1.23)	0.010	62.2%	0.906	6	1	0.63-1.70
Breast overall	1.11 (1.04-1.19)	1.11 (1.04-1.19)	0.000	70.2%	0.805	28	15	0.85-1.45
Breast postmenopausal	1.14 (1.06-1.22)	1.13 (1.02-1.25)	0.100	37.5%	0.601	11	7	0.89-1.45
Endometrial	1.29 (1.25-1.34)	1.58 (1.35-1.85)	0.000	88.0%	0.032	22	13	0.77-3.22
Ovary	0.93 (0.82-1.06)	0.94 (0.78-1.13)	0.110	50.3%	0.296	3	1	0.46-1.91
Prostate	0.97 (0.95-1.00)	1.05 (0.99-1.12)	0.000	65.8%	0.001	24	8	0.83-1.33
Kidney (all)	1.63 (1.56-1.70)	1.60 (1.48-1.73)	0.000	61.3%	0.533	32	14	1.11-2.32
Kidney (men)	1.29 (1.16-1.44)	1.29 (1.16-1.44)	0.433	1.1%	0.044	12	5	1.13-1.47
Kidney (women)	1.59 (1.43-1.77)	1.59 (1.43-1.77)	0.689	0.00%	0.665	13	6	1.41-1.79
Renal Pelvis/Urinary tract	1.26 (1.06-1.50)	1.26 (1.06-1.57)	0.214	33.1%	0.866	3	2	0.61-2.60
Bladder	1.06 (0.98-1.14)	1.06 (0.92-1.22)	0.003	69.9%	0.998	6	3	0.68-1.65
SBP/DBP								
Kidney T/B SBP	1.94 (1.66-2.26)	1.95 (1.65-2.31)	0.320	12.2%	0.619	10	1	1.43-2.66
Kidney T/B DBP	1.71 (1.47-2.00)	1.78 (1.40-2.26)	0.027	49.2%	0.634	9	1	0.89-3.54

Footnote

The ICD-10 codes for cancer used were: Colon (C18, C19), Rectum (C20), Colorectal (C18, C19, C20), Liver/HCC (C22), Gallbladder/ECC/Biliary Tract (C23, C24), Pancreas (C25), Breast (C50), Endometrial (C54), Ovarian (C56), Prostate (C61), Kidney (C64), Renal Pelvis/Urinary Tract (C65, C66), Bladder (C67).

Abbreviations: T/B, top vs bottom analysis; SBP, systolic blood pressure; DBP, diastolic blood pressure.

Outcome	Number of Studies	Random Effects	95% Prediction Interval	I^2	Egger's p
**DBP Top vs Bottom**					
Colon	3	1.02 (0.81, 1.27)	0.24, 4.39	0	0.888
Colorectal	4	1.23 (1.04, 1.45)	0.93, 1.62	1.7	0.762
Prostate	5	1.00 (0.86, 1.17)	0.58, 1.75	82.1	0.127
Kidney	8	1.80 (1.36, 2.38)	0.81, 4.01	54.6	0.703
**SBP Top vs Bottom**					
Colon	3	1.07 (0.85, 1.36)	0.19, 5.94	6.9	0.151
Colorectal	6	1.19 (1.02, 1.40)	0.87, 1.64	20.3	0.449
Pancreas	3	0.98 (0.88, 1.09)	0.49, 1.96	0	0.58
Trachea/ Bronchus/ Lung	3	1.02 (0.71, 1.46)	0.01, 72.26	77.1	0.719
Prostate	6	1.00 (0.88, 1.13)	0.68, 1.46	71.6	0.17
Kidney	9	1.94 (1.60, 2.36)	1.26, 3.00	24.8	0.711
Brain/CNS	3	1.35 (0.99, 1.84)	0.18, 10.07	0	0.431

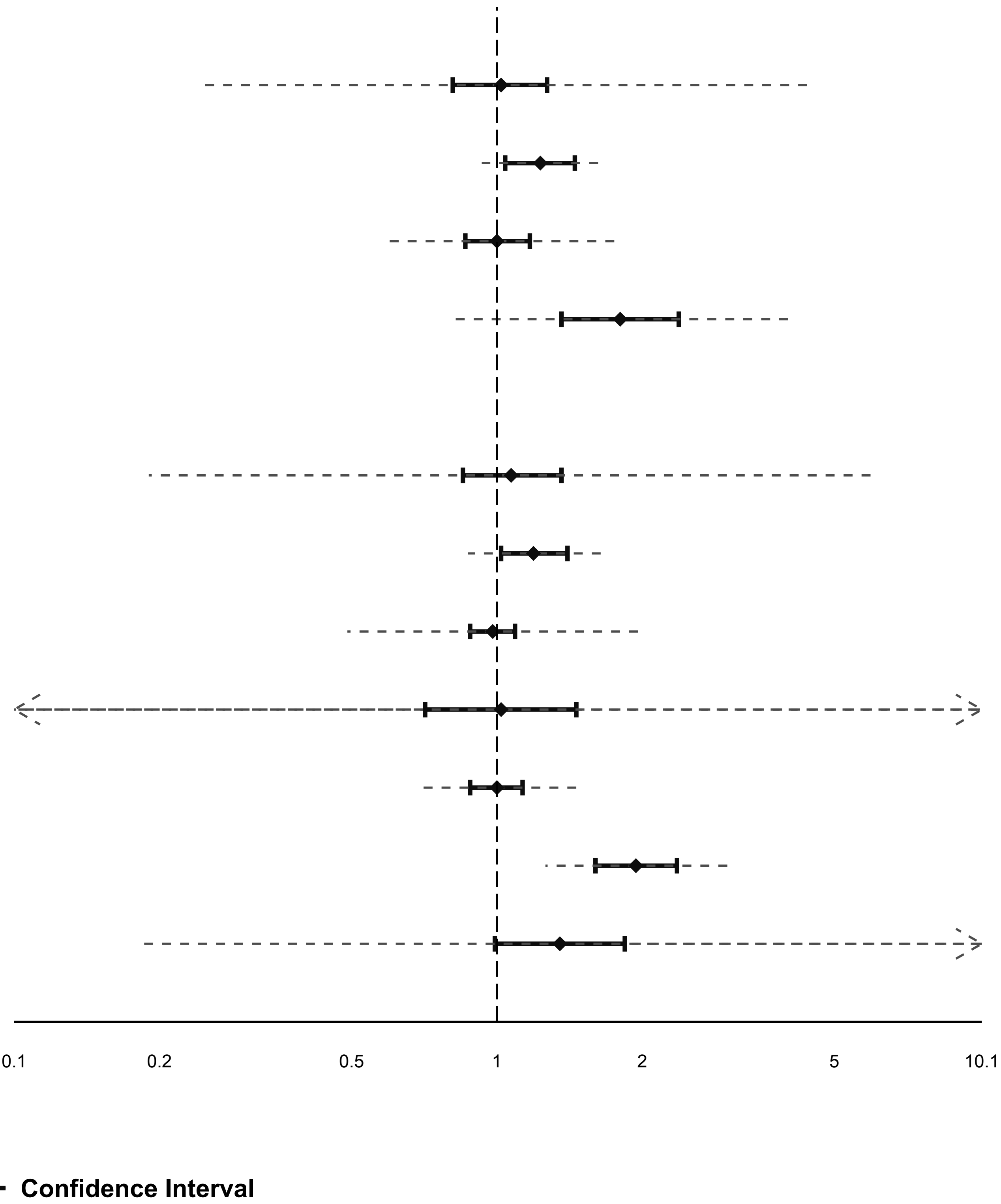

Supplement Figure 1. Summary relative risks and 95% confidence intervals of prospective studies for the association between cancer risk and systolic and diastolic blood pressure (Top vs Bottom). Abbreviations: DBP, diastolic blood pressure; SBP, systolic blood pressure.

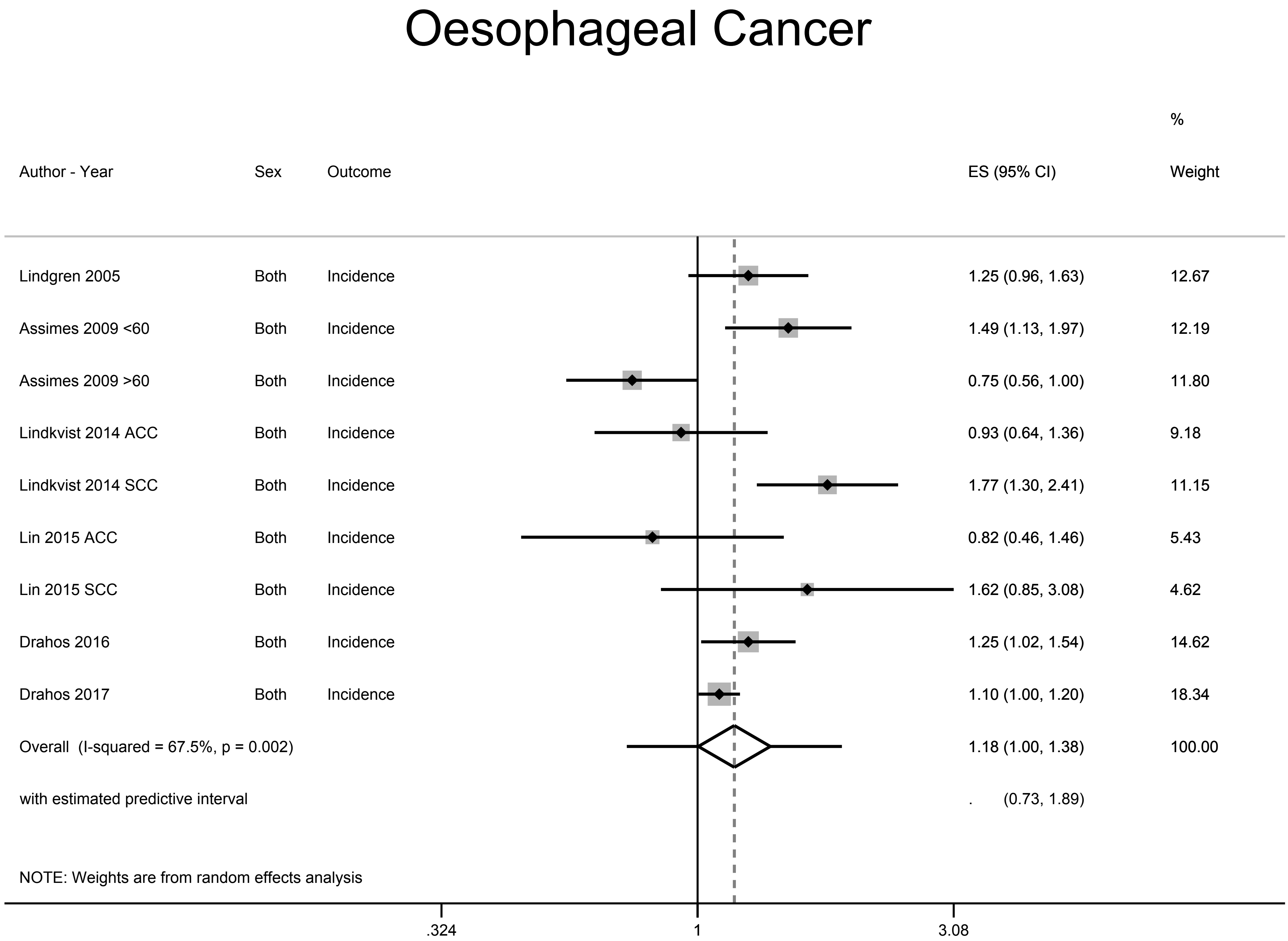

Supplement Figure 2. Meta-analysis of prospective studies for the association between hypertension and oesophageal cancer risk.

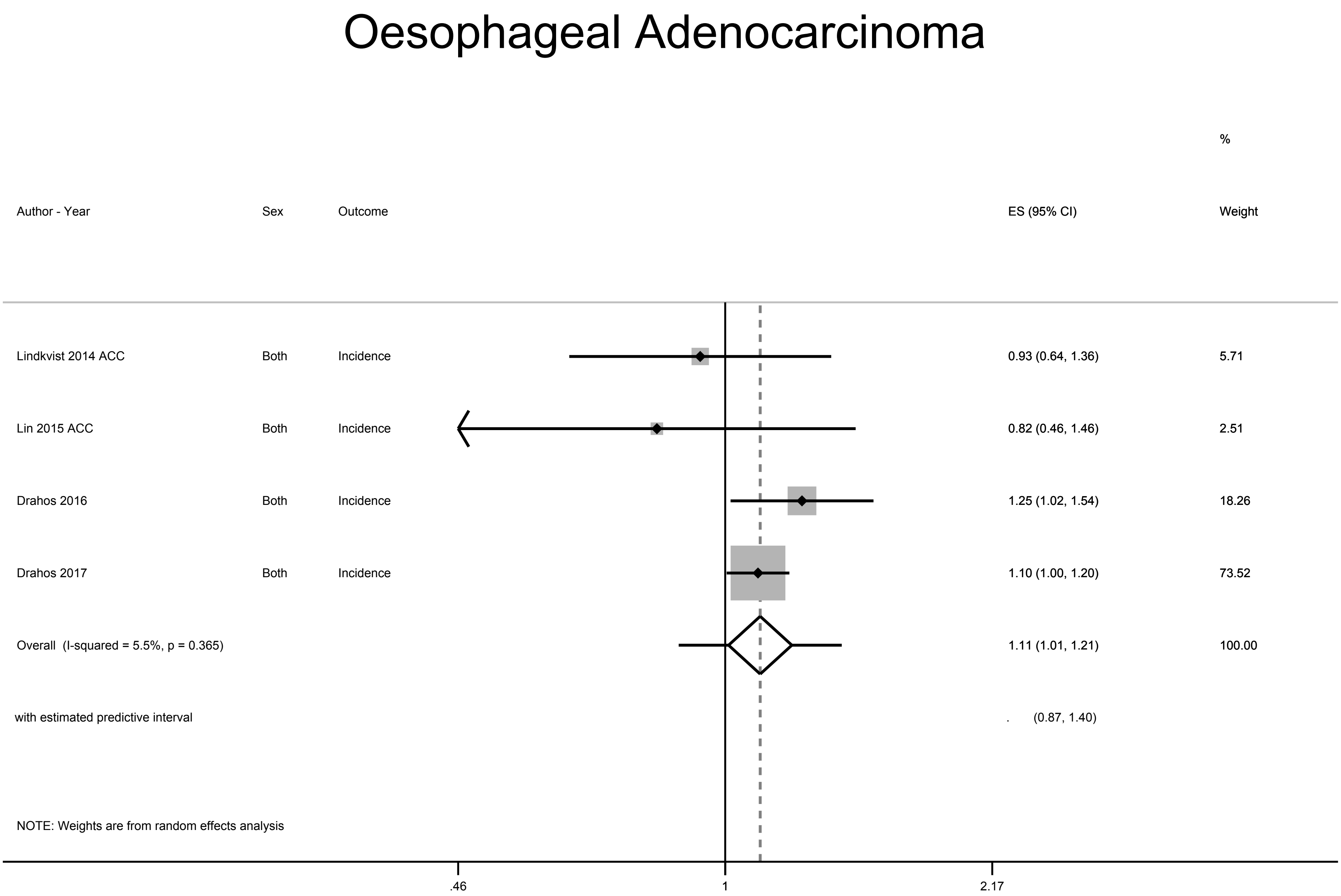

Supplement Figure 3. Meta-analysis of prospective studies cancer for the association between hypertension and oesophageal adenocarcinoma risk.

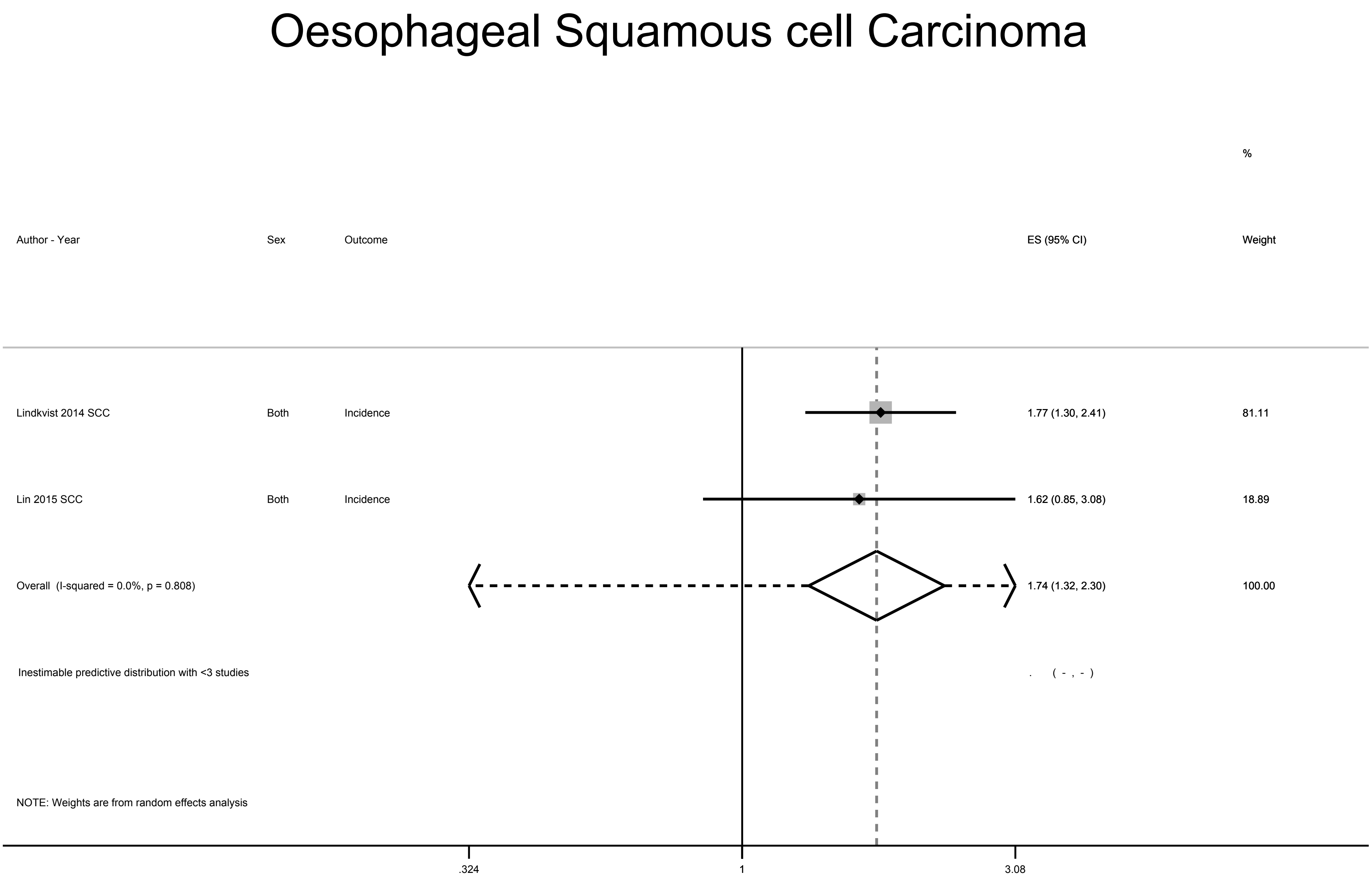

Supplement Figure 4. Meta-analysis of prospective studies for the association between hypertension and oesophageal squamous cell carcinoma risk.

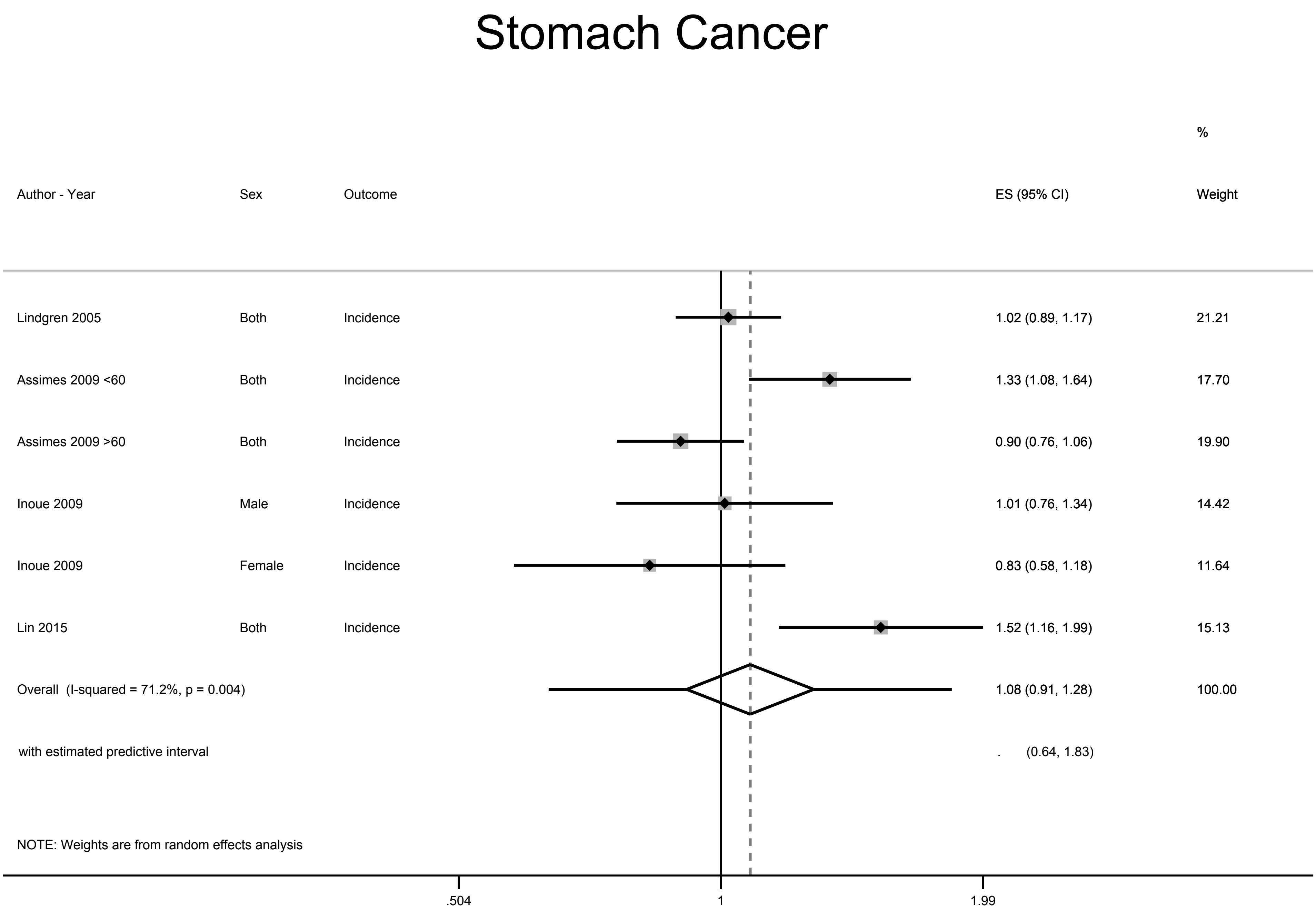

Supplement Figure 5. Meta-analysis of prospective studies for the association between hypertension and stomach cancer risk.

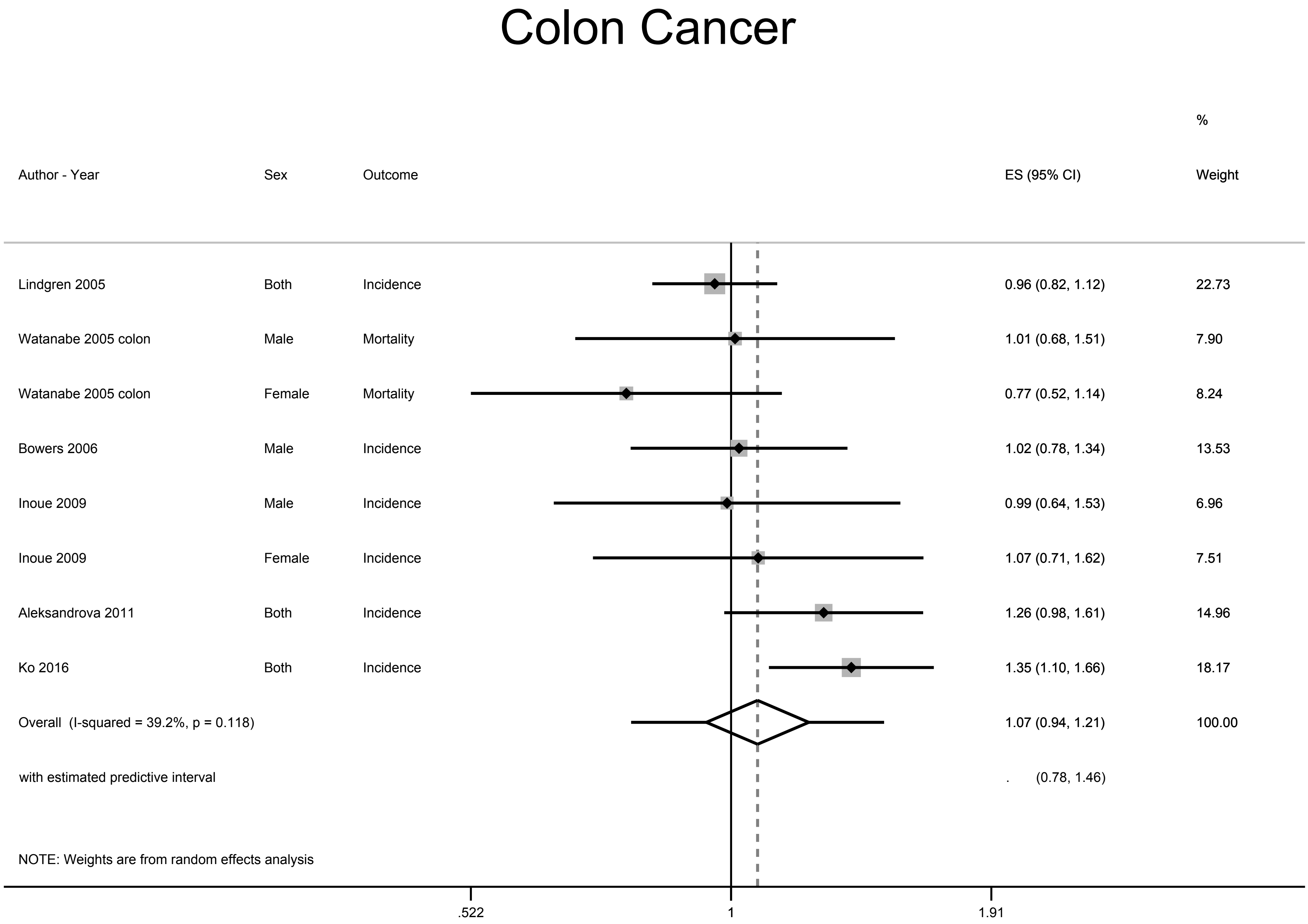

Supplement Figure 6. Meta-analysis of prospective studies for the association between hypertension and colon cancer risk.

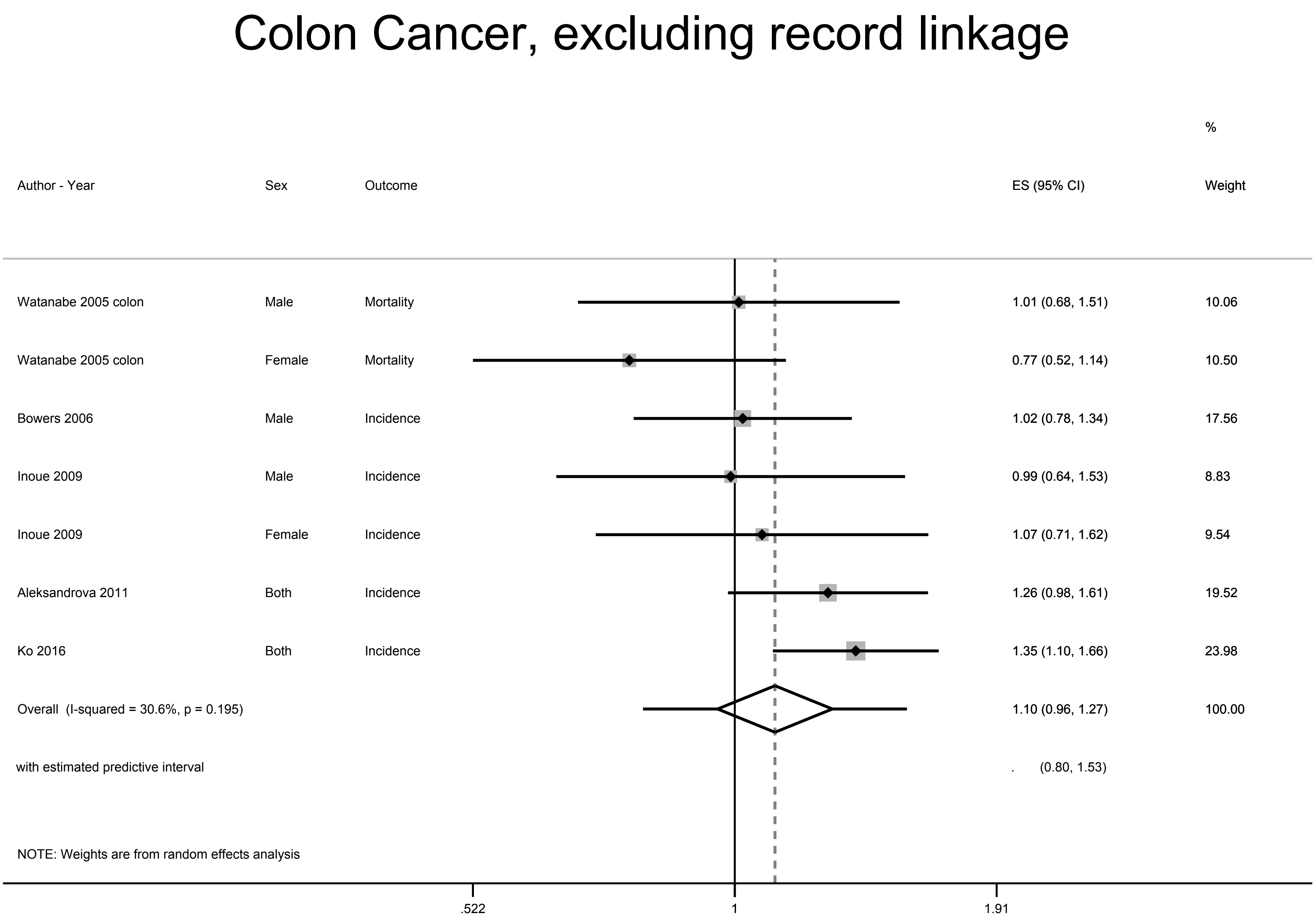

Supplement Figure 7. Meta-analysis of prospective studies for the association between hypertension and colon cancer risk, excluding record-linkage studies.

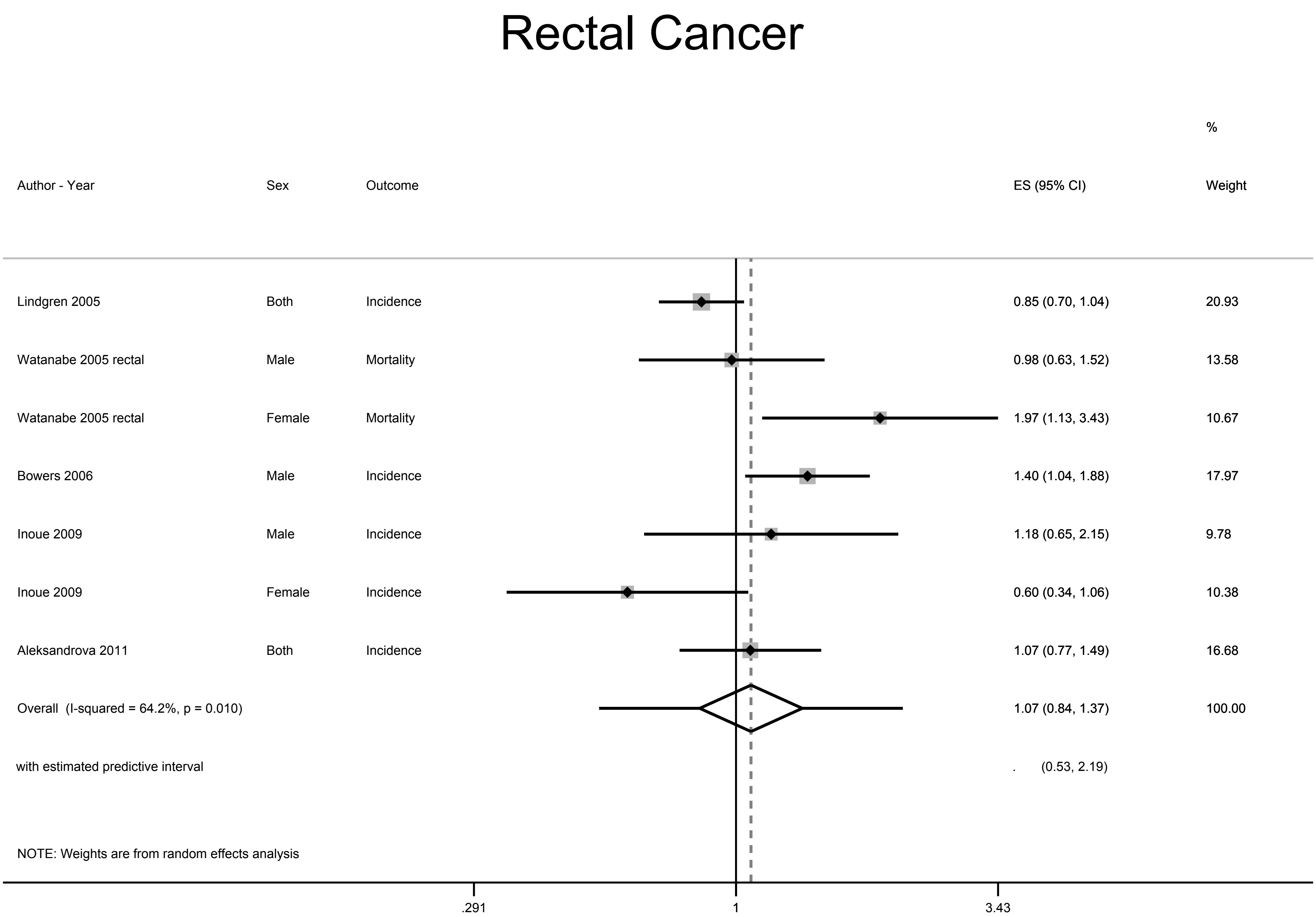

Supplement Figure 8. Meta-analysis of prospective studies for the association between hypertension and rectal cancer risk.

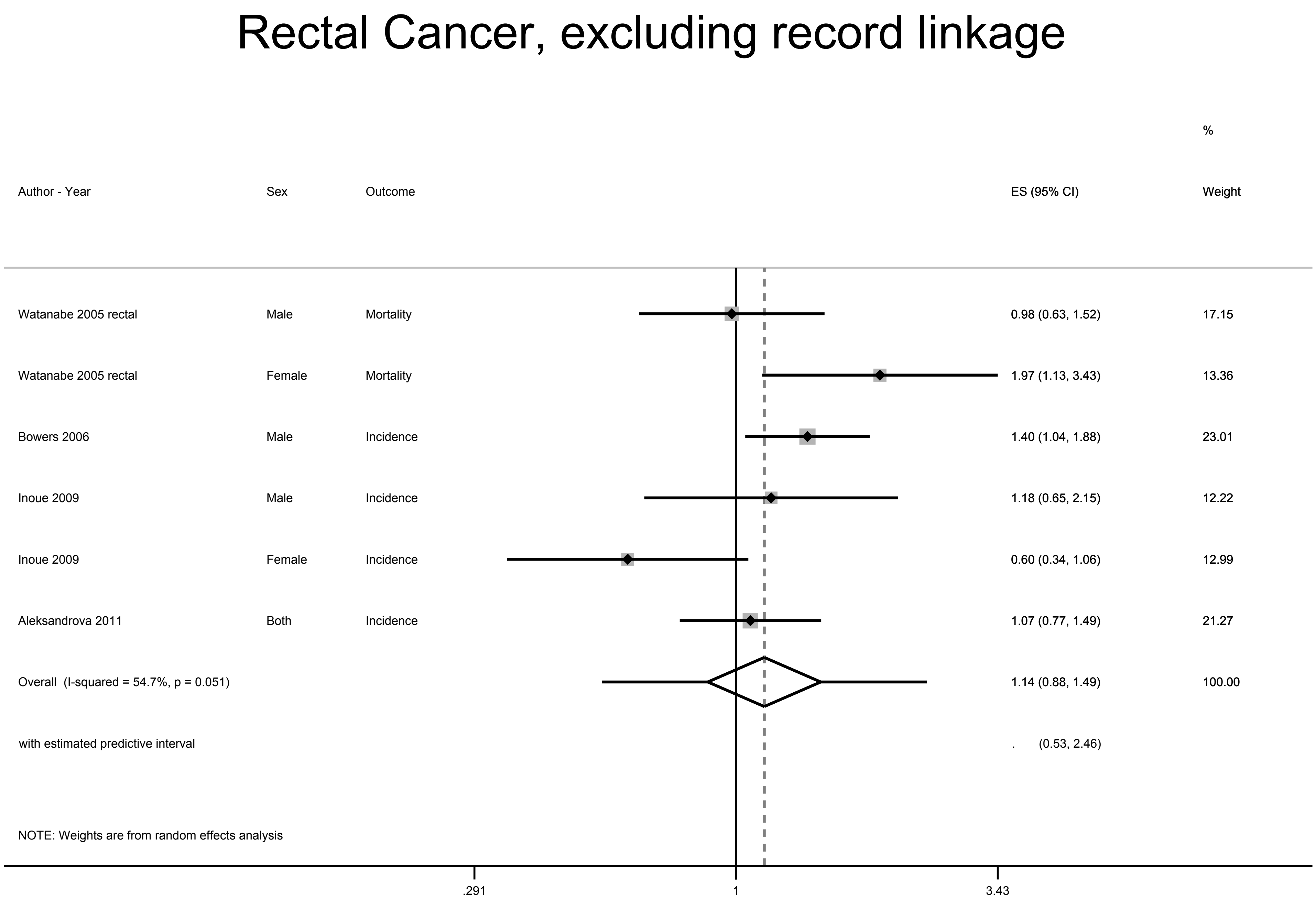

Supplement Figure 9. Meta-analysis of prospective studies for the association between hypertension and rectal cancer risk, excluding record-linkage studies.

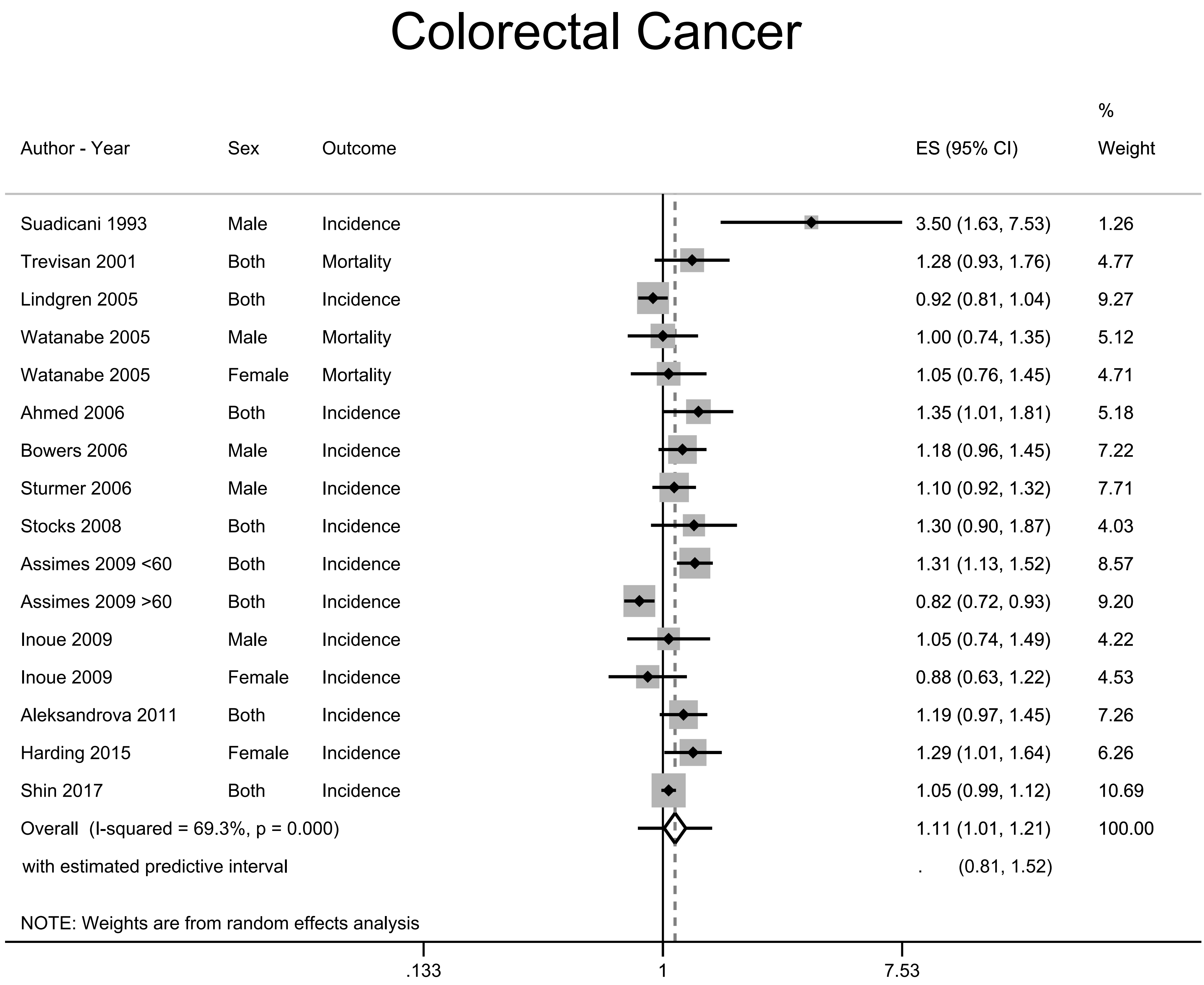

Supplement Figure 10. Meta-analysis of prospective studies for the association between hypertension and colorectal cancer risk.

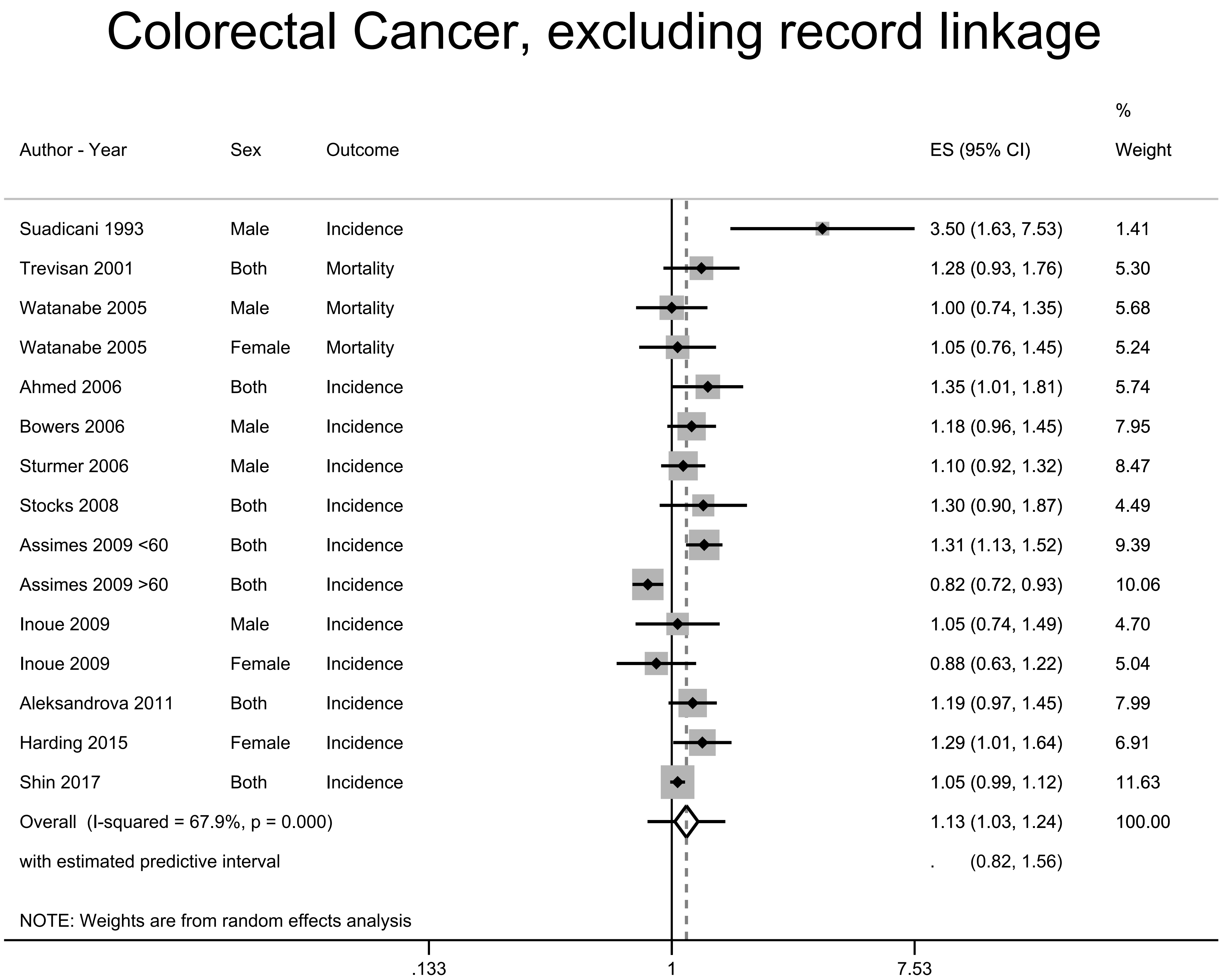

Supplement Figure 11. Meta-analysis of prospective studies for the association between hypertension and colorectal cancer risk, excluding record-linkage studies.

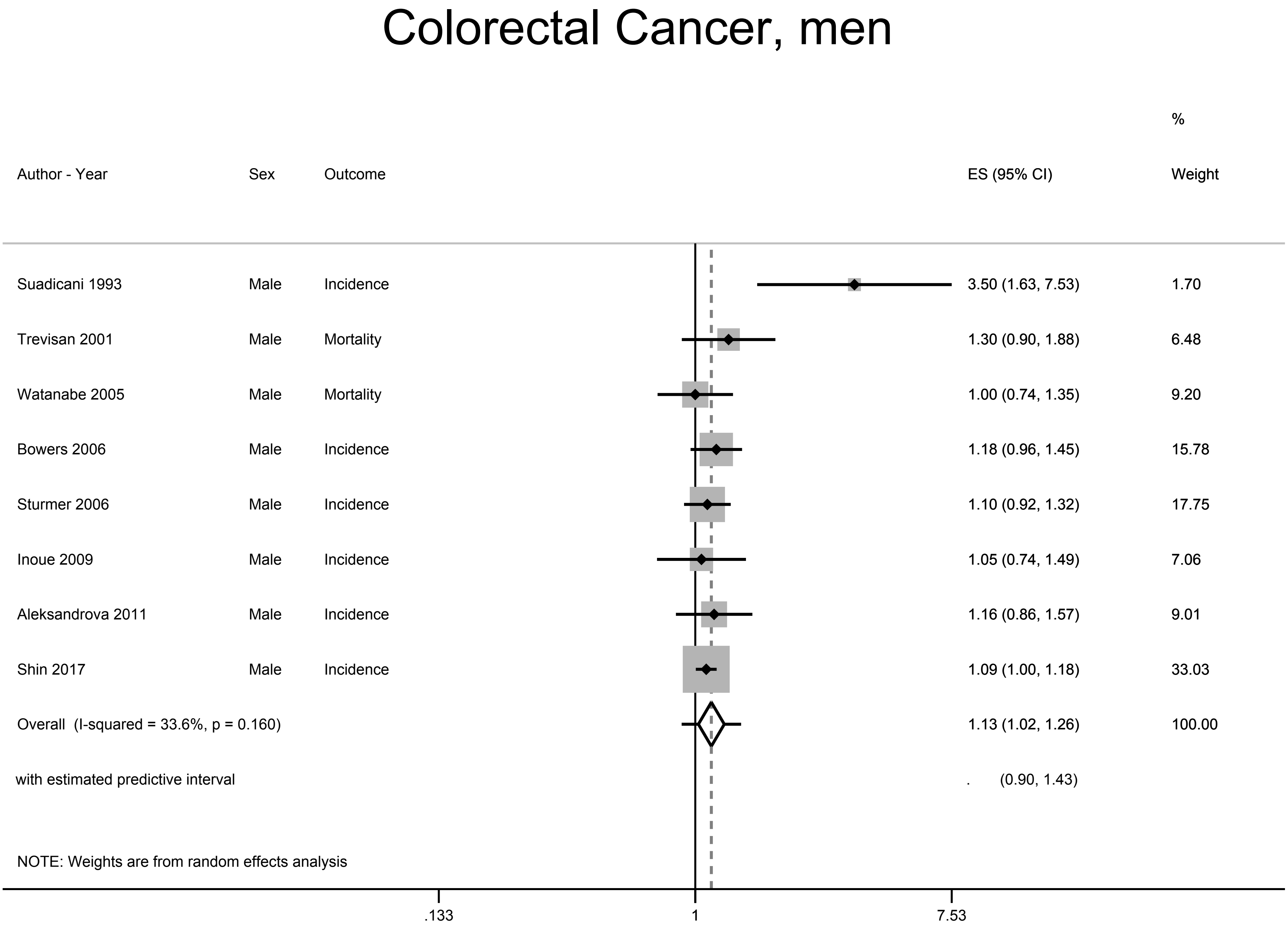

Supplement Figure 12. Meta-analysis of prospective studies for the association between hypertension and colorectal cancer risk, in men.

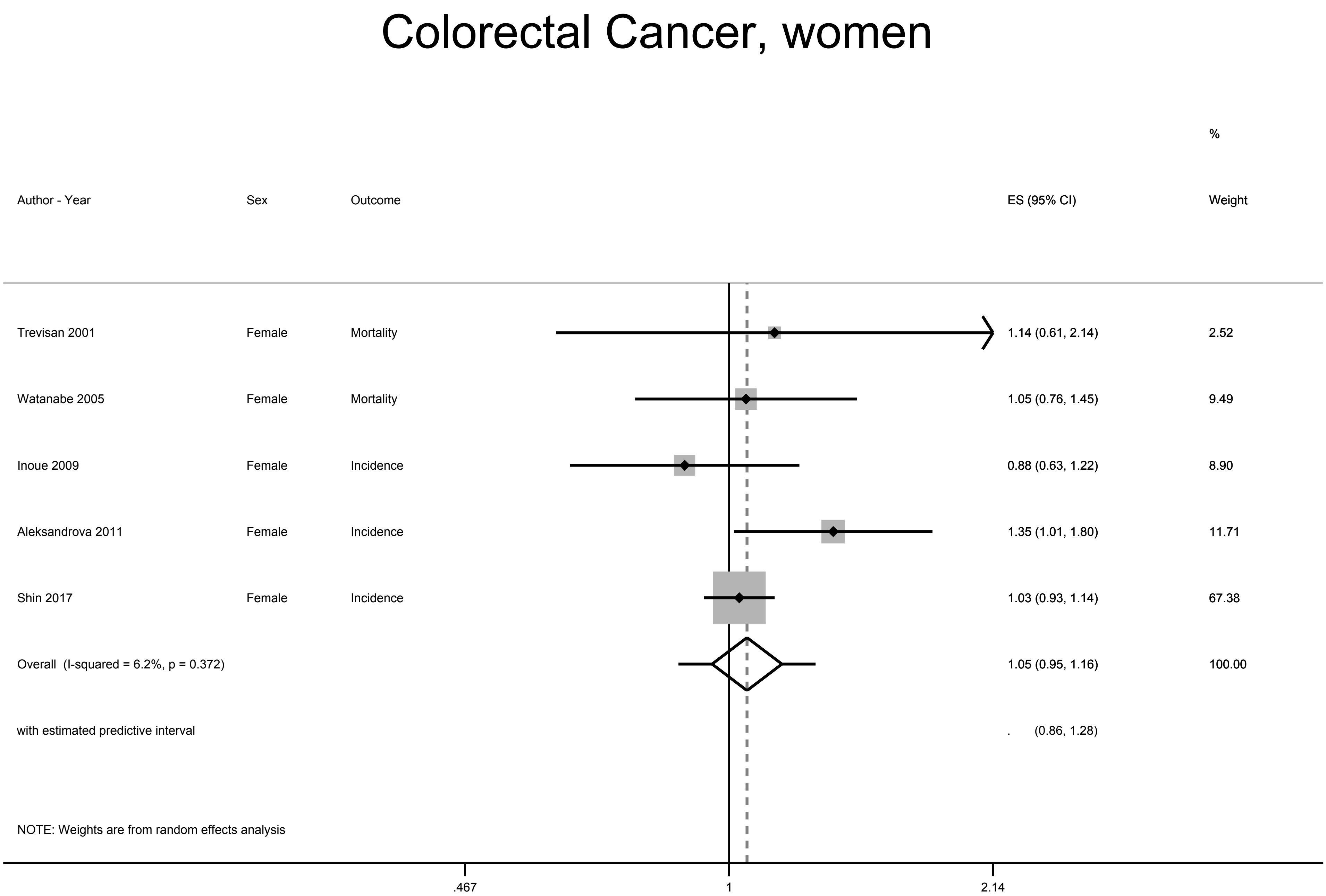

Supplement Figure 13. Meta-analysis of prospective studies for the association between hypertension and colorectal cancer risk, in women.

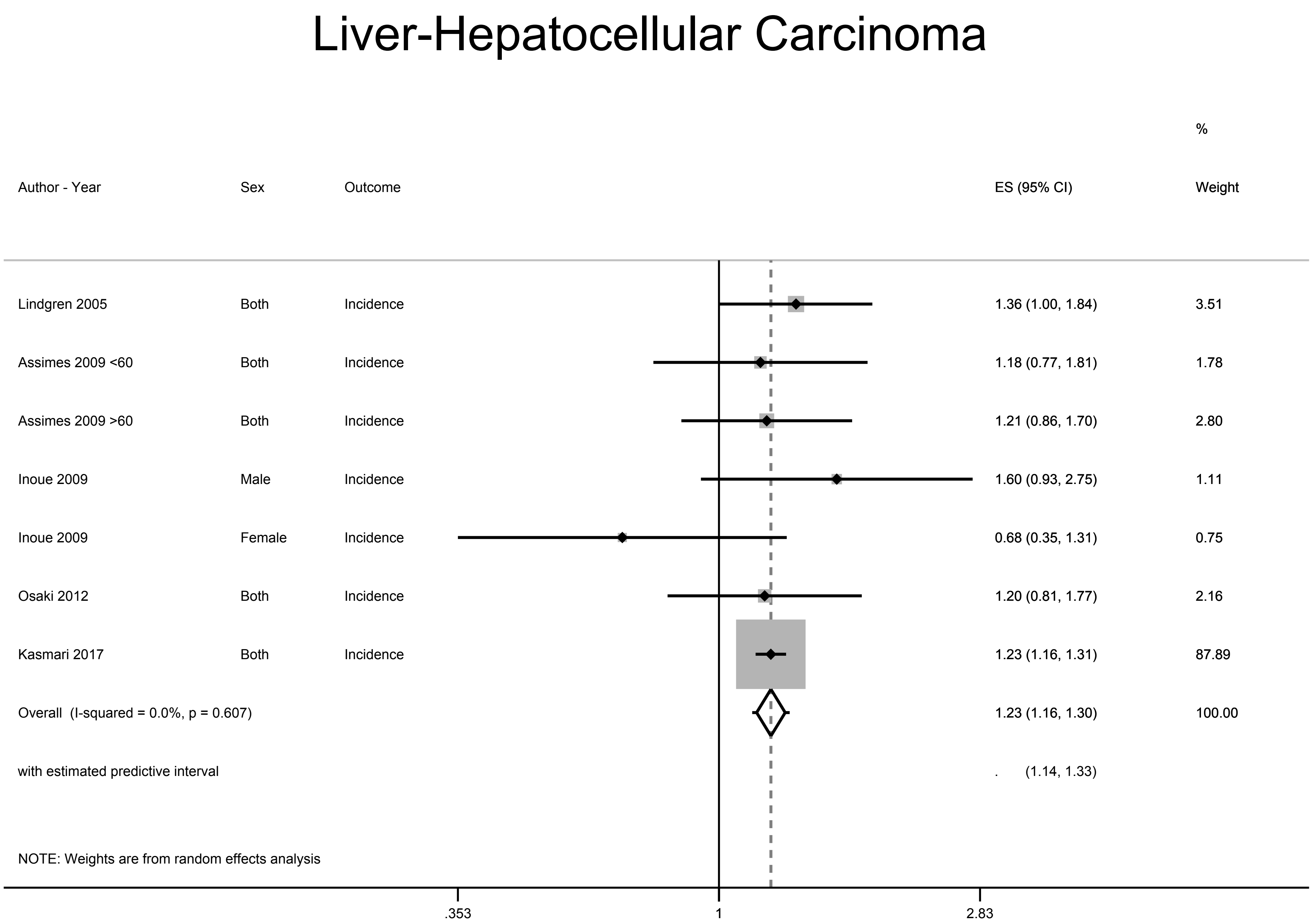

Supplement Figure 14. Meta-analysis of prospective studies for the association between hypertension and liver/hepatocellular cancer risk.

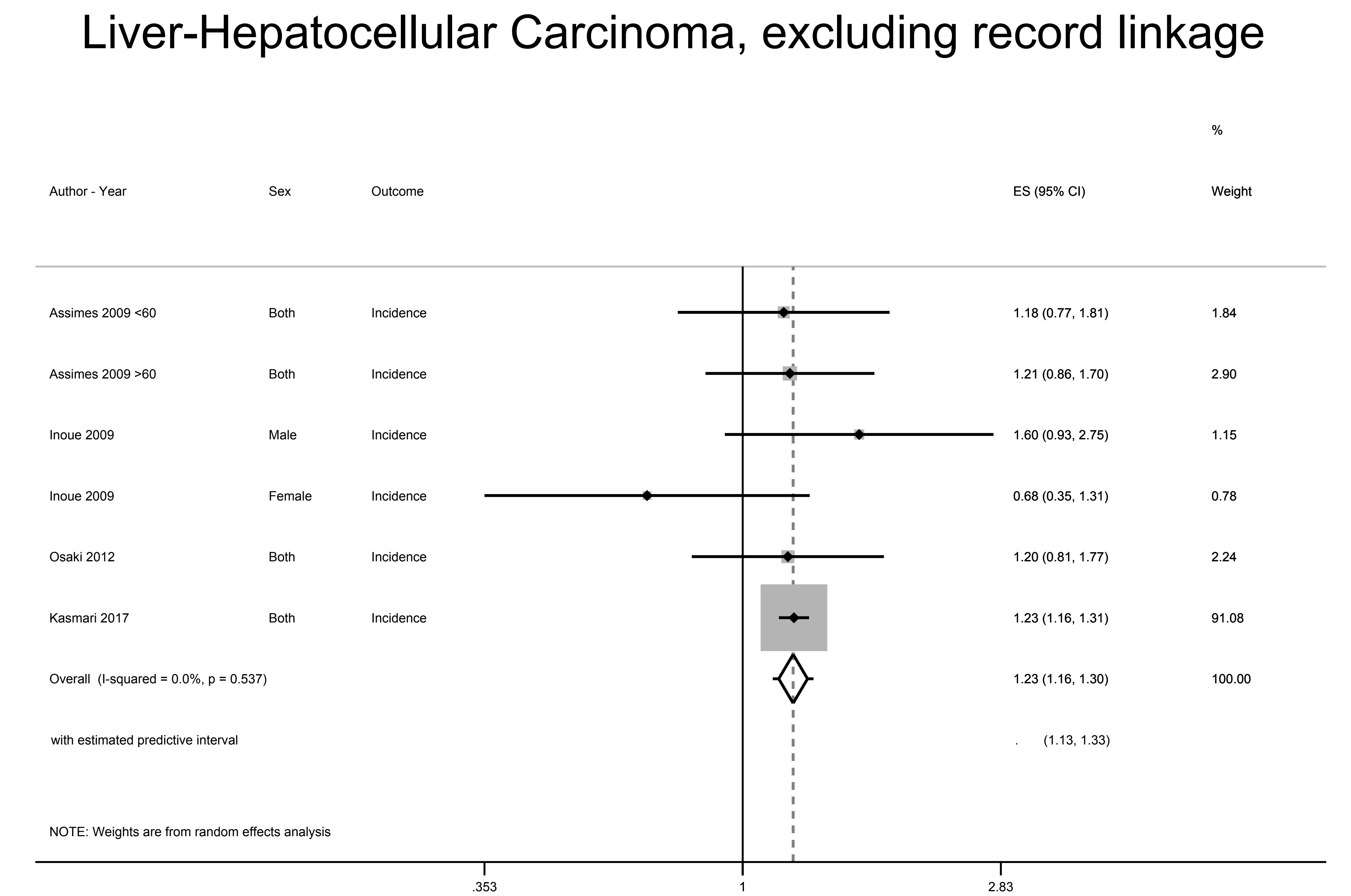

Supplement Figure 15. Meta-analysis of prospective studies for the association between hypertension and liver/hepatocellular cancer risk, excluding record-linkage studies.

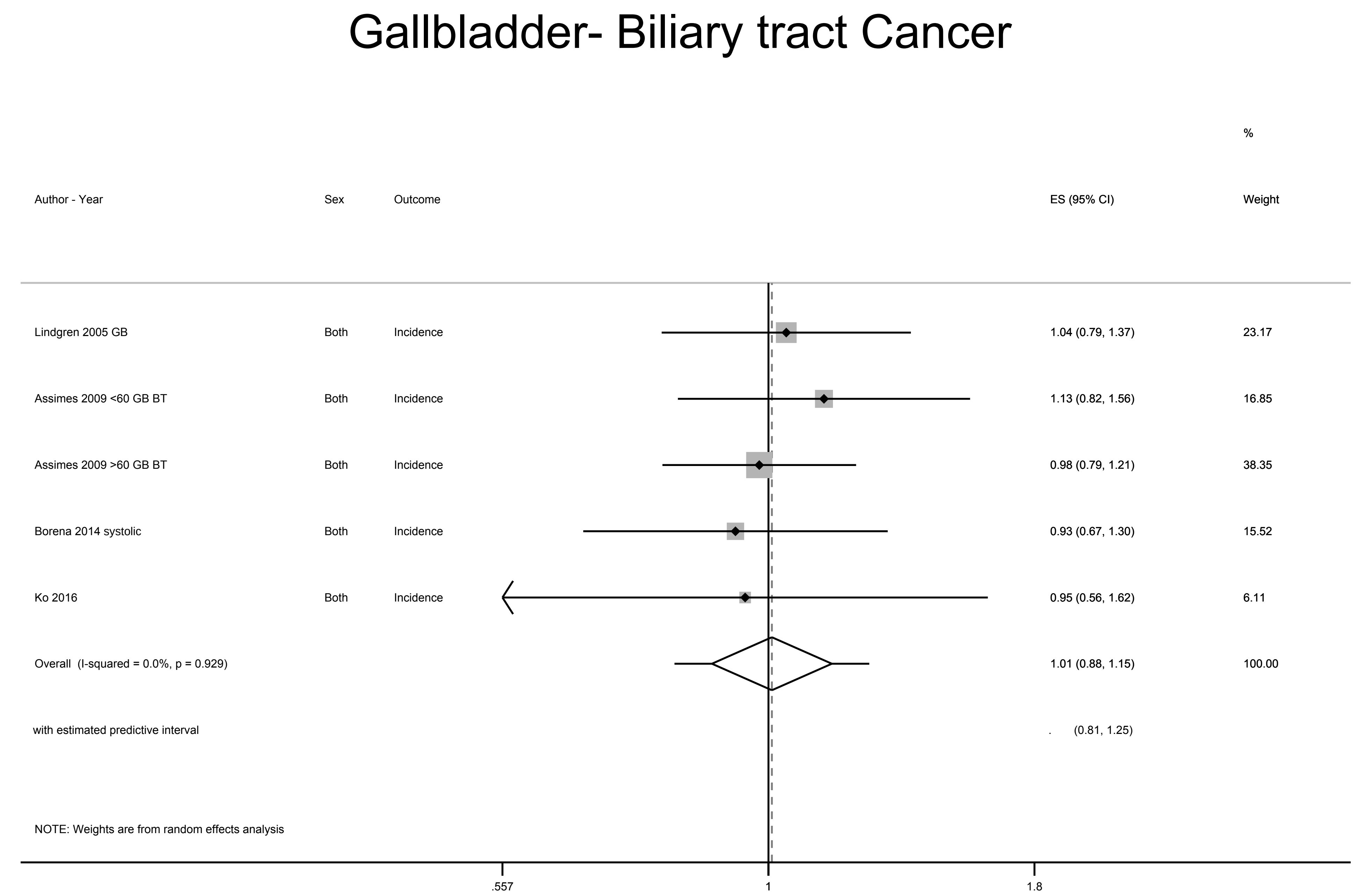

Supplement Figure 16. Meta-analysis of prospective studies for the association between hypertension and gall bladder/biliary tract cancer risk.

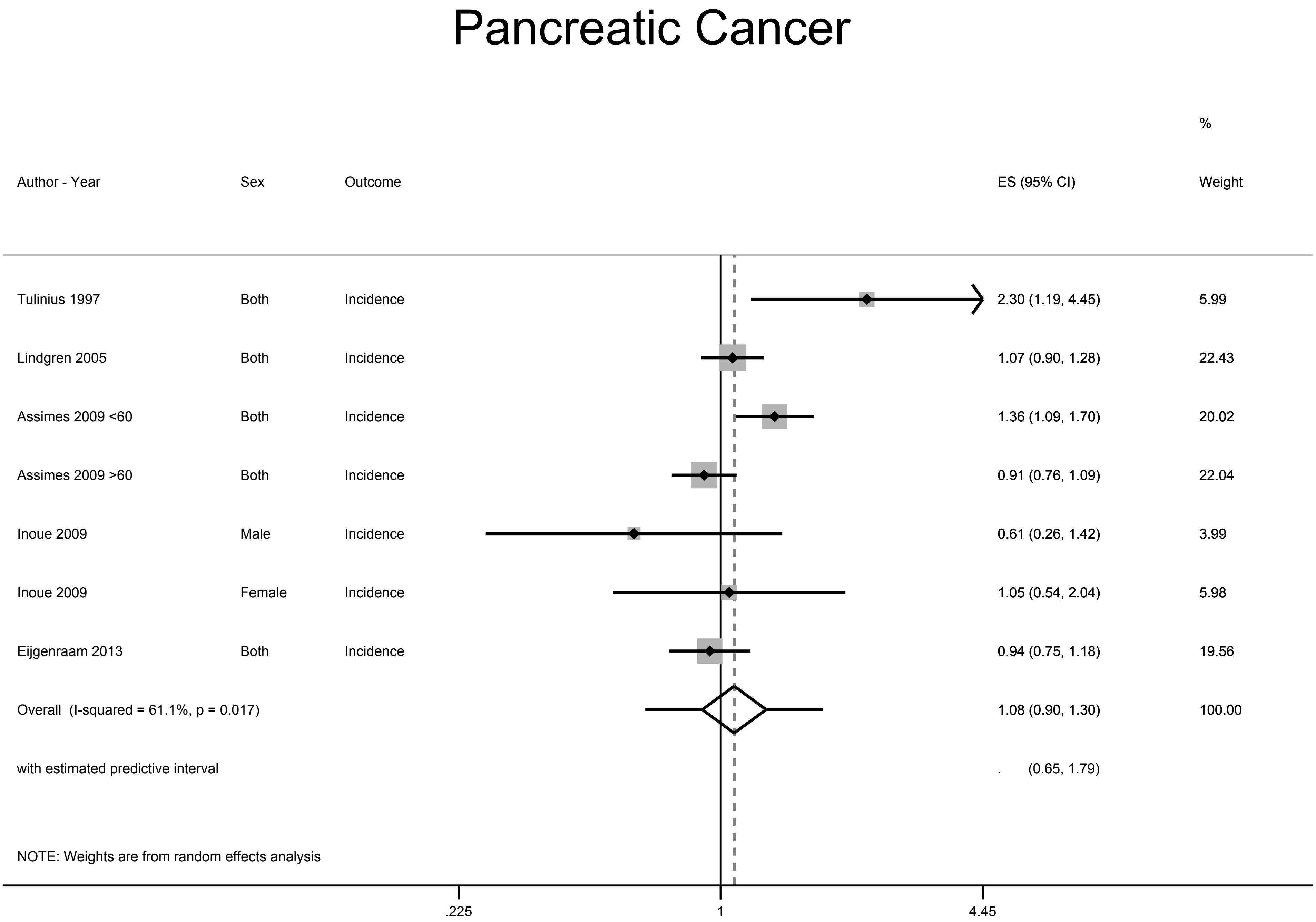

Supplement Figure 17. Meta-analysis of prospective studies for the association between hypertension and pancreatic cancer risk.

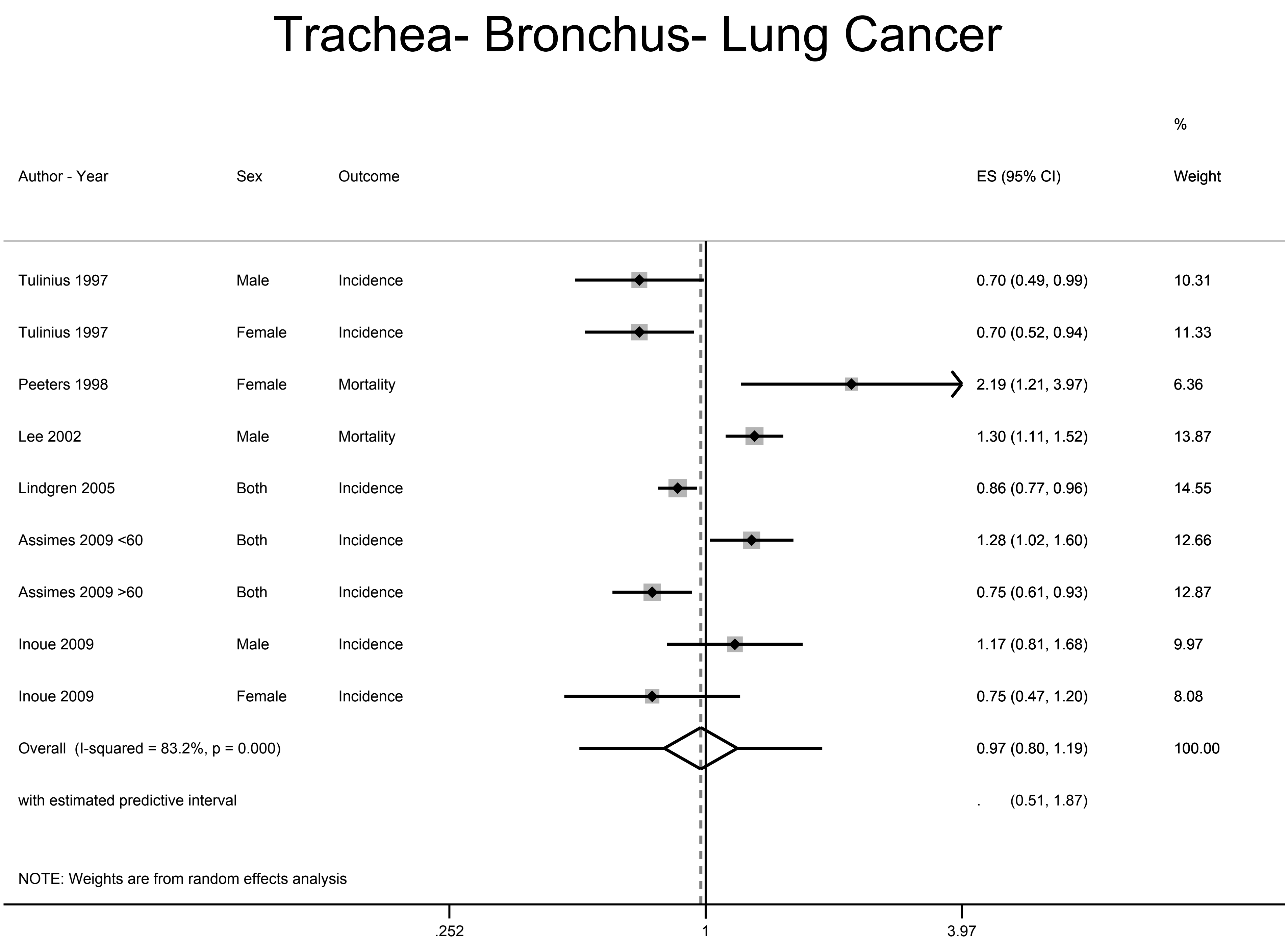

Supplement Figure 18. Meta-analysis of prospective studies for the association between hypertension and trachea/bronchus/lung cancer risk.

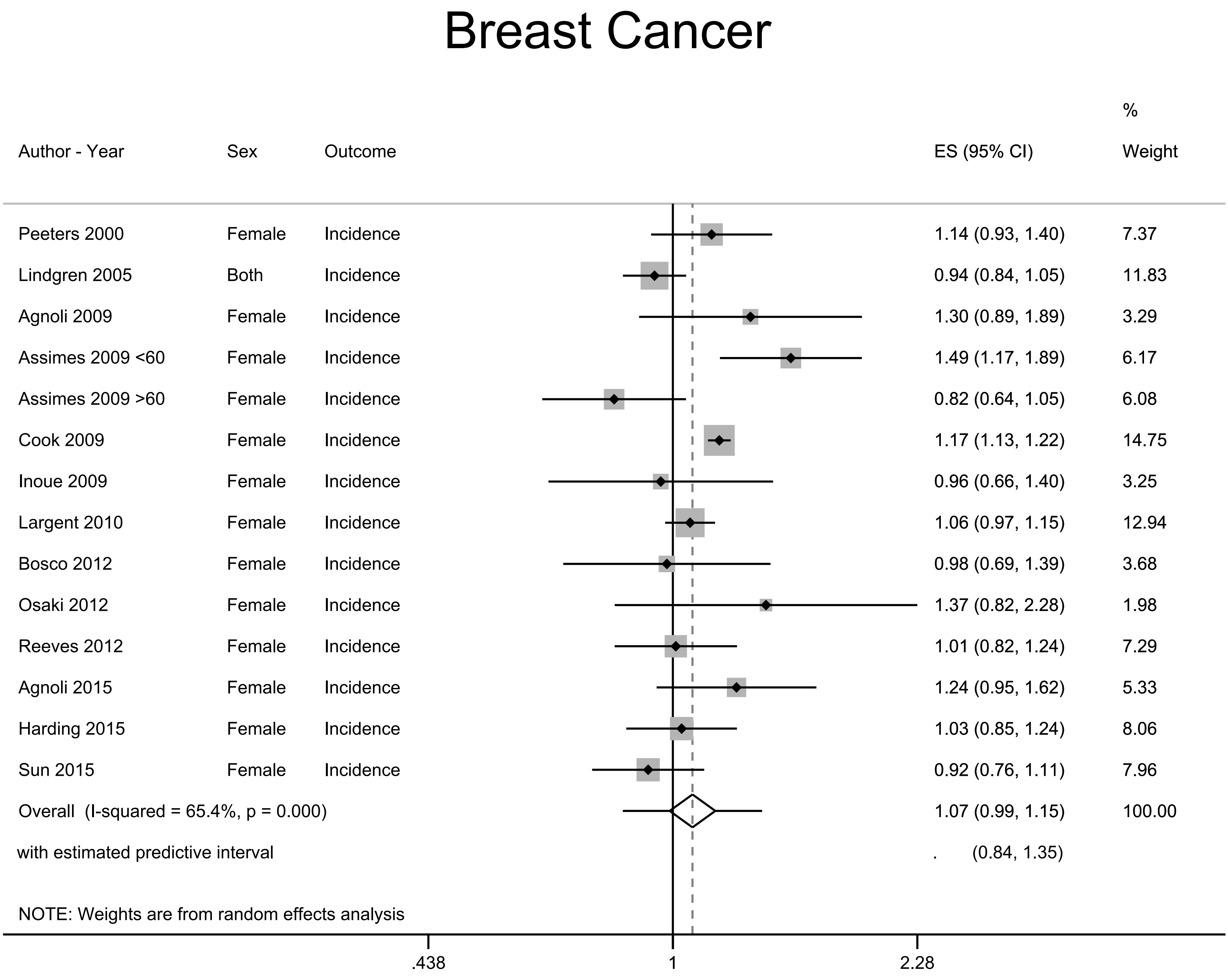

Supplement Figure 19. Meta-analysis of prospective studies for the association between hypertension and breast cancer risk.

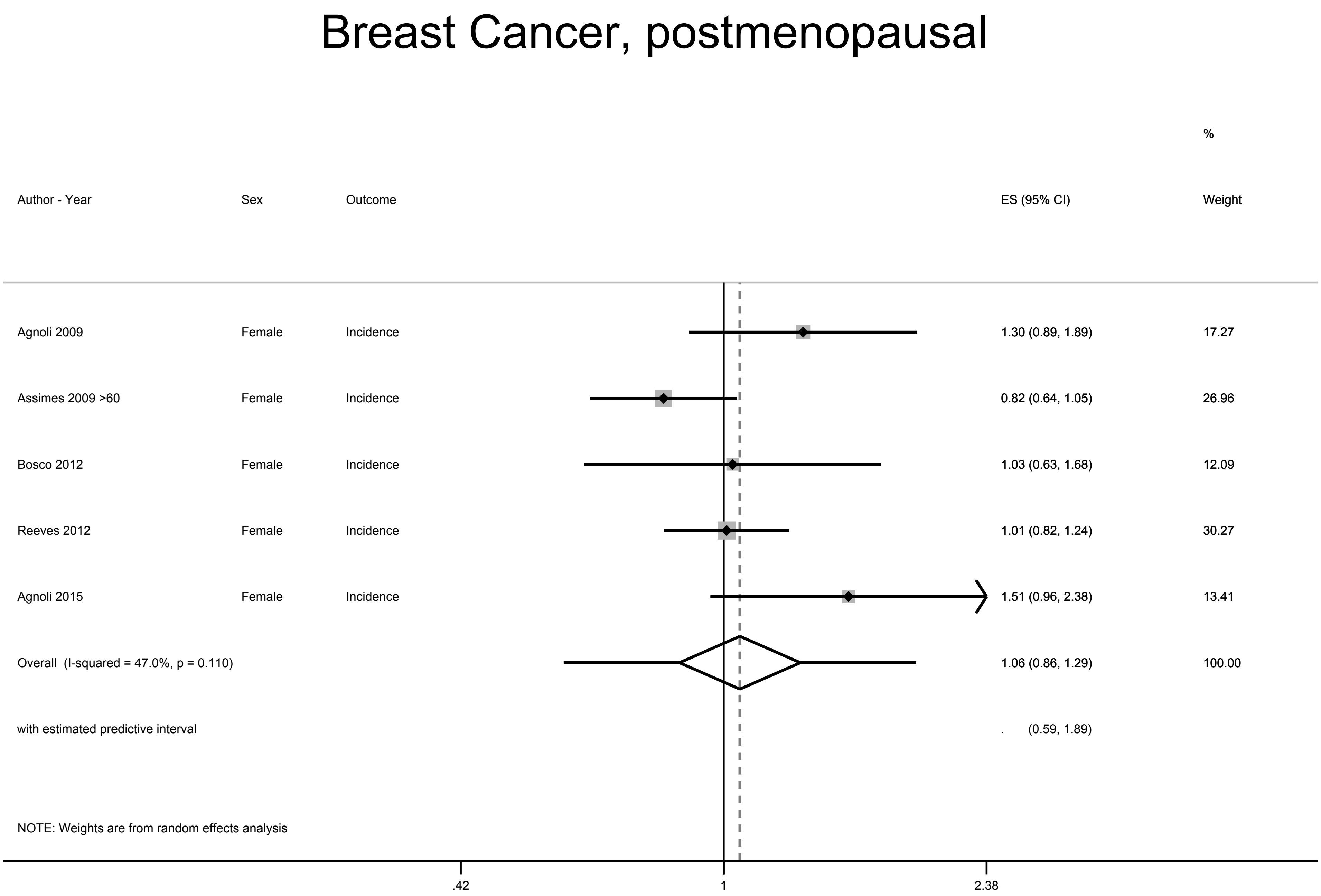

Supplement Figure 20. Meta-analysis of prospective studies for the association between hypertension and breast cancer risk, in postmenopausal women.

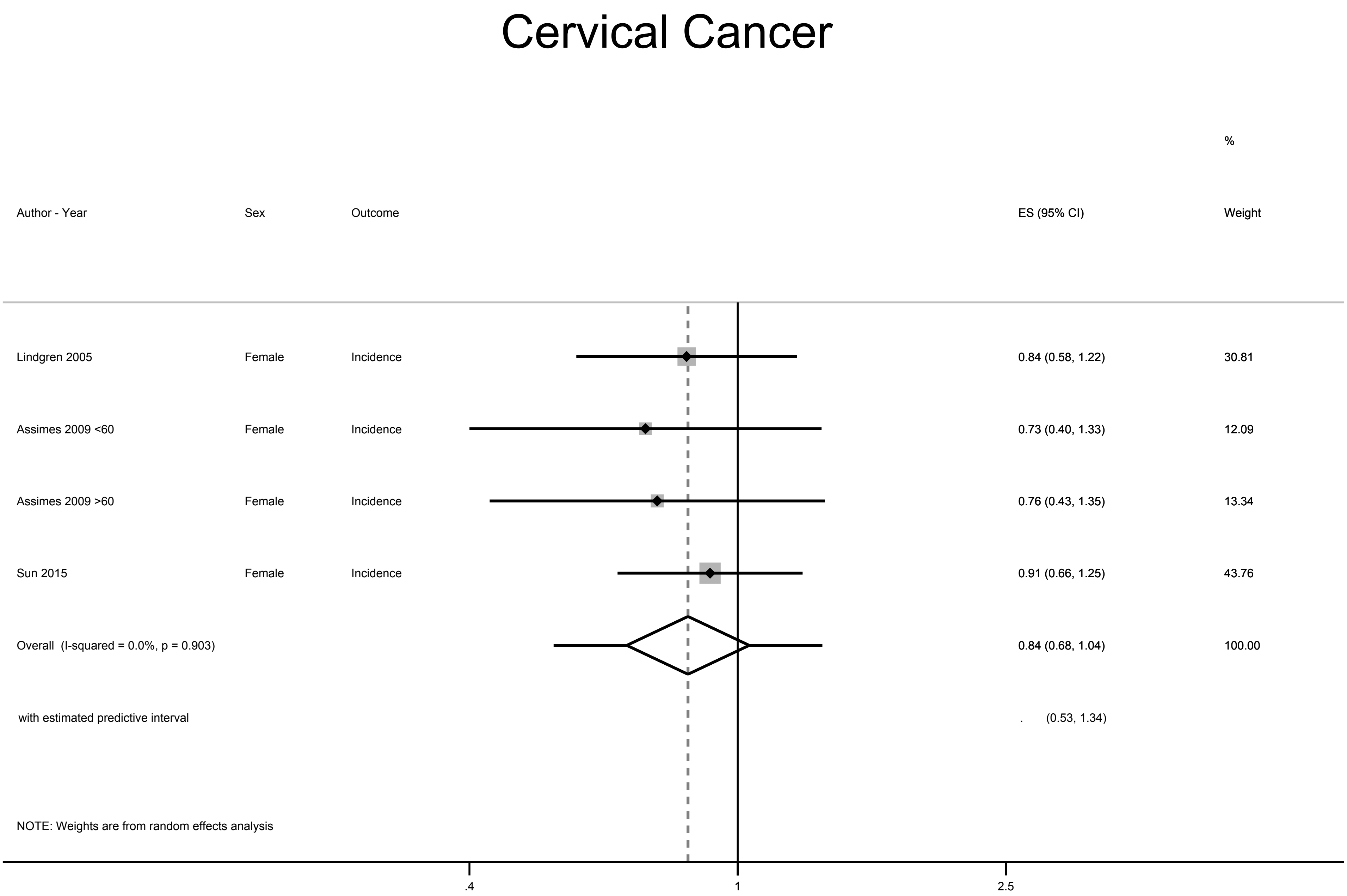

Supplement Figure 21. Meta-analysis of prospective studies for the association between hypertension and cervical cancer risk.

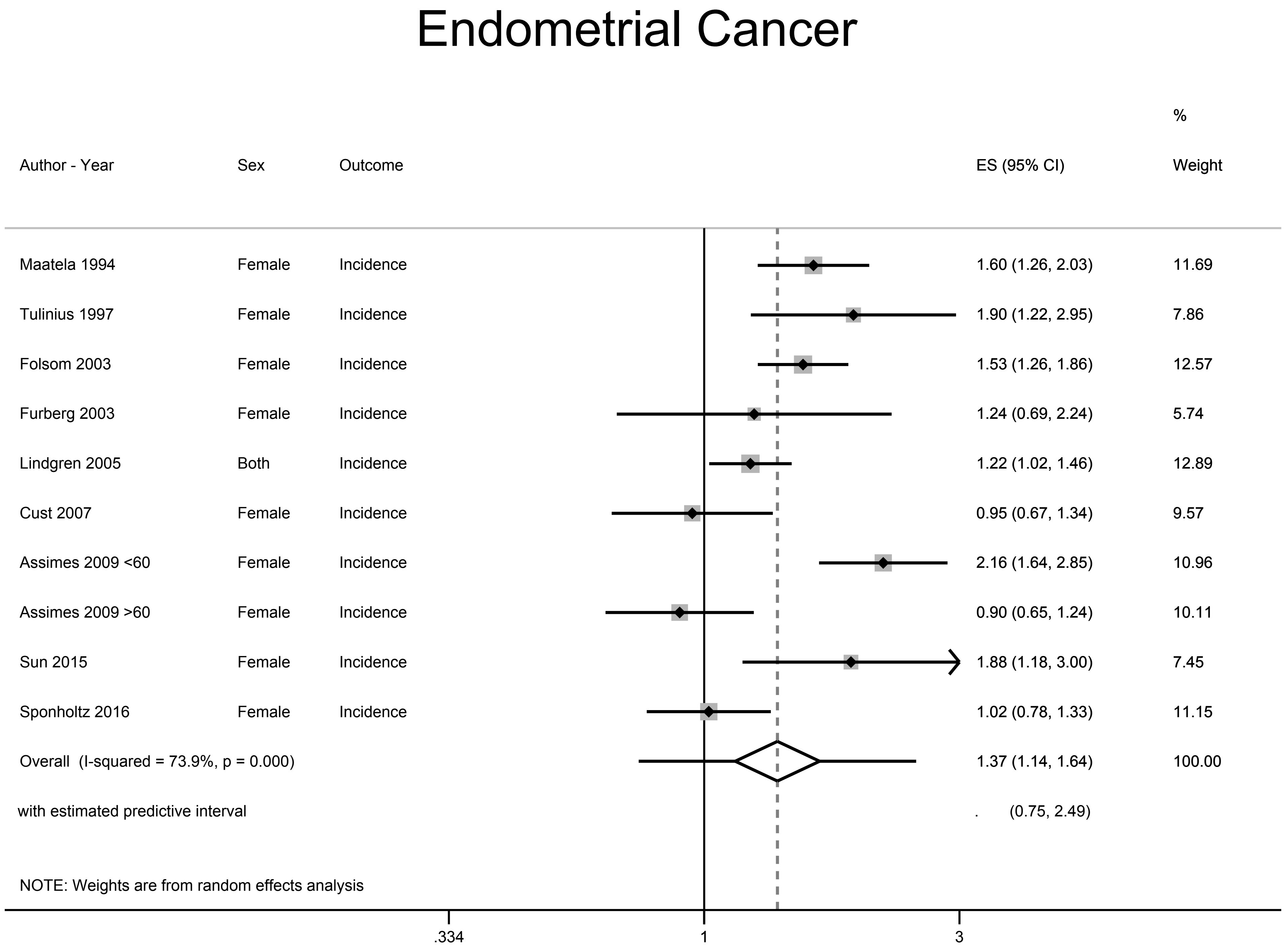

Supplement Figure 22. Meta-analysis of prospective studies for the association between hypertension and endometrial cancer risk.

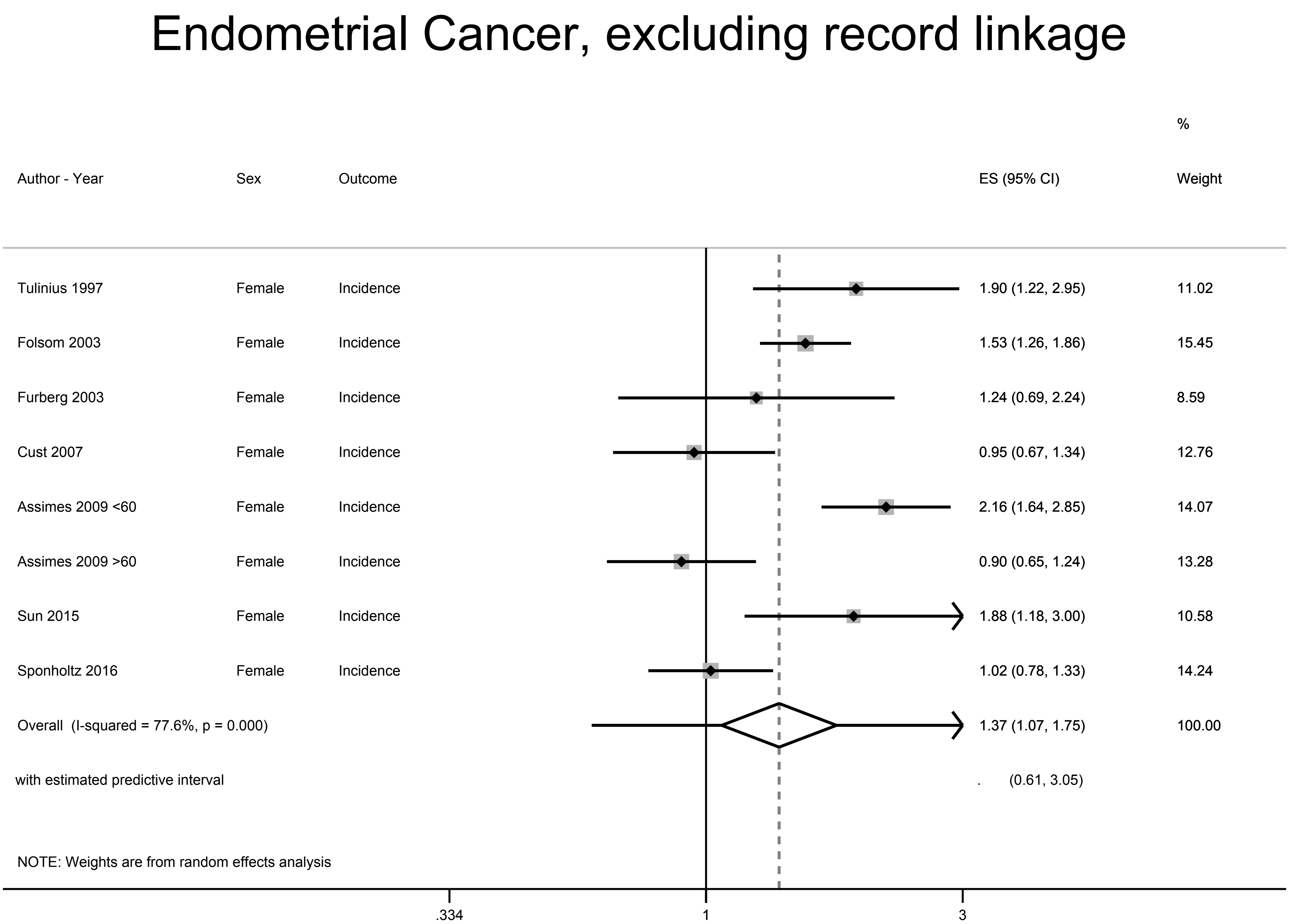

Supplement Figure 23. Meta-analysis of prospective studies for the association between hypertension and endometrial cancer risk, excluding record-linkage studies.

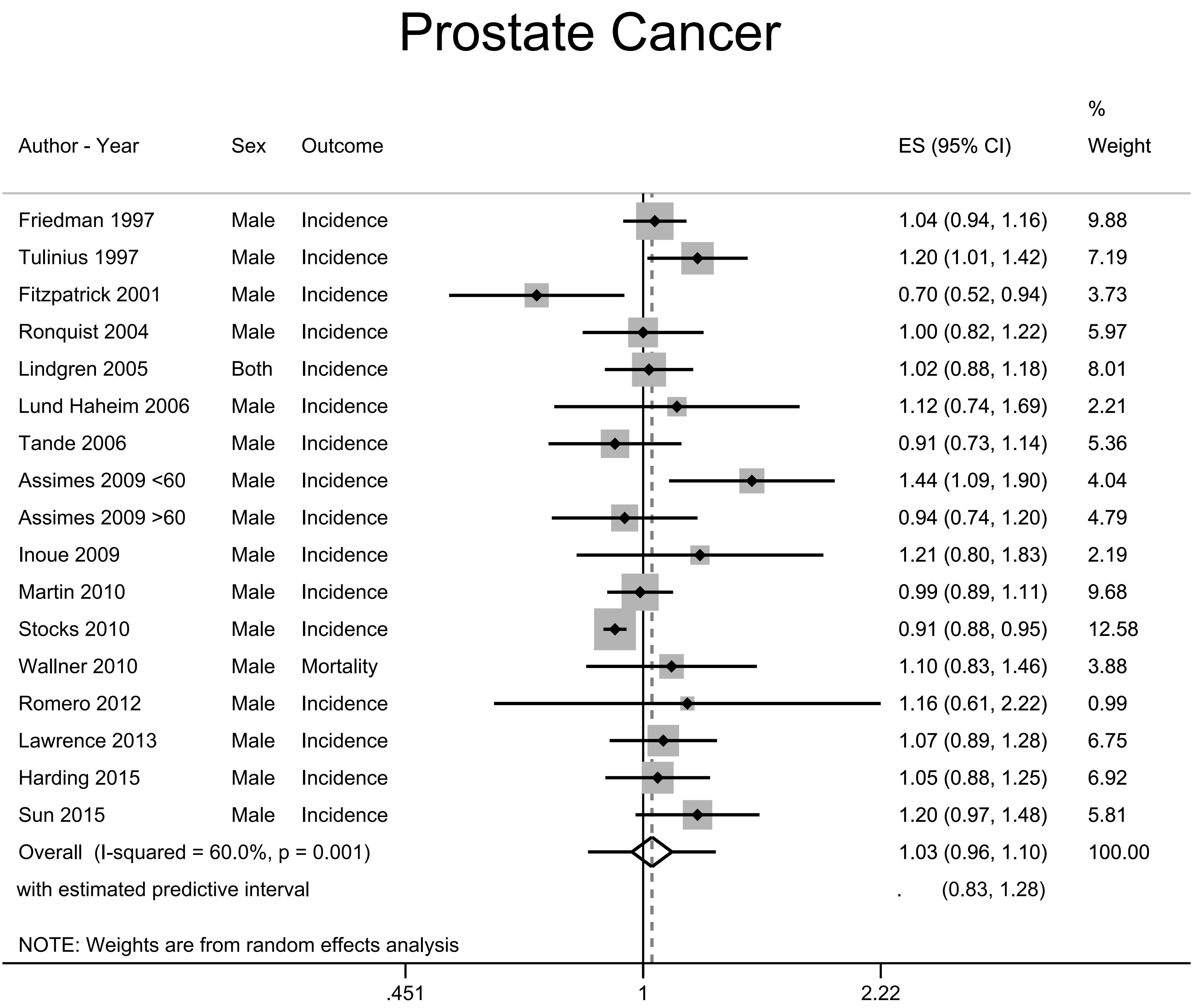

Supplement Figure 24. Meta-analysis of prospective cancer for the association between hypertension and prostate cancer risk in prospective studies.

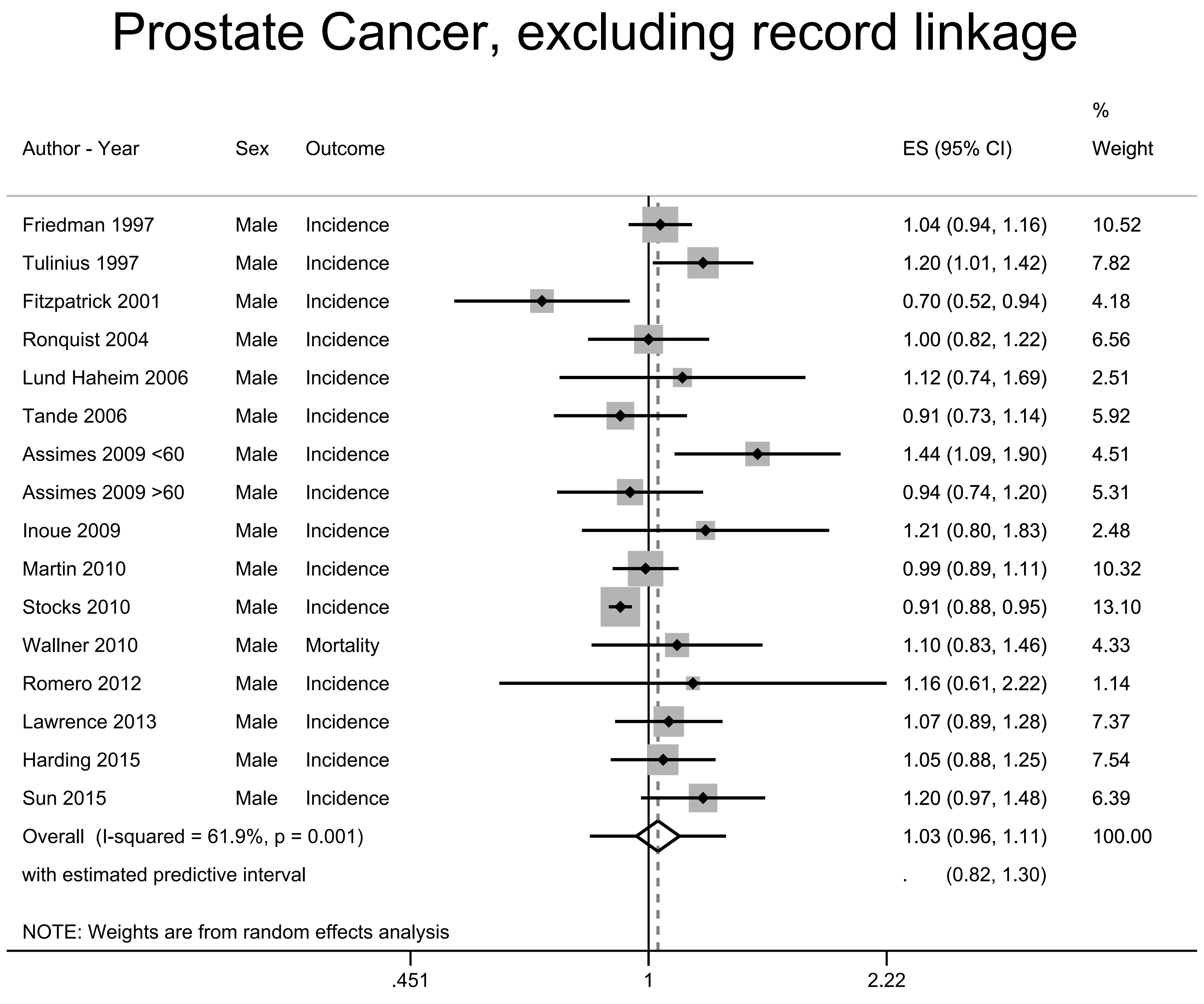

Supplement Figure 25. Meta-analysis of prospective studies for the association between hypertension and prostate cancer risk, excluding record-linkage studies.

Kidney-Renal Cancer

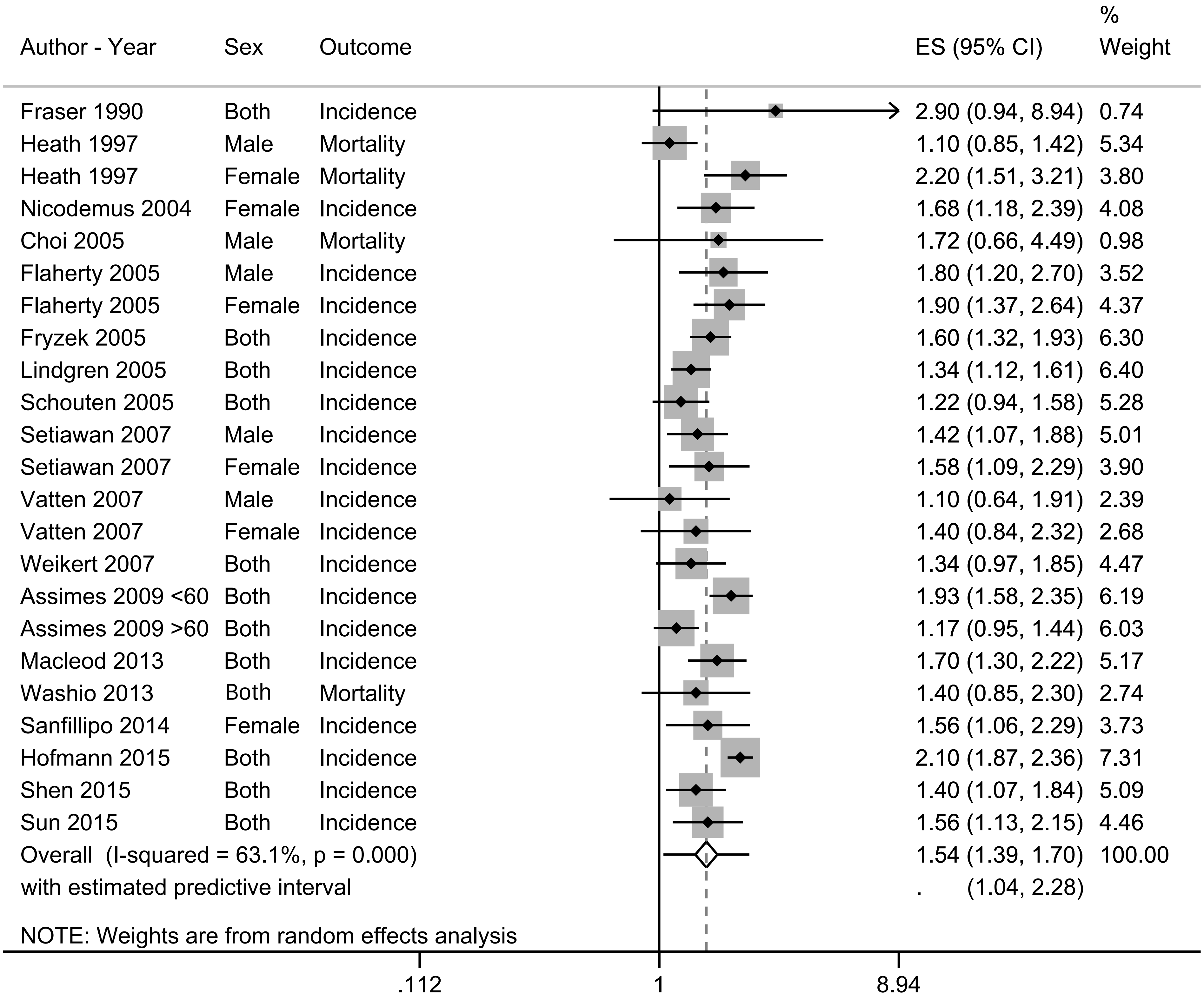

Supplement Figure 26. Meta-analysis of prospective studies for the association between hypertension and kidney/renal cancer risk.

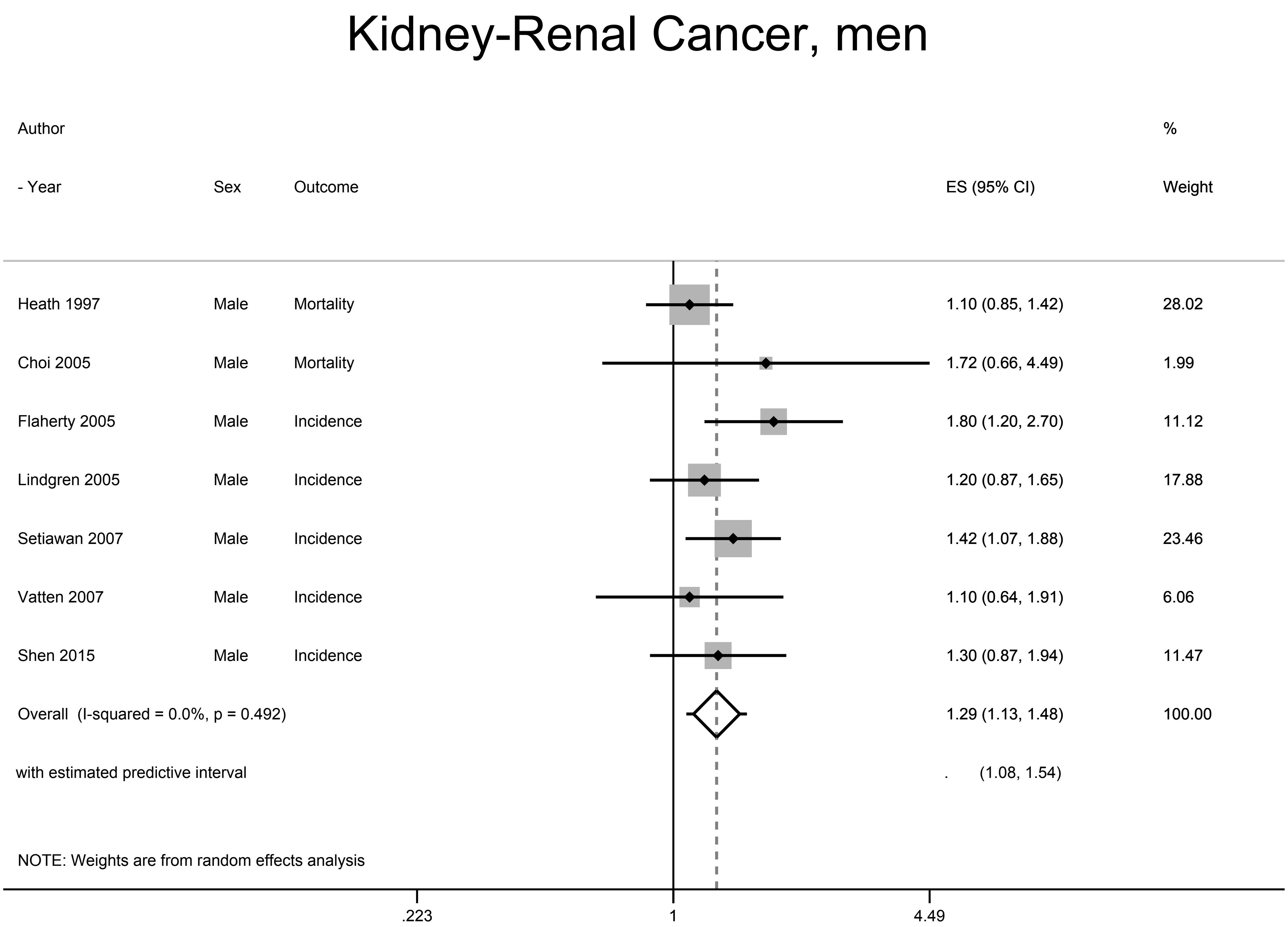

Supplement Figure 27. Meta-analysis of prospective studies for the association between hypertension and kidney/renal cancer risk, in men.

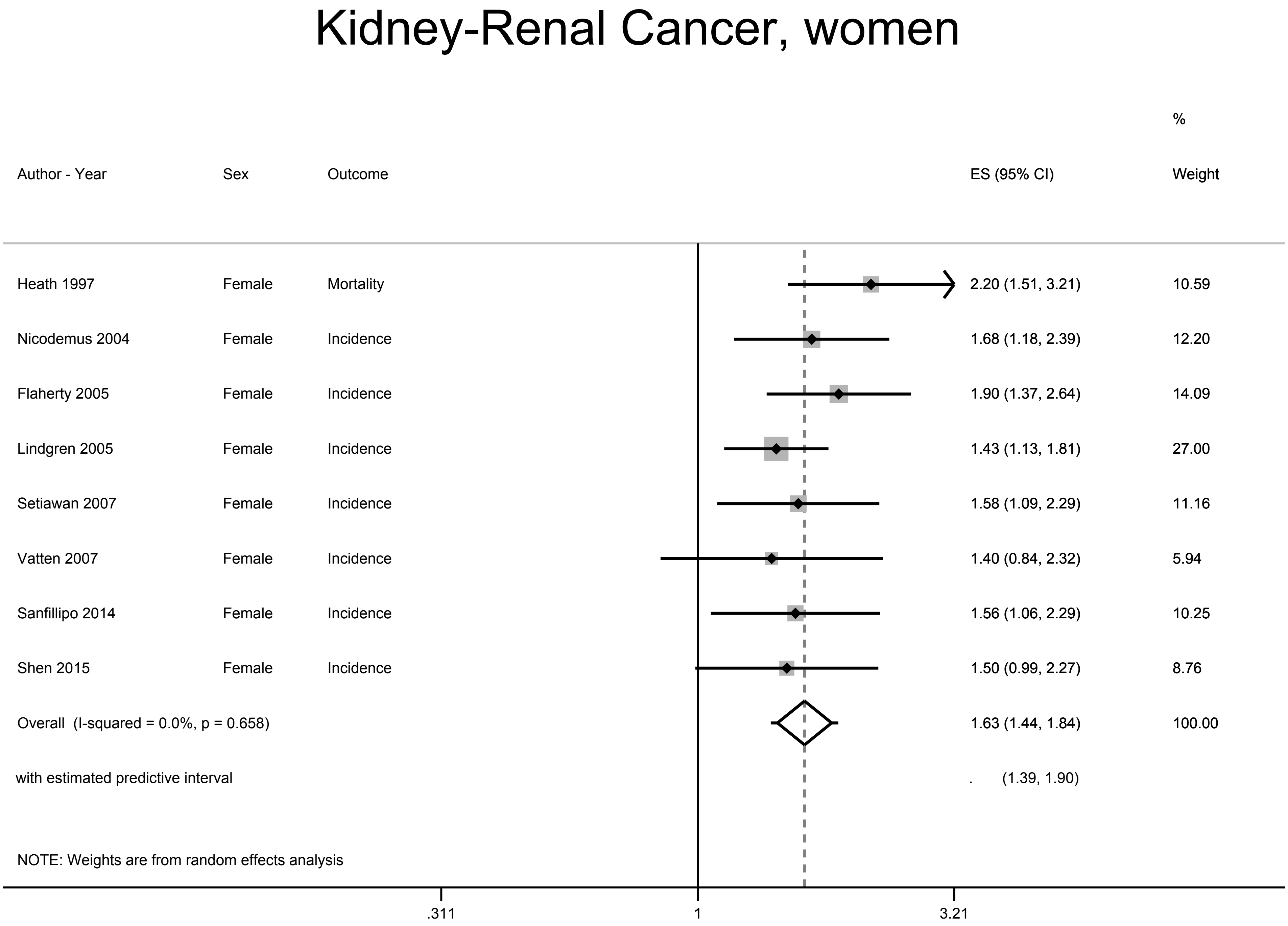

Supplement Figure 28. Meta-analysis of prospective studies for the association between hypertension and kidney/renal cancer risk, in women.

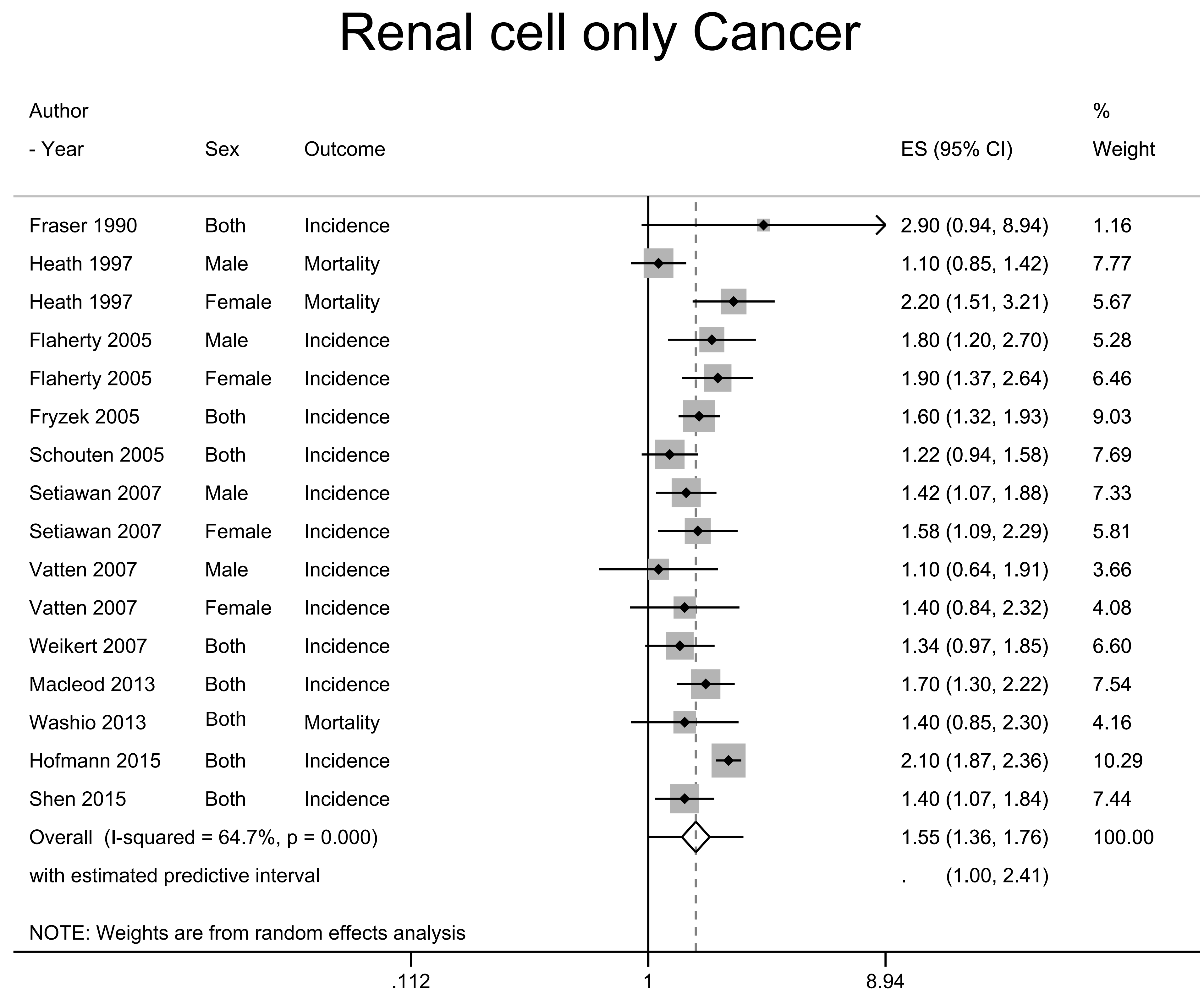

Supplement Figure 29. Meta-analysis of prospective studies for the association between hypertension and renal cell specific cancer risk.

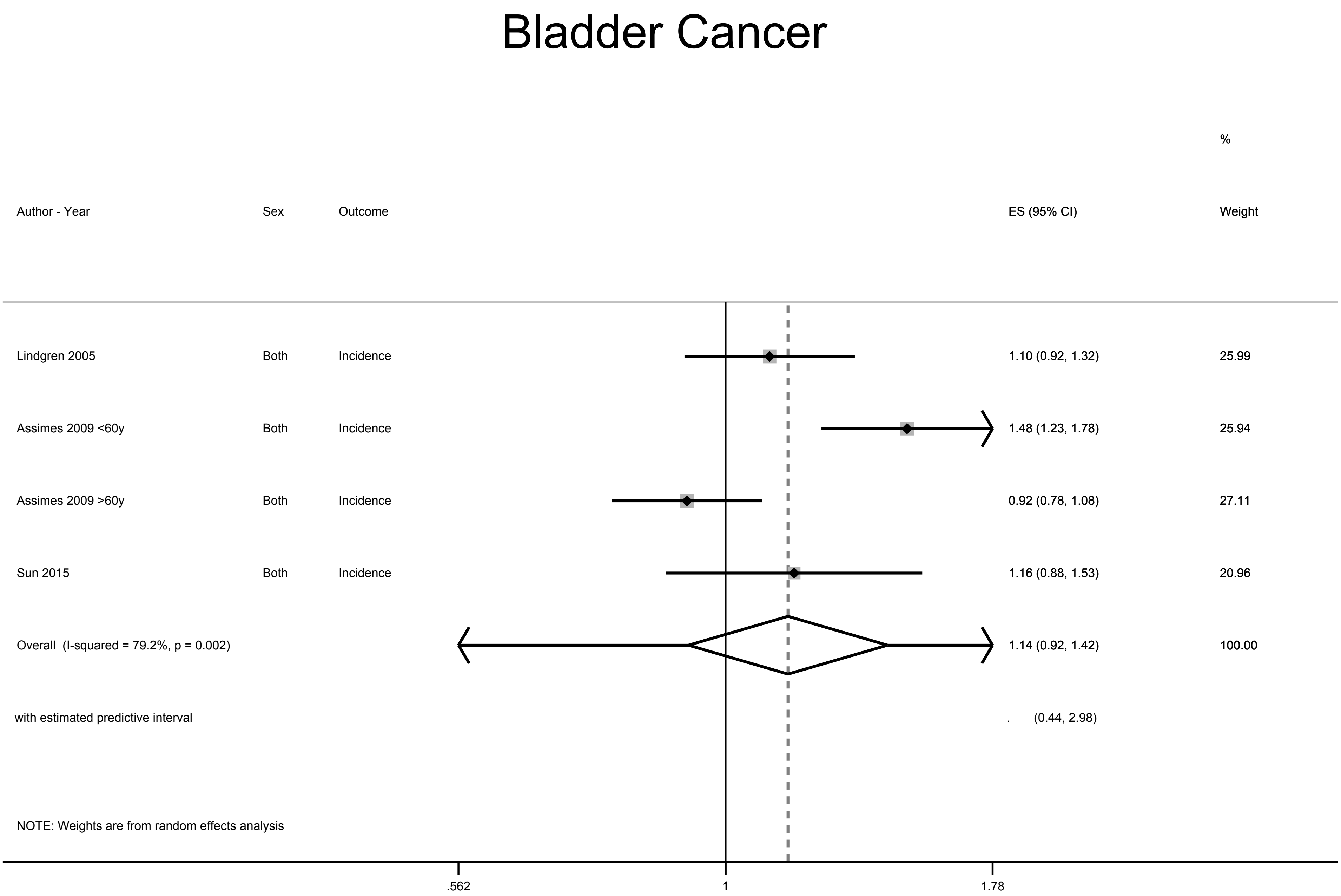

Supplement Figure 30. Meta-analysis of prospective studies for the association between hypertension and bladder cancer risk.

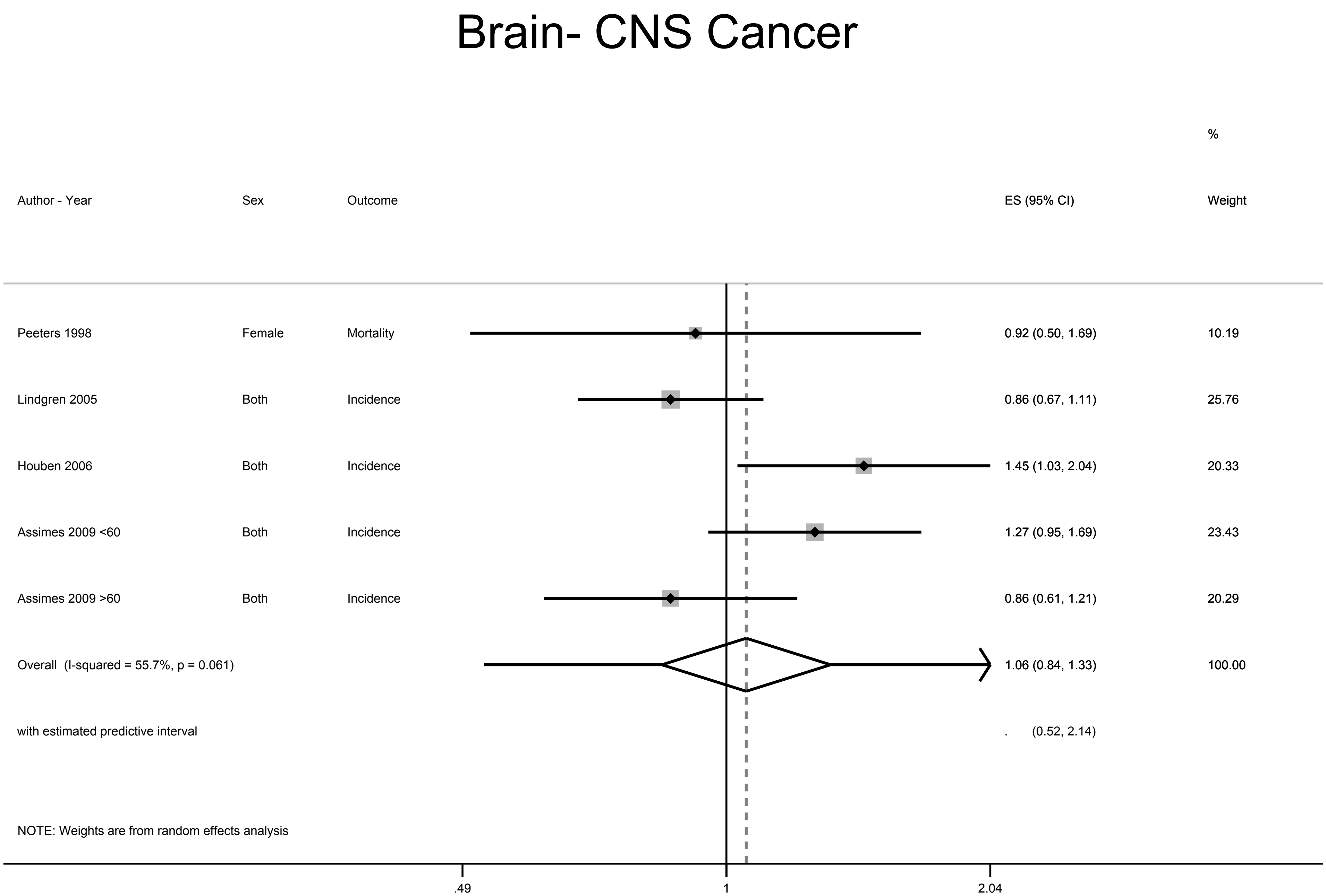

Supplement Figure 31. Meta-analysis of prospective studies for the association between hypertension and brain/CNS cancer risk. Abbreviations: CNS, central nervous system.

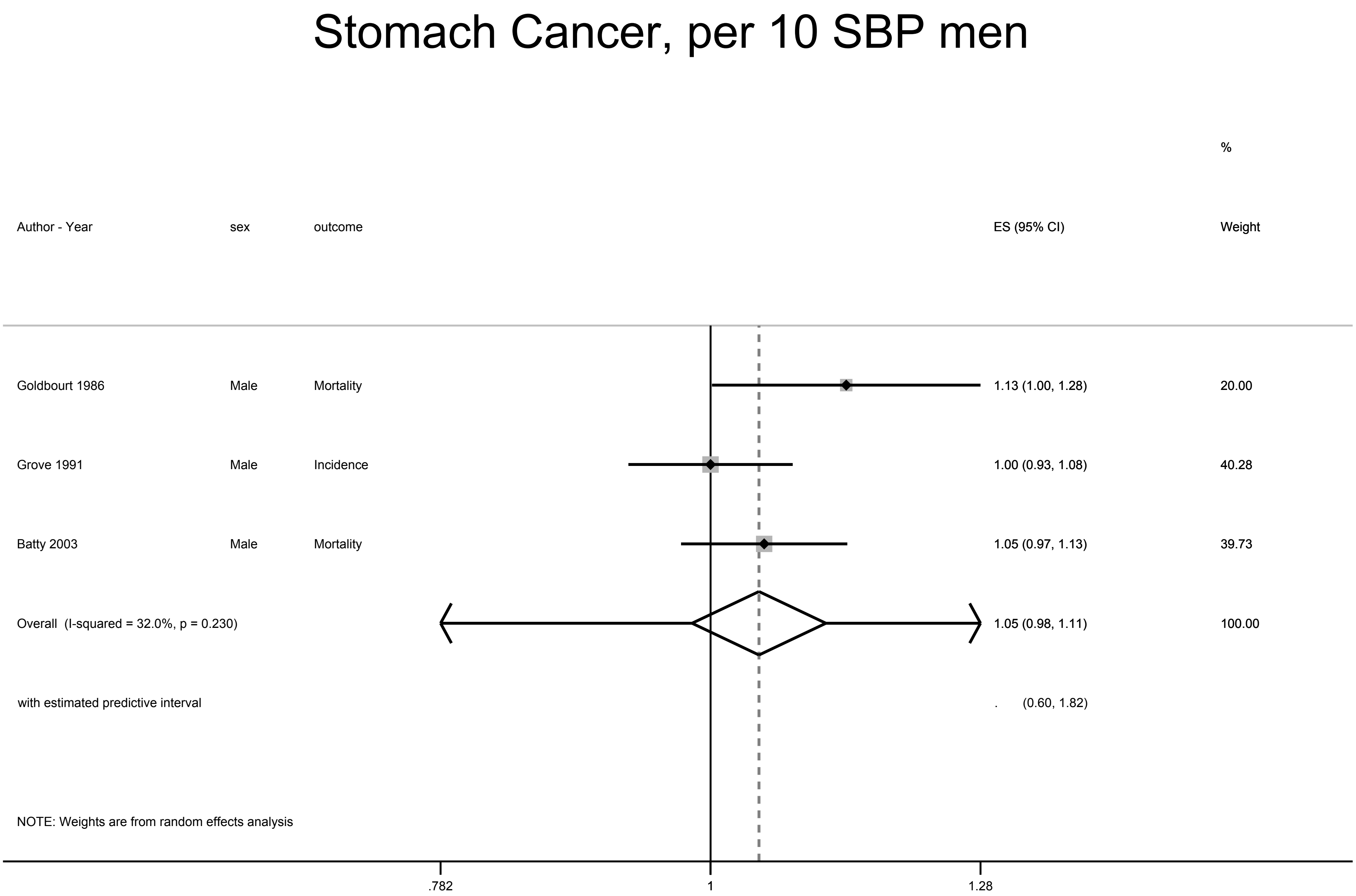

Supplement Figure 32. Meta-analysis of prospective studies for the association between stomach cancer risk and 10mmHg increase in systolic blood pressure. Abbreviations: SBP, systolic blood pressure.

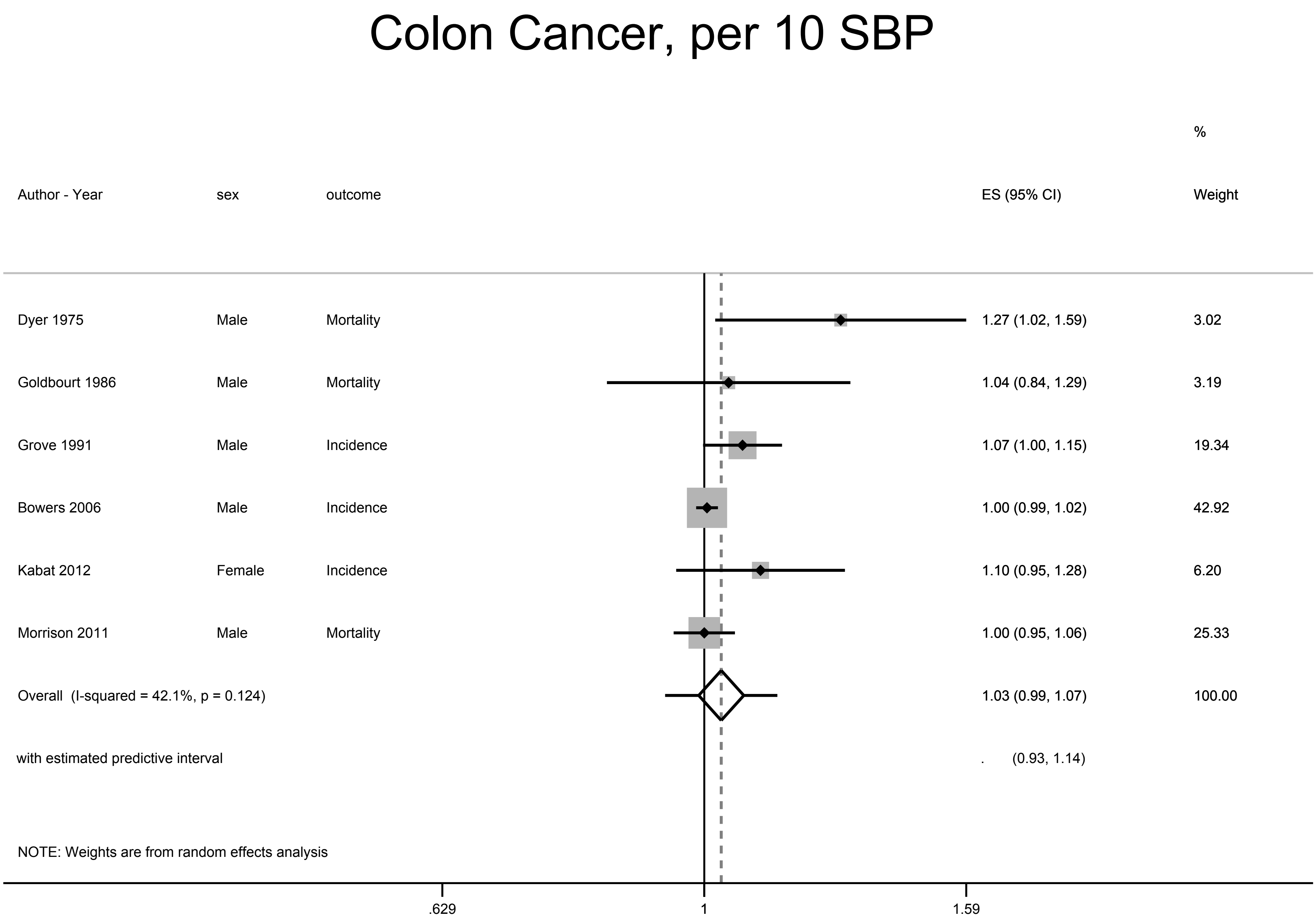

Supplement Figure 33. Meta-analysis of prospective studies for the association between colon cancer risk and 10mmHg increase in systolic blood pressure. Abbreviations: SBP, systolic blood pressure.

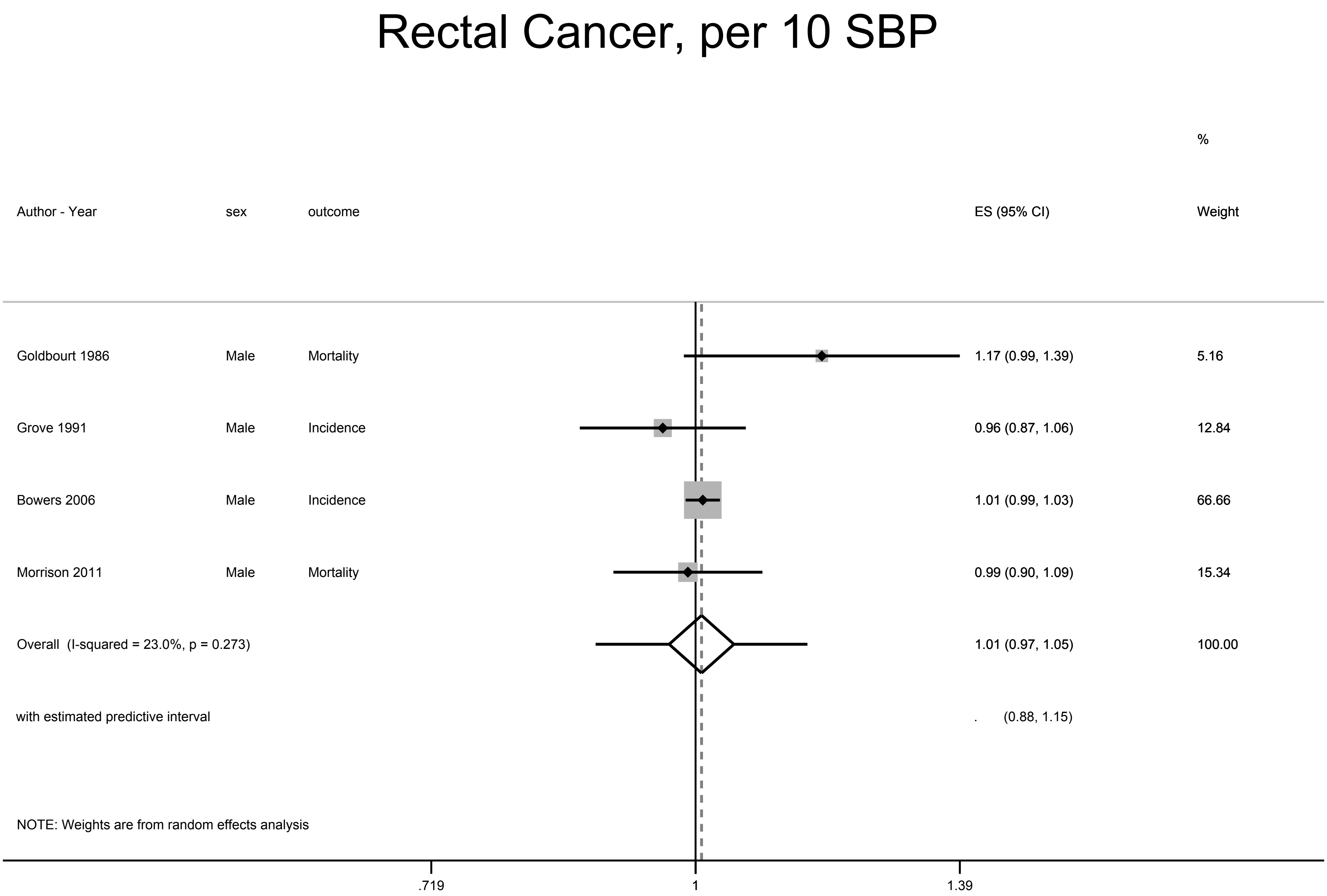

Supplement Figure 34. Meta-analysis of prospective studies for the association between rectal cancer risk and 10mmHg increase in systolic blood pressure.
Abbreviations: SBP, systolic blood pressure.

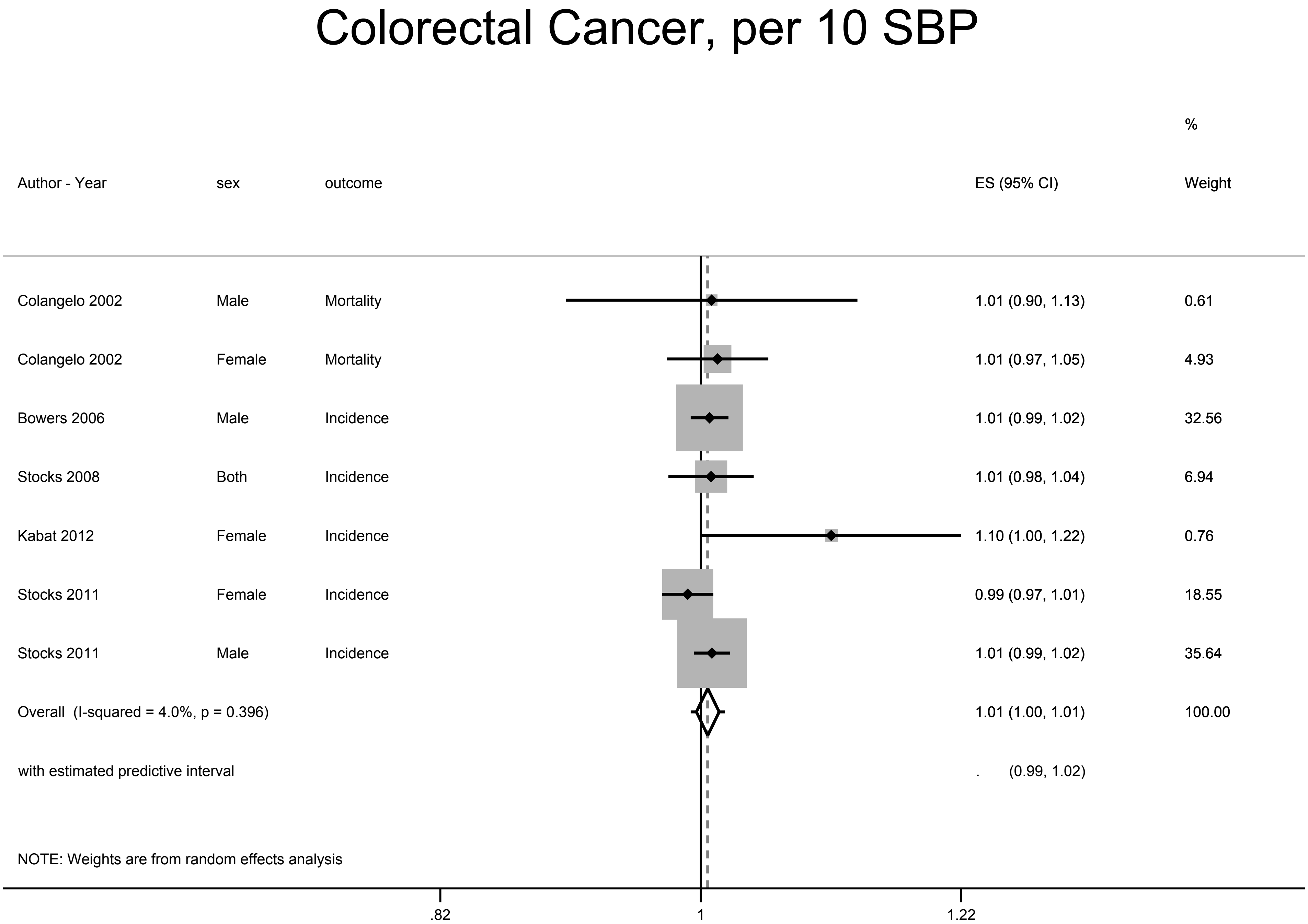

Supplement Figure 35. Meta-analysis of prospective studies for the association between colorectal cancer risk and 10mmHg increase in systolic blood pressure. Abbreviations: SBP, systolic blood pressure.

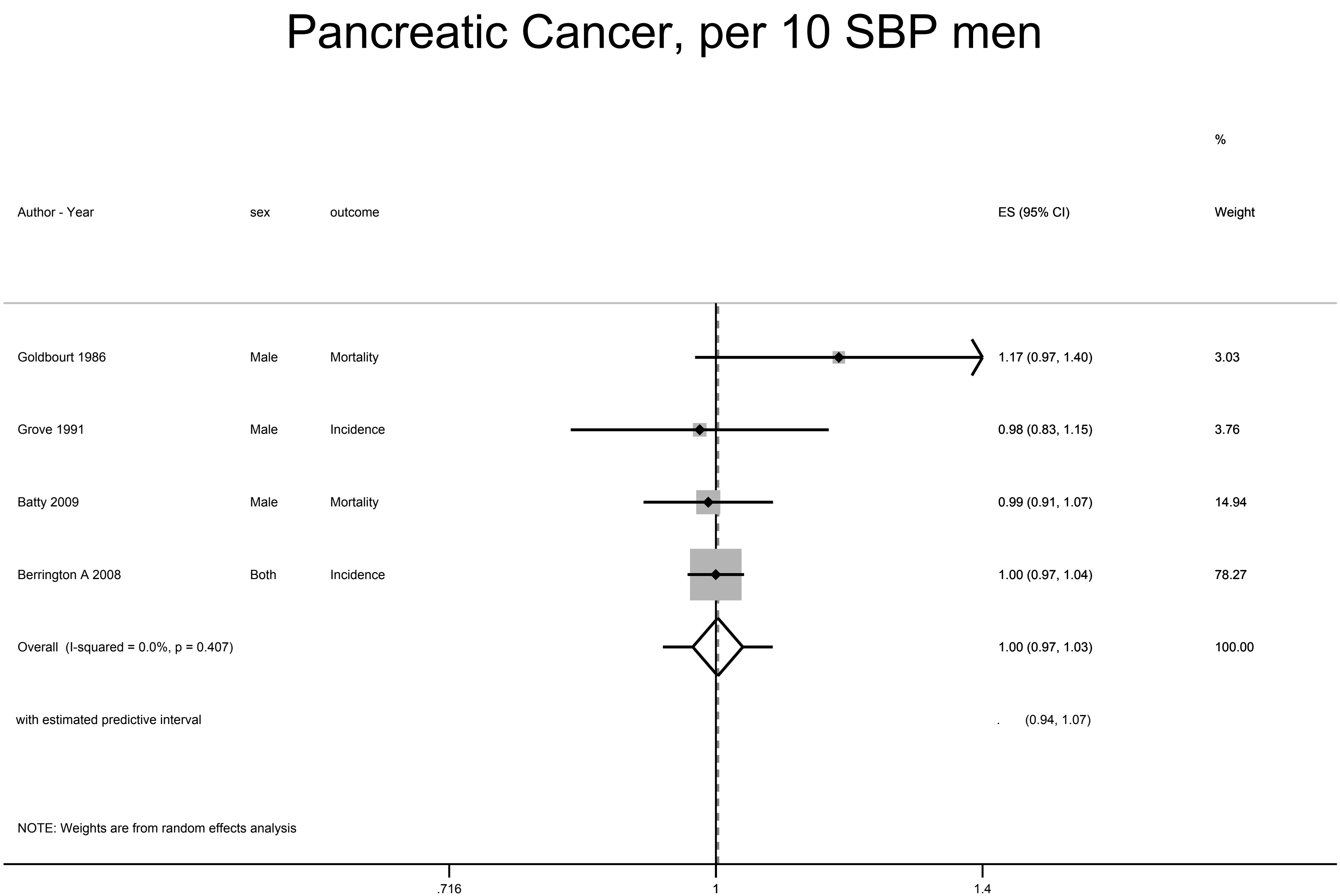

Supplement Figure 36. Meta-analysis of prospective studies for the association between pancreatic cancer risk and 10mmHg increase in systolic blood pressure. Abbreviations: SBP, systolic blood pressure.

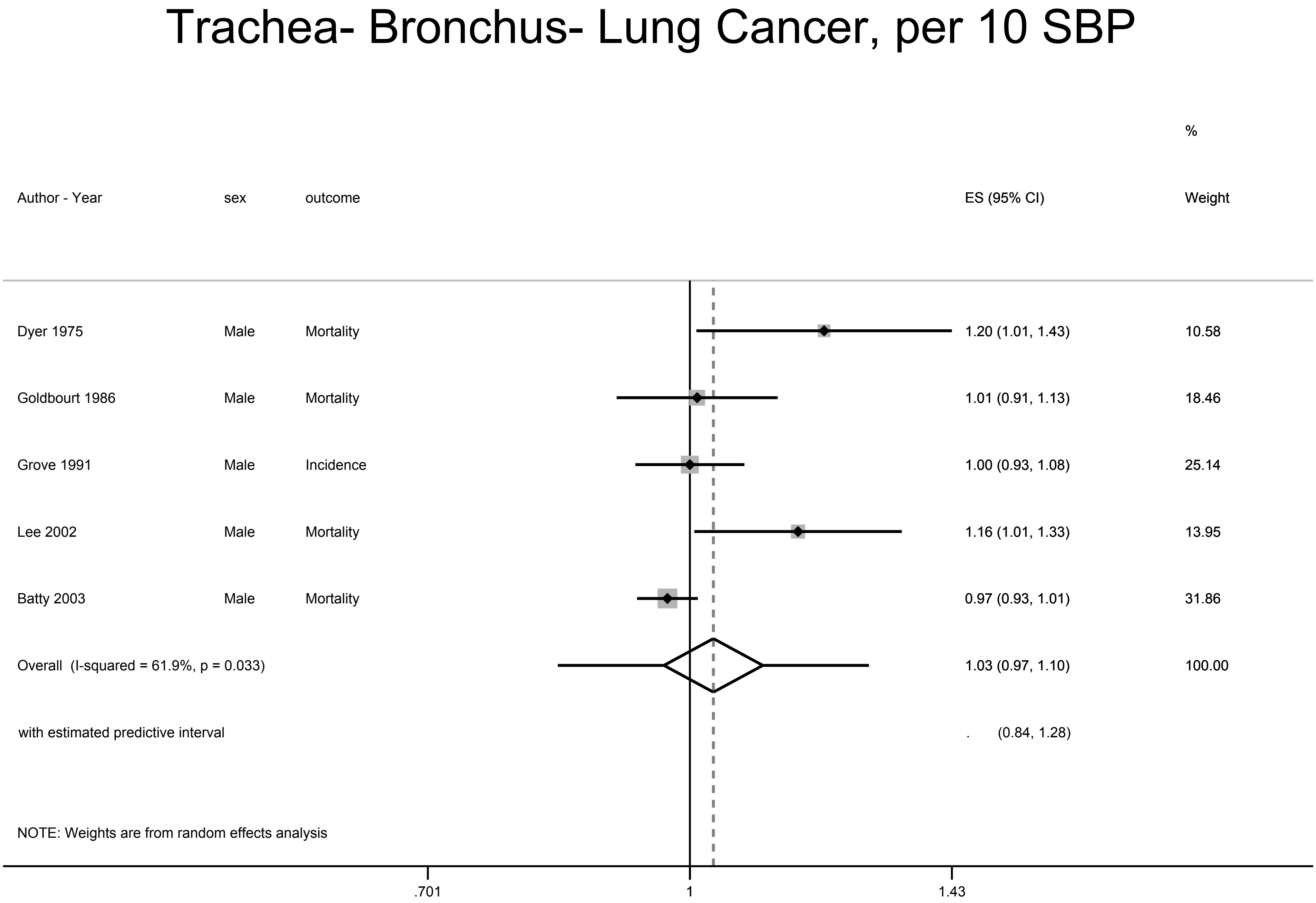

Supplement Figure 37. Meta-analysis of prospective studies for the association between trachea/bronchus/lung cancer risk and 10mmHg increase in systolic blood pressure. Abbreviations: SBP, systolic blood pressure.

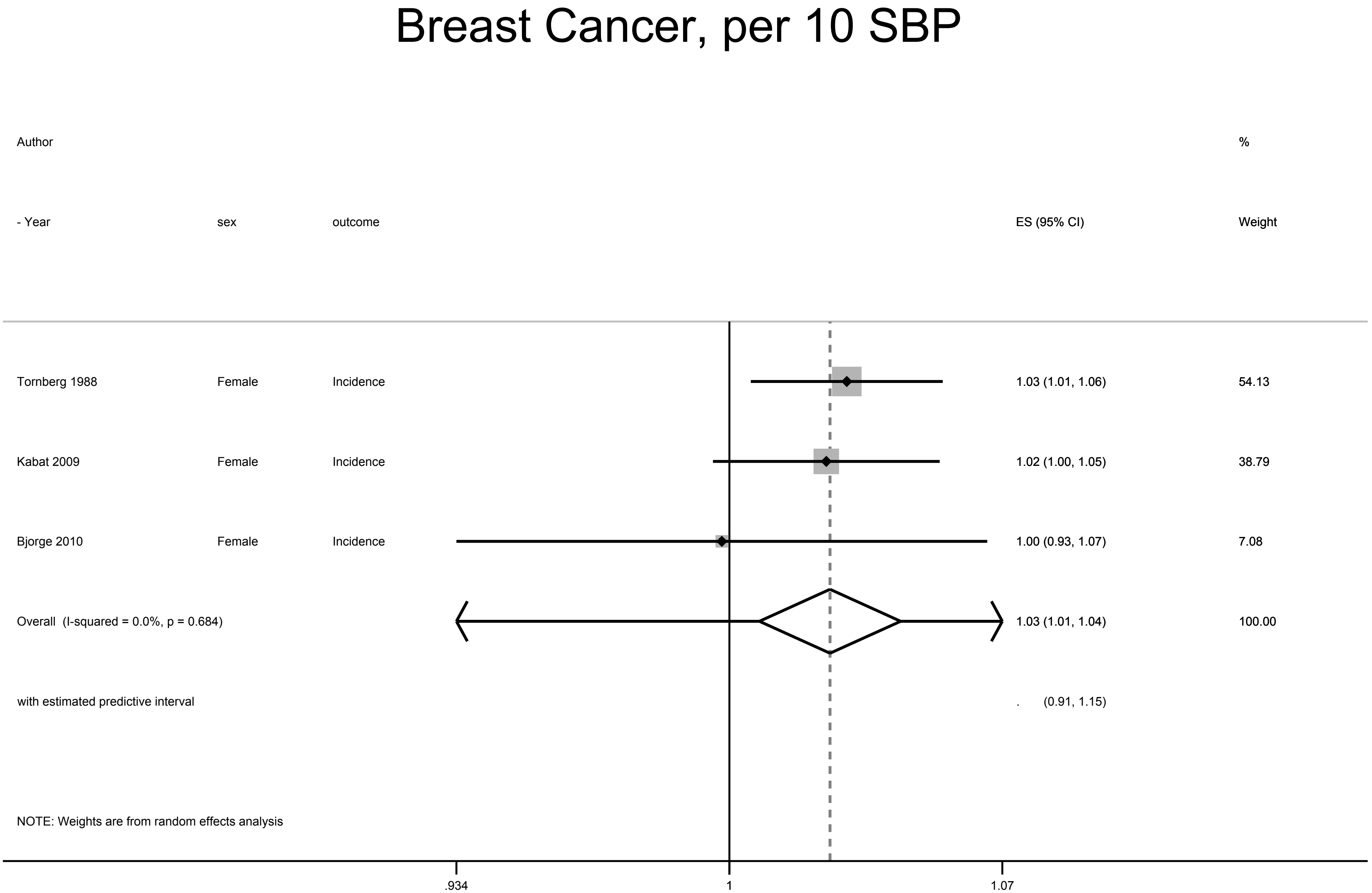

Supplement Figure 38. Meta-analysis of prospective studies for the association between breast cancer risk and 10mmHg increase in systolic blood pressure. Abbreviations: SBP, systolic blood pressure.

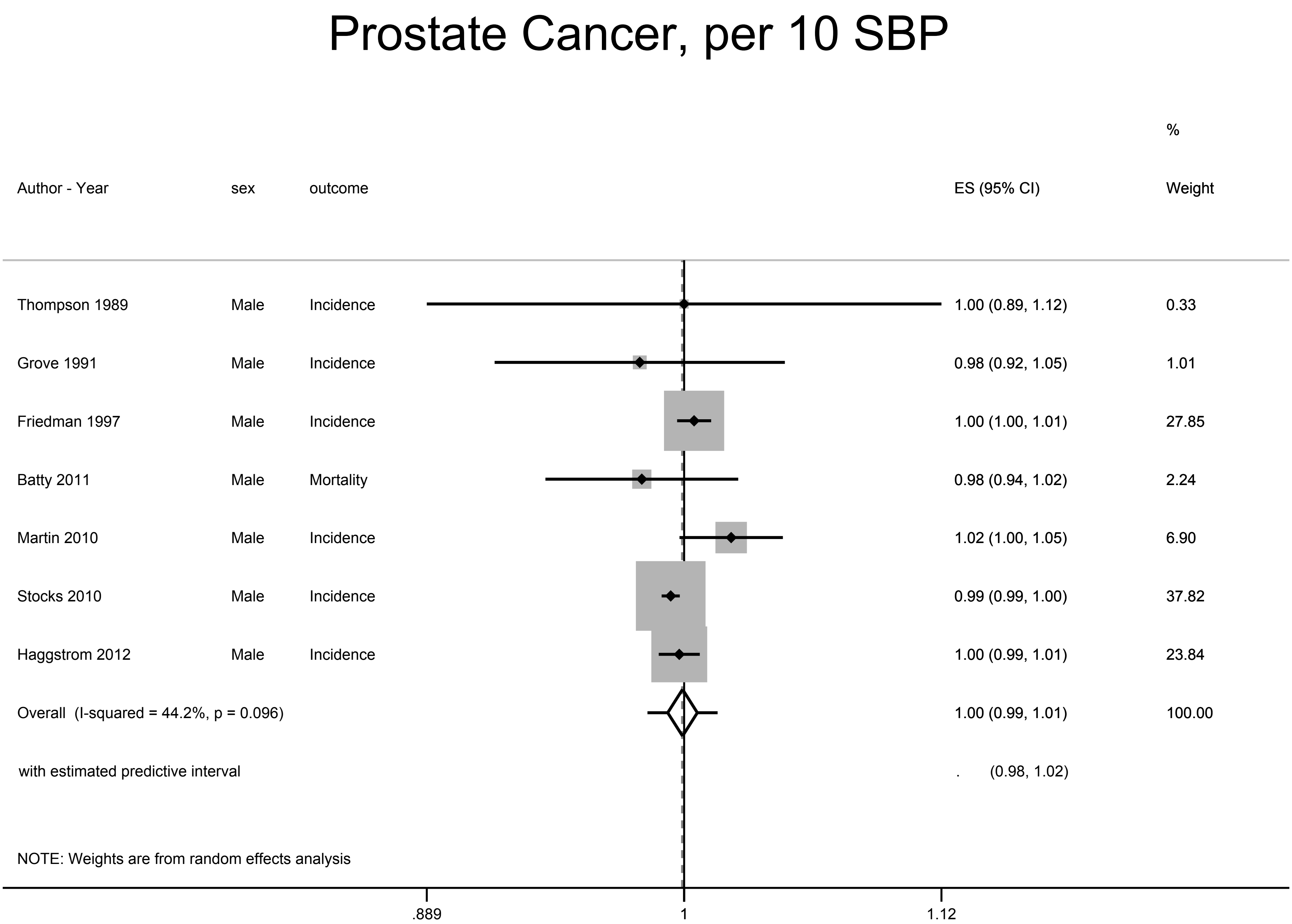

Supplement Figure 39. Meta-analysis of prospective studies for the association between prostate cancer risk and 10mmHg increase in systolic blood pressure.
Abbreviations: SBP, systolic blood pressure.

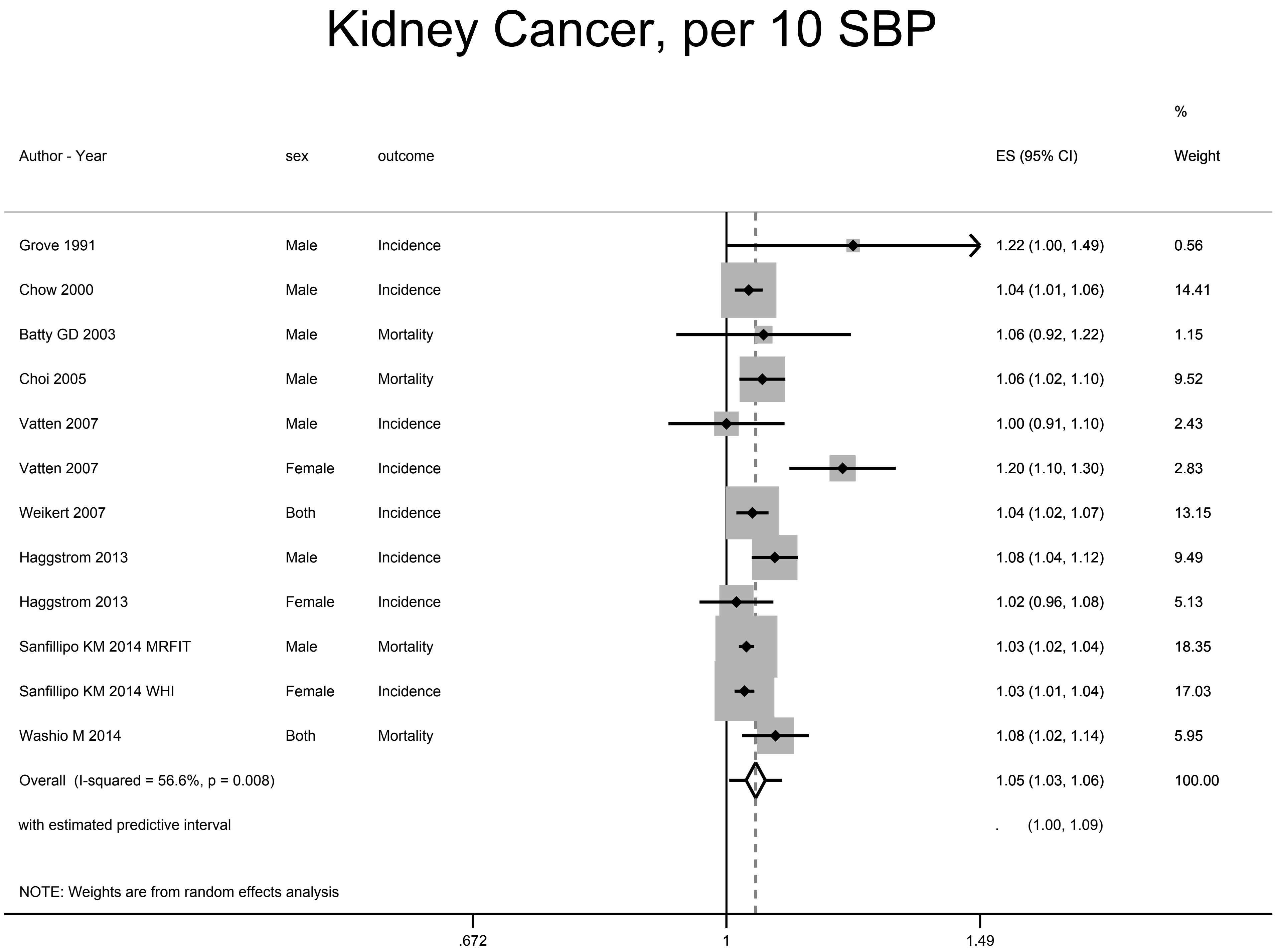

Supplement Figure 40. Meta-analysis of prospective studies for the association between kidney cancer risk and 10mmHg increase in systolic blood pressure. Abbreviations: SBP, systolic blood pressure.

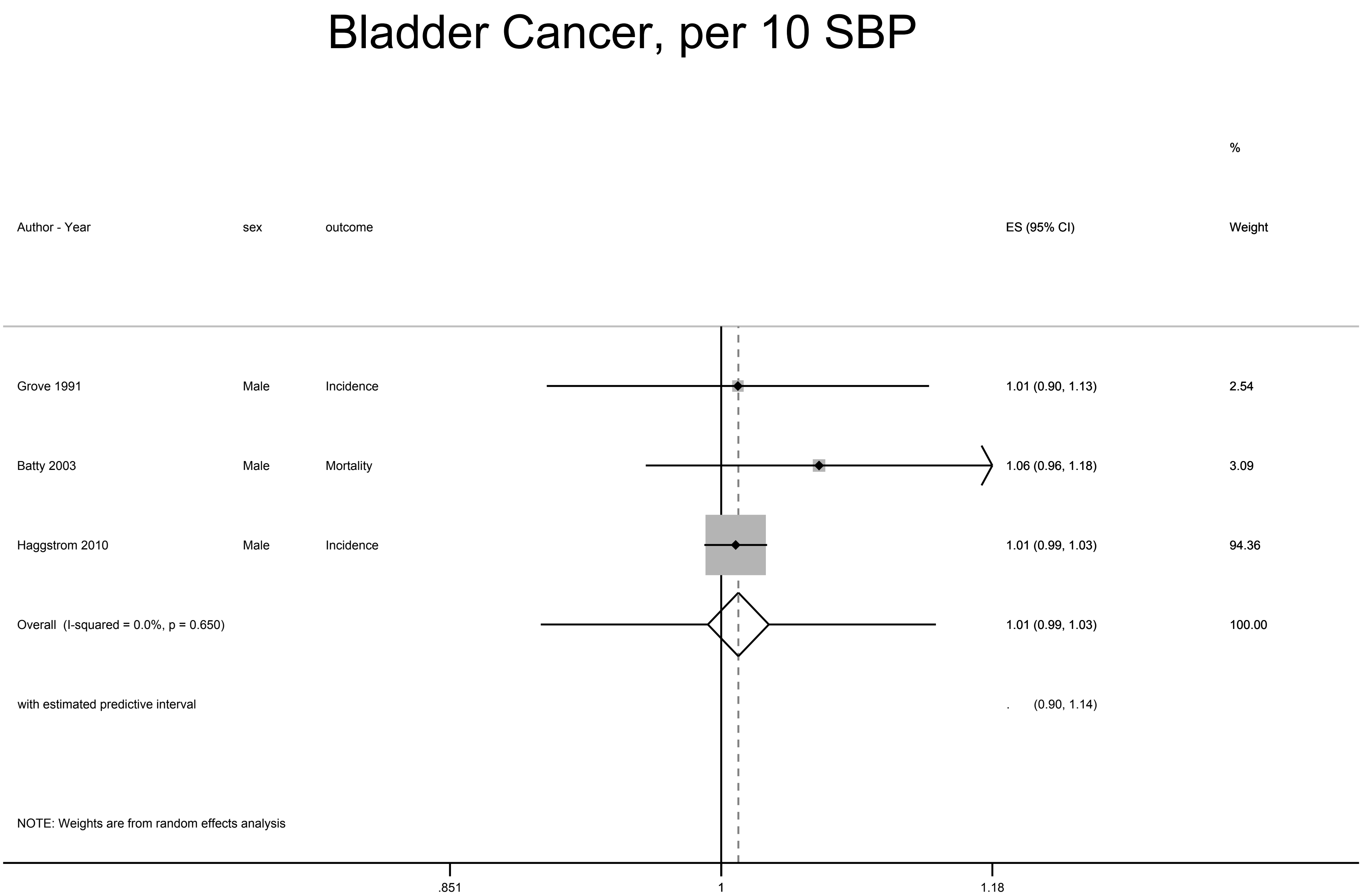

Supplement Figure 41. Meta-analysis of prospective studies for the association between bladder cancer risk and 10mmHg increase in systolic blood pressure.
Footnote: This meta-analysis included only men. Abbreviations: SBP, systolic blood pressure.

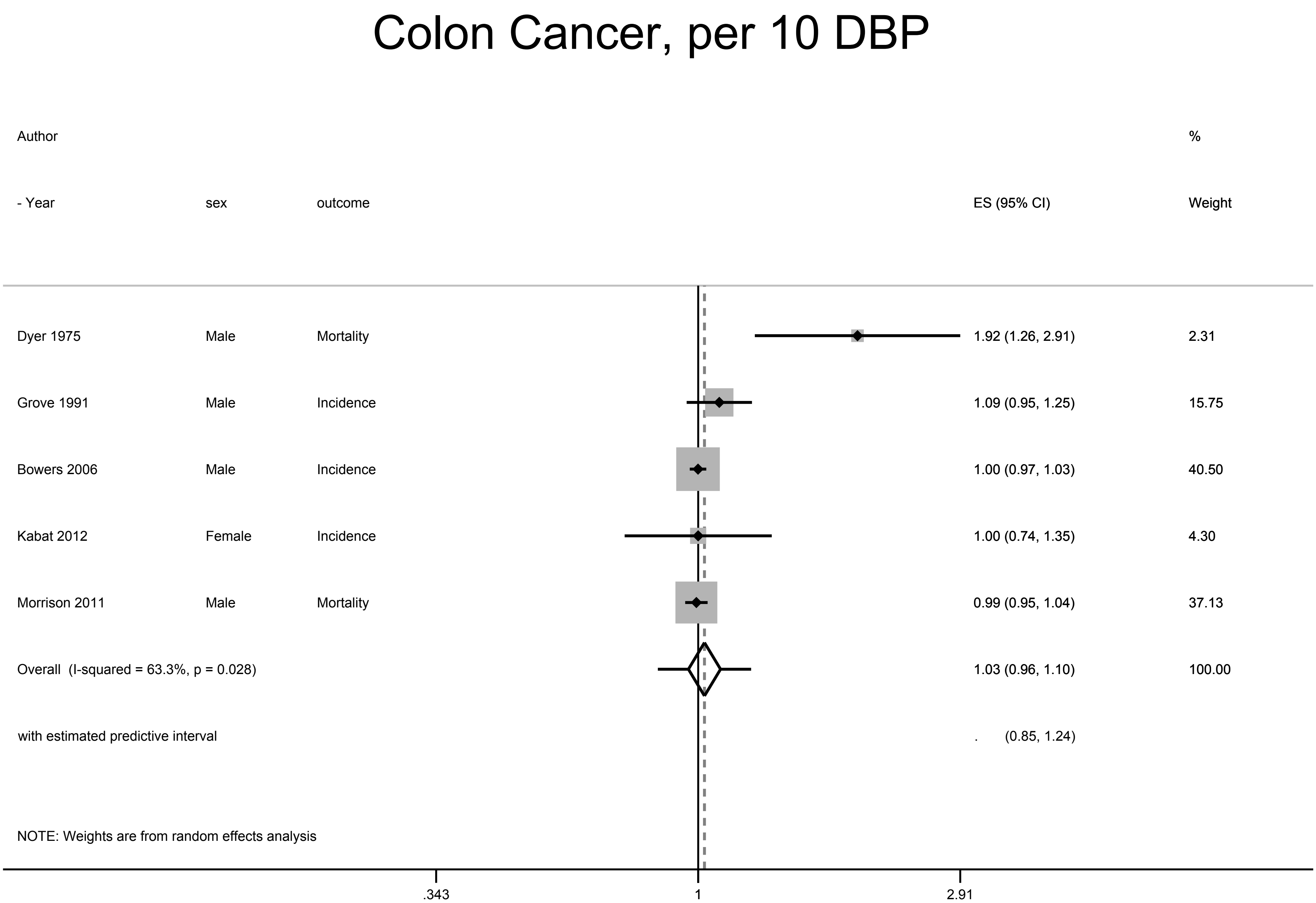

Supplement Figure 42. Meta-analysis of prospective studies for the association between colon cancer risk and 10mmHg increase in diastolic blood pressure. Abbreviations: DBP, diastolic blood pressure.

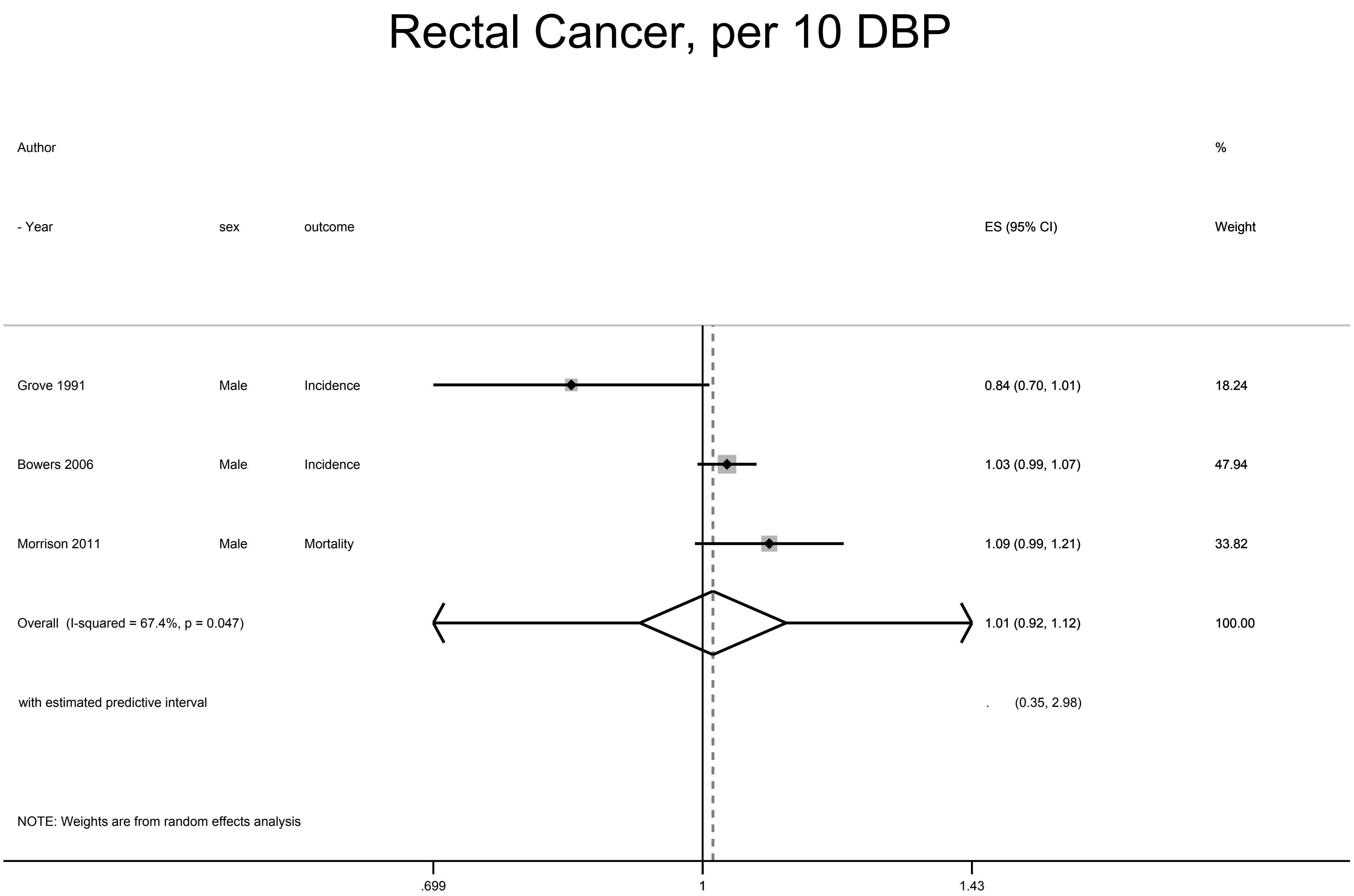

Supplement Figure 43. Meta-analysis of prospective studies for the association between rectal cancer risk and 10mmHg increase in diastolic blood pressure. Abbreviations: DBP, diastolic blood pressure.

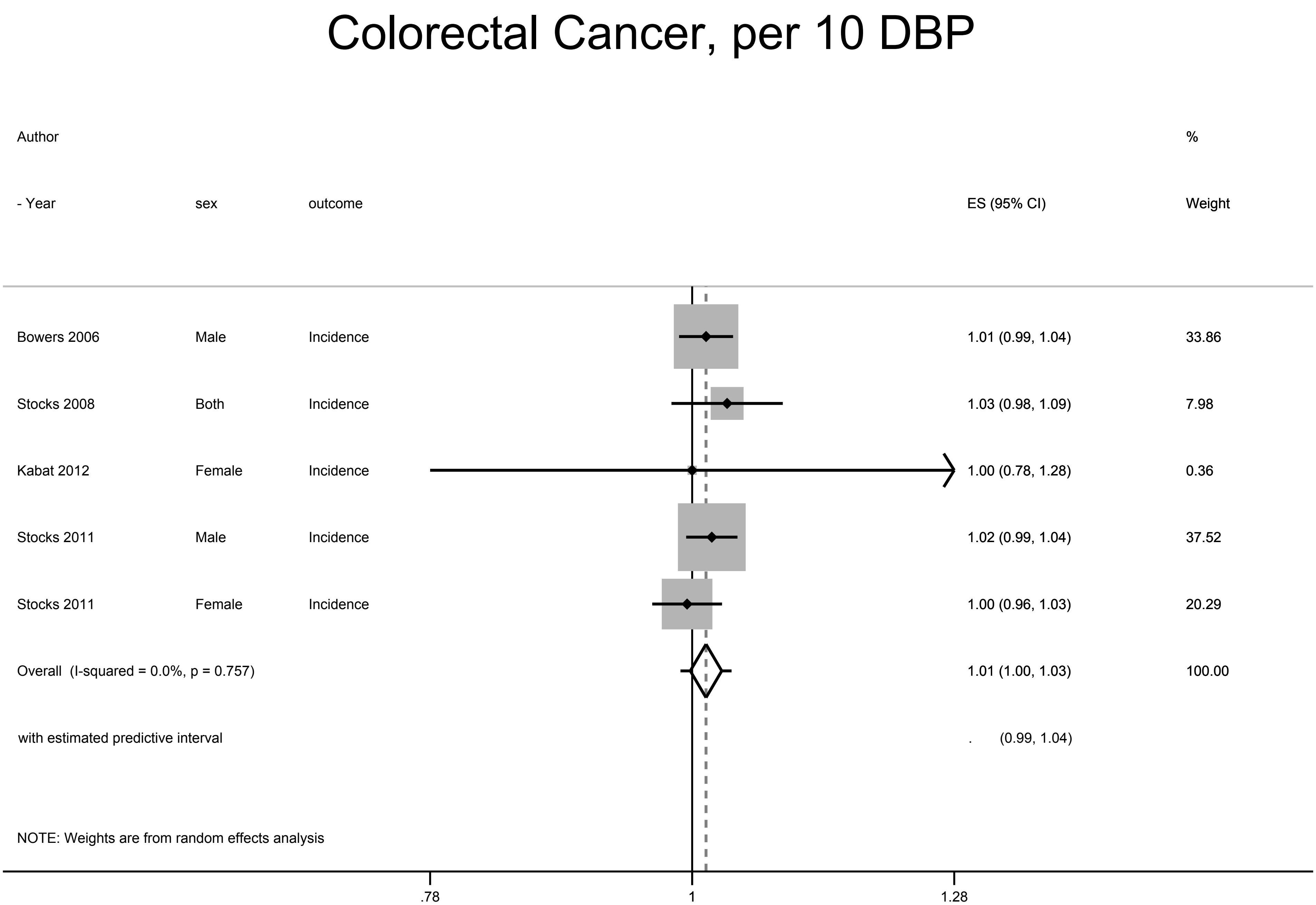

Supplement Figure 44. Meta-analysis of prospective studies for the association between colorectal cancer risk and 10mmHg increase in diastolic blood pressure. Abbreviations: DBP, diastolic blood pressure.

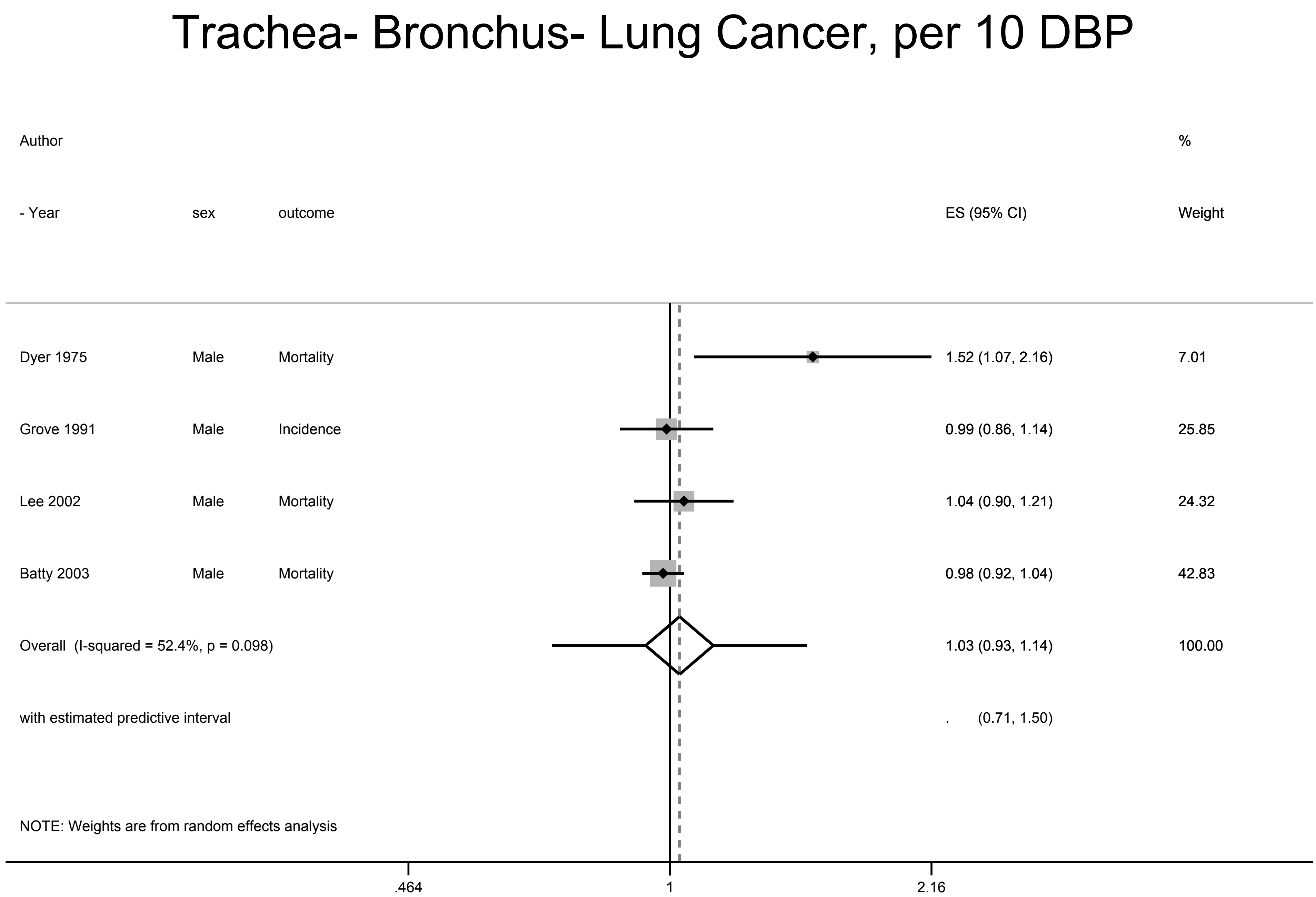

Supplement Figure 45. Meta-analysis of prospective studies for the association between trachea/bronchus/lung cancer risk and 10mmHg increase in diastolic blood pressure. Abbreviations: DBP, diastolic blood pressure.

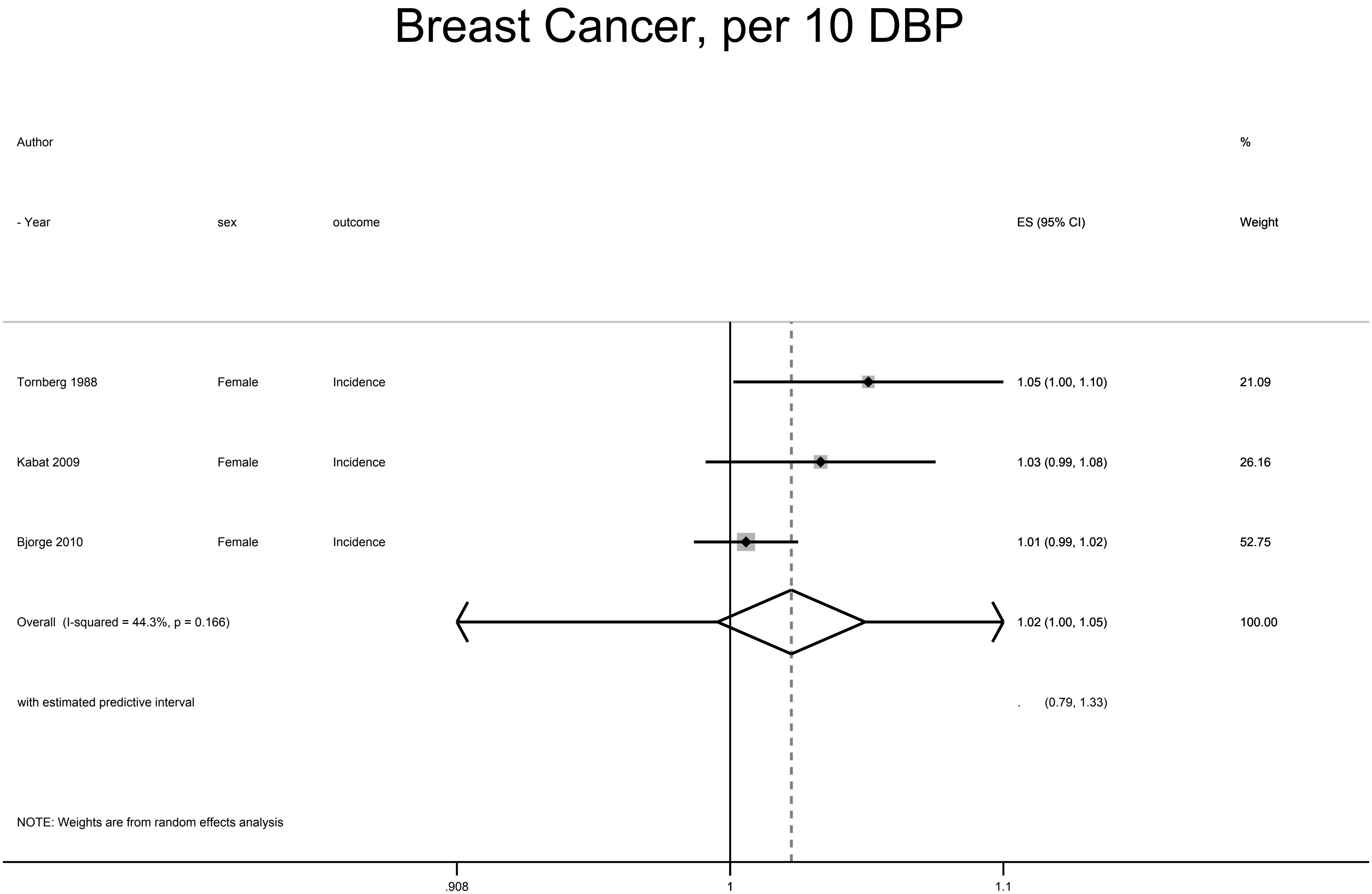

Supplement Figure 46. Meta-analysis of prospective studies for the association between breast cancer risk and 10mmHg increase in diastolic blood pressure. Abbreviations: DBP, diastolic blood pressure.

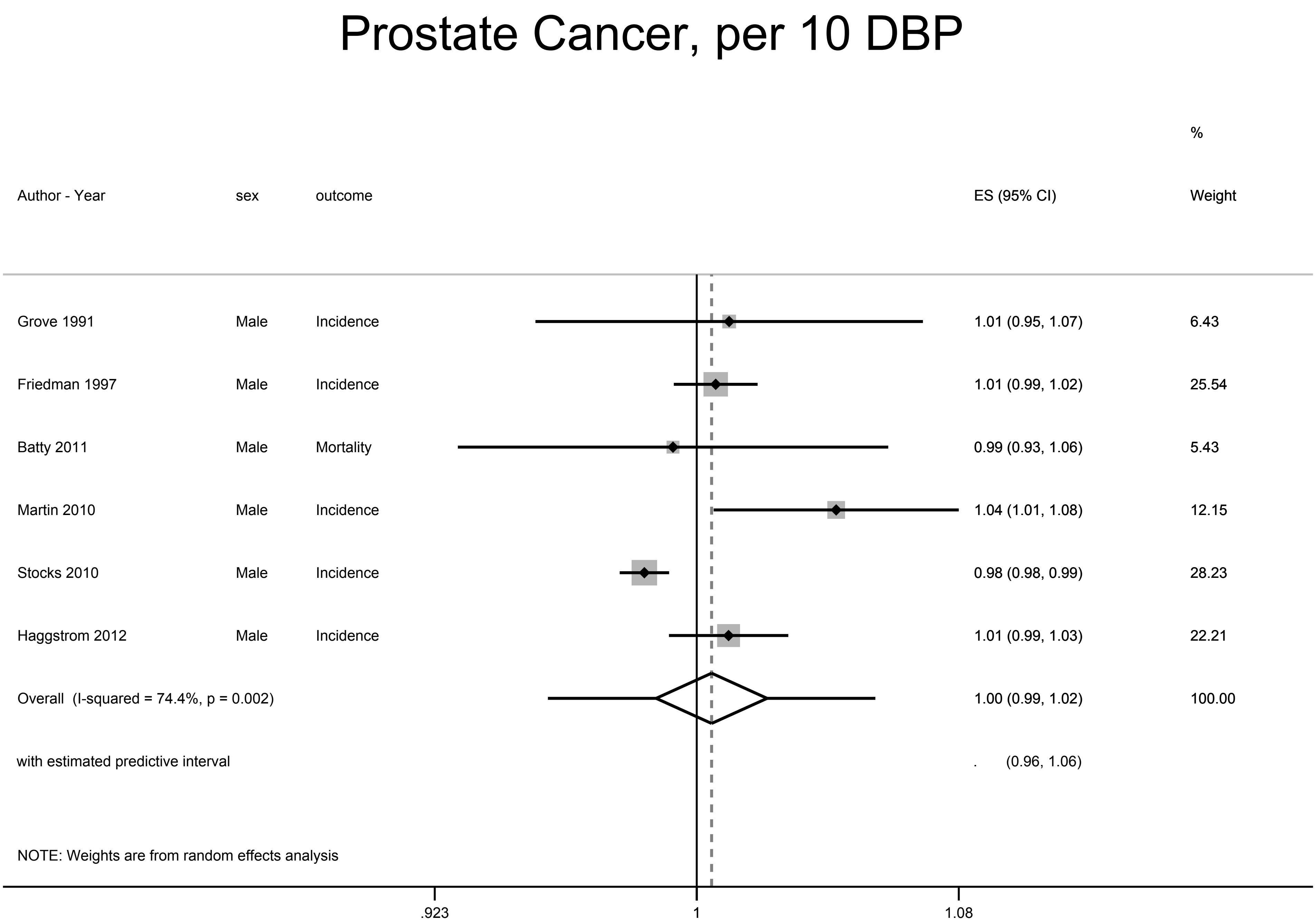

Supplement Figure 47. Meta-analysis of prospective studies for the association between prostate cancer risk and 10mmHg increase in diastolic blood pressure. Abbreviations: DBP, diastolic blood pressure.

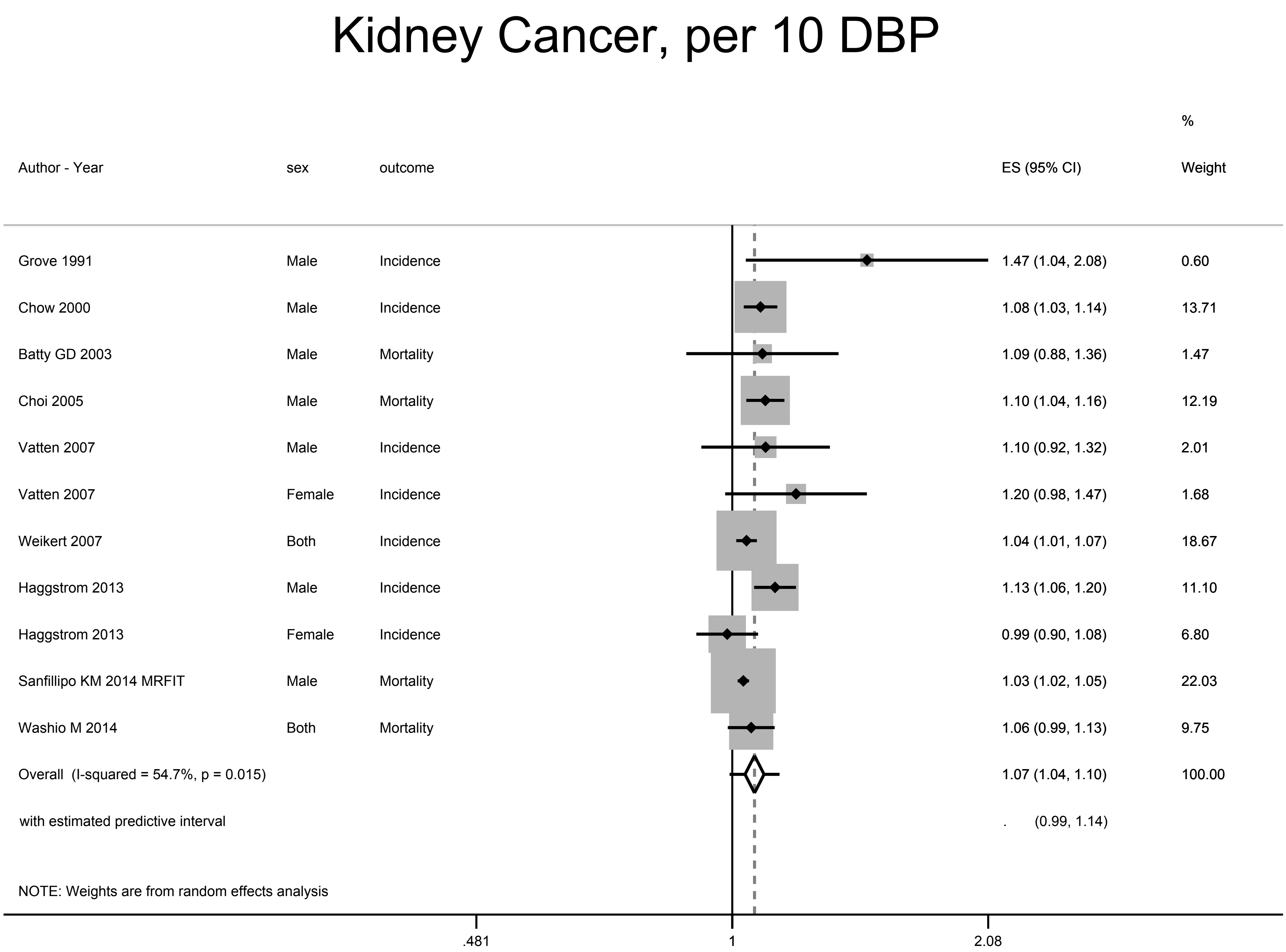

Supplement Figure 48. Meta-analysis of prospective studies for the association between kidney cancer risk and 10mmHg increase in diastolic blood pressure. Abbreviations: DBP, diastolic blood pressure.

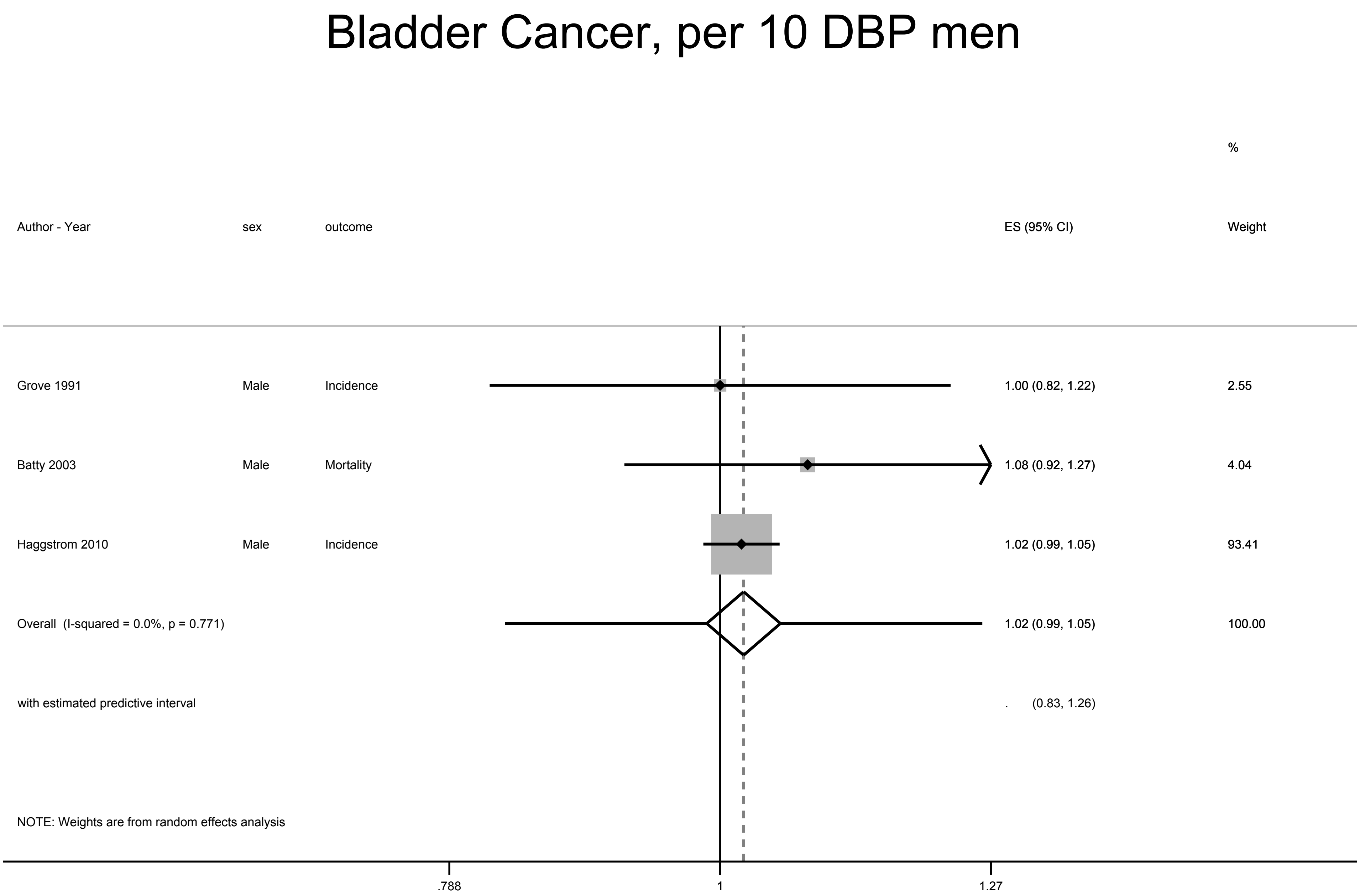

Supplement Figure 49. Meta-analysis of prospective studies for the association between bladder cancer risk and 10mmHg increase in diastolic blood pressure.
Footnote: This meta-analysis included only men. Abbreviations: DBP, diastolic blood pressure.

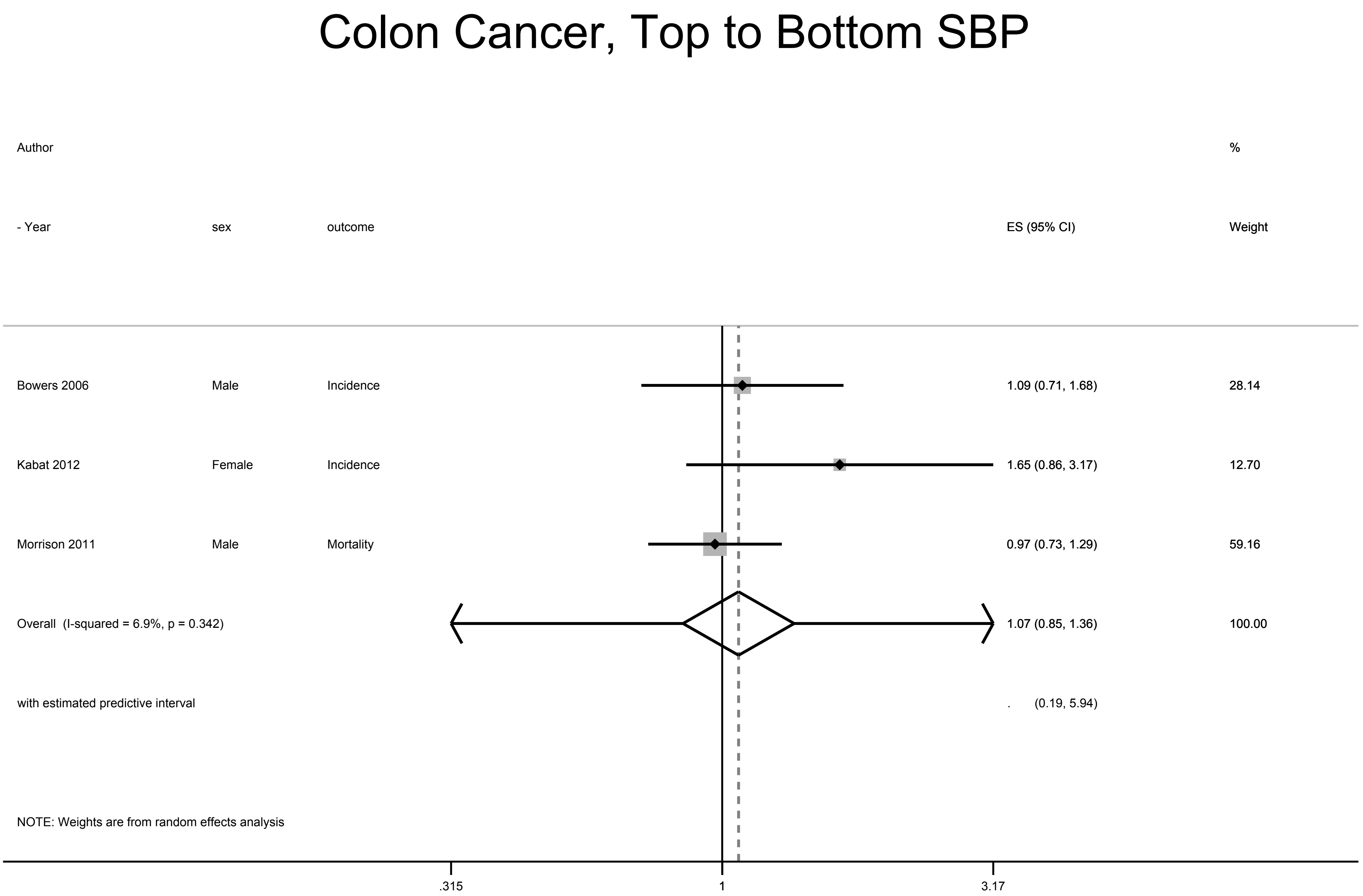

Supplement Figure 50. Meta-analysis of prospective studies for the association between systolic blood pressure (Top vs Bottom) and colon cancer risk.
Abbreviations: SBP, systolic blood pressure.

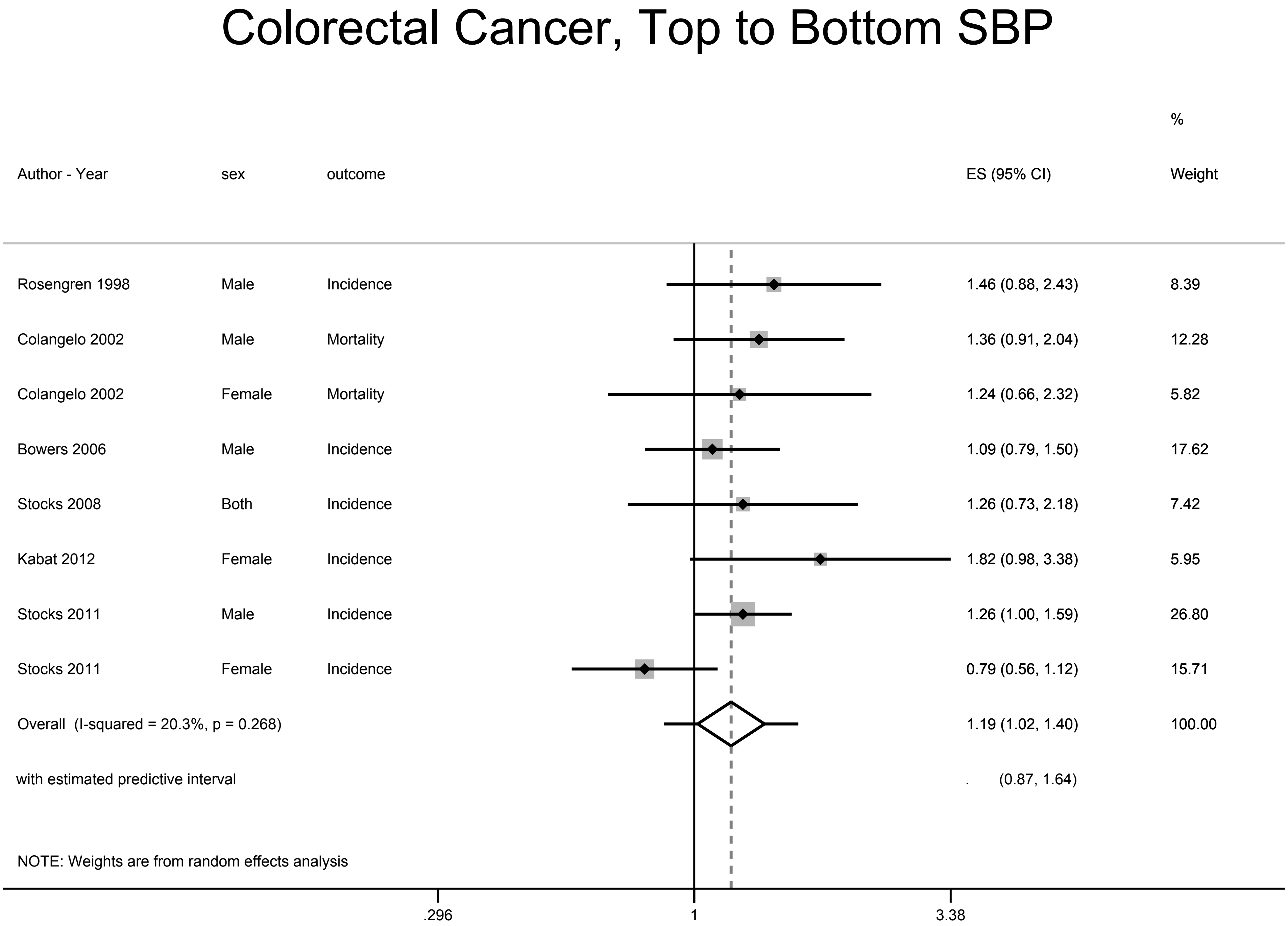

Supplement Figure 51. Meta-analysis of prospective studies for the association between systolic blood pressure (Top vs Bottom) and colorectal cancer risk. Abbreviations: SBP, systolic blood pressure.

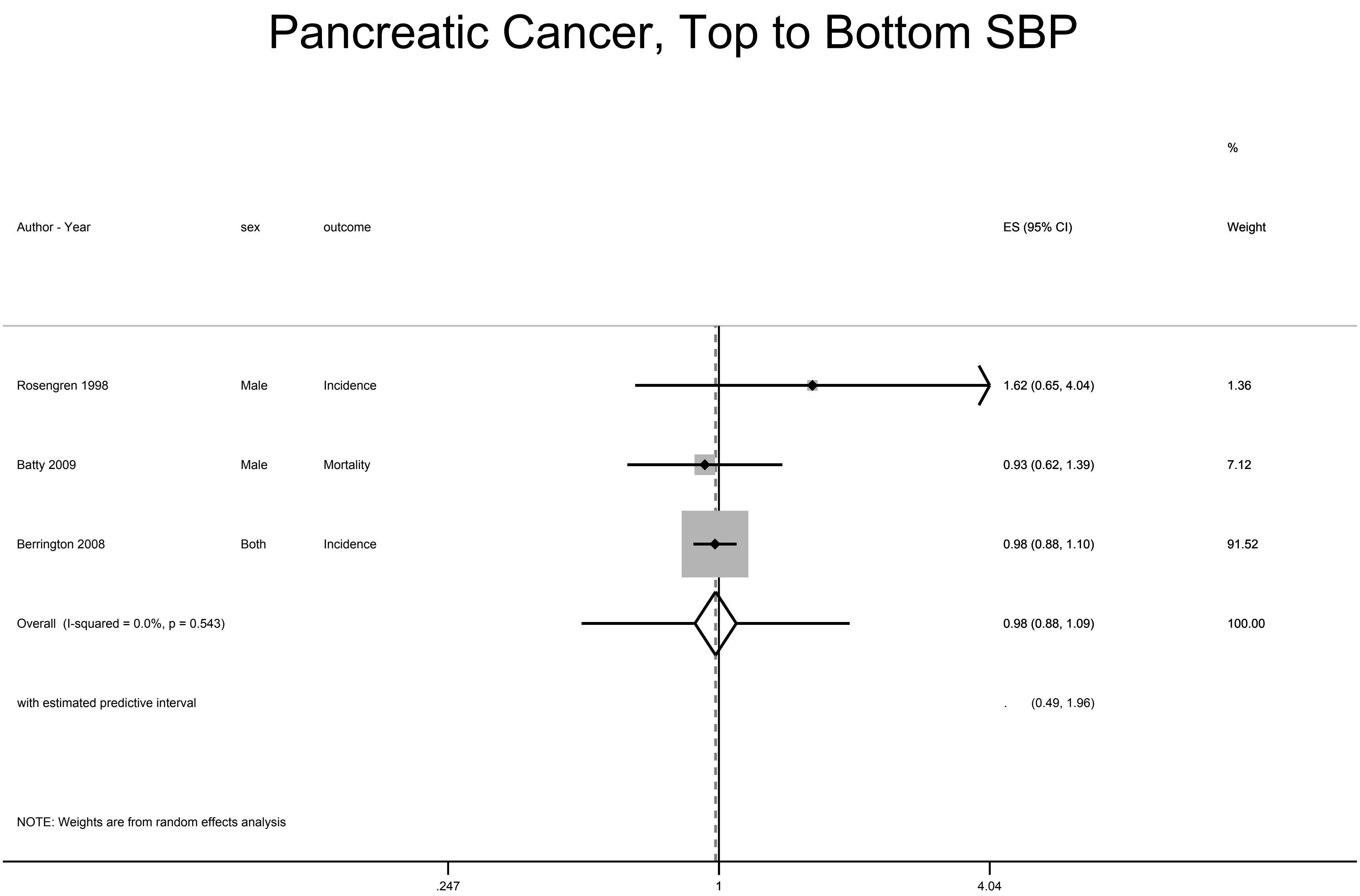

Supplement Figure 52. Meta-analysis of prospective studies for the association between systolic blood pressure (Top vs Bottom) and pancreatic cancer risk.
Abbreviations: SBP, systolic blood pressure.

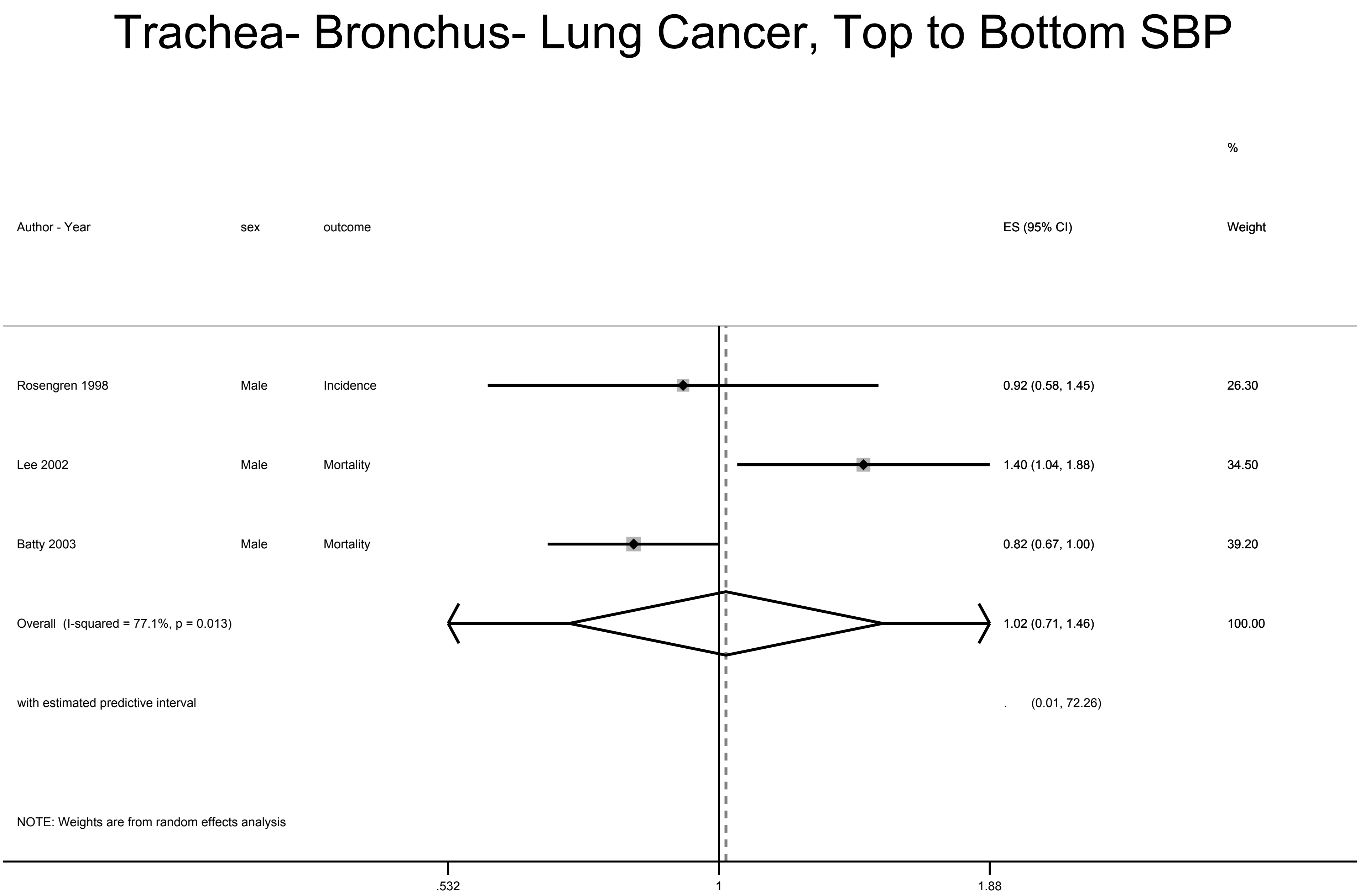

Supplement Figure 53. Meta-analysis of prospective studies for the association between systolic blood pressure (Top vs Bottom) and trachea/bronchus/lung cancer risk. Abbreviations: SBP, systolic blood pressure.

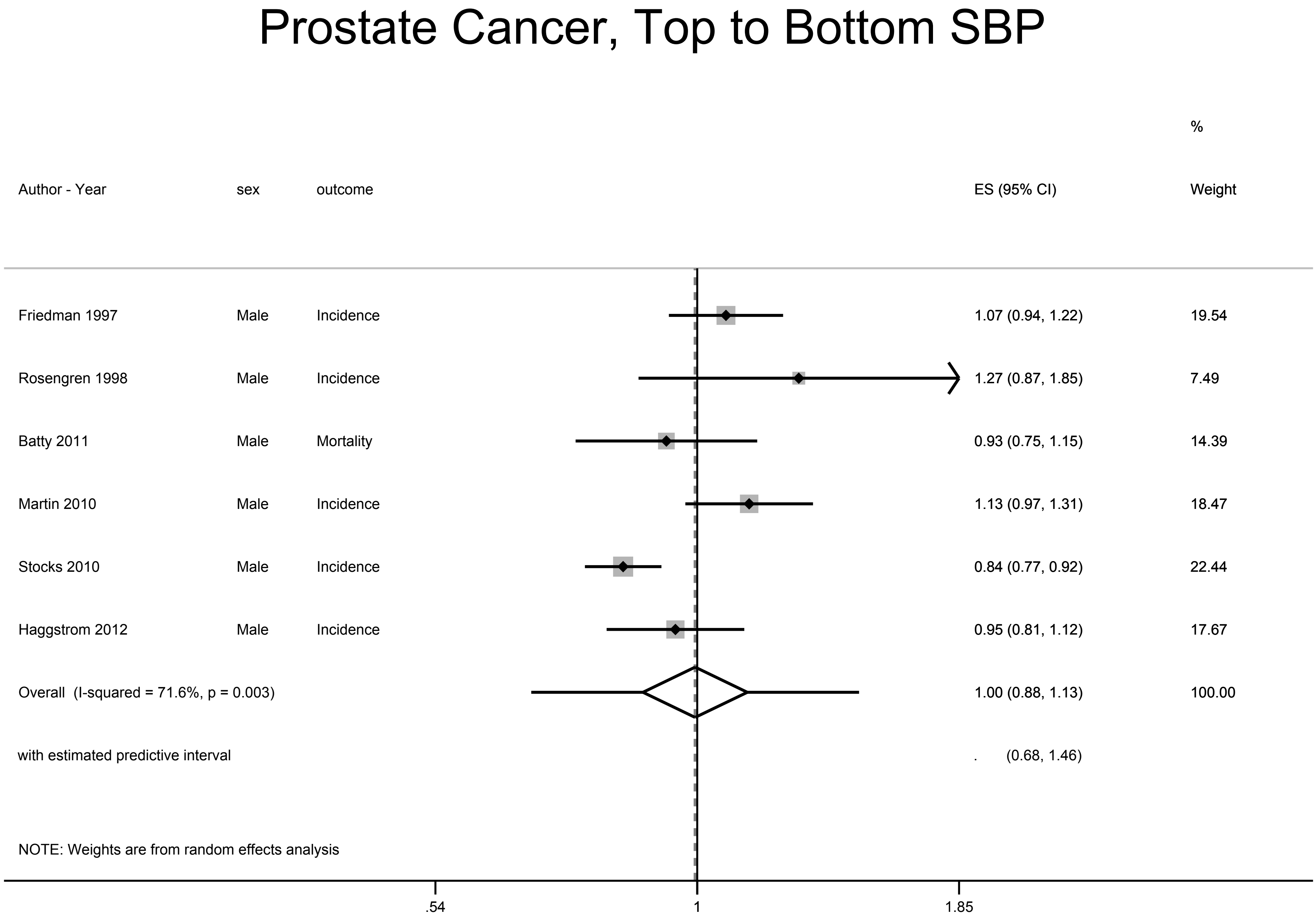

Supplement Figure 54. Meta-analysis of prospective studies for the association between systolic blood pressure (Top vs Bottom) and prostate cancer risk. Abbreviations: SBP, systolic blood pressure.

Kidney Cancer, Top to Bottom SBP

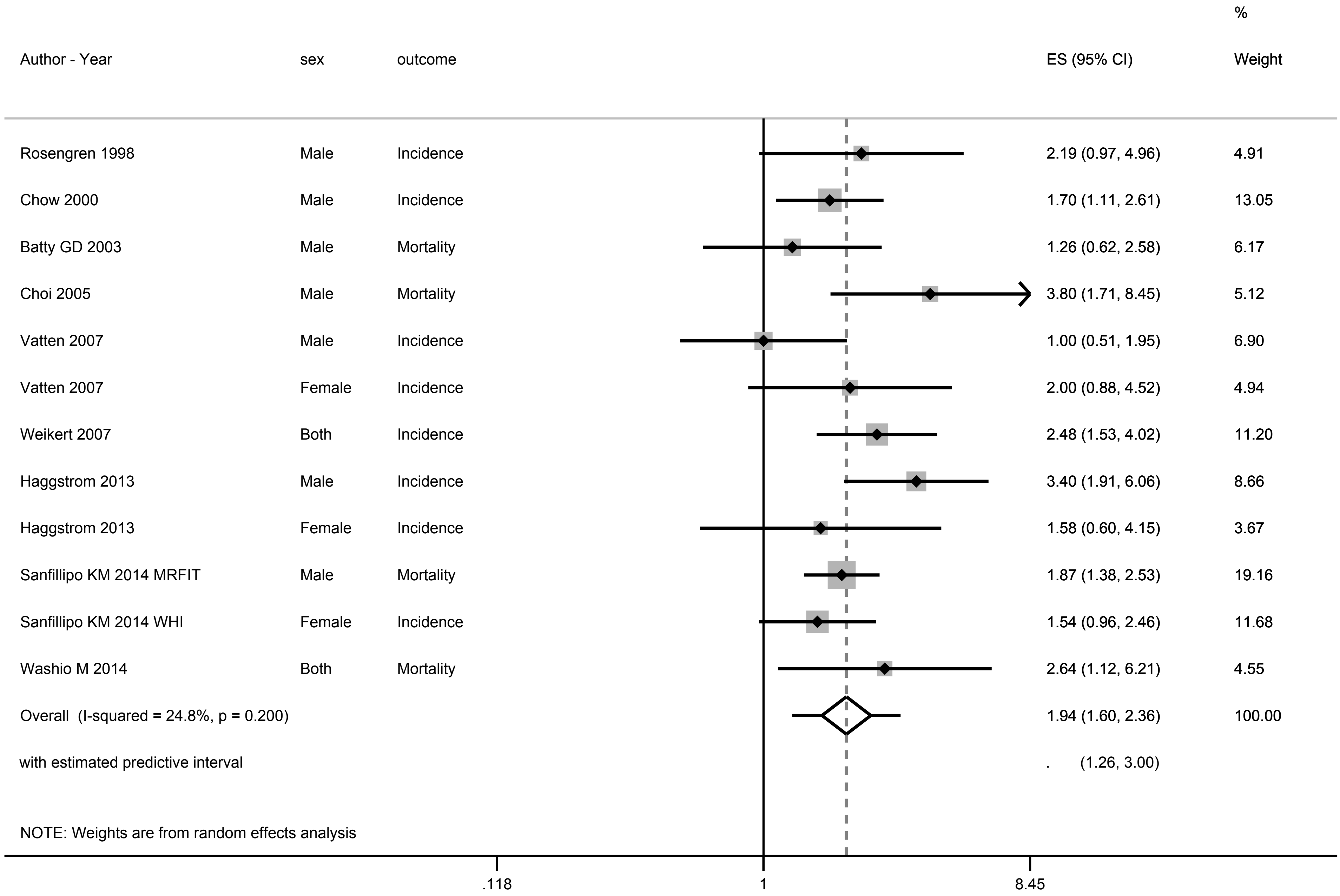

Supplement Figure 55. Meta-analysis of prospective studies for the association between systolic blood pressure (Top vs Bottom) and kidney cancer risk. Abbreviations: SBP, systolic blood pressure.

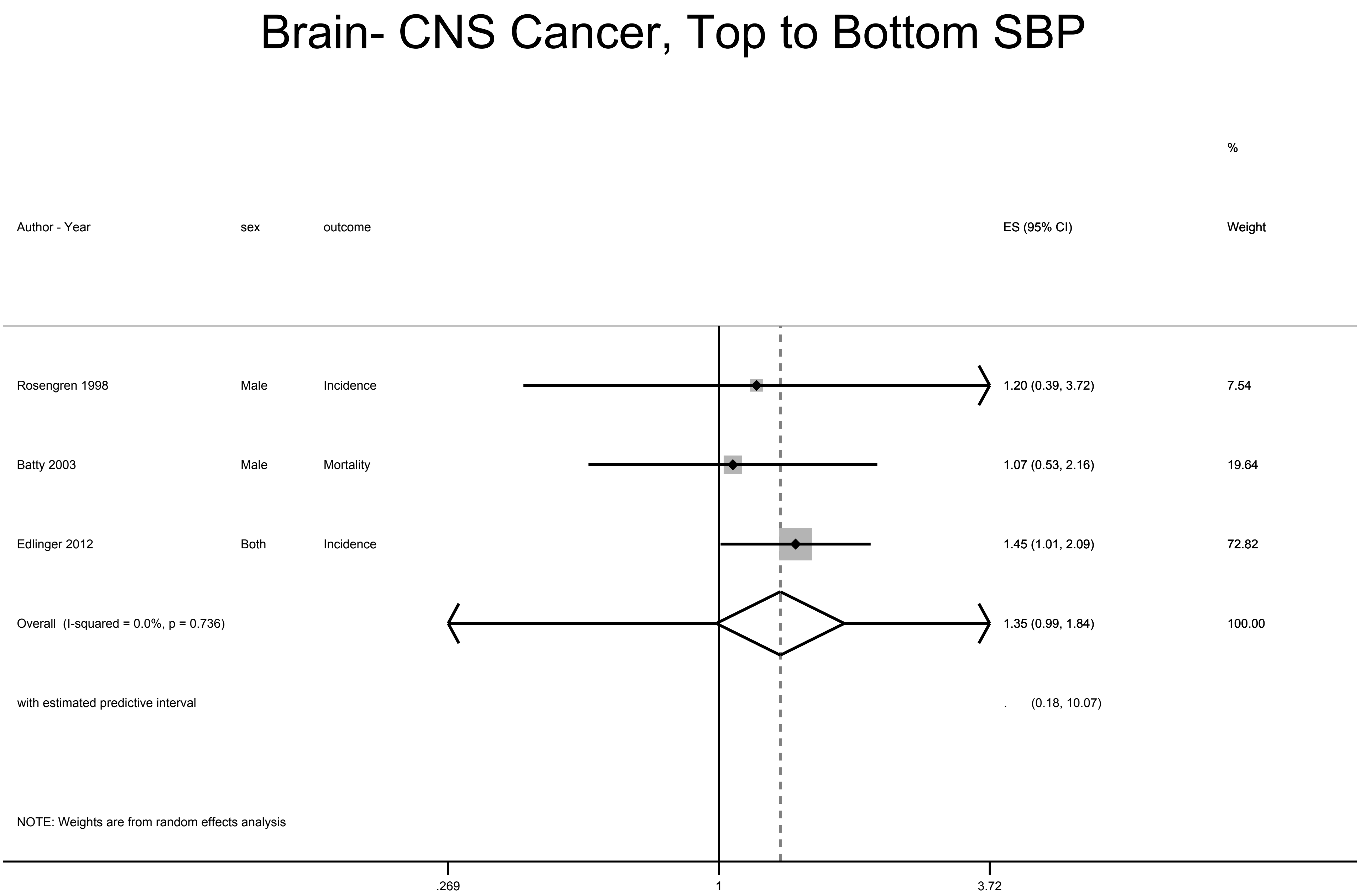

Supplement Figure 56. Meta-analysis of prospective studies for the association between systolic blood pressure (Top vs Bottom) and brain/CNS cancer risk. Abbreviations: SBP, systolic blood pressure; CNS, central nervous system.

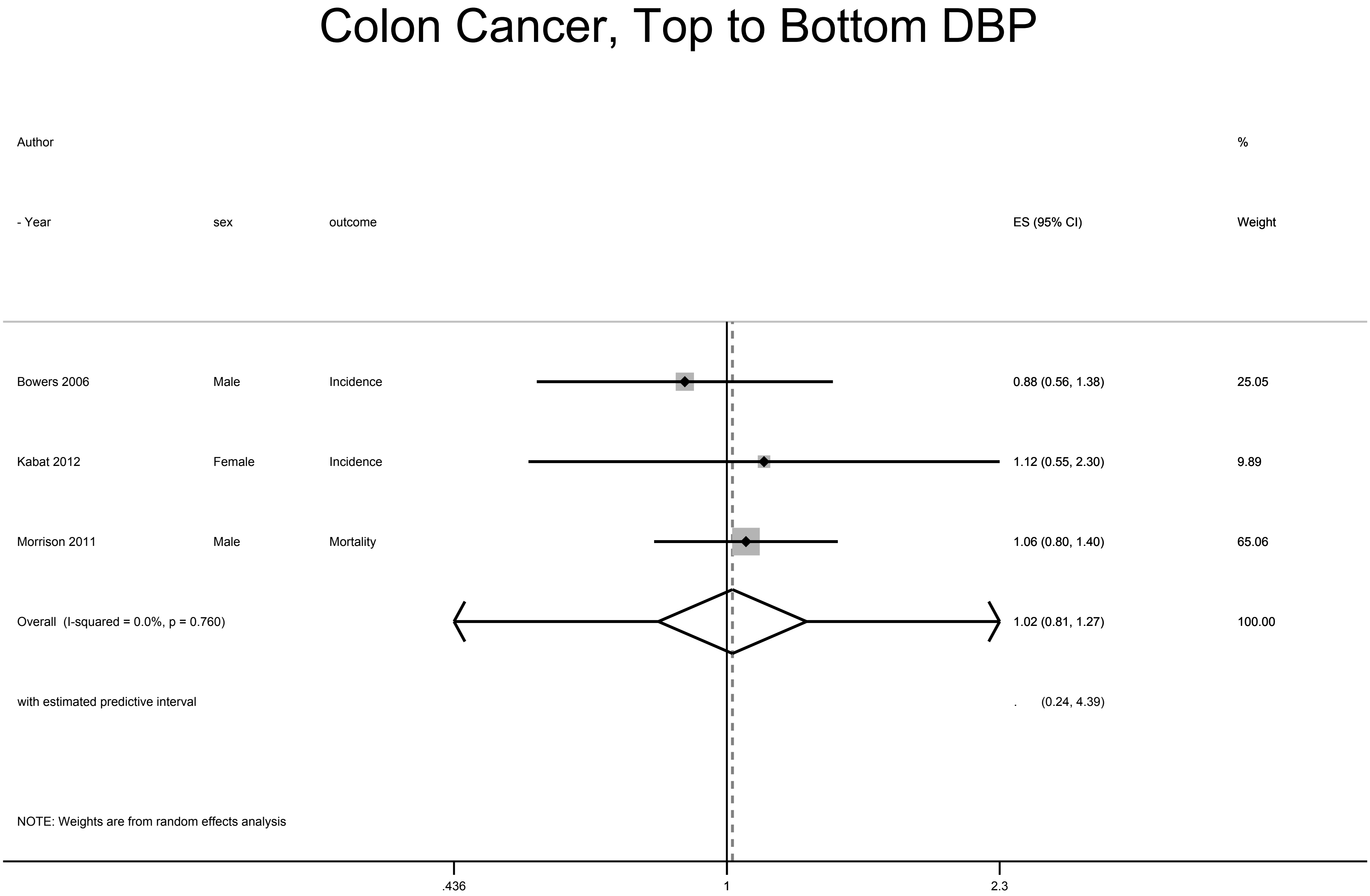

Supplement Figure 57. Meta-analysis of prospective studies for the association between diastolic blood pressure (Top vs Bottom) and colon cancer risk.
Abbreviations: DBP, diastolic blood pressure.

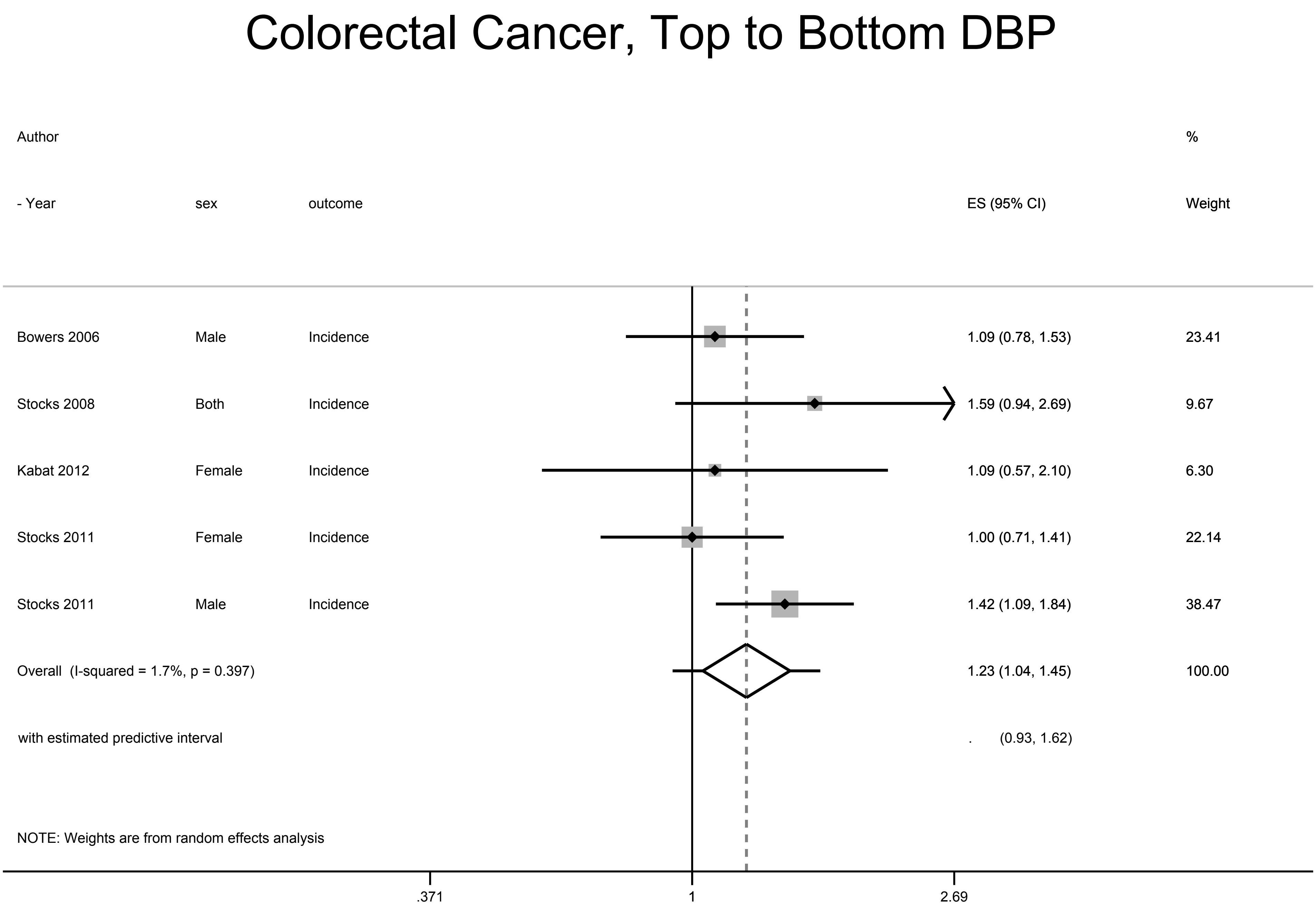

Supplement Figure 58. Meta-analysis of prospective studies for the association between diastolic blood pressure (Top vs Bottom) and colorectal cancer risk. Abbreviations: DBP, diastolic blood pressure.

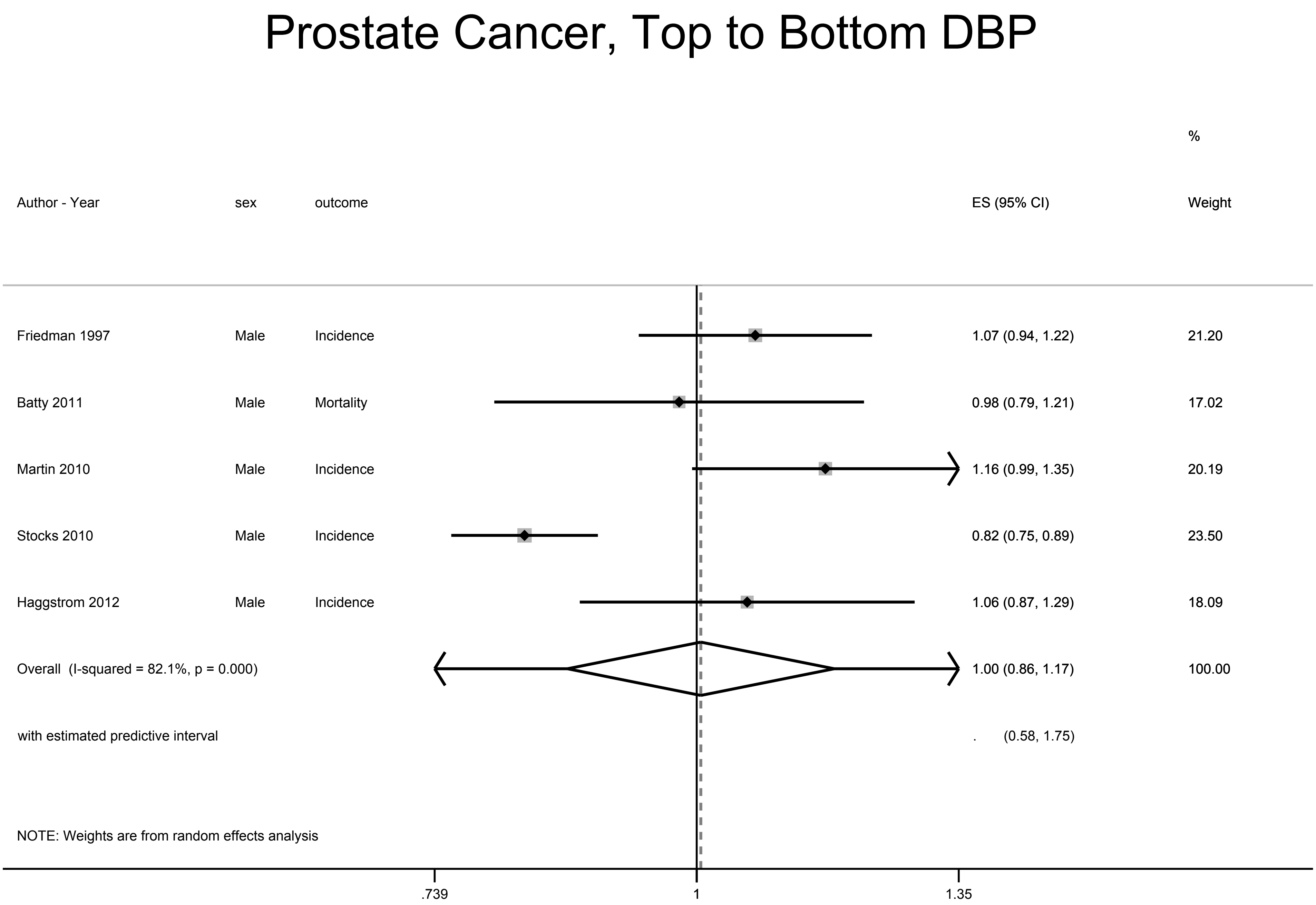

Supplement Figure 59. Meta-analysis of prospective studies for the association between diastolic blood pressure (Top vs Bottom) and prostate cancer risk. Abbreviations: DBP, diastolic blood pressure.

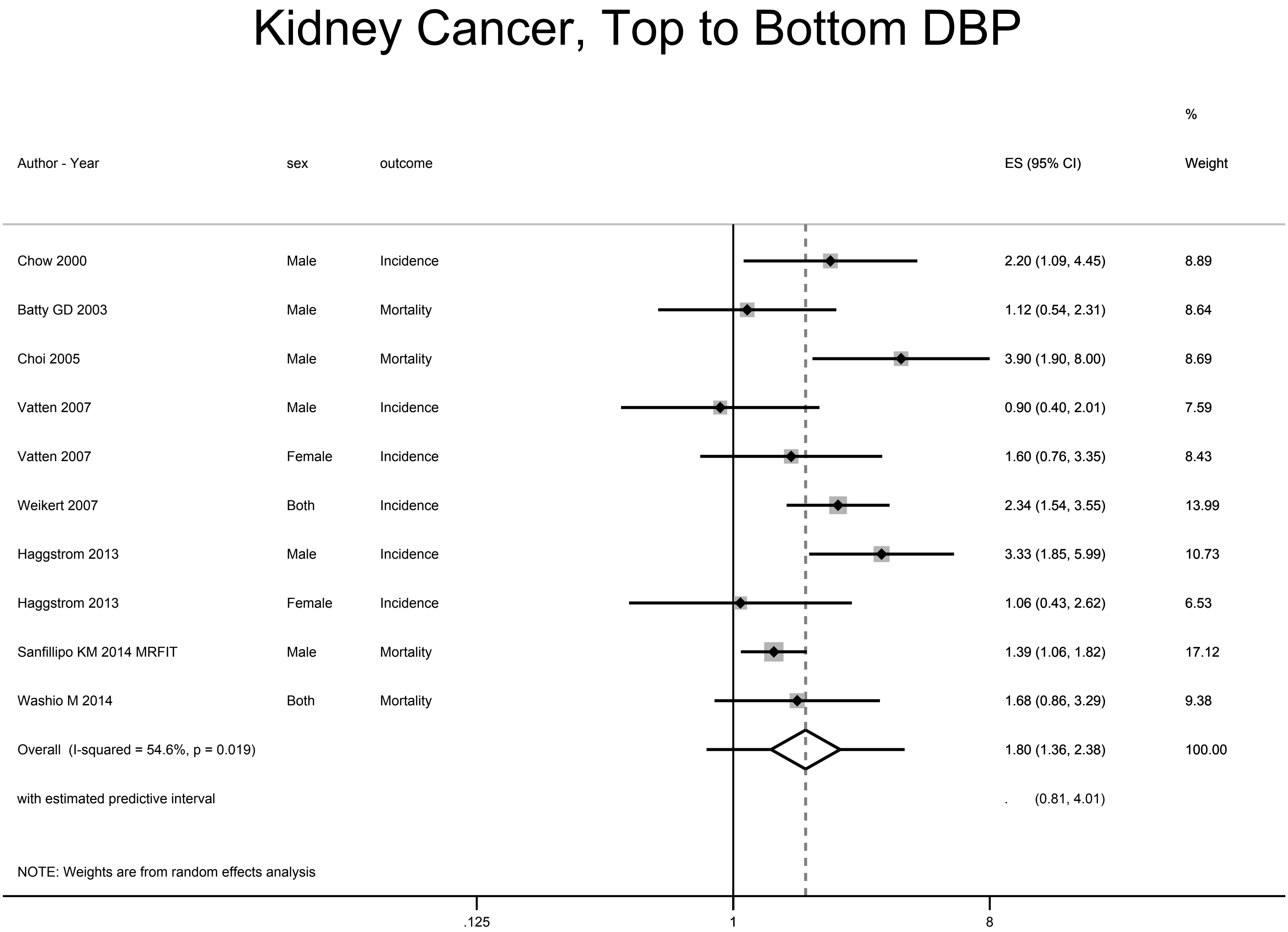

Supplement Figure 60. Meta-analysis of prospective studies for the association between diastolic blood pressure (Top vs Bottom) and kidney cancer risk. Abbreviations: DBP, diastolic blood pressure.

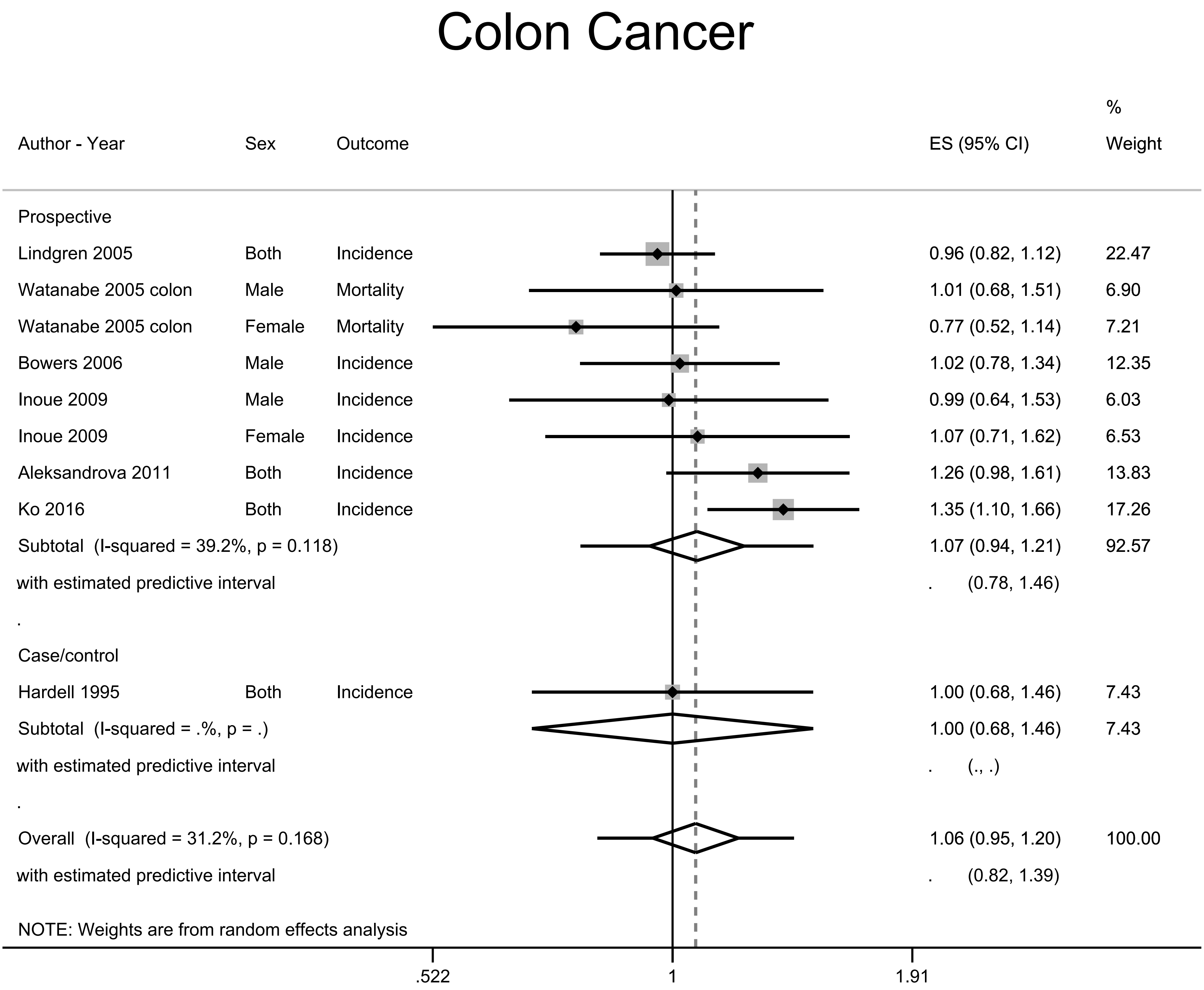

Supplement Figure 61. Meta-analysis of prospective and case-control studies for the association between hypertension and colon cancer risk.

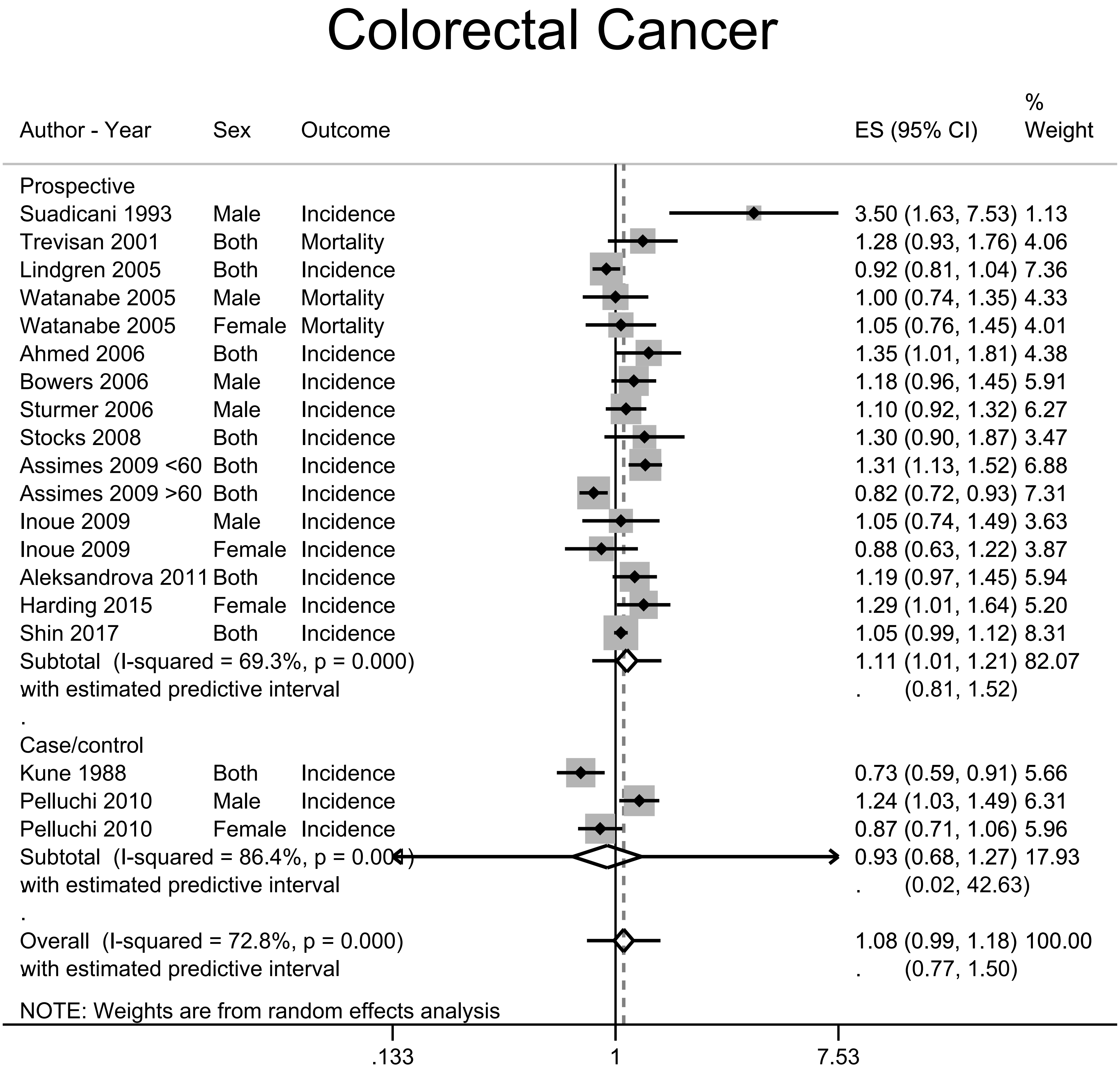

Supplement Figure 62. Meta-analysis of prospective and case-control studies for the association between hypertension and colorectal cancer risk.

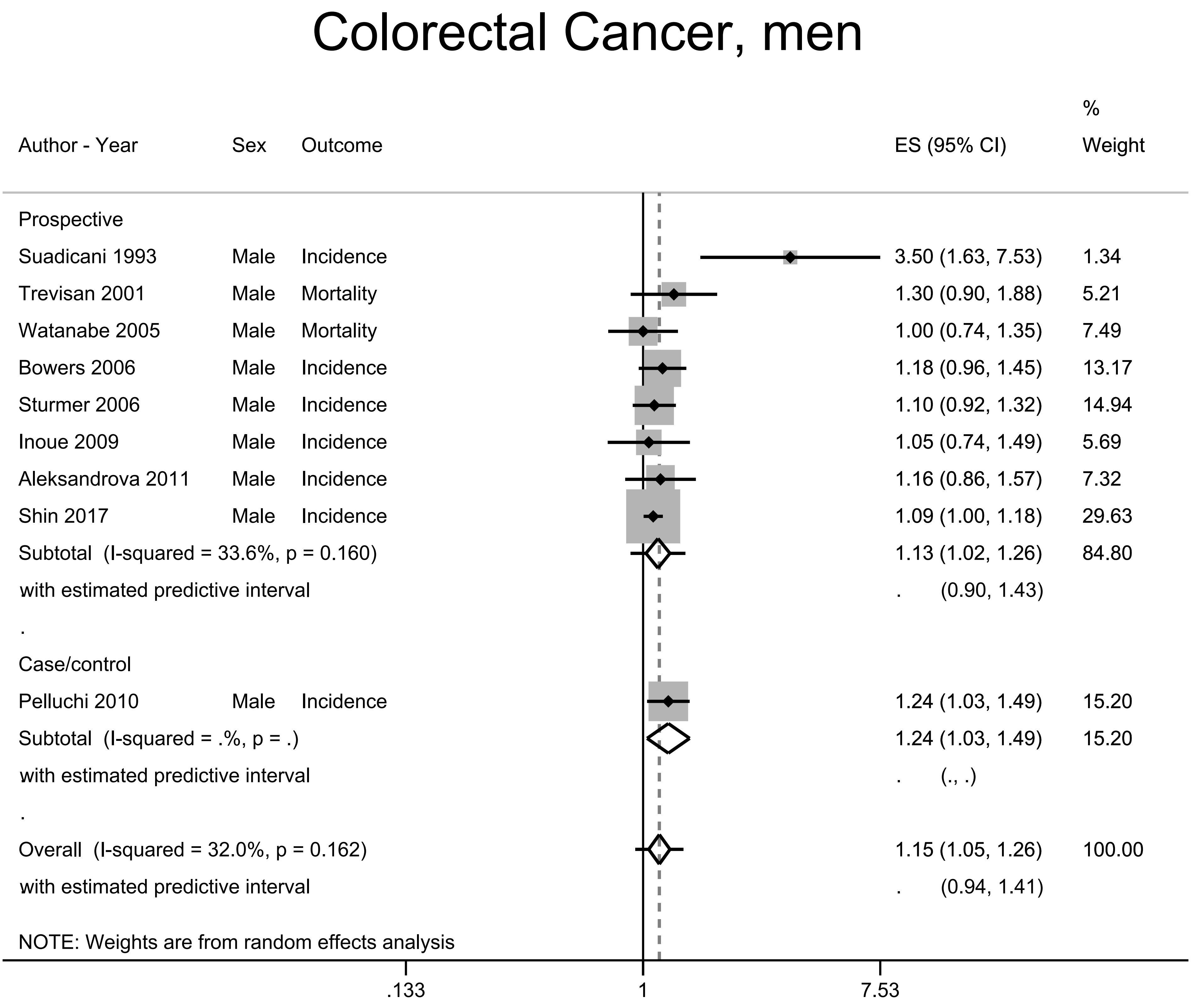

Supplement Figure 63. Meta-analysis of prospective and case-control studies for the association between hypertension and colorectal cancer risk, in men.

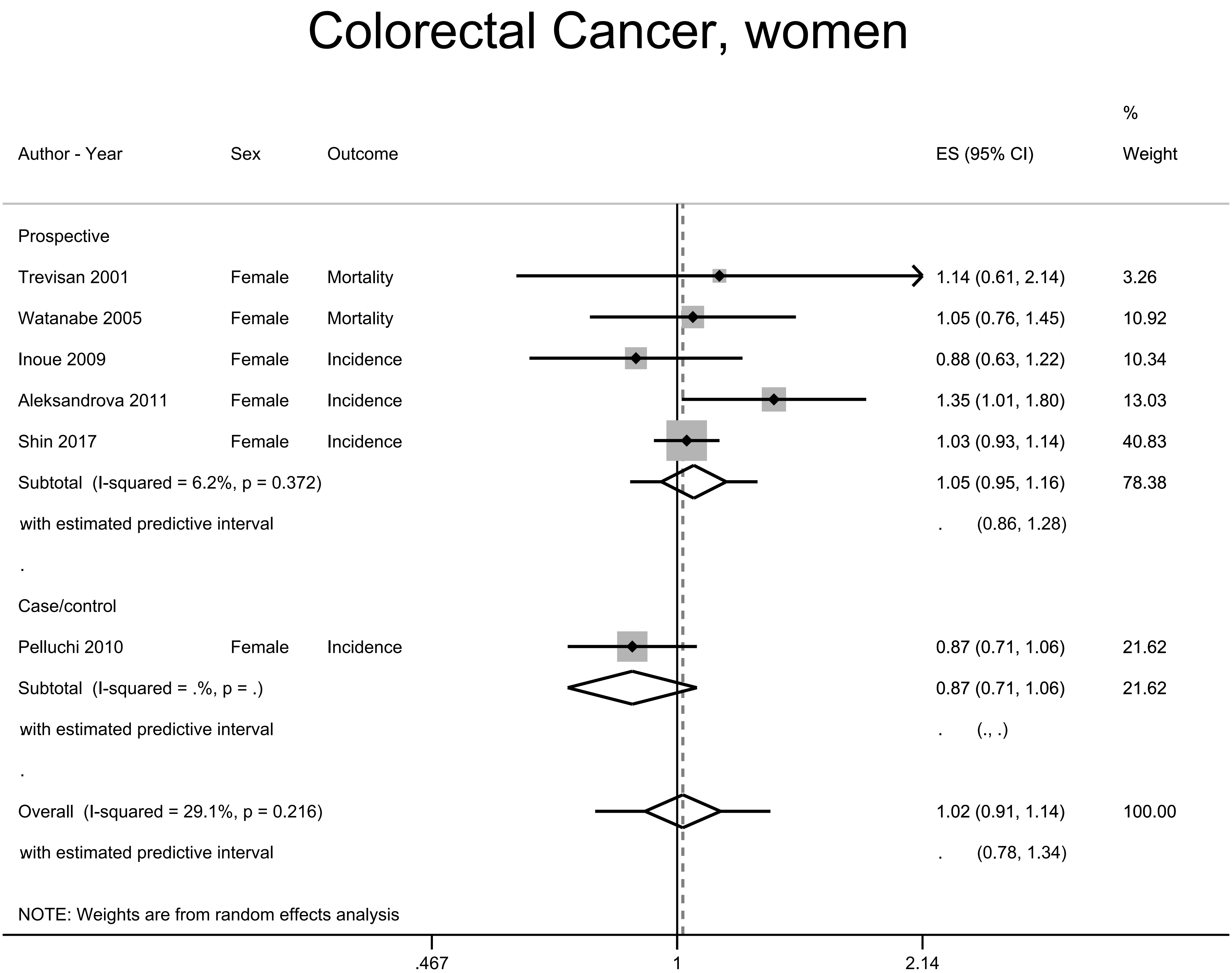

Supplement Figure 64. Meta-analysis of prospective and case-control studies for the association between hypertension and colorectal cancer risk, in women.

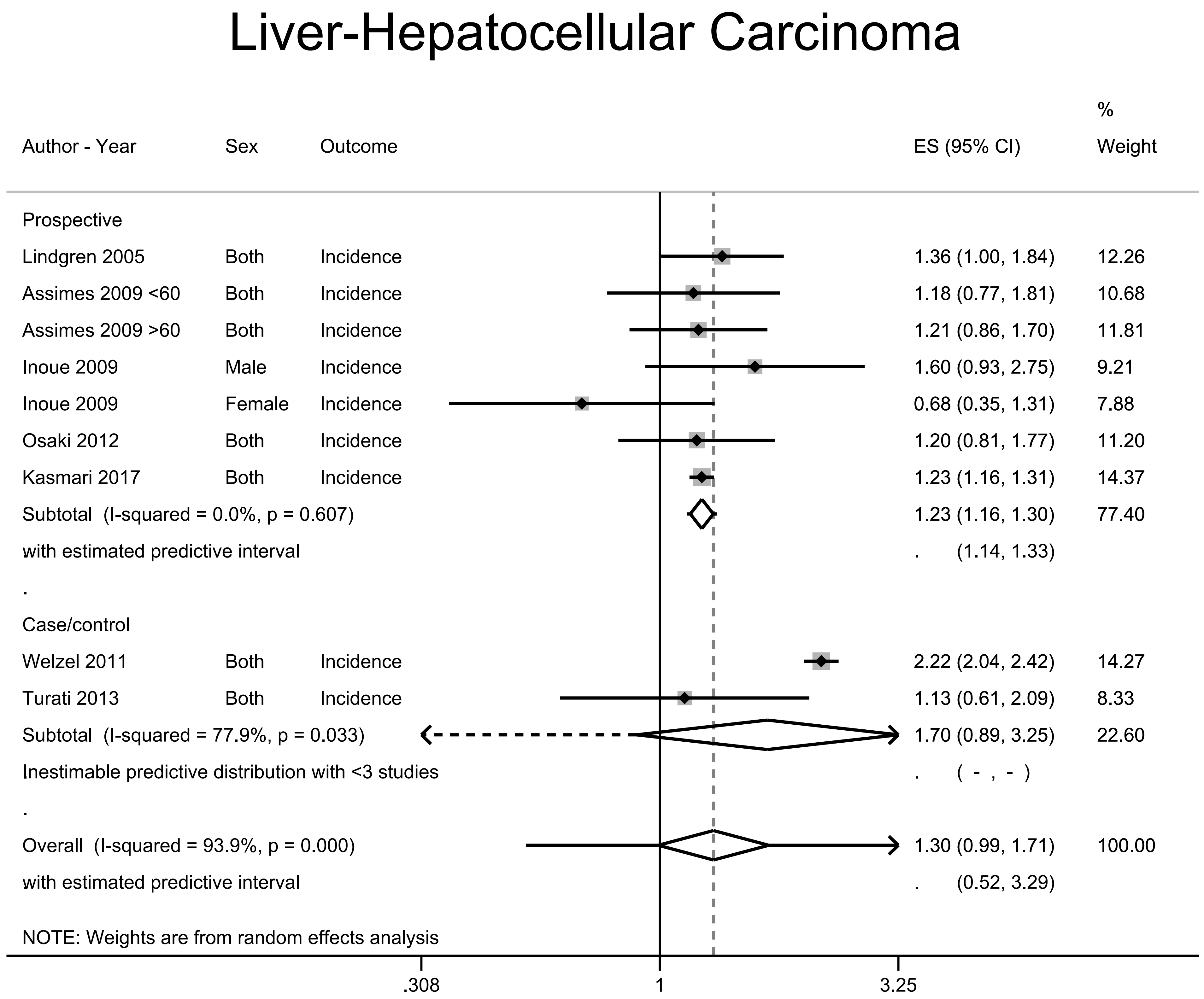

Supplement Figure 65. Meta-analysis of prospective and case-control studies for the association between hypertension and liver/hepatocellular cancer risk.

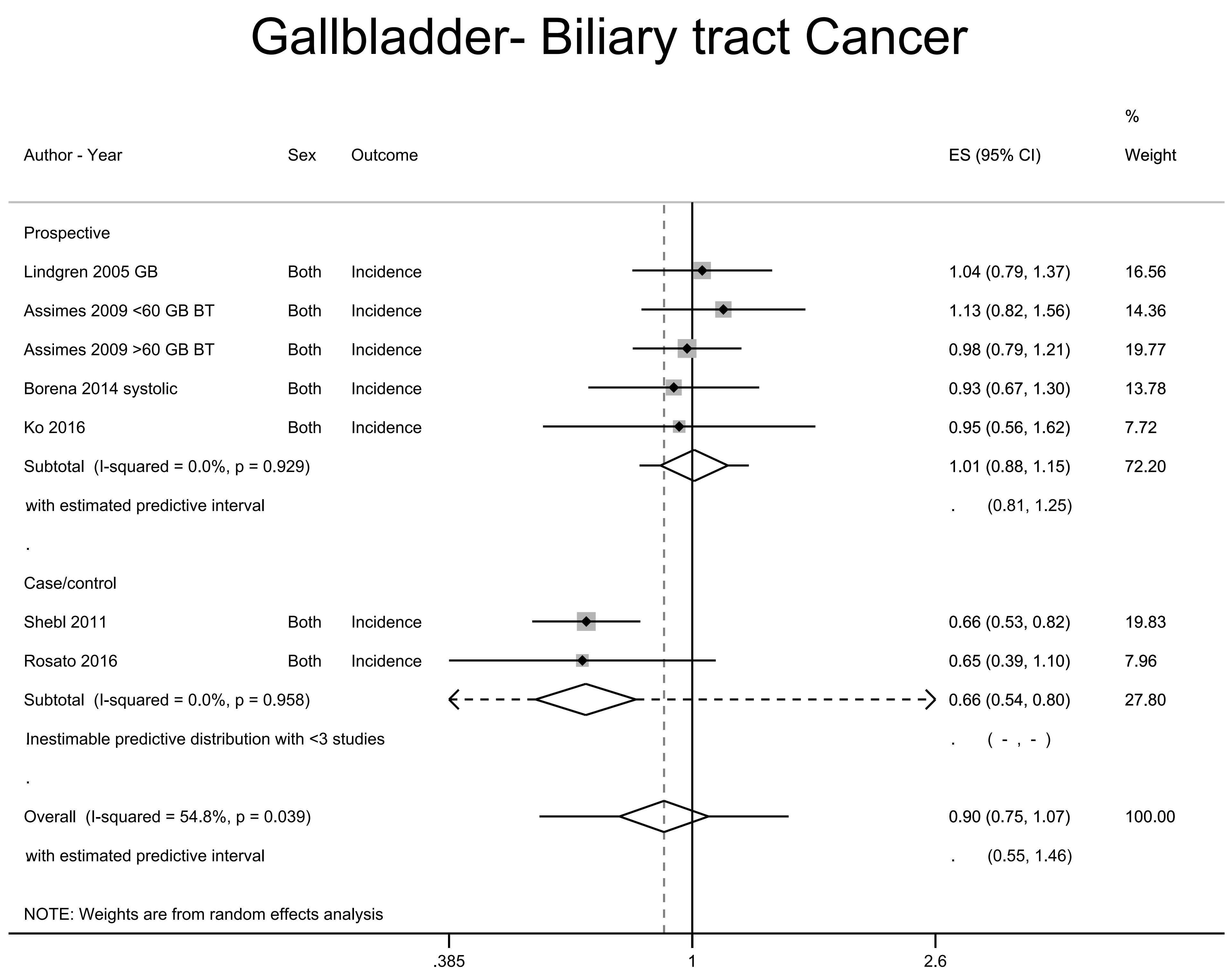

Supplement Figure 66. Meta-analysis of prospective and case-control studies for the association between hypertension and gall bladder/biliary tract cancer risk.

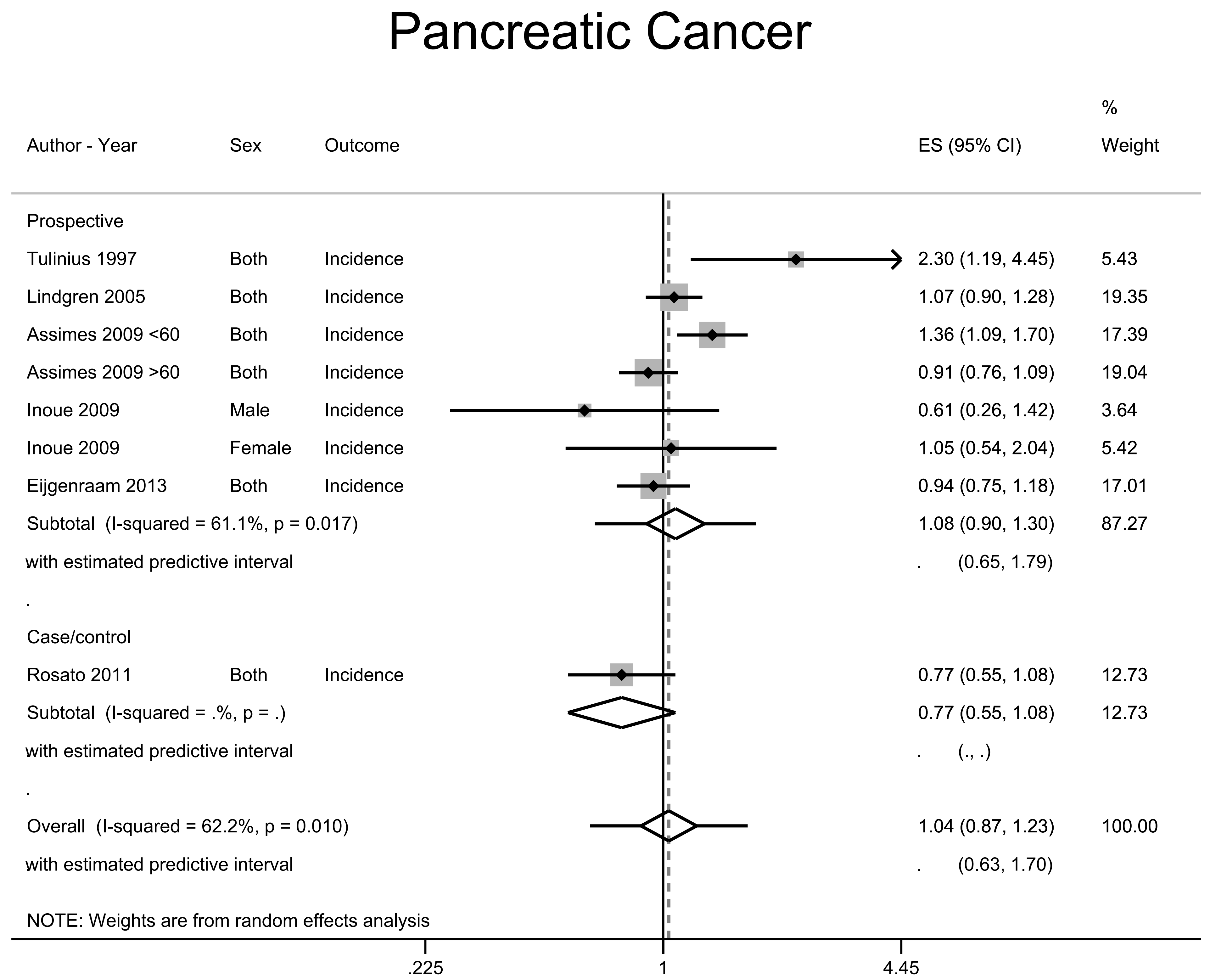

Supplement Figure 67. Meta-analysis of the association of prospective and case-control studies for the association between hypertension and pancreatic cancer risk.

Breast Cancer

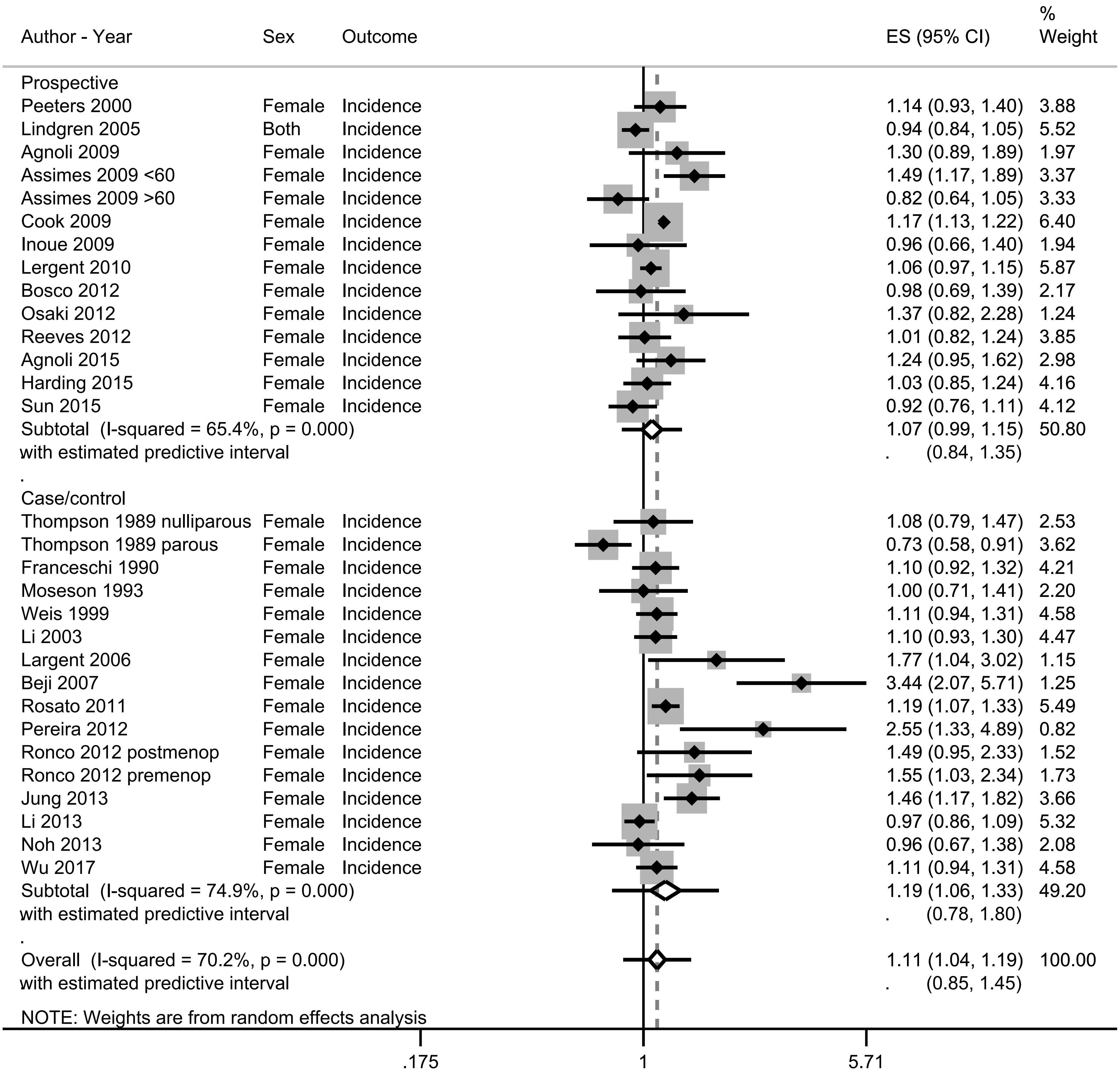

Supplement Figure 68. Meta-analysis of prospective and case-control studies for the association between hypertension and breast cancer risk.

Breast Cancer, postmenopausal

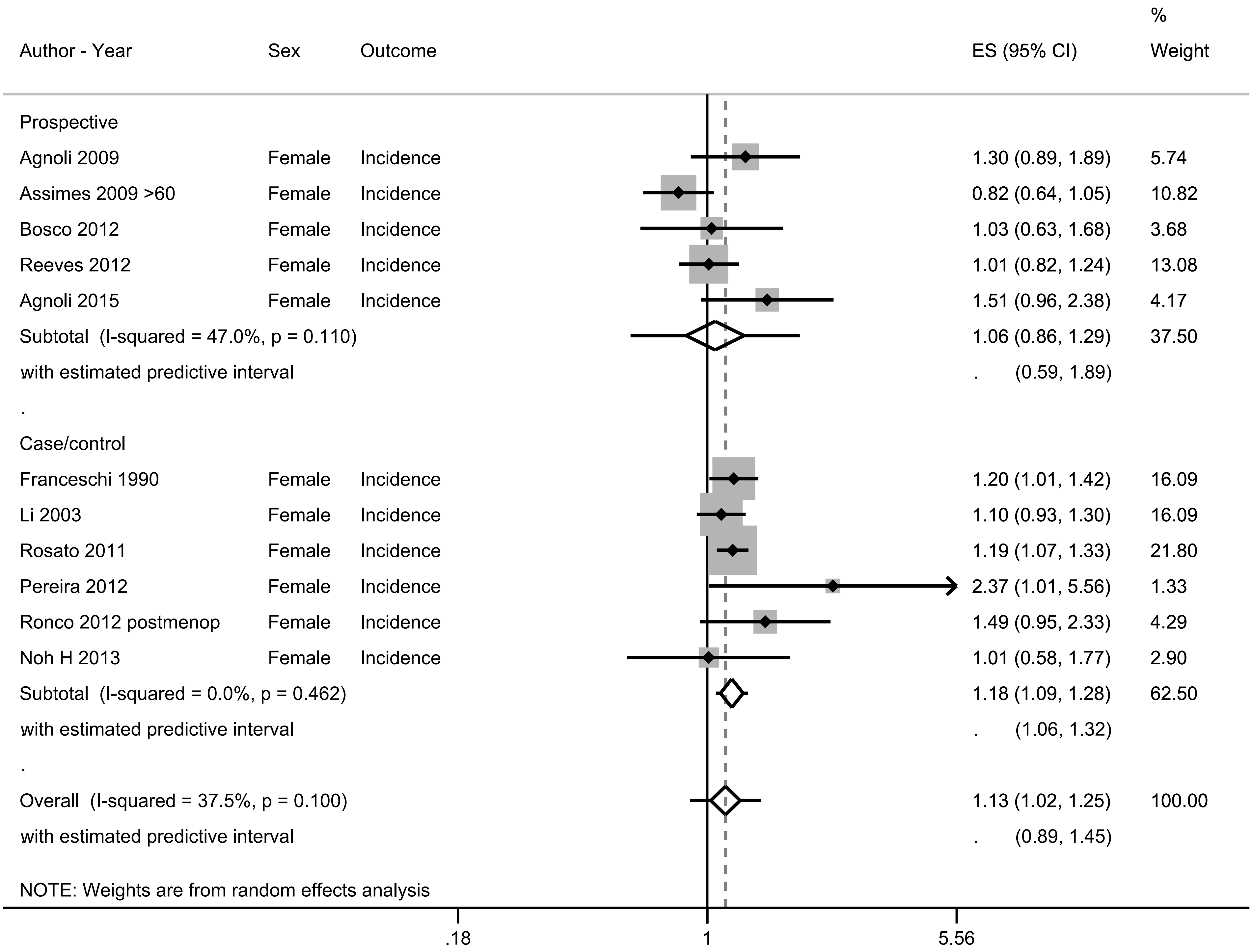

Supplement Figure 69. Meta-analysis of prospective and case-control studies for the association between hypertension and breast cancer risk, in postmenopausal women.

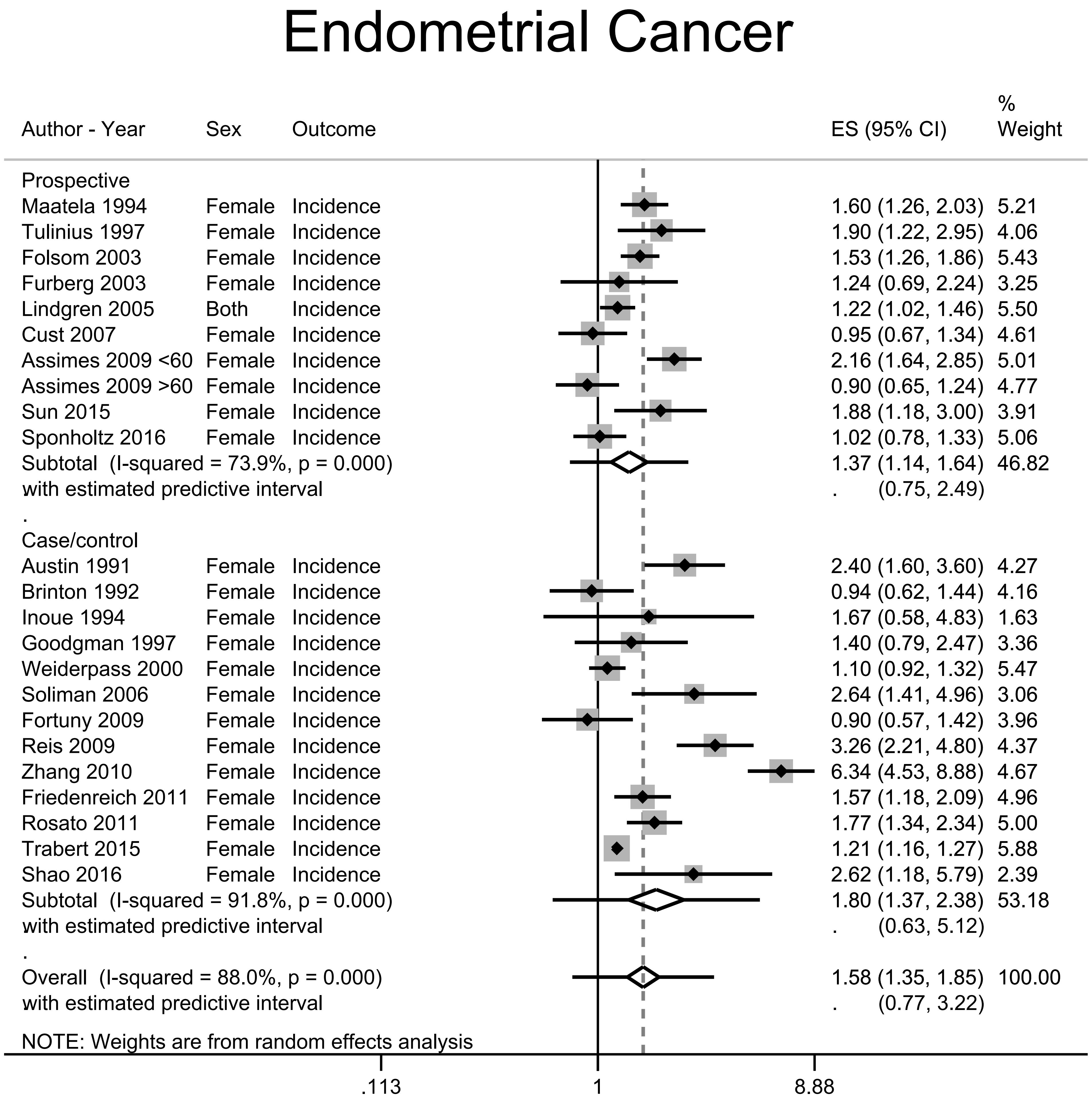

Supplement Figure 70. Meta-analysis of prospective and case-control studies for the association between hypertension and endometrial cancer risk.

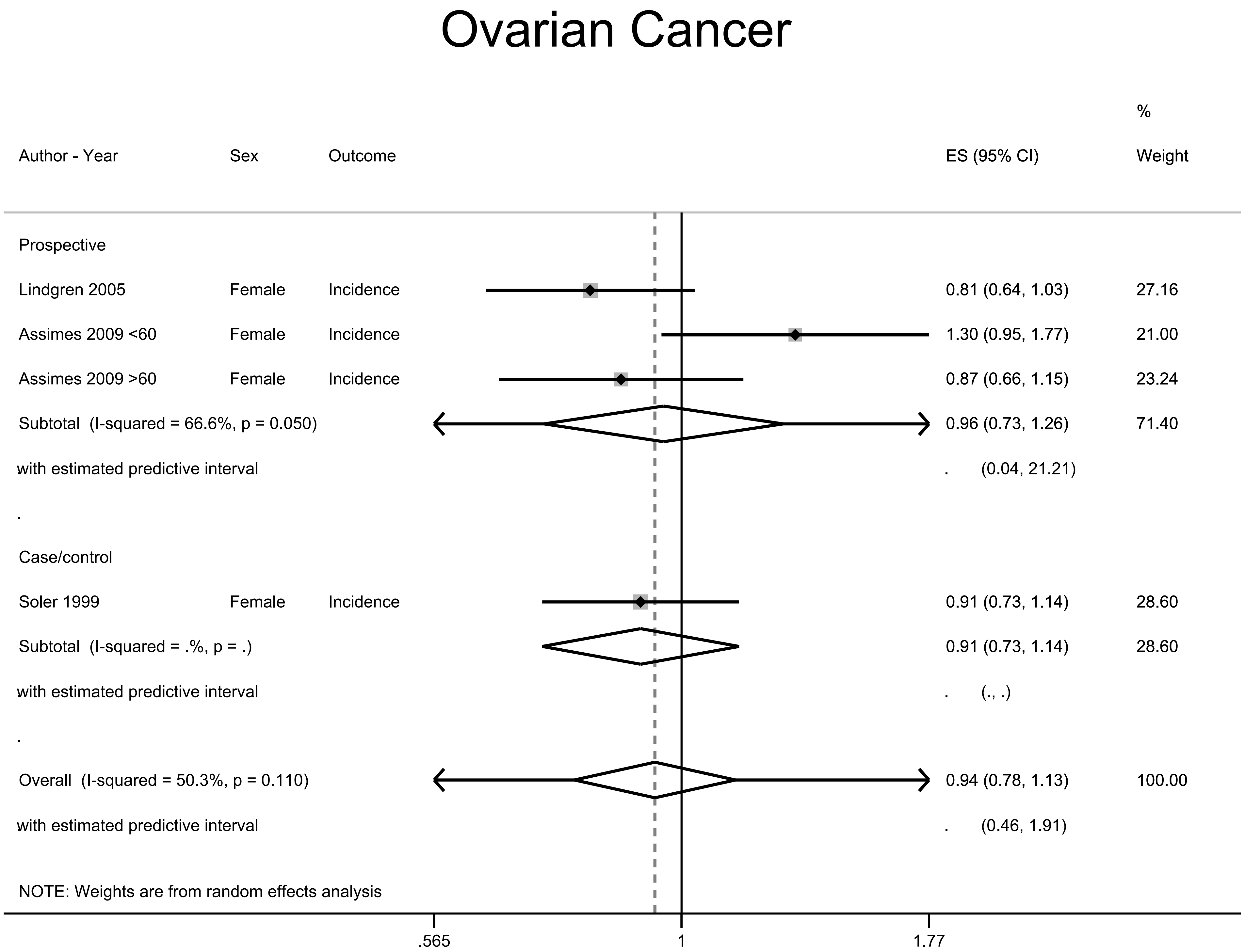

Supplement Figure 71. Meta-analysis of prospective and case-control studies for the association between hypertension and ovarian cancer risk.

Prostate Cancer

Author - Year	Sex	Outcome	ES (95% CI)	% Weight
Prospective				
Friedman 1997	Male	Incidence	1.04 (0.94, 1.16)	6.72
Tulinius 1997	Male	Incidence	1.20 (1.01, 1.42)	5.21
Fitzpatrick 2001	Male	Incidence	0.70 (0.52, 0.94)	2.95
Ronquist 2004	Male	Incidence	1.00 (0.82, 1.22)	4.46
Lindgren 2005	Both	Incidence	1.02 (0.88, 1.18)	5.69
Lund Haheim 2006	Male	Incidence	1.12 (0.74, 1.69)	1.82
Tande 2006	Male	Incidence	0.91 (0.73, 1.14)	4.07
Assimes 2009 <60	Male	Incidence	1.44 (1.09, 1.90)	3.17
Assimes 2009 >60	Male	Incidence	0.94 (0.74, 1.20)	3.69
Inoue 2009	Male	Incidence	1.21 (0.80, 1.83)	1.81
Martin 2010	Male	Incidence	0.99 (0.89, 1.11)	6.62
Stocks 2010	Male	Incidence	0.91 (0.88, 0.95)	8.07
Wallner 2010	Male	Mortality	1.10 (0.83, 1.46)	3.05
Romero 2012	Male	Incidence	1.16 (0.61, 2.22)	0.84
Lawrence 2013	Male	Incidence	1.07 (0.89, 1.28)	4.95
Harding 2015	Male	Incidence	1.05 (0.88, 1.25)	5.05
Sun 2015	Male	Incidence	1.20 (0.97, 1.48)	4.36
Subtotal (I-squared = 60.0%, p = 0.001)			1.03 (0.96, 1.10)	72.52
with estimated predictive interval			. (0.83, 1.28)	
.				
Case/control				
Oishi 1989	Male	Incidence	0.71 (0.39, 1.31)	0.94
Perron L 2004	Male	Incidence	0.98 (0.88, 1.09)	6.81
Beebe-Dimmer 2007	Male	Incidence	2.36 (1.49, 3.73)	1.53
Beebe-Dimmer 2009	Male	Incidence	1.02 (0.73, 1.43)	2.46
Weinmann 2010	Male	Mortality	1.20 (0.99, 1.46)	4.64
Ganesh 2011	Male	Incidence	2.80 (1.20, 6.52)	0.52
Pelluchi 2011	Male	Incidence	1.14 (0.96, 1.36)	5.08
Lapierre 2015	Male	Incidence	0.93 (0.80, 1.09)	5.49
Subtotal (I-squared = 72.5%, p = 0.001)			1.13 (0.96, 1.33)	27.48
with estimated predictive interval			. (0.70, 1.82)	
.				
Overall (I-squared = 65.8%, p = 0.000)			1.05 (0.99, 1.12)	100.00
with estimated predictive interval			. (0.83, 1.33)	

NOTE: Weights are from random effects analysis

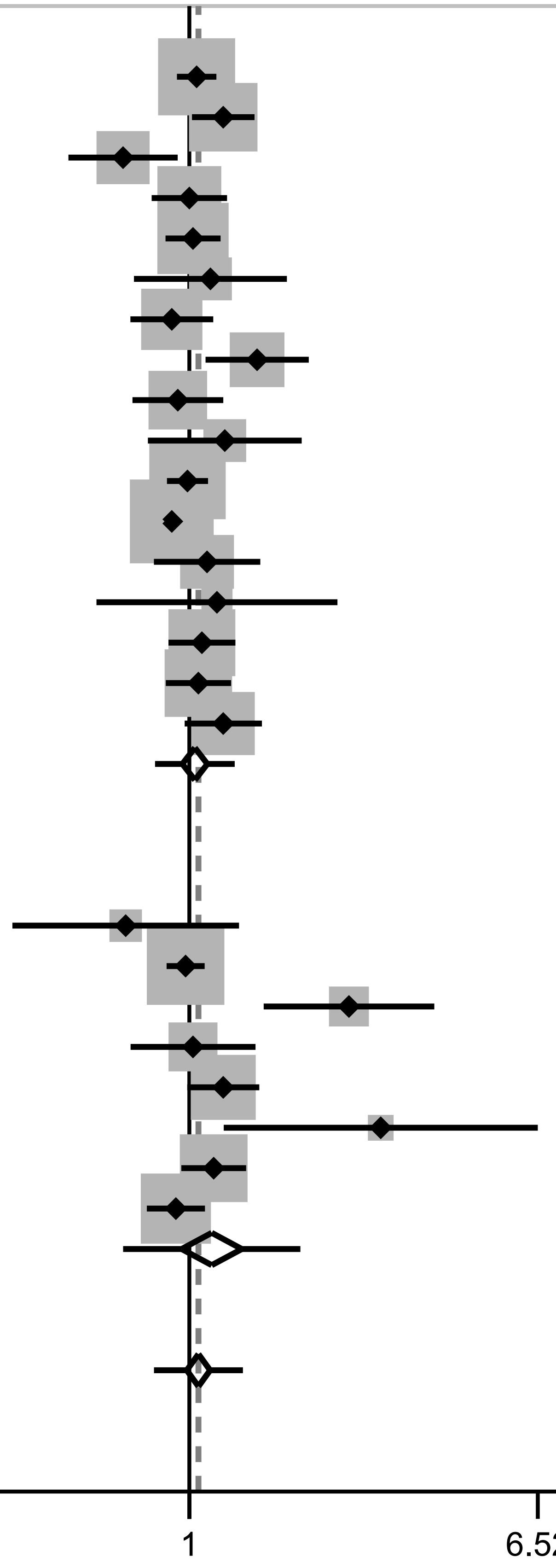

Supplement Figure 72. Meta-analysis of prospective and case-control studies for the association between hypertension and prostate cancer risk.

Kidney-Renal Cancer

Author - Year	Sex	Outcome	ES (95% CI)	% Weight
Prospective				
Fraser 1990	Both	Incidence	2.90 (0.94, 8.94)	0.43
Heath 1997	Male	Mortality	1.10 (0.85, 1.42)	3.22
Heath 1997	Female	Mortality	2.20 (1.51, 3.21)	2.27
Nicodemus 2004	Female	Incidence	1.68 (1.18, 2.39)	2.44
Choi 2005	Male	Mortality	1.72 (0.66, 4.49)	0.58
Flaherty 2005	Male	Incidence	1.80 (1.20, 2.70)	2.10
Flaherty 2005	Female	Incidence	1.90 (1.37, 2.64)	2.62
Fryzek 2005	Both	Incidence	1.60 (1.32, 1.93)	3.82
Lindgren 2005	Both	Incidence	1.34 (1.12, 1.61)	3.88
Schouten 2005	Both	Incidence	1.22 (0.94, 1.58)	3.18
Setiawan 2007	Male	Incidence	1.42 (1.07, 1.88)	3.01
Setiawan 2007	Female	Incidence	1.58 (1.09, 2.29)	2.33
Vatten 2007	Male	Incidence	1.10 (0.64, 1.91)	1.42
Vatten 2007	Female	Incidence	1.40 (0.84, 2.32)	1.59
Weikert 2007	Both	Incidence	1.34 (0.97, 1.85)	2.68
Assimes 2009 <60	Both	Incidence	1.93 (1.58, 2.35)	3.75
Assimes 2009 >60	Both	Incidence	1.17 (0.95, 1.44)	3.65
Mcleod 2013	Both	Incidence	1.70 (1.30, 2.22)	3.11
Washio 2013		Mortality	1.40 (0.85, 2.30)	1.63
Sanfillipo 2014	Female	Incidence	1.56 (1.06, 2.29)	2.23
Hofmann 2015	Both	Incidence	2.10 (1.87, 2.36)	4.45
Shen 2015	Both	Incidence	1.40 (1.07, 1.84)	3.06
Sun 2015	Both	Incidence	1.56 (1.13, 2.15)	2.67
Subtotal (I-squared = 63.1%, p = 0.000)			1.54 (1.39, 1.70)	60.14
with estimated predictive interval			. (1.04, 2.28)	
.				
Case/control				
Kreiger N 1992	Male	Incidence	1.40 (0.83, 2.37)	1.50
Kreiger N 1992	Female	Incidence	1.30 (0.72, 2.36)	1.26
McCreadie 1992	Both	Incidence	1.80 (1.35, 2.39)	2.96
Finkle 1993	Female	Incidence	1.10 (0.62, 1.96)	1.32
Mellemgaard 1994	Male	Incidence	1.80 (1.10, 2.96)	1.63
Mellemgaard 1994	Female	Incidence	1.80 (1.06, 3.07)	1.48
Weinmann 1994	Male	Incidence	2.00 (1.14, 3.52)	1.37
Chow W 1995	Both	Incidence	1.50 (1.14, 1.97)	3.06
McLaughlin 1995	Both	Incidence	1.40 (1.18, 1.67)	3.96
Yuan 1998	Both	Incidence	2.20 (1.83, 2.64)	3.87
Shapiro 1999	Male	Incidence	1.30 (0.69, 2.46)	1.14
Shapiro 1999	Female	Incidence	2.50 (1.21, 5.15)	0.93
Zucchetto 2006	Both	Incidence	1.74 (1.42, 2.13)	3.72
Brennan 2008	Both	Incidence	1.25 (1.05, 1.48)	4.00
Colt 2011	Both	Incidence	2.00 (1.65, 2.43)	3.79
Wang 2012	Both	Incidence	2.68 (1.84, 3.90)	2.29
Lai 2013	Both	Incidence	2.05 (1.23, 3.42)	1.57
Subtotal (I-squared = 60.2%, p = 0.001)			1.70 (1.50, 1.93)	39.86
with estimated predictive interval			. (1.12, 2.58)	
.				
Overall (I-squared = 61.3%, p = 0.000)			1.60 (1.48, 1.73)	100.00
with estimated predictive interval			. (1.11, 2.32)	

NOTE: Weights are from random effects analysis

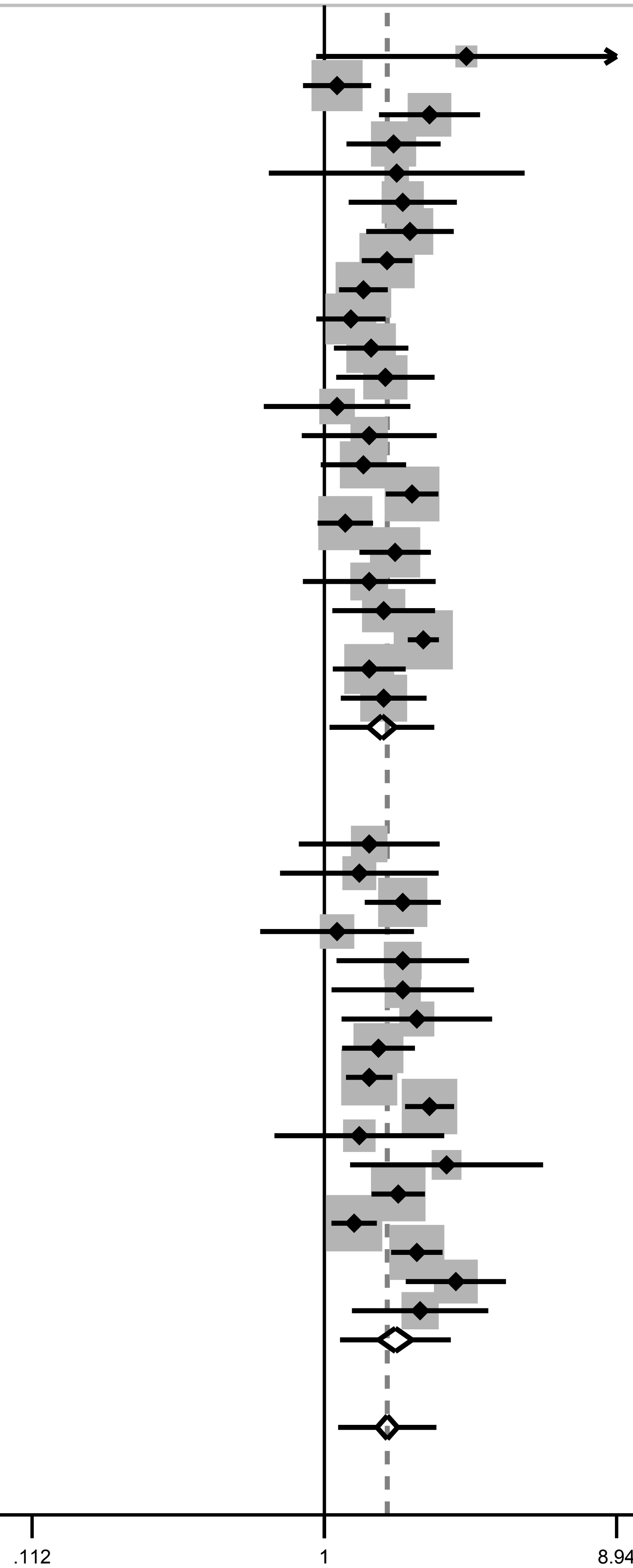

Supplement Figure 73. Meta-analysis of prospective and case-control studies for the association between hypertension and kidney/renal cancer risk.

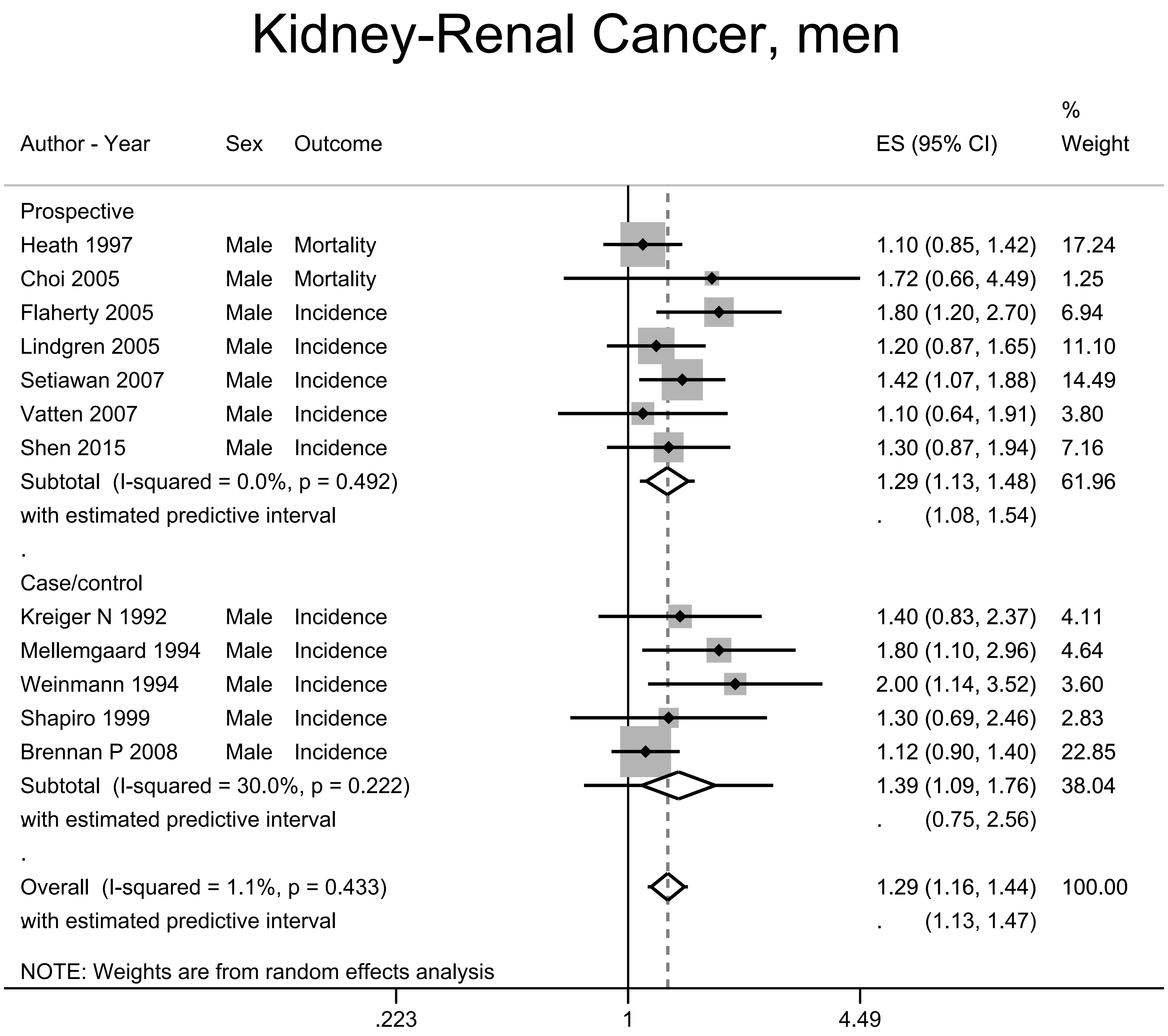

Supplement Figure 74. Meta-analysis of prospective and case-control studies for the association between hypertension and kidney/renal cancer risk, in men.

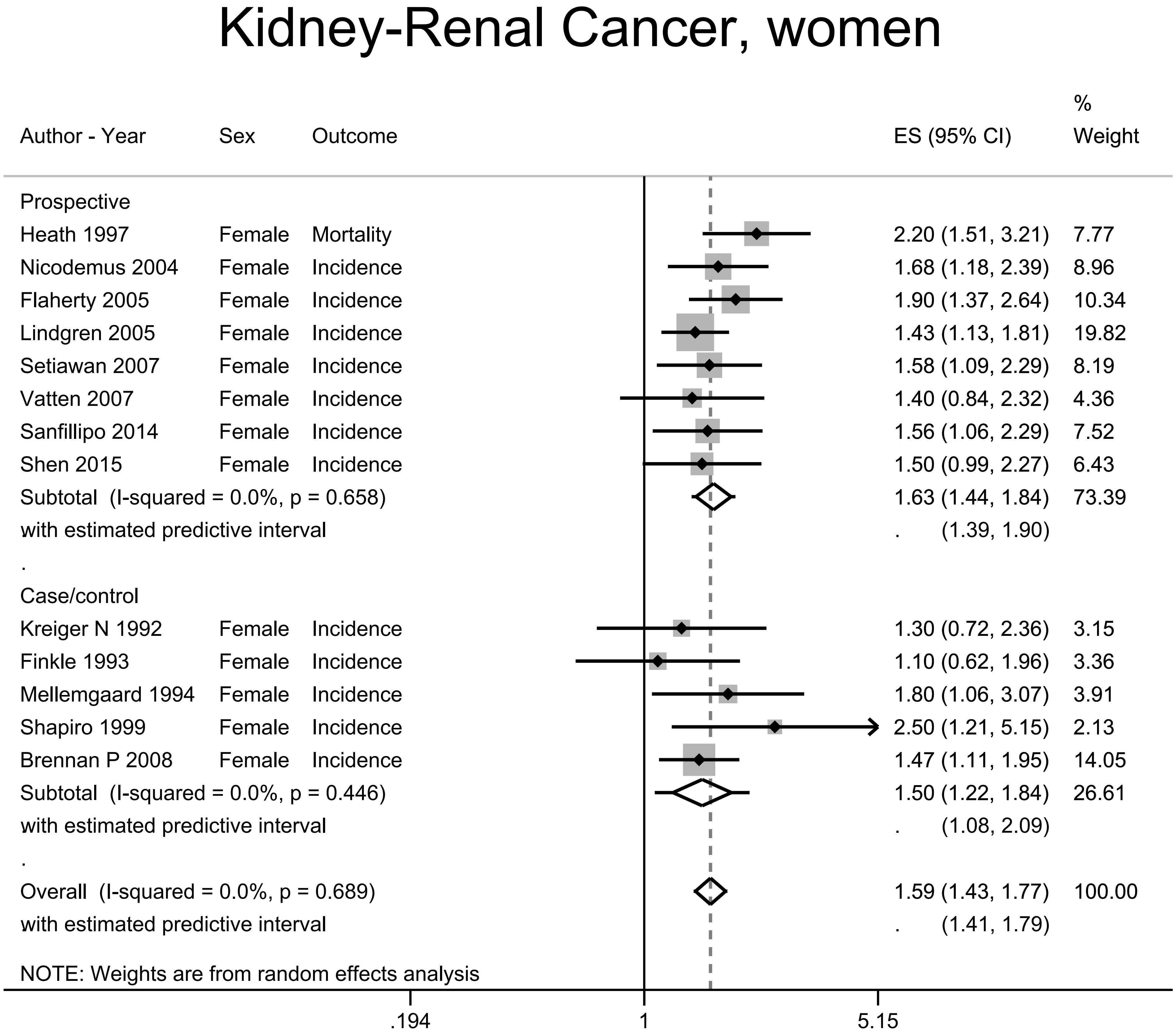

Supplement Figure 75. Meta-analysis of prospective and case-control studies for the association between hypertension and kidney/renal cancer risk, in women.

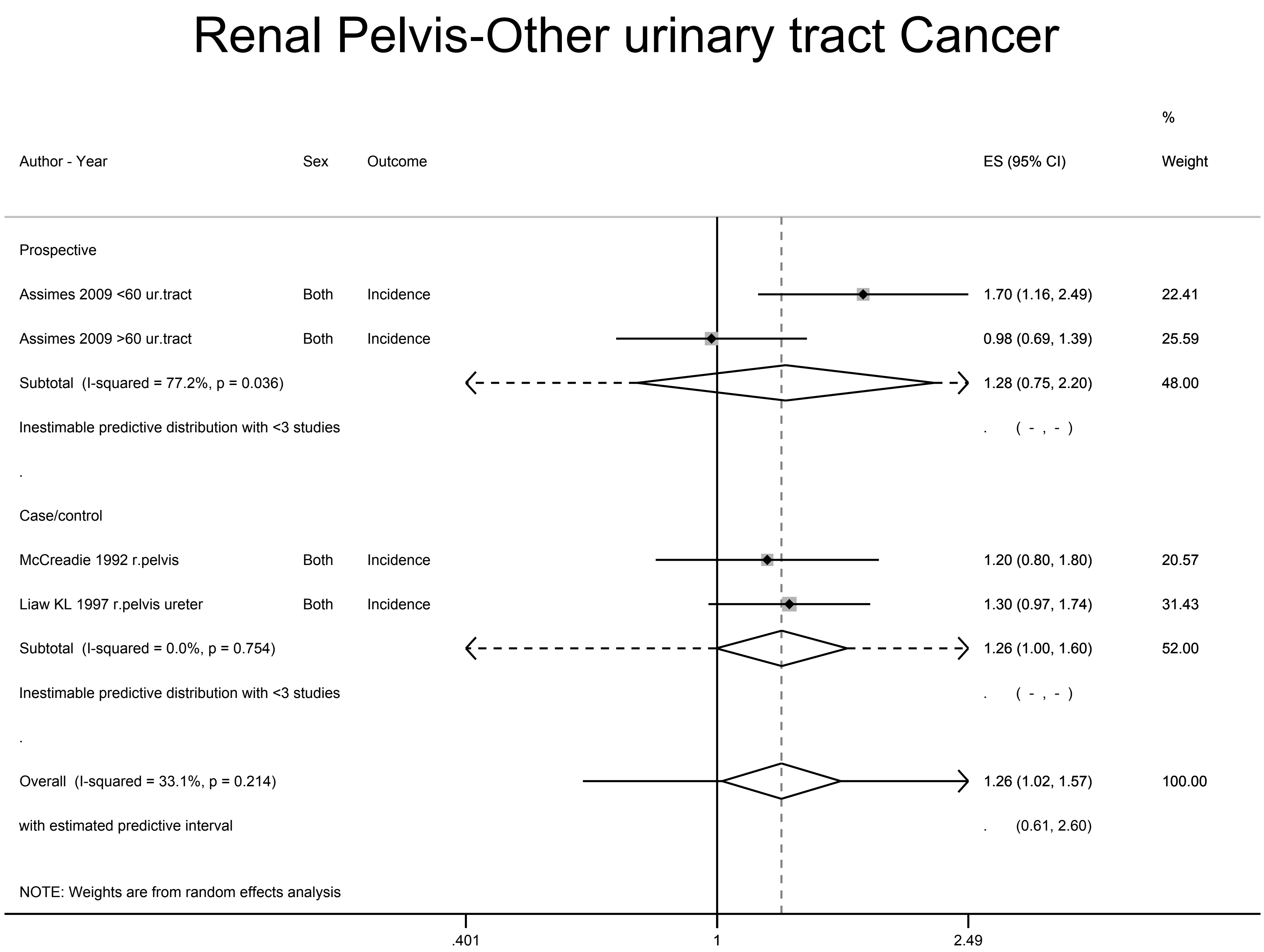

Supplement Figure 76. Meta-analysis of prospective and case-control studies for the association between hypertension and renal pelvis/urinary tract cancer risk.

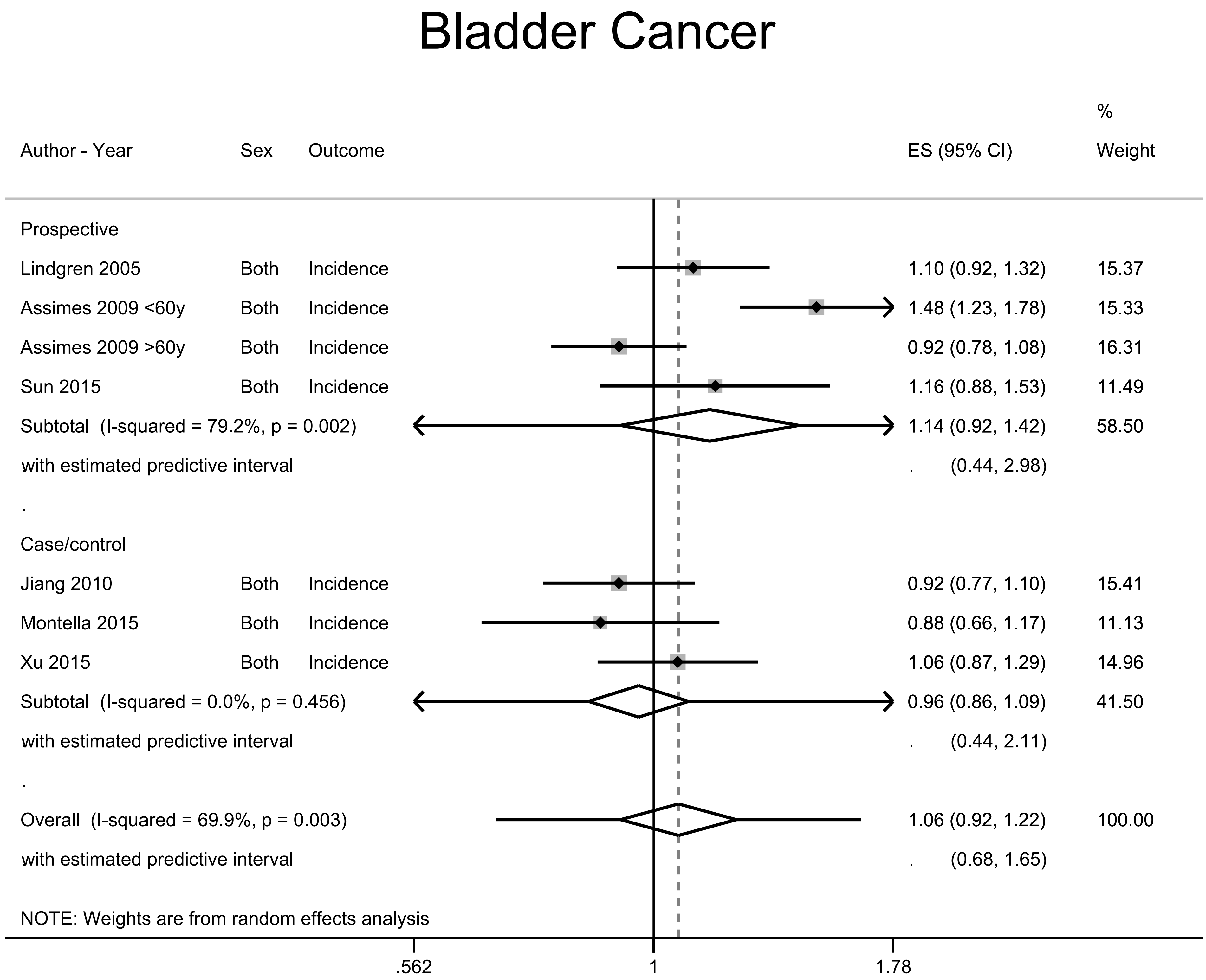

Supplement Figure 77. Meta-analysis of prospective and case-control studies for the association between hypertension and bladder cancer risk.

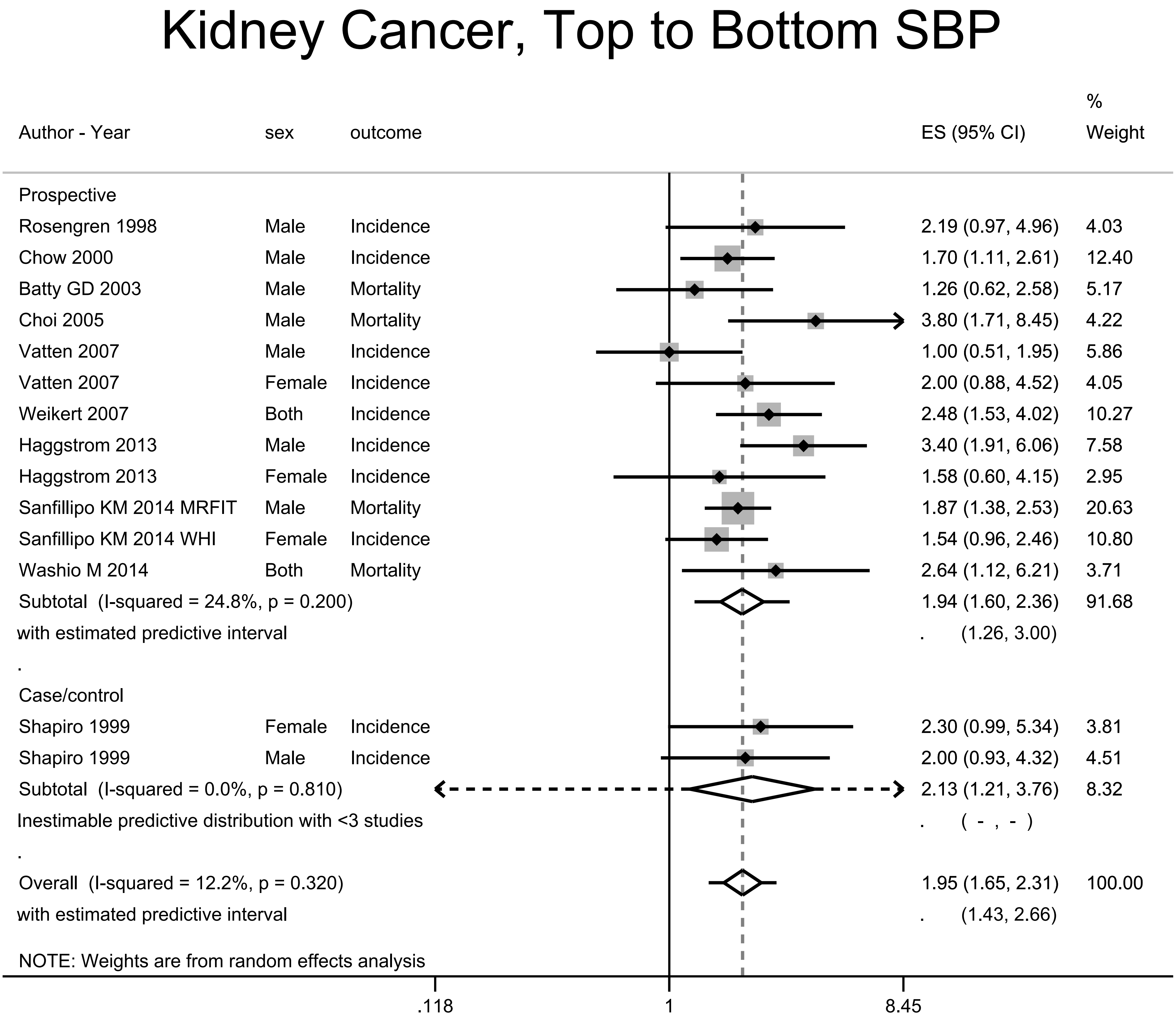

Supplement Figure 78. Meta-analysis of prospective and case-control studies for the association between systolic blood pressure (Top vs Bottom) and kidney cancer risk. Abbreviations: SBP, systolic blood pressure.

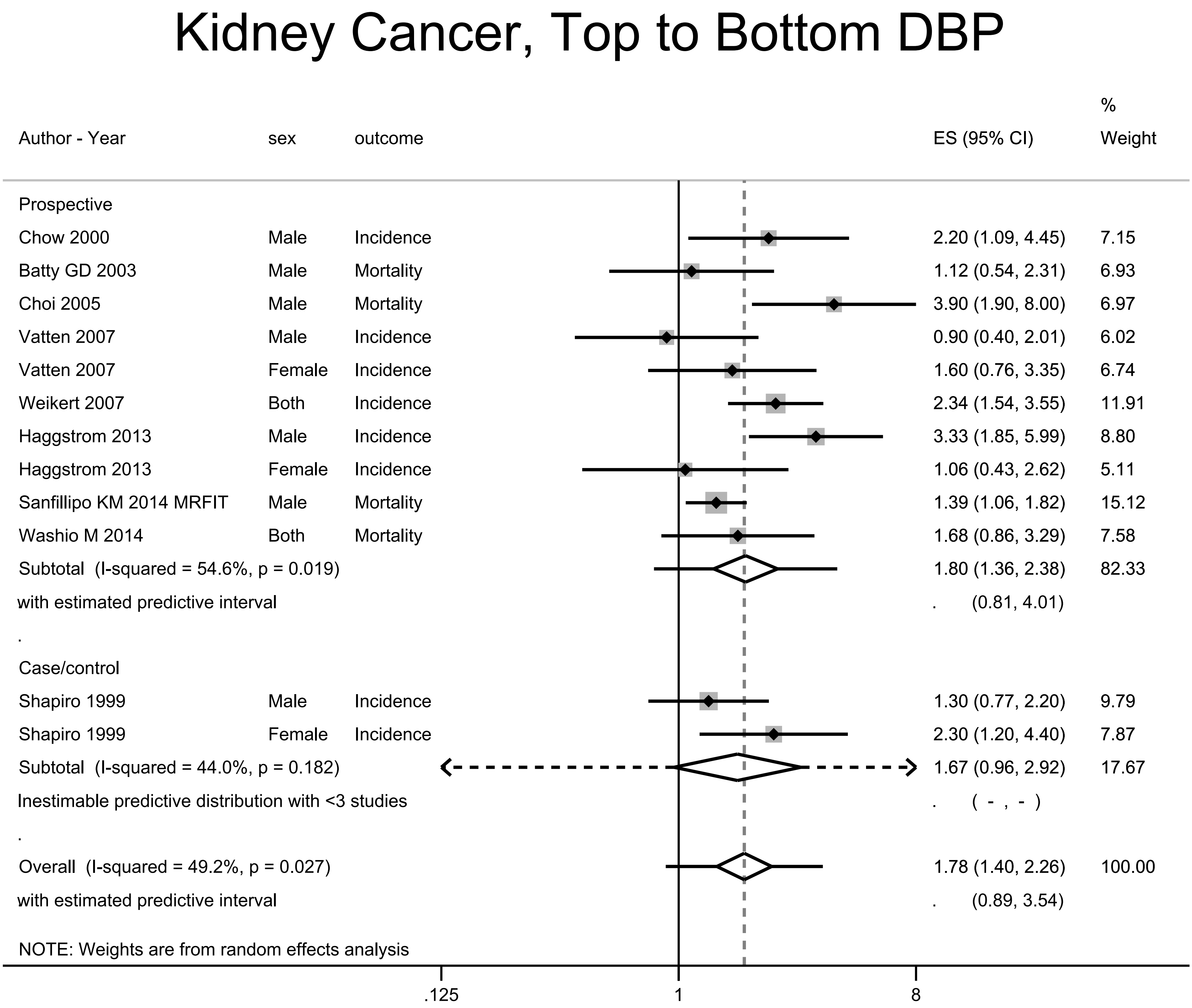

Supplement Figure 79. Meta-analysis of prospective and case-control studies for the association between diastolic blood pressure (Top vs Bottom) and kidney cancer risk. Abbreviations: DBP, diastolic blood pressure.
